# Synthesis and Characterization of Photoswitchable Covalent Ligands for the β_2_‐Adrenoceptor

**DOI:** 10.1002/anie.202424038

**Published:** 2025-04-10

**Authors:** Ulrike Wirth, Eduard Neu, Davide Provasi, Steffen Feustel, Maximilian F. Schmidt, Harald Hübner, Dorothée Weikert, Marta Filizola, Burkhard König, Peter Gmeiner

**Affiliations:** ^1^ Faculty of Chemistry and Pharmacy University of Regensburg 93053 Regensburg Germany; ^2^ Department of Chemistry and Pharmacy Friedrich‐Alexander‐Universität Erlangen‐Nürnberg 91058 Erlangen Germany; ^3^ FAU NeW 91058 Erlangen Germany; ^4^ Department of Pharmacological Sciences Icahn School of Medicine at Mount Sinai New York NY USA

**Keywords:** Computational chemistry, Covalent ligand, GPCR, Medicinal chemistry, Photopharmacology

## Abstract

The β_2_‐adrenergic receptor (β_2_AR) is a critical target for the treatment of airway diseases such as asthma or chronic obstructive pulmonary disease (COPD). To better understand its mechanism of action and study its dynamics, photoswitchable ligands provide a distinct advantage due to their ability to be activated with high spatiotemporal control. In this study, we developed a series of molecular tools featuring different combinations of pharmacophores, covalent warheads, and linker lengths. These compounds were characterized using highly specific assay protocols to evaluate their covalent binding capabilities, with the goal of identifying optimal covalently‐bound photoswitches. Among these, the covalently‐bound photoswitchable receptor agonist **6** exhibited a significant functional switch. To gain deeper insight into the binding thermodynamics and kinetics of this molecular tool, as well as the molecular determinants involved, we performed metadynamics (MetaD) simulations and analyzed their results using a Markov State Model (MSM) obtained by applying the Maximum Caliber (MaxCal) principle to the MetaD‐derived free energies. These studies suggest that photoswitching of compound **6** occurs within the binding pocket of the β_2_AR. Consequently, compound **6** holds promise as a valuable tool for investigating β_2_AR activation kinetics and dynamics with high accuracy, thereby facilitating high‐resolution biophysical studies.

## Graphical abstract

A structure‐based design of a covalent photoswitchable ligand for the β_2_‐adrenergic receptor, a therapeutically relevant GPCR, is described. This tool facilitates the modification of the intrinsic activity of the protein by light. Computational analysis of its mechanism of action suggests that the photoswitch takes place within the binding pocket.

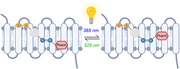

## Introduction

Significant strides have been made in the structural elucidation of G protein‐coupled receptors (GPCRs) by X‐ray crystallography and cryo‐electron microscopy (cryo‐EM). However, these techniques typically yield static images of specific receptor states. The dynamic nature of ligand‐induced conformational changes in GPCRs remains elusive, and understanding the binding kinetics of ligands is critical to deciphering the functional signaling mechanisms mediated by these receptors.^[^ ^1^ ^]^ Although biological assays are effective in measuring a ligand's residence time,^[^ ^2^, ^3^ ^]^ computer simulations can offer unique atomic‐level insights into ligand binding kinetics.

To investigate GPCR activation dynamics and signal transduction independently of ligand association and dissociation, a ligand with very slow off‐kinetics that can alter its intrinsic activity via external control is essential.^[^ ^4^ ^]^ Photoswitchable ligands that are covalently bound to the target receptor meet these criteria. These ligands can bind directly to the receptor through an engineered mutation,^[^ ^5^ ^]^ a fusion protein,^[^ ^6^ ^]^ or attachment to a membrane‐anchoring protein in close proximity to the receptor.^[^ ^7^ ^]^ Photoswitches are molecular structures that can be controlled by external light stimuli, which alter a molecule's geometry and, consequently, its functional properties. These structures enable rapid and bistable photoswitching of ligands between different intrinsic activities.^[^ ^8^, ^9^ ^]^

Photoswitchable covalent ligands will be extremely helpful tools to deepen our molecular understanding of GPCR function and signaling. Ligands that can be actively switched between an “on” and “off” state while being bound within a receptor's binding pocket could provide several advantages over a conventional test set of agonists, antagonists, or biased ligands. The covalently bound ligand enables the optical control of the receptor activation state with high spatiotemporal precision, making it ideally suited for kinetic studies. The permanent presence of the ligand disentangles the signaling process from underlying ligand dissociation and association processes that may obscure the kinetics of the actual signal transduction events. This will be helpful for example in single‐molecule microscopy studies or for biochemical approaches studying conformational changes. Importantly, photoswitching between a biased‐ and non‐biased receptor activation state of a covalently bound ligand may help to unravel the molecular determinants and mechanisms underlying functional selectivity, which remain largely unknown, despite considerable scientific efforts.

Moreover, the cooperative effects between an orthosteric photoswitchable ligand and allosteric modulators for the different receptor activation states may be directly studied in an identical cellular test system, again allowing to precisely study their molecular mechanism of (inter)action. Photoswitchable allosteric modulators may be of interest to study the modulation of the signaling of endogenous ligands, especially in more complex in vitro test systems, e.g. organoids that retain a tonic signaling activity. In fact, the in vivo applicability of real‐time optical control of GPCR signaling by photoswitchable ligands has already been demonstrated in zebrafish^[^ ^10^ ^]^ and mice.^[^ ^11^ ^]^

Based on this principle, reversibly binding photochromic ligands have been developed for various GPCRs, including CXCR3,^[^ ^12^ ^]^ μOR,^[^ ^13^ ^]^ mGlu_5_,^[^ ^14^ ^]^ CB_1_,^[^ ^15^ ^]^ H_3_R,^[^ ^16^ ^]^ α_2A_AR,^[^ ^17^ ^]^ and Y_4_R.^[^ ^18^ ^]^ A photoswitchable antagonist has also been reported for the β_2_AR, an important therapeutic drug target for the treatment of airway diseases such as asthma or chronic obstructive pulmonary disease (COPD).^[^ ^19^ ^]^

**Figure 1:**
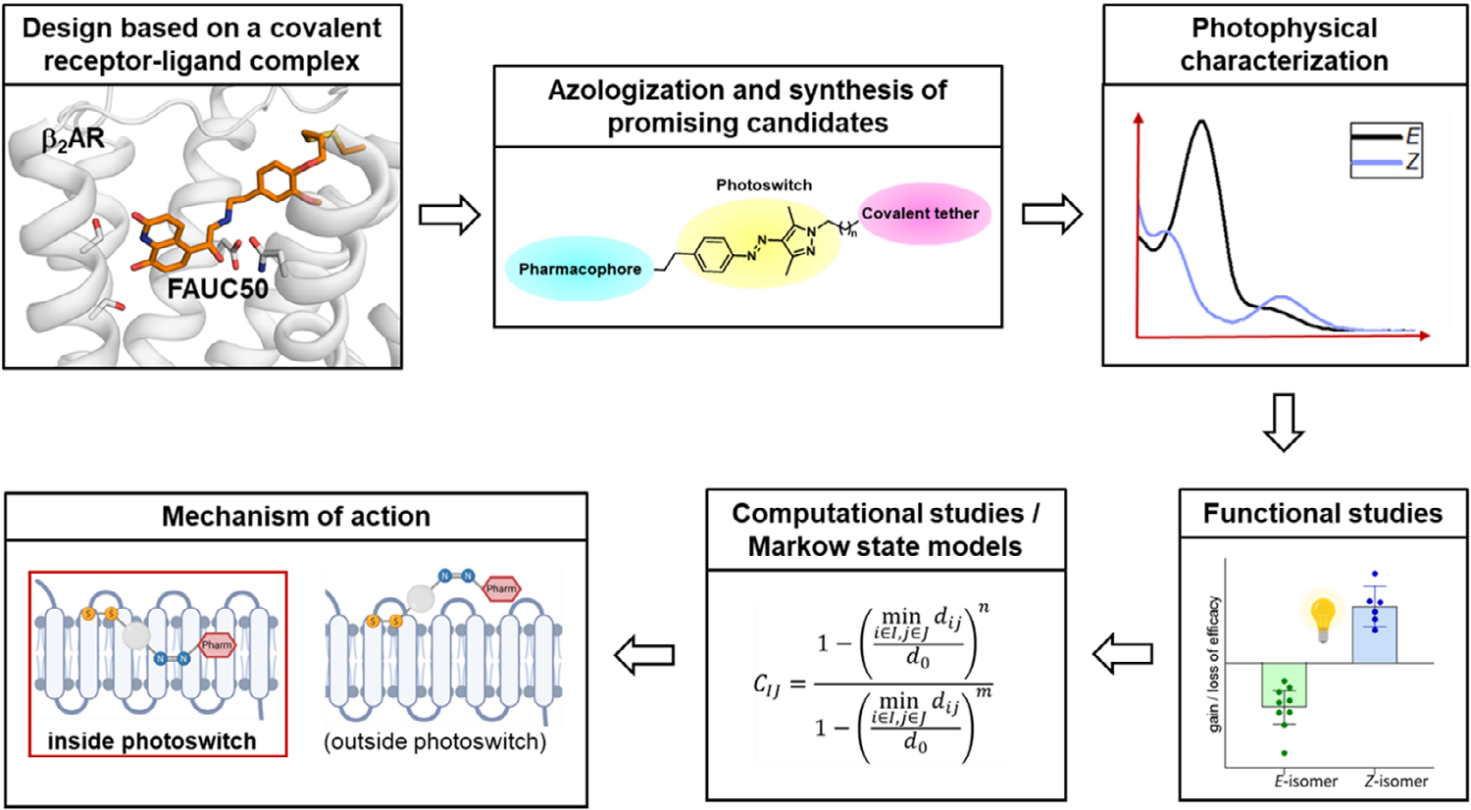
Structure‐based generation and biomolecular investigation of a covalent photoswitchable β_2_AR ligand.

In this study, we present the structure‐based generation of a covalent photoswitchable β_2_AR ligand and the investigation of its mechanism of action (Figure 1). We synthesized nine covalently binding photoswitchable ligands for the β_2_AR, among which compound **6** emerged as a promising candidate. Interestingly, compound **6** complements tethered photoswitches commonly used for ligand‐gated ion channels^[^ ^20^ ^]^ and family C GPCRs.^[^ ^21^ ^]^ These systems often feature long linkers to bridge significant distances between the protein anchor and the binding site, allowing the ligand to potentially disengage from the receptor before, during, or after photoswitching. We offer insights into the photoswitching mechanism of compound **6** by providing an atomic‐level understanding of its binding kinetics, achieved through metadynamics (MetaD) simulations analyzed using Markov State Models (MSMs) and the Maximum Caliber (MaxCal) principle. The data suggest that compound **6** undergoes photoswitching within the binding pocket of the β_2_AR.

## Results

### Design

**Figure 2:**
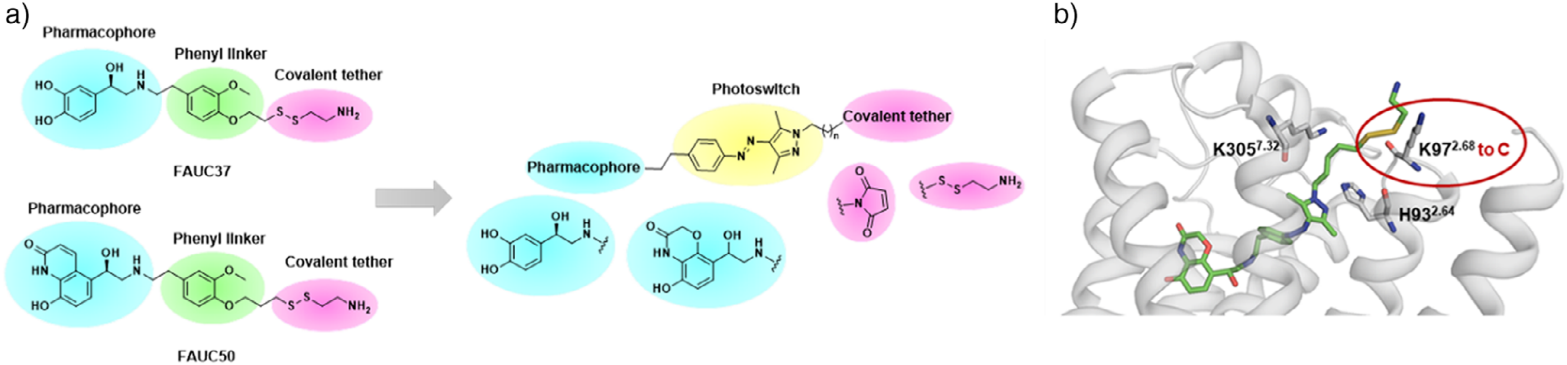
a) The design approach of photoswitchable ligands is based on the covalent agonists FAUC 37 and FAUC 50. b) Docking model of compound **6** bound to the β_2_AR (4LDO).

The design of the covalent photoswitchable ligands was inspired by the previously reported covalent agonists FAUC50 and FAUC37, which enabled the determination of the first structures of irreversible agonist‐bound GPCRs.^[^ ^4^, ^22^ ^]^ These molecules are composed of three main functional units: a pharmacophore, a 1,4‐disubstituted aryl linker, and a covalent tether. In this study, we replaced the aryl linker with a photoswitchable moiety (Figure 2a), using arylazopyrazoles due to their advantages over azobenzenes, such as longer thermal half‐lives and higher photostationary states (PSS).^[^ ^23^ ^]^ The three moieties were connected by short carbon spacers, and the linker between the photoswitch and the covalent tether was optimized to match the distance between the covalent anchoring site and the orthosteric binding site. Two pharmacophores were selected: norepinephrine (NE), the endogenous ligand, to provide insights closely aligned with the natural physiological functions, and BI‐167107, a high‐affinity synthetic agonist featuring a 4‐hydroxybenzoxazinone head group previously used to support the crystallization of the active state of β_2_AR.^[^ ^2^ ^]^ We chose maleimides^[^ ^24^, ^25^ ^]^ and disulfides^[^ ^4^, ^22^, ^26^, ^27^ ^]^ as covalently‐binding units. Although disulfides specifically target cysteines, maleimides can interact with both cysteines and lysines. A total of nine photoswitchable ligands were developed, differing in linker lengths, pharmacophores, and warheads. Recent studies on photoactive covalent dopamine receptor ligands revealed a loss of intrinsic activity upon covalent binding.^[^ ^5^ ^]^ To address this concern, we carefully planned both the ligand design and the receptor engineering. Since cysteines are not always present in orthosteric binding sites, we engineered them in the receptor by point mutation. Suitable anchoring sites on the β_2_AR included H93^2.64^, K97^2.68^, and K305^7.32^, chosen based on their distance and spatial orientation relative to the basic nitrogen of epinephrine's catechol head group or the 4‐hydroxybenzoxazinone equivalent moiety of BI‐167107. The size of the ligand and its ability to undergo effective isomerization were influenced by the choice of the anchoring site and the available space. To probe the different anchoring sites, molecular docking studies were conducted. Docking results indicated that introducing a cysteine at K97^2.68^, between the transmembrane helix (TM) 2 and the extracellular loop (ECL) 1, provided promising binding poses and close proximity between the warhead and the potential anchor point (Figure 2b). In contrast, the distances to K305^7.32^C or H93^2.64^C of the β_2_‐adrenergic receptors were less favorable. Docking of those mutants with disulfide‐ and maleimide‐based ligands did not align with the binding pose of adrenaline, and no significant differences between *E‐* and *Z*‐isomers were observed. Ultimately, the K97^2.68^C mutant emerged as a promising target for anchoring, supported by favorable docking poses.

### Synthesis

The photoswitchable ligands **1–9** were synthesized as outlined in Schemes 1, 2, 3, 4 (see also, Figures S1–S9). The synthesis of the heterocyclic pharmacophore was conducted according to Sweis et al.,^[^ ^28^ ^]^ leading to the production of **16**. The ketone **16** was reduced using sodium borohydride to yield the corresponding alcohol. The azide **17** was simultaneously deprotected and reduced to obtain **18** in excellent yield (Scheme 1).

**Scheme 1:**
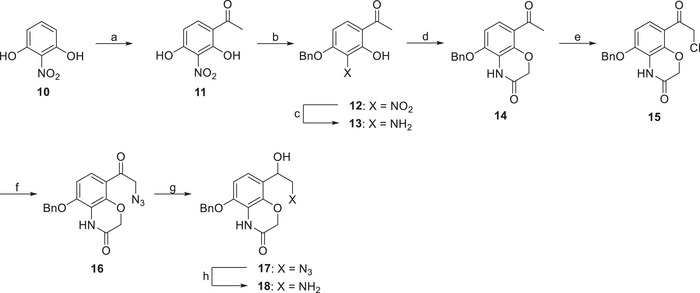
Reagents and conditions: a) Ac_2_O, AlCl_3_, nitrobenzene, 100 °C, 5 h, 97%; b) BnBr, NaHCO_3_, MeCN, reflux, 7 h, 90%; c) Zn dust, AcOH, rt, 2 h, 89%; d) 2‐chloroacetyl chloride, NaHCO_3_, Cs_2_CO_3_, DMF, 100 °C, 20 h, 79%; e) BTMA‐ICl_2_, CH_2_Cl_2_, AcOH, water, 65 °C, 20 h, 90%; f) NaN_3_, DMF, rt, 2 h, 78%; g) NaBH_4_, MeOH, 0 °C, 2 h, 73%; h) Pd/C, H_2_, AcOH, rt, 5 h, 94%.

**Scheme 2:**
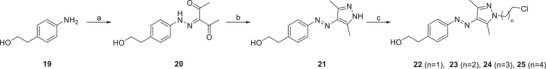
Reagents and conditions: a) NaNO_2_, HCl, AcOH, 0 °C, 45 min, then pentane‐2,4‐dione, NaOAc, EtOH, rt, 30 min, 87%; b) hydrazine monohydrate, EtOH, reflux, 3 h, 93%; c) NaH, anhydrous DMF, rt, 30 min, then 1‐bromo‐2‐chloroethane/1‐bromo‐3‐chloropropane/1‐bromo‐4‐chlorobutane/1‐bromo‐5‐chloropentane, rt, 2 h, 60%–83%.

**Scheme 3:**
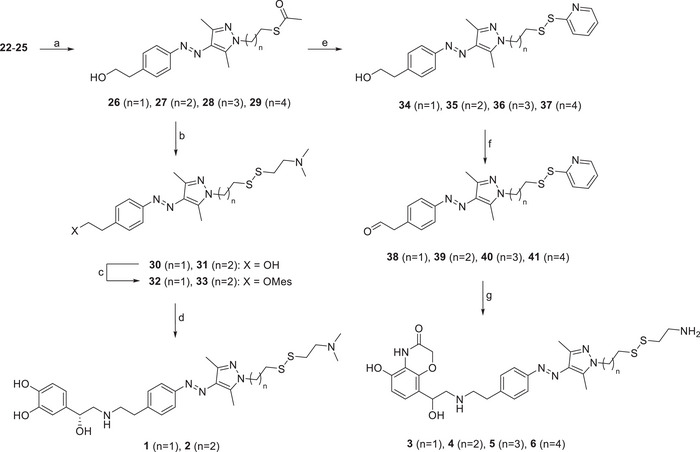
Reagents and conditions: a) potassium thioacetate, THF, reflux, 16 h, 80%–91%; b) 1: 2,2′‐dithiodipyridine, MeOH, NaOCH_3_, rt, 2 h, argon, 2: 2‐(dimethylamino)ethanethiol hydrochloride, rt, 30 min, argon, 59%–60% over two steps; c) MesCl, K_2_CO_3_, CH_2_Cl_2_, 0 °C to rt, 2 h, not isolated; d) norepinephrine, DMSO, argon, 70 °C, 19 h, 8%–19%; e) 2,2′‐dithiodipyridine, LiOH, MeOH, 40 °C, 4 h, 54%–66%; f) DMP, CH_2_Cl_2_, 0 °C, 1.5 h, argon, not isolated; g) **18**, NaCNBH_3_, MeOH, rt, 16 h, then cysteamine hydrochloride, rt, 1 h, 3%–5%.

**Scheme 4:**
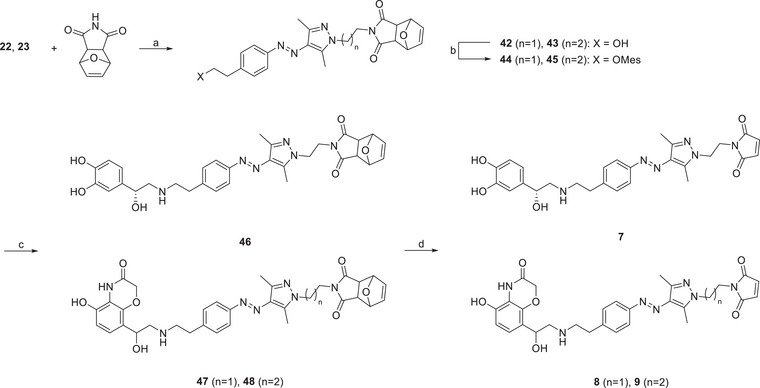
Reagents and conditions: a) K_2_CO_3_, DMF, 50 °C, 16 h, 45%–50%; b) MesCl, NEt_3_, CH_2_Cl_2_, 0 °C to rt, 4 h, not isolated; c) norepinephrine/**18**, DMSO, argon, 70 °C, 16 h, 10%–18%; d) DMSO, argon, 110 °C, 3 h, 85%–98%.

The synthesis of the photoswitchable moiety for the covalent ligands began with compound **19**. After diazotation and azo coupling, **20** was obtained. Subsequent condensation with hydrazine monohydrate produced compound **21**. In the following step, the linkers were introduced. Due to a significant elimination side reaction, bromochloro‐substituted linker equivalents were favored over dibromo analogs. Hence, linkers ranging from two to five carbon atoms were introduced, yielding the building blocks **22–25** (Scheme 2).

From these intermediates, the disulfides and maleimides were synthesized. Nucleophilic replacement gave the thioesters **26–29**. For the norepinephrine‐based ligands, thioesters **26** and **27** were converted in situ to thiopyridyl disulfides, which were subsequently transformed into disulfides **30** and **31**. Subsequent mesitylation and nucleophilic substitution with norepinephrine yielded the covalent ligands **1** and **2**.

For the synthesis of the heterocyclic ligands, the thioesters **26–29** were subjected to methanolysis, isolating the thiopyridyl disulfides **34–37**. Oxidation with Dess–Martin periodane yielded the aldehydes **38–41**, which were subjected to a reductive amination reaction with **18**. After replacing the thiopyridyl group by cysteamine, the covalent ligands **3–6** were obtained (Scheme 3).

The ligands with a maleimide group as the covalent tether were synthesized starting from **22** and **23**, which were reacted with a protected maleimide to produce **42** and **43**. The hydroxyl group was activated by methane sulfonylation and replaced with either norepinephrine or **18,** yielding compounds **46–48**. In the final step, a Retro‐Diels–Alder reaction was employed to deprotect the maleimide, resulting in the formation of ligands **7–9** (Scheme 4).

### Photochemical Characterization

**Figure 3:**
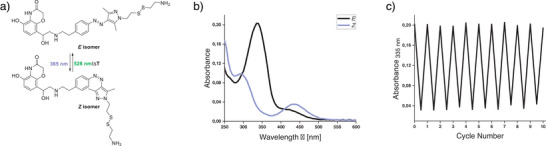
Photophysical characterization of **3** (10 µM + 0.5% DMSO in Tris buffer (50 mM Tris, 1 mM EDTA, 1 mM MgCl_2_, pH 7.4)). a) Light‐induced *E*/*Z*‐photoisomerization. b) UV/Vis spectra of both isomers. c) Cycle performance. Ligands **1**, **2**, **4–9** displayed similar photophysical properties (Figures S10–S37).

The photophysical properties of all photoswitches were investigated in the aqueous buffer to mimic the conditions of biological assays. All arylazopyrazoles could be reversibly toggled between *E‐* and *Z‐*isomers by irradiation with 365 and 528 nm light, respectively (Figure 3). Switching over several cycles showed that the compounds exhibit high fatigue resistance. The UV/Vis spectra showed the typical absorptions of the *E*‐isomer (black curve) and *Z*‐isomer (purple curve). All compounds displayed high PSS in both directions and long thermal half‐lives that are typical for arylazopyrazoles.^[^ ^23^ ^]^ Table 1 summarizes the photophysical properties of all photoswitches.

**Table 1:** Summarized photochemical properties of **1–9** in buffer.^a)^

Compound	λ_max_ (*E*) (nm)	λ_max_ (*Z*) (nm)	λ_iso_ (nm)	t_1/2_ (d)	PSS (*E* → *Z*)^b)^	PSS (*Z* → *E*)^c)^
**1** (‐S‐S‐/n = 2)	335	435	295, 409	3.9	89%	90%
**2** (‐S‐S‐/n = 3)	335	433	295, 409	4.9	89%	87%
**3** (‐S‐S‐/n = 2)	337	434	295, 409	15.2	95%	89%
**4** (‐S‐S‐/n = 3)	339	436	295, 411	15.7	96%	92%
**5** (‐S‐S‐/n = 4)	338	436	298, 409	16.7	94%	92%
**6** (‐S‐S‐/n = 5)	340	435	295, 411	13.3	95%	93%
**7** (‐mal‐/n = 2)	335	433	295, 409	4.5	92%	85%
**8** (‐mal‐/n = 2)	337	435	300, 400	18.6	95%	94%
**9** (‐mal‐/n = 3)	339	435	298, 405	20.4	95%	93%

^a)^ Buffer: Tris buffer: 50 mM Tris, 1 mM EDTA, 1 mM MgCl_2_.

^b)^ PSS determination was done by HPLC at the appropriate isosbestic points, irradiation with 365 nm.

^c)^ PSS determination was done by HPLC at the appropriate isosbestic points, irradiation with 528 nm. mal = maleimide, n = linker length. PSS data [b] and [c] are obtained from a single experiment.

### Biological Characterization

**Figure 4:**
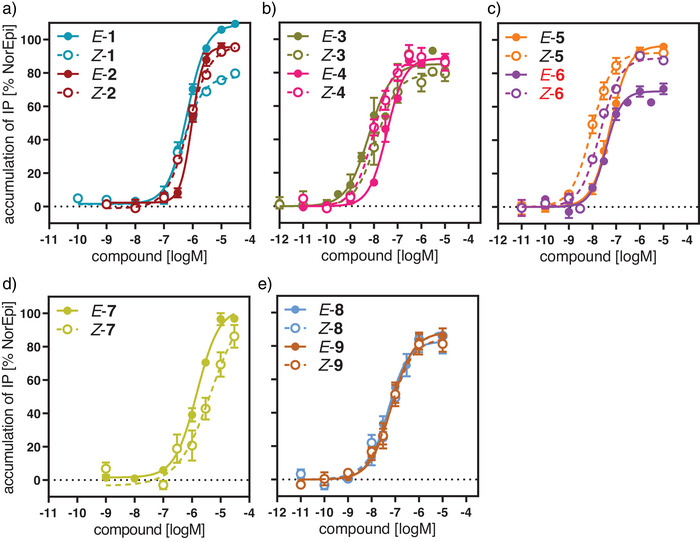
Functional activity of the photoswitches **1–9** at the β_2_ receptor applying an IP‐One assay. a) Dose‐response curves for the *E‐* and *Z*‐isomer of the catechols **1, 2**. b,c) Activation curves for the BI‐167107 derivatives with a disulfide group **3–6**. d,e) Receptor activation curves for the maleimide bearing catechol **7** (d) and the BI‐167107 analogs **8,9** (e). Data represent mean ± SEM from 3–5 independent experiments each conducted in duplicate.

Initial biological investigations focused on the functional characterization of photoswitches **1–9** at the β_2_‐adrenergic receptor (β_2_AR). To evaluate the differential effects of the pharmacophores, photoswitchable entities, and linker units on receptor activation, we performed functional studies. Specifically, we conducted an IP accumulation assay (IP‐One) in HEK293T cells transiently expressing the human β_2_AR and the hybrid G‐protein Gα_qs_.^[^ ^29^ ^]^ Dose‐response curves of the *E‐* and *Z‐*isomers showed agonist activity for all compounds, with E_max_ values between 78% and 108% (Figure 4, Table S1). Ligands containing the BI‐167107 pharmacophore (**3–6, 8, 9**) exhibited potencies in the low nanomolar range, with EC_50_ values between 7.2 nM (*E*‐**3**) and 79 nM (*E*‐**5**). In contrast, catechol derivatives **1**, **2**, and **7** activated the receptor at micromolar concentrations (EC_50_  =  0.48 µM (*Z*‐**1**) to EC_50_  =  4.3 µM (*Z*‐**7**)). Interestingly, the disulfide‐linked derivative **6** exhibited a marked difference in maximum efficacy between the two isomers, with an E_max_  =  70% for *E*‐**6** and E_max_  =  85% for *Z*‐**6**. These data qualified all compounds for further experiments to investigate covalent receptor binding and photo‐induced switching of the bound ligand.

To facilitate disulfide formation or Michael addition of suitable covalent ligands, a β_2_AR mutant was engineered by replacing lysine at position 2.68 on TM2 with a reactive cysteine (K97^2.68^C). Evaluation of the K97^2.68^C mutant revealed activation properties of reference compounds comparable to those observed with wild‐type receptors (norepinephrine: EC_50_  =  3.2 µM for the mutant vs. EC_50_  =  0.98 µM for the wild‐type; epinephrine: EC_50_  =  0.22 µM for the mutant vs. EC_50_  =  0.16 µM for the wild‐type) (Figure S38, Table S2).

**Figure 5:**
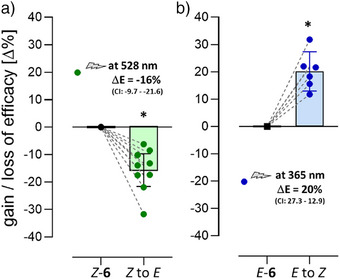
Change of intrinsic activity of the covalently bound test compound **6** by photoswitching within the binding site of the β_2_‐adrenoceptor. Gain and loss of efficacy were determined for each experiment by comparing the efficacy of the covalently bound compound before and after irradiation. a) Loss of efficacy for the *Z*‐isomer of **6** (black dot) after photoswitching the irreversibly bound species into the *E*‐isomer by irradiation at 528 nm. b) Gain of efficacy for the *E*‐isomer (black square) after photoswitching to the *Z*‐isomer by irradiation at 365 nm. Each data point represents the result of a single experiment calculated by paired analysis when the efficacy value of the non‐irradiated isomer was set as 0. Each bar shows the individual data points and mean ΔE values (and 95% CI) of **9** (*Z* to *E*) and **6** (*E* to *Z*) individual experiments each conducted in quadruplicate and applying a biosensor‐based β‐arrestin recruitment assay with the β_2_AR mutant K97^2.68^C. Statistical significance of ΔE was analyzed by a paired t‐test, * *p* values = 0.0003 (*Z* to *E*), 0.0008 (*E* to *Z*).

To assess covalent binding to the receptor and subsequent switching of the bound isomer within the binding site, we developed a bioluminescence resonance energy transfer (BRET)‐based biosensor system expressing an RLuc‐tagged β‐arrestin 2 and GFP‐tagged enhanced bystander protein CAAX in HEK293T cells. The assay allowed us to determine receptor‐stimulated recruitment of β‐arrestin to β_2_AR and the bystander protein CAAX, both located in the cell membrane.^[^ ^30^ ^]^ To facilitate covalent binding, 1 µM of the *Z*‐isomers of **1–9** were pre‐incubated with cells for 90 min. After washing to remove all reversibly‐bound ligands and blocking with propranolol, activation properties were determined following a 15‐min incubation. To identify specific agonist effects of the *Z‐* and *E*‐isomers, cells were treated in two different ways after the blocking step. Specifically, half of the cells were kept in the dark to preserve the *Z*‐isomer, while the second half was irradiated with light at 528 nm to induce switching from the *Z‐* to the *E*‐isomer. Detailed analysis of the BRET data revealed activation profiles with E_max_ values ranging from 2% to 52% relative to the effect of norepinephrine, indicating a partially irreversible occupation of the receptor (Figure S39). Although catechol derivatives **1**, **2**, and **7** exhibited only moderate E_max_ values (2%–19%), the four‐carbon linker‐substituted disulfide **5** (E_max_ = 52%) and its five‐carbon analog **6** (E_max_ = 51%) displayed the highest intrinsic effects. Comparison of the data for all *Z*‐isomers with the intrinsic effects observed after irradiation showed that compound **6** exhibited the most significant difference, consistent with results from the IP accumulation assay reflecting G protein activation (Figure 4). A detailed analysis of the efficacy shift (ΔE) upon photoswitching, performed using paired experimental analysis (Figure 5), revealed that irradiation of the covalently bound *Z*‐**6** at 528 nm caused a significant reduction of receptor activation of 16% (Figure 5a). In contrast, photoswitching of *E*‐**6** (irradiation at 365 nm) to yield the *Z*‐isomer resulted in an efficacy increase of 20% (Figure 5b). These data clearly indicate that compound **6** functions as a covalent photoswitch at the β_2_AR.

### Computational Investigation of Compound Photoswitching and Exit From the Orthosteric Ligand‐binding Pocket

**Figure 6:**
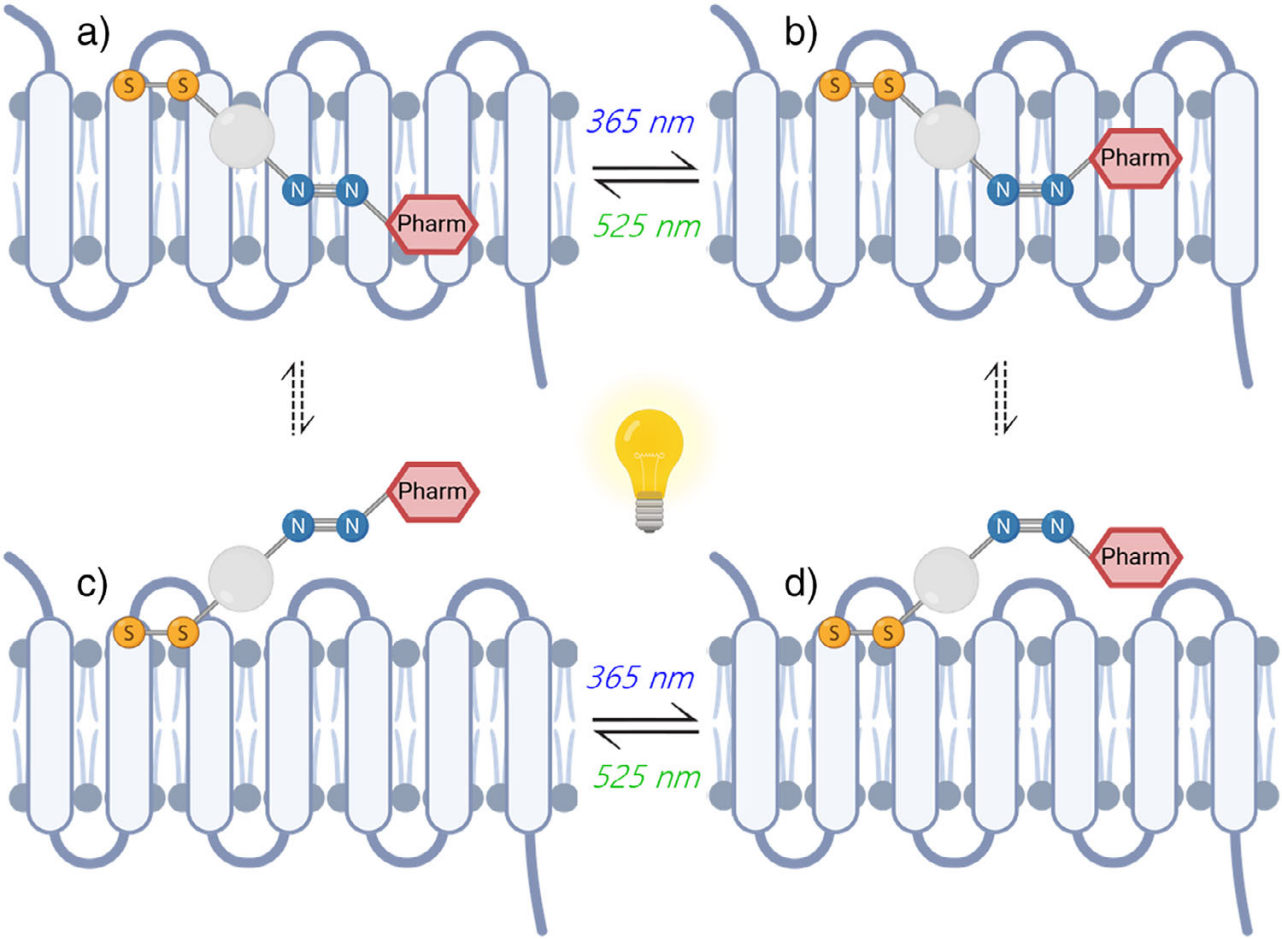
Illustration depicting the different pathways for the compound's photoswitching. In the direct pathway, photoswitching occurs within the orthosteric ligand‐binding pocket (a to b). In the alternative indirect pathway, the ligand first exits the receptor (a to c), undergoes photoswitching outside the receptor (c to d), and then reenters the ligand‐binding site (d to b).

The observed difference in efficacy between the *E‐* and *Z‐*isomers of the covalently‐bound photoactive ligand **6** raised questions about their binding modes and pathways to the β_2_AR and, in particular, whether photoswitching occurs within the ligand‐binding pocket or outside the receptor (Figure 6). To address these questions, we first investigated the stability of the binding mode of *Z*‐**6** within the β_2_AR ligand‐binding pocket, as proposed by molecular docking, using standard molecular dynamics (MD) simulations. After a 50 ns MD production run, we inverted the dihedral restraint on the azo bond of compound *Z*‐**6** to allow isomerization from the *Z‐* to the *E*‐isomer and to study the dynamics of compound *E*‐**6** through further MD simulations. Notably, the *E*‐**6** isomer formed and remained stable during MD simulations, suggesting that the biomolecular environment of the β_2_AR binding pocket allows isomerization (Figure S40).

**Figure 7:**
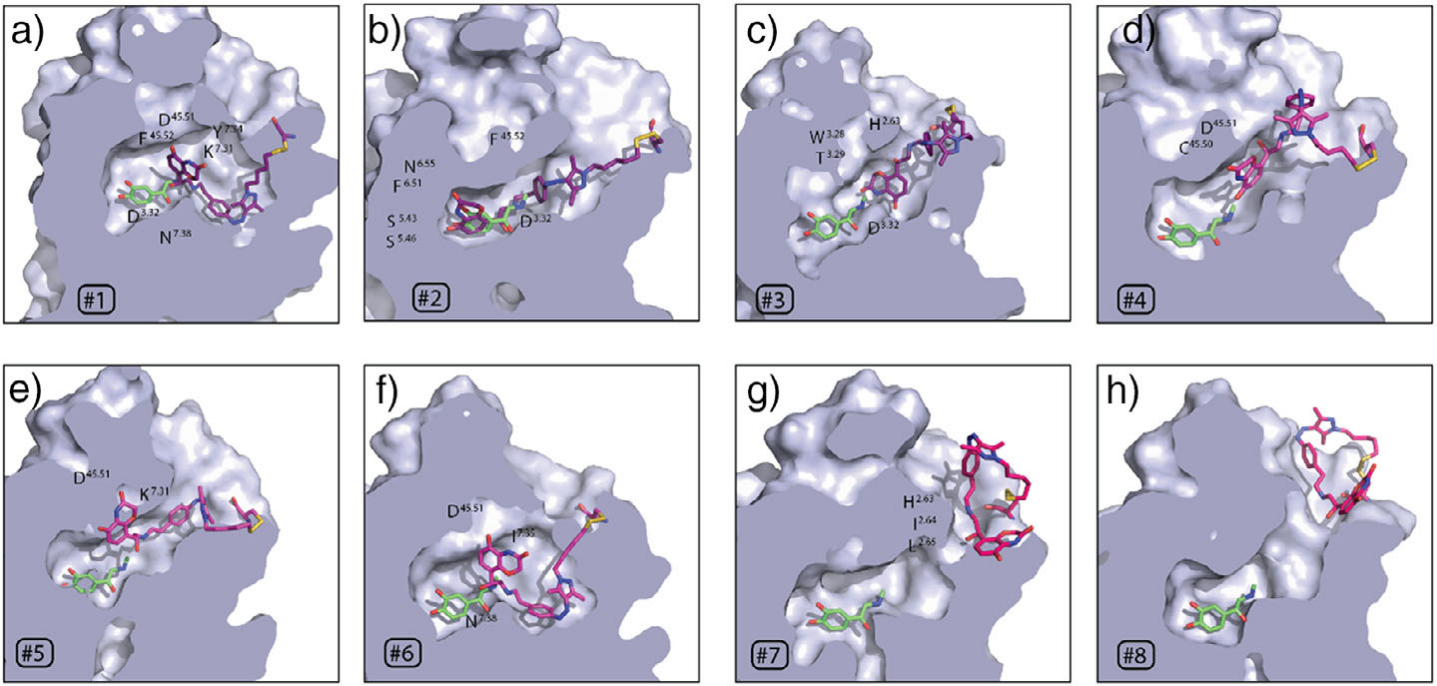
Representative conformations of the eight different clusters #1 to #8 identified along the exit pathway of the covalently‐bound *Z*‐**6** compound, grouped based on the distinct intermolecular interactions with the protein and intramolecular interactions within the ligand. The binding mode of the epinephrine pharmacophore within the experimental structure (PDB: 4LDO)^[^ ^32^ ^]^ is shown in green as a reference. The approximate locations of residues involved in interactions with the pharmacophore (i.e., contact distances within 4 Å) are indicated by residue labels. a) (#1): D^3.32^, D^45.51^, F^45.52^, K^7.31^, Y^7.34^, N^7.38^; b) (#2): D^3.32^, F^45.52^, S^5.43^, S^5.46^, F^6.51^, N^6.55^; C) (#3): H^2.63^, W^3.28^, T^3.29^, D^3.32^; d) (#4): C^45.50^, D^45.51^; e) (#5): D^45.51^, K^7.31^; f) (#6): D^45.51^, I^7.35^, N^7.38^; g) (#7): H^2.63^, I^2.64^, L^2.65^; h) (#8): none. The receptor structure is truncated, with helices TM1, TM6, and TM7 removed for clarity.

After confirming that there are no steric constraints preventing photoswitching within the ligand‐binding pocket, we sought to determine whether photoswitching is confined to the binding pocket. To investigate the exit of the covalently‐bound compound *Z*‐**6** from the β_2_AR ligand‐binding pocket and ultimately the receptor TM bundle, as well as to estimate its conformational stability and residence time along the proposed exit pathway, we conducted multiple walker funnel MetaD simulations^[^ ^31^ ^]^ and analyzed their results with MSMs obtained by applying the MaxCal principle to the MetaD‐derived free energies. Our analysis confirmed the stable binding pose of the covalently bound ligand *Z*‐**6** within the ligand‐binding pocket. It also revealed additional probable states along the compound's exit pathway, characterized by variations in intramolecular interactions within the ligand, ligand‐receptor interactions, relative binding free energies, and residence times. Grouping these ligand conformations based on intermolecular interactions with the protein and intramolecular interactions within the ligand revealed eight clusters. Representative conformations of these clusters (Figure 7) show different locations of the heterocyclic pharmacophore relative to its crystallographic binding pose.^[^ ^32^ ^]^ Specifically, clusters #1–#3 featured conformational states with the heterocyclic head group forming polar interactions with D113^3.32^ (numbers in superscript refer to established generic numbering^[^ ^33^, ^34^ ^]^), either overlapping with or near its crystallographic binding pose within the orthosteric ligand‐binding pocket (Figure 7a–c, Figure S41). Three other clusters (clusters #4–#6) displayed a loss of key pharmacophore interactions with D113^3.32^ and adopted an intermediate position between the crystallographic binding pose and locations outside the β_2_AR TM bundle (Figure 7d–f, Figure S41). The remaining clusters (clusters #7 and #8) presented the pharmacophore outside the TM bundle without interactions with D113^3.32^ (Figure 7g,h, Figure S41).

Among the different binding poses of the covalently‐bound compound *Z*‐**6** at the ligand‐binding pocket, the pharmacophore of the most populated cluster (#2, representing 68% of the reweighed MetaD simulations; Table S3) overlapped with the respective head group of epinephrine in the crystallographic pose (PDB: 4LDO)^[^ ^32^ ^]^ and engaged identical ligand‐receptor interactions with S203^5.43^, S207^5.46^, N293^6.55^, and the conserved salt‐bridge with D113^3.32^ (Figure 7b, Figure S41). Notably, this was the only pose in which *Z*‐**6** interacted with S203^5.43^ and S207^5.46^. In all highly probable binding poses within the TM bundle, the ligand interacted with ECL2, involving, among other residues, the conserved F193^45.52^, which has been implicated in biased agonism.^[^ ^35^ ^]^ A secondary pose within the β_2_AR orthosteric ligand‐binding pocket (cluster #1, accounting for 32% of the reweighted MetaD simulations; Table S3 and Figure 7a) positioned the pharmacophore away from the key serines in TM5 toward crucial residues such as K305^7.31^, Y255^7.34^, or N259^7.38^ in TM7. The binding states of *Z*‐**6** with the pharmacophore in intermediate positions between its crystallographic binding pose and locations outside the β_2_AR TM bundle (clusters #4, #5, and #6), showed a loss of the experimentally confirmed interactions, except for those with conserved residues in ECL2 (D192^45.51^), which were replaced by new interactions (Figure 7d–f, Figure S41), such as those with K305^7.31^ and I309^7.35^. These interactions correspond to an allosteric pocket previously described for the β_2_AR bound to the partial agonist salmeterol^[^ ^36^ ^]^ and for the M2 muscarinic receptor.^[^ ^37^ ^]^ The two conformational states in cluster #7 indicated interactions between the pharmacophore and the top of TM2 (residues H93^2.63^, I94^2.64^, and L95^2.65^) (Figure 7g, Figure S41), whereas in cluster #8, the pharmacophore was completely disengaged from the protein (Figure 7h).

Free‐energy reconstruction revealed that the aggregated free‐energy difference between states where the pharmacophore of the covalently‐bound compound *Z*‐**6** resides within the orthosteric ligand‐binding pocket (clusters #1–#3) and those in intermediate or external positions was 8.6 ± 0.3 and 12.57 ± 0.01 kT, respectively, confirming that the orthosteric ligand‐binding pocket is the most probable binding site for this compound (Table S3). Notably, simulations of non‐covalently bound Z‐**6** showed similar preferences, with the most probable binding state within the orthosteric ligand‐binding pocket separated from alternative or outside locations by 11.23 ± 0.09 and 12 ± 0.3 kT, respectively (Table S3).

Although the thermodynamic data confirm that the *Z*‐**6** ligand primarily binds at the β_2_AR orthosteric ligand‐binding pocket, kinetic insights are necessary to determine the likelihood of photoswitching occurring inside or outside this pocket. To address this, we derived unbiased kinetic information from the MetaD simulations by applying the MaxCal approach to the MetaD‐derived free energies to obtain MSMs and calculating mean‐first passage times (MFPTs) and residence times for the Z‐**6** ligand by applying transition path theory^[^ ^38^ ^]^ to the MaxCal transition matrix. The MFPTs from bound states within the β_2_AR orthosteric ligand‐binding pocket to unbound states were estimated to be 2.5 ± 0.3 ms, whereas the MFPT from alternative states not interacting with D113^3.32^ to the bound state was 1.5 ± 0.1 ms (Table S4). Moreover, the residence time in the orthosteric ligand binding pocket was about three times longer than the residence time at alternative sites (Table S4). The corresponding on‐ and off timescales for the non‐covalently bound *Z*‐**6** were 510 ± 30 and 340 ± 10 µs, respectively, indicating a longer residence time for *Z*‐**6** within the β_2_AR orthosteric ligand‐binding pocket when covalently bound to the receptor.

Taken together, these computational findings, supported by experimental data, suggest that photoswitching predominantly occurs within the β_2_AR ligand‐binding pocket. Compared to our earlier study,^[^ ^18^ ^]^ which demonstrated photoswitching within the orthosteric binding pocket by reducing rebinding events, the current study provides a more detailed mechanistic and kinetic analysis of the process.

## Conclusion

The β_2_AR is a valuable drug target and a paradigmatic GPCR that is frequently used for mechanistic studies on receptor activation. Here, we describe the first covalent β_2_AR agonist (**6**) that is photoswitchable in its receptor‐bound state. Recent studies on photoactive covalent dopamine receptor ligands indicated a loss of intrinsic activity upon covalent binding.^[^ ^5^ ^]^ Therefore, we carefully designed both the ligand and the receptor engineering.

The covalent ligand was synthesized by incorporating an arylazopyrazole moiety between its pharmacophore and a disulfide‐functionalized tether. Compound **6** complements retinal in the rhodopsin system, which gets hydrolyzed and leaves the receptor after isomerization to the *trans*‐isomer. For subsequent photoswitching cycles, opsin requires binding another equivalent of *cis*‐retinal.^[^ ^39^, ^40^ ^]^ The system described here is based on a β_2_AR mutant with a cysteine anchor located adjacent to the orthosteric binding pocket (K97^2.68^C). Due to the close proximity of the pharmacophore to the cysteine anchor and the shape of the deep binding pocket, dissociation and re‐association of the pharmacophore before or after photoswitching are unlikely for steric reasons. Computational investigations of **6**, supported by experimental data, provided strong evidence that photoswitching occurs within the binding pocket. The photophysical characterization of our set of test compounds showed excellent properties, including reversible switching, high fatigue resistance, a high PSS, and long thermal half‐lives. As the wild‐type receptor lacks a free cysteine required for covalent binding, the ligands were tested on an engineered receptor (K97^2.68^C). Compound **6** will serve as a valuable research tool, allowing us to study receptor kinetics and dynamics with high accuracy through high‐resolution biophysical studies. More than 30% of the approved drugs and drug candidates exert their activity by modulating GPCRs. Beyond the challenging applications for the β_2_AR, photoswitchable covalent ligands will be extremely helpful tools to deepen our molecular understanding of GPCR function and signaling. For example, photoswitchable allosteric modulators or biased ligands will be of interest to study the modulation of the signaling by endogenous ligands in more complex in vitro assay systems including organoids.

## Conflict of Interests

The authors declare no conflict of interest.

## Supporting information



Supporting Information

## Data Availability

The data that support the findings of this study are available in the supplementary material of this article.
