# Supplementary material for: Synthesis and Characterization of Photoswitchable Covalent Ligands for the β2‐Adrenoceptor
Source: Angew Chem Int Ed Engl. 2025 Apr 10;64(24):e202424038. doi: 10.1002/anie.202424038 (PMC12144893; doi:10.1002/anie.202424038)
Supplement: Supplementary file 1 — Supporting Information [file ANIE-64-e202424038-s001.pdf]

Synthesis and Characterization of Photoswitchable Covalent Ligands for the β_2-Adrenoceptor

Table of Content

1. Chemistry 3
  1.1. General Methods 3
  1.2. Synthetic Procedures 5
  1.3. Purity 25
  1.4. Photophysical Characterization 27
    1.4.1. UV/Vis Spectra and Cycle Performance 27
    1.4.2. Photostationary States 30
    1.4.3. Thermal Half-lives 34
2. Biological Investigations 37
  2.1. Biological Characterization 38
    2.1.1. IP-One Assay on Functional Activity of the Photoswitches 1-9 at the β_2-adrenoceptor. 38
    2.1.2. Determination of Receptor Activation after Covalent Binding and Photoswitching Applying an Arrestin Recruitment Assay 40
3. Computational Methods 42
  3.1. Molecular docking 42
  3.2. Molecular dynamics simulations 42
  3.3. Metadynamics simulations 44
  3.4. Clustering and Markov state modeling analysis 45
4. Abbreviations 49
5. NMR-Spectra 52
6. References 84

1. Chemistry

1.1. General Methods

Reagents and working techniques

Starting materials and commercial reagents were purchased from Acros, Alfa Aesar, Fisher, Fluka, Fluorochem, Merck, Sigma-Aldrich, TCI and VWR and were used without further purification. Solvents were used in p.a. quality or dried according to common procedures if necessary.

All reactions with oxygen- or moisture-sensitive reagents were carried out in glassware which was dried before use by heating under vacuum. Dry nitrogen or argon were used as inert gas atmosphere.

Analytical techniques

Nuclear magnetic resonance spectroscopy (NMR)

All NMR spectra were measured at room temperature using a Bruker Avance 300 (300 MHz for ^{1}H and 75 MHz for ^{13}C) or a Bruker Avance 400 (400 MHz for ^{1}H and 101 MHz for ^{13}C) or a Bruker Avance 600 (600 MHz for ^{1}H and 151 MHz for ^{13}C) NMR spectrometer. All chemical shifts are reported in δ-scale as parts per million [ppm] (multiplicity, coupling constant J, number of protons) relative to the solvent residual peaks or relative to tetramethylsilane as the internal standard. Coupling constants J are given in Hertz [Hz]. Abbreviations used for signal multiplicity: ^{1}H–NMR: s = singlet, d = doublet, dd = doublet of doublets, ddd = doublet of doublets of doublets, dt = doublet of triplets, t = triplet, td= triplet of doublets, q = quartet, and m = multiplet.

Mass spectrometry (MS)

All mass spectra were recorded on an Bruker timsTOF, Agilent Q-TOF 6540 UHD, Finnigan MAT SSQ 710 A, Jeol AccuTOF GCX or ThermoQuest Finnigan TSQ 7000 spectrometer.

HPLC-MS analyses

HPLC-MS analyses were performed on a Thermo Scientific Dionex Ultimate 3000 HPLC system combined with a DAD detector (230 nm; 254 nm) and either using a Kinetex 2.6 u mesh C8 100A (2.1 x 75 mm, 2.6 µm) or an Agilent ZORBAX Eclipse XDB-C8 (3.5 x 100 mm, 3.5 µm). For mass detection, a BRUKER amazon SL mass spectrometer using ESI ionization was incorporated. The solvent system was Methanol/H20 + 0.1% formic acid (25% for 0.2 min, gradient 25 - 100% in 5.8 min, 100% for 2.5 min, gradient 100 - 25% in 0.5 min, 25% for 3 min) at a flow rate of 0.3 mL/min for Kinetex column and 0.4 mL/min for Agilent

UV-Vis absorption spectroscopy

Absorption spectra were recorded on a UV/VIS Agilent Cary 100 spectrometer or a Specord 200 Plus double-beam photometer.

UV/Vis Plate Reader

Thermal half-lives were measured on a 96-well plate in a Thermo Scientific Multiskan® Spectrum.

Thin layer chromatography (TLC)

Analytical thin layer chromatography (TLC) was performed on silica gel coated alumina plates (MN precoated TLC-sheets ALUGRAM® Xtra SIL G/UV254). Visualization was done by UV-light (254 nm or 366 nm) or staining with ninhydrin or $KMnO_4$ solution.

Column chromatography

Column chromatography was performed using normal-grade silica gel (SiO2, 60 Ǻ) under gravity flow conditions in a standard glass column setup with isocratic elution (composition of the mobile phase is given for each experiment) or on a Biotage Isolera One automated flash purification system with UV/Vis detector.

Analytical RP-HPLC

Analytical RP-HPLC were measured on an Agilent 1200 Series HPLC system (column: Agilent ZORBAX Eclipse XCB-C8 (4.6 x 150 mm, 5 µm) and an Agilent 1220 Infinity LC System (column: P/No 00F-4251-B0, Phenomenex Luna® 3 µm C18(2) 100 Å, LC column 150x2.0 mm).

Preparative RP-HPLC

Purification by preparative HPLC was conducted on a preparative HPLC Agilent Series 1100 system (column: Agilent ZORBAX XDB-C8 (21.2 x 150 mm, 5µm)) and an Agilent 1260 Infinity LC System (column: P/No 00G-4253-P0-AX, Phenomenex Luna® 10 µm C18(2) 100 Å, LC column 250x21.2 mm). The eluent systems were used as specified. After the purification process, solvents were removed by lyophilization.

Light sources (LEDs)

Switching experiments were done with a 365 nm LED (SSC VIOSYS CUN66A1B, 700 mA, 1250 mW) and 528 nm LED (OSRAM Oslon SSL 80 green, 500 mA, 34 mW).

1.2. Synthetic Procedures

8-(2-Azido-1-hydroxyethyl)-5-(benzyloxy)-2*H*-benzo[*b*][1,4]oxazin-3(4*H*)-one (**17**)

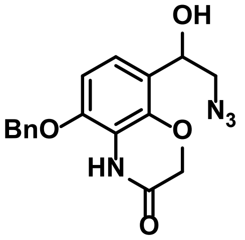

Chemical Formula: $C_{17}H_{16}N_4O_4$
340,34

To a stirred solution of **16** (1.0 g, 2.9 mmol, 1.0 eq.) in anhydrous methanol at 0 °C, $NaBH_4$ (111 mg, 2.9 mmol, 1.0 eq.) was added. The mixture was stirred for 2 h at 0 °C and was then quenched with H_2O. After extraction with ethyl acetate the combined phases were dried over $MgSO_4$, filtered and the solvent was evaporated. The crude product was used for automated column chromatography (PE/EtOAc, 0-80%). The pure product was obtained as a beige solid (720 mg, 2.1 mmol, 73%).

^{1}H NMR (400 MHz, DMSO-d6) δ 10.12 (s, 1H), 7.56 – 7.51 (m, 2H), 7.36 (t, J = 7.3 Hz, 2H), 7.33 – 7.26 (m, 1H), 7.04 (d, J = 8.6 Hz, 1H), 6.77 (d, J = 8.7 Hz, 1H), 5.17 (s, 2H), 4.98 (dd, J = 6.6, 4.5 Hz, 1H), 4.53 (d, J = 3.7 Hz, 2H), 3.27 – 3.21 (m, 2H).

^{13}C NMR (101 MHz, DMSO) δ 164.4, 145.2, 140.8, 136.8, 128.2, 127.7, 127.6, 122.8, 120.1, 116.4, 106.6, 69.9, 67.0, 66.5, 56.1.
ESI-MS: m/z (%) = 341.12 $(M+H)^+$.

8-(2-Amino-1-hydroxyethyl)-5-hydroxy-2*H*-benzo[*b*][1,4]oxazin-3(4*H*)-one (**18**)

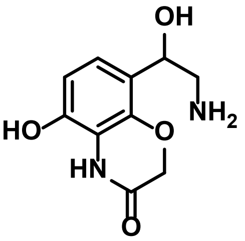

Chemical Formula: $C_{10}H_{12}N_2O_4$
224,22

Compound **17** (1.0 g, 2.9 mmol, 1.0 equiv.) was dissolved in acetic acid (40 mL). Pd/C (10 mol%, 309 mg) was added. The mixture was stirred for 5 h at rt in a hydrogen atmosphere (10 bar). The mixture was filtered over celite and the solvent was removed *in vacuo.* The residue was dissolved in MeOH and a spoon of Amerlite IRA-67 (ion-exchange resin) was added. The mixture was stirred for 2 h and

afterwards filtered. The solvent was removed and the product was afforded as an off-white solid (620 mg, 2.8 mmol, 94%). The product was used without further purification.

^{1}H NMR (400 MHz, Methanol-d4) δ 7.02 (d, J = 8.5 Hz, 1H), 6.57 (d, J = 8.5 Hz, 1H), 5.10 (dd, J = 9.1, 3.4 Hz, 1H), 4.58 (d, J = 3.5 Hz, 2H), 3.15 (dd, J = 12.6, 3.4 Hz, 1H), 2.98 (dd, J = 12.6, 9.1 Hz, 1H).
^{13}C NMR (101 MHz, MeOD) δ 166.7, 146.6, 142.7, 121.8, 120.8, 116.3, 110.1, 68.1, 65.5, 46.1.
ESI-MS: m/z (%) = 225.09 $(M+H)^{+}$.

3-(2-(4-(2-Hydroxyethyl)phenyl)hydrazineylidene)pentane-2,4-dione (**20**)

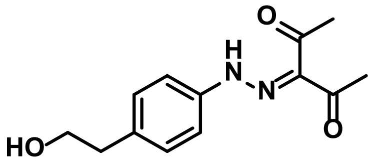

Chemical Formula: $C_{13}H_{16}N_2O_3$
248,28

2-(4-Aminophenyl)ethanol (10.0 g, 73.0 mmol, 1.0 eq.) was dissolved in AcOH (109 mL) and conc. HCl (18 mL) and the mixture was cooled to 0 °C. $NaNO_2$ (6.0 g, 87.0 mmol, 1.2 eq.) was dissolved in a minimum amount of water and added to the cooled reaction mixture. The mixture was stirred for 45 min and subsequently transferred to a mixture of acetylacetone (9.7 mL, 95 mmol, 1.3 eq.) and NaOAc (17.9 g, 220 mmol, 3.0 eq.) in EtOH (73 mL). The mixture turned yellow and was stirred for further 30 min. Icewater was added and the obtained precipitate was filtered off, washed with EtOH/H_2O (1:1) and dried under vacuum to obtain a yellow solid (15.8 g, 63.7 mmol, 87%).

^{1}H NMR (400 MHz, Chloroform-d) δ 14.67 (s, 1H), 7.30 – 7.24 (m, 2H), 7.20 – 7.17 (m, 2H), 3.78 (t, J = 6.5 Hz, 2H), 2.79 (t, J = 6.5 Hz, 2H), 2.50 (s, 3H), 2.39 (s, 3H).

^{13}C NMR (101 MHz, $CDCl_3$) δ 198.0, 197.3, 140.3, 136.7, 133.2, 130.4, 116.6, 63.7, 38.8, 31.8, 26.8.

ESI-MS: m/z (%) = 249.12 $(M+H)^{+}$.

(*E*)-2-(4-((3,5-Dimethyl-1*H*-pyrazol-4-yl)diazenyl)phenyl)ethan-1-ol (**21**)

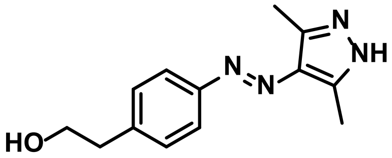

Chemical Formula: $C_{13}H_{16}N_4O$
244,30

Compound **20** (10.0 g, 40.3 mmol, 1.0 eq.) was dissolved in EtOH (200 mL). Then, hydrazine monohydrate solution (64% hydrazine monohydrate in water, 3.15 g, 3.05 mL, 40.3 mmol, 1.0 eq.) was added and the reaction mixture was refluxed for 3 h. The solvent was almost completely removed and the remaining volume was poured onto icewater and the formed precipitate was filtered off, washed with EtOH/H_2O (1:1) and dried under vacuum to obtain a yellow solid (9.2 g, 37.7 mmol, 93%).

^{1}H NMR (400 MHz, DMSO-d6) δ 7.65 – 7.60 (m, 2H), 7.37 – 7.30 (m, 2H), 3.64 (t, J = 7.0 Hz, 2H), 2.78 (t, J = 6.9 Hz, 2H), 2.44 (s, 6H).

^{13}C NMR (101 MHz, DMSO) δ 151.5, 141.4, 140.3, 134.1, 129.6, 121.2, 109.6, 62.0, 38.8, 11.9.

ESI-MS: m/z (%) = 245.14 $(M+H)^+$.

General procedure for compounds **22**-**25**

NaH (60% dispersion in mineral oil, 1.5 eq.) was added in portions at to a stirred solution of **21** (1.0 eq.) in anhydrous DMF under nitrogen. The solution was stirred for 30 min. Then the solution was added dropwise to a solution of 1-bromo-2-chloroethane/1-bromo-3-chloropropane/1-bromo-4-chlorobutane/1-bromo-5-chloropentane (3.0 eq.) in anhydrous DMF. The mixture was stirred for 2 h at rt. Then, water was added and the mixture was extracted with ethyl acetate. The organic phase was washed with water and brine. The organic phase was dried over $MgSO_4$ and the solvent was removed *in vacuo*. The crude product was purified by column chromatography (PE/EtOAc, 20-100%).

(*E*)-2-(4-((1-(2-Chloroethyl)-3,5-dimethyl-1*H*-pyrazol-4-yl)diazenyl)phenyl)ethan-1-ol (**22**)

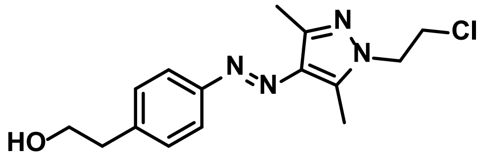

Chemical Formula: $C_{15}H_{19}ClN_4O$
306,79

Yield: 60%

^{1}H NMR (400 MHz, Methanol-d4) δ 7.73 – 7.66 (m, 2H), 7.38 – 7.30 (m, 2H), 4.36 (t, J = 5.8 Hz, 2H), 3.94 (t, J = 5.8 Hz, 2H), 3.79 (t, J = 7.0 Hz, 2H), 2.87 (t, J = 7.0 Hz, 2H), 2.61 (s, 3H), 2.45 (s, 3H).

^{13}C NMR (101 MHz, MeOD) δ 153.4, 143.8, 142.5, 142.0, 135.7, 130.6, 122.8, 64.0, 50.9, 43.8, 40.0, 14.2, 9.9.

ESI-MS: m/z (%) = 307.13 $(M+H)^+$.

(*E*)-2-(4-((1-(3-Chloropropyl)-3,5-dimethyl-1*H*-pyrazol-4-yl)diazenyl)phenyl)ethan-1-ol (**23**)

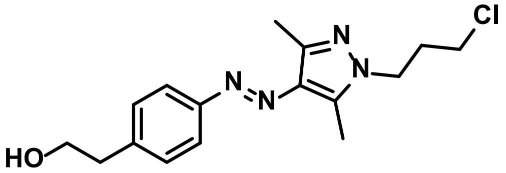

Chemical Formula: $C_{16}H_{21}ClN_4O$
320,82

Yield: 83%

^{1}H NMR (400 MHz, Methanol-d4) δ 7.72 – 7.67 (m, 2H), 7.36 – 7.31 (m, 2H), 4.23 (t, J = 6.8 Hz, 2H), 3.80 (t, J = 7.0 Hz, 2H), 3.58 (t, J = 6.2 Hz, 2H), 2.88 (t, J = 7.0 Hz, 2H), 2.62 (s, 3H), 2.45 (s, 3H), 2.34 – 2.25 (m, 2H).

^{13}C NMR (101 MHz, MeOD) δ 153.5, 143.5, 142.5, 141.0, 135.8, 130.7, 122.8, 64.0, 46.6, 42.4, 40.0, 33.6, 14.0, 9.7.

ESI-MS: m/z (%) = 321.15 (M+H)$^+$.

(*E*)-2-(4-((1-(4-Chlorobutyl)-3,5-dimethyl-1*H*-pyrazol-4-yl)diazenyl)phenyl)ethan-1-ol (**24**)

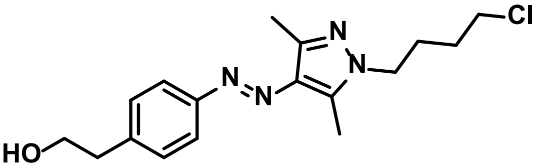

Chemical Formula: $C_{17}H_{23}ClN_4O$
334,85

Yield: 80%

^{1}H NMR (400 MHz, Methanol-d4) δ 7.72 – 7.66 (m, 2H), 7.36 – 7.30 (m, 2H), 4.08 (t, J = 7.1 Hz, 2H), 3.79 (t, J = 7.0 Hz, 2H), 3.58 (t, J = 6.5 Hz, 2H), 2.87 (t, J = 7.0 Hz, 2H), 2.58 (s, 3H), 2.44 (s, 3H), 2.00 – 1.90 (m, 2H), 1.82 – 1.72 (m, 2H).

^{13}C NMR (101 MHz, MeOD) δ 153.4, 143.2, 142.4, 140.4, 135.8, 130.6, 122.8, 64.0, 49.0, 45.1, 40.0, 30.7, 28.3, 14.0, 9.8.

ESI-MS: m/z (%) = 335.16 (M+H)$^+$.

(*E*)-2-(4-((1-(5-Chloropentyl)-3,5-dimethyl-1*H*-pyrazol-4-yl)diazenyl)phenyl)ethan-1-ol (**25**)

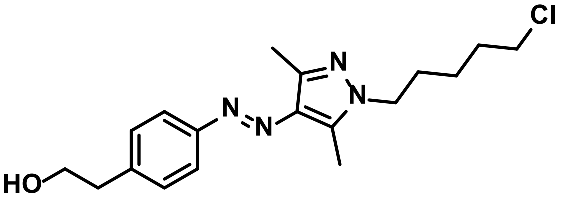

Chemical Formula: $C_{18}H_{25}ClN_4O$
348,88

Yield: 76%

^{1}**H NMR** (400 MHz, Methanol-d4) δ 7.71 – 7.66 (m, 2H), 7.35 – 7.30 (m, 2H), 4.05 (t, J = 7.2 Hz, 2H), 3.79 (t, J = 7.0 Hz, 2H), 3.55 (t, J = 6.6 Hz, 2H), 2.87 (t, J = 7.0 Hz, 2H), 2.57 (s, 3H), 2.44 (s, 3H), 1.89 – 1.73 (m, 4H), 1.51 – 1.42 (m, 2H).

^{13}**C NMR** (101 MHz, MeOD) δ 153.4, 143.1, 142.4, 140.4, 135.8, 130.6, 122.8, 64.0, 49.6, 45.4, 40.0, 33.3, 30.2, 25.0, 14.0, 9.8.

ESI-MS: m/z (%) = 349.18 $(M+H)^+$.

General procedure for compounds **26**-**29**

Compounds **22-25** (1.0 eq.) and potassium thioacetate (1.9 eq.) were refluxed in THF overnight. Solvent was removed under reduced pressure. The crude product was purified by column chromatography (PE/EtOAc, 30-100%).

(*E*)-S-(2-(4-((4-(2-Hydroxyethyl)phenyl)diazenyl)-3,5-dimethyl-1*H*-pyrazol-1-yl)ethyl) ethanethioate (**26**)

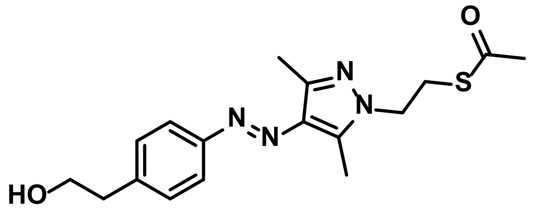

Chemical Formula: $C_{17}H_{22}N_4O_2S$
346,45

Yield: 80%

^{1}**H NMR** (400 MHz, Chloroform-d) δ 7.71 (d, J = 8.0 Hz, 2H), 7.30 (d, J = 8.3 Hz, 2H), 4.20 – 4.13 (m, 2H), 3.88 (d, J = 6.4 Hz, 2H), 3.30 – 3.24 (m, 2H), 2.90 (t, J = 6.6 Hz, 2H), 2.59 (s, 3H), 2.47 (s, 3H), 2.35 (s, 3H).

^{13}**C NMR** (101 MHz, $CDCl_3$) δ 195.6, 152.4, 143.0, 140.3, 139.2, 135.0, 129.6, 122.0, 63.5, 47.8, 39.1, 30.7, 29.1, 14.1, 9.9.

ESI-MS: m/z (%) = 347.15 $(M+H)^+$.

(*E*)-S-(3-(4-((4-(2-Hydroxyethyl)phenyl)diazenyl)-3,5-dimethyl-1*H*-pyrazol-1-yl)propyl) ethanethioate (**27**)

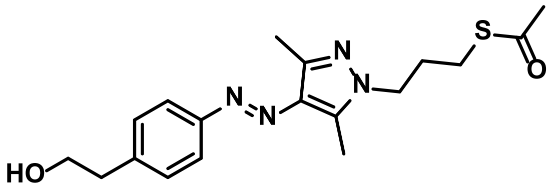

Chemical Formula: $C_{18}H_{24}N_4O_2S$
360,48

Yield: 91%

^{1}H NMR (300 MHz, Methanol-d4) δ 7.70 (d, J = 8.4 Hz, 2H), 7.34 (d, J = 8.4 Hz, 2H), 4.12 (t, J = 7.0 Hz, 2H), 3.79 (t, J = 7.0 Hz, 2H), 2.88 (td, J = 7.0, 1.5 Hz, 4H), 2.59 (s, 3H), 2.45 (s, 3H), 2.32 (s, 3H), 2.12 – 2.03 (m, 2H).

^{13}C NMR (75 MHz, MeOD) δ 197.0, 153.4, 143.3, 142.4, 140.7, 135.9, 130.7, 122.8, 64.0, 40.0, 30.9, 30.7, 30.5, 26.8, 14.0, 9.8.

ESI-MS: m/z (%) = 361.17 $(M+H)^+$.

(*E*)-S-(4-(4-((4-(2-Hydroxyethyl)phenyl)diazenyl)-3,5-dimethyl-1*H*-pyrazol-1-yl)butyl) ethanethioate (**28**)

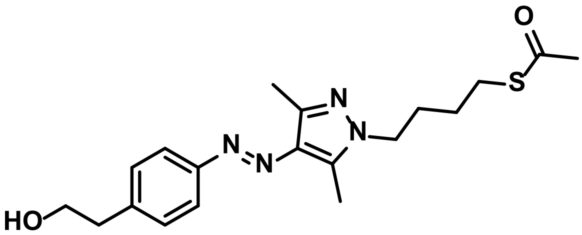

Chemical Formula: $C_{19}H_{26}N_4O_2S$
374,50

Yield: 82%

^{1}H NMR (400 MHz, Chloroform-d) δ 7.72 (d, J = 8.3 Hz, 2H), 7.31 (d, J = 8.1 Hz, 2H), 4.03 (t, J = 7.2 Hz, 2H), 3.89 (t, J = 6.6 Hz, 2H), 2.91 (dt, J = 10.6, 6.9 Hz, 4H), 2.57 (s, 3H), 2.48 (s, 3H), 2.32 (s, 3H), 1.91 (p, J = 7.4 Hz, 2H), 1.63 (p, J = 7.3 Hz, 3H).

^{13}C NMR (101 MHz, $CDCl_3$) δ 195.9, 152.6, 142.6, 140.1, 138.4, 135.2, 129.7, 122.1, 63.7, 48.5, 39.2, 30.8, 29.1, 28.6, 26.9, 14.1, 10.0.

ESI-MS: m/z (%) = 375.19 $(M+H)^+$.

(*E*)-S-(5-(4-((4-(2-Hydroxyethyl)phenyl)diazenyl)-3,5-dimethyl-1*H*-pyrazol-1-yl)pentyl) ethanethioate (**29**)

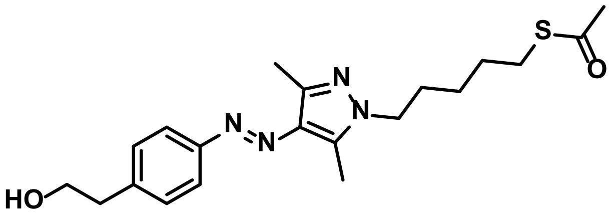

Chemical Formula: $C_{20}H_{28}N_4O_2S$
388,53

Yield: 87%

^{1}H NMR (400 MHz, Chloroform-d) δ 7.69 (d, J = 8.3 Hz, 2H), 7.28 (d, J = 8.3 Hz, 2H), 3.97 (t, J = 7.2 Hz, 2H), 3.85 (t, J = 6.6 Hz, 2H), 2.89 (t, J = 6.6 Hz, 2H), 2.83 (t, J = 7.3 Hz, 2H), 2.54 (s, 3H), 2.47 (s, 3H), 2.29 (s, 3H), 1.82 (p, J = 7.4 Hz, 2H), 1.64 – 1.53 (m, 2H), 1.38 (tt, J = 10.2, 6.2 Hz, 2H).

^{13}C NMR (101 MHz, $CDCl_3$) δ 195.9, 152.4, 142.4, 140.2, 138.3, 135.0, 129.6, 121.9, 63.5, 48.7, 39.1, 30.7, 29.5, 29.2, 28.8, 25.8, 14.0, 9.9.

ESI-MS: m/z (%) = 389.20 $(M+H)^+$.

General procedure for compounds **34-37**

Compounds **26-29** (1.0 eq.) and 2,2'-dithiopyridine (1.2 eq.) were dissolved in methanol. Dropwise over 1 h aqueous LiOH solution (1 M, 1.2 eq.) was added. After 3 h at 40 °C, the reaction mixture was concentrated and purified by column chromatography (PE/EtOAc, 20-100%).

(*E*)-2-(4-((3,5-Dimethyl-1-(2-(pyridin-2-yldisulfaneyl)ethyl)-1*H*-pyrazol-4-yl)diazenyl)phenyl)ethan-1-ol (**34**)

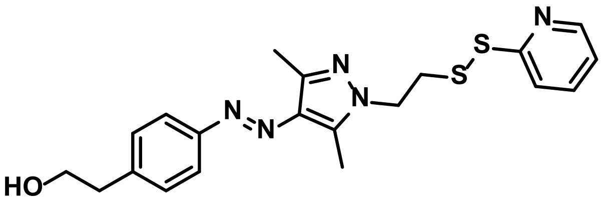

Chemical Formula: $C_{20}H_{23}N_5OS_2$
413,56

Yield: 54%

^{1}H NMR (400 MHz, Chloroform-d) δ 8.52 – 8.45 (m, 1H), 7.72 (d, J = 7.9 Hz, 2H), 7.64 (dq, J = 4.1, 1.3 Hz, 2H), 7.31 (d, J = 8.0 Hz, 2H), 7.15 – 7.07 (m, 1H), 4.37 (t, J = 6.8 Hz, 2H), 3.90 (t, J = 6.6 Hz, 2H), 3.26 (t, J = 6.8 Hz, 2H), 2.93 (t, J = 6.6 Hz, 2H), 2.58 (s, 3H), 2.48 (s, 3H).

^{13}C NMR (101 MHz, $CDCl_3$) δ 159.3, 152.5, 150.0, 143.2, 140.2, 139.4, 137.3, 135.1, 129.7, 122.1, 121.2, 120.2, 63.7, 47.3, 39.2, 37.7, 14.2, 10.1.

ESI-MS: m/z (%) = 414.14 $(M+H)^+$.

(*E*)-2-(4-((3,5-Dimethyl-1-(3-(pyridin-2-yldisulfaneyl)propyl)-1*H*-pyrazol-4-yl)diazenyl)phenyl)ethan-1-ol (**35**)

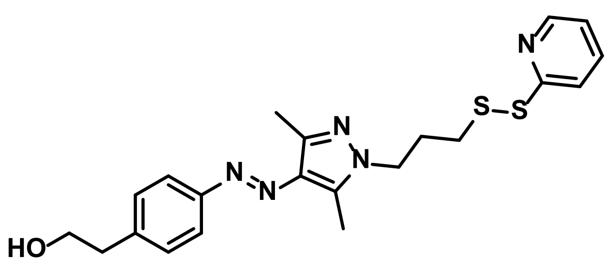

Chemical Formula: $C_{21}H_{25}N_5OS_2$
427,59

Yield: 58%

^{1}H NMR (400 MHz, Methanol-d4) δ 8.37 (ddd, J = 4.9, 1.7, 1.0 Hz, 1H), 7.81 – 7.74 (m, 2H), 7.71 – 7.67 (m, 2H), 7.37 – 7.32 (m, 2H), 7.18 (ddd, J = 6.7, 4.9, 1.8 Hz, 1H), 4.18 (t, J = 6.8 Hz, 2H), 3.80 (t, J = 7.0 Hz, 2H), 2.88 (t, J = 7.0 Hz, 2H), 2.81 (t, J = 7.1 Hz, 2H), 2.58 (s, 3H), 2.42 (s, 3H), 2.24 (p, J = 6.9 Hz, 2H).

^{13}C NMR (101 MHz, MeOD) δ 161.0, 153.4, 150.4, 143.3, 142.5, 140.6, 139.1, 135.9, 130.6, 122.8, 122.4, 121.3, 64.0, 48.0, 40.0, 36.1, 29.8, 14.0, 9.9.

ESI-MS: m/z (%) = 428.16 $(M+H)^+$.

(*E*)-2-(4-((3,5-Dimethyl-1-(4-(pyridin-2-yldisulfaneyl)butyl)-1*H*-pyrazol-4-yl)diazenyl)phenyl)ethan-1-ol (**36**)

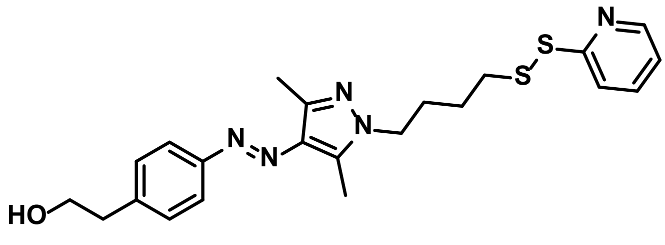

Chemical Formula: $C_{22}H_{27}N_5OS_2$
441,61

Yield: 60%

^{1}H NMR (400 MHz, Chloroform-d) δ 8.41 (ddd, J = 4.9, 1.9, 0.9 Hz, 1H), 7.72 – 7.67 (m, 2H), 7.65 (dt, J = 8.1, 1.1 Hz, 1H), 7.59 (td, J = 7.8, 1.8 Hz, 1H), 7.32 – 7.27 (m, 2H), 7.03 (ddd, J = 7.3, 4.8, 1.2 Hz, 1H),

3.98 (t, J = 7.0 Hz, 2H), 3.87 (t, J = 6.6 Hz, 2H), 2.90 (t, J = 6.6 Hz, 2H), 2.79 (t, J = 7.2 Hz, 2H), 2.50 (s, 3H), 2.46 (s, 3H), 1.97 – 1.88 (m, 2H), 1.72 (dq, J = 10.5, 7.4 Hz, 2H).

^{13}C NMR (101 MHz, $CDCl_3$) δ 160.2, 152.4, 149.7, 142.5, 140.2, 138.3, 137.1, 135.0, 129.6, 122.0, 120.7, 119.8, 63.5, 48.4, 39.1, 38.3, 28.7, 26.1, 14.0, 9.9.

ESI-MS: m/z (%) = 442.17 $(M+H)^+$.

(*E*)-2-(4-((3,5-Dimethyl-1-(5-(pyridin-2-yldisulfaneyl)pentyl)-1*H*-pyrazol-4-yl)diazenyl)phenyl)ethan-1-ol (**37**)

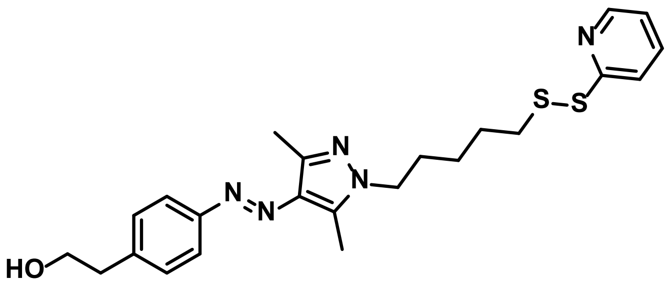

Chemical Formula: $C_{23}H_{29}N_5OS_2$
455,64

Yield: 66%

^{1}H NMR (400 MHz, Chloroform-d) δ 8.45 (dt, J = 5.0, 1.3 Hz, 1H), 7.74 – 7.70 (m, 2H), 7.70 – 7.67 (m, 1H), 7.62 (td, J = 7.7, 1.8 Hz, 1H), 7.31 (d, J = 8.2 Hz, 2H), 7.06 (ddd, J = 7.3, 4.8, 1.1 Hz, 1H), 3.99 (t, J = 7.2 Hz, 2H), 3.89 (t, J = 6.6 Hz, 2H), 2.92 (t, J = 6.6 Hz, 2H), 2.78 (t, J = 7.3 Hz, 2H), 2.54 (s, 3H), 2.48 (s, 3H), 1.83 (p, J = 7.4 Hz, 2H), 1.74 (t, J = 7.5 Hz, 2H), 1.49 – 1.40 (m, 2H).

^{13}C NMR (101 MHz, $CDCl_3$) δ 160.5, 152.6, 149.7, 142.5, 140.1, 138.3, 137.1, 135.1, 129.7, 122.0, 120.7, 119.8, 63.7, 48.8, 39.2, 38.7, 29.7, 28.6, 25.7, 14.1, 10.0.

ESI-MS: m/z (%) = 456.19 $(M+H)^+$.

General procedure for compounds **38-41**

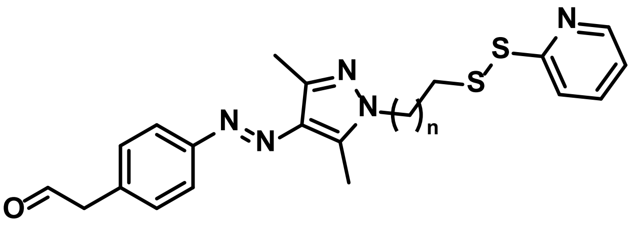

38 (n=1), **39** (n=2), **40** (n=3), **41** (n=4)

Compounds **38**-**41** (1.0 eq.) were dissolved in CH_2Cl_2 under an argon atmosphere and Dess Martin periodinane (1.5 eq.) was added. The mixture was stirred at 0 °C for 1.5 h. Then, EtOAc was added and the mixture was extracted with aqueous sat. $Na_2S_2O_3$, sat. $NaHCO_3$ and sat. NaCl. The combined organic layers were dried over $MgSO_4$ and after filtration the solvent was removed under reduced pressure to get the crude compound which was carried on to the reductive amination immediately without further purification.

General procedure for compounds **3-6**

Compound **18** (1.0 eq.), **38-41** (1.5 eq.) and $NaCNBH_3$ (1.5 eq.) were dissolved in MeOH under an argon atmosphere. The mixture was stirred at rt for overnight. EtOAc was added and the solution was washed with sat. aqueous $NaHCO_3$ solution and H_2O. The organic layer was dried over $MgSO_4$, filtered and the solvent was removed *in vacuo*. The crude product was dissolved in MeOH and cysteamine hydrochloride (2.0 eq.) was added to stir the mixture for 1 h at rt. The solvent was removed *in vacuo* and the crude product was purified by preparative HPLC (column: Luna 10, 250 x 21 mm; flow: 22 mL/min, solvent A: H_2O (0.05% TFA), solvent B: MeCN; gradient A/B: 0-20 min: 90/10, 20-25 min: 2/98) to obtain a yellow solid.

(*E*)-8-(2-((4-((1-(2-((2-Aminoethyl)disulfaneyl)ethyl)-3,5-dimethyl-1*H*-pyrazol-4-yl)diazenyl)phenethyl)amino)-1-hydroxyethyl)-5-hydroxy-2*H*-benzo[*b*][1,4]oxazin-3(4*H*)-one (**3**)

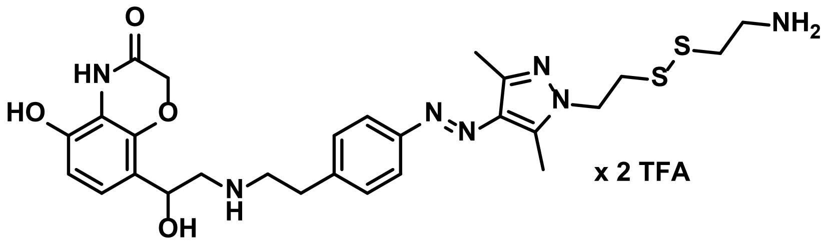

Chemical Formula: $C_{27}H_{35}N_7O_4S_2$
585,74

Yield: 5%

^{1}H NMR (400 MHz, DMSO-d6) δ 10.05 (s, 1H), 9.98 (s, 1H), 8.66 (s, 1H), 7.91 (s, 3H), 7.70 (d, J = 8.3 Hz, 2H), 7.39 (d, J = 8.4 Hz, 2H), 6.92 (d, J = 8.5 Hz, 1H), 6.56 (d, J = 8.5 Hz, 1H), 5.98 (s, 1H), 5.08 (d, J = 8.9 Hz, 1H), 4.53 (d, J = 2.2 Hz, 2H), 4.35 (t, J = 6.7 Hz, 2H), 3.18 (t, J = 6.6 Hz, 4H), 3.12 (d, J = 6.2 Hz, 4H), 2.97 (q, J = 8.7, 6.8 Hz, 4H), 2.59 (s, 3H), 2.38 (s, 3H).

^{13}C NMR (151 MHz, DMSO) δ 164.2, 151.9, 144.9, 141.2, 140.9, 140.0, 138.9, 134.3, 129.5, 121.7, 120.0, 119.8, 115.3, 109.1, 67.0, 62.8, 52.2, 47.9, 47.1, 37.7, 36.5, 34.0, 31.1, 14.0, 9.4.

ESI-MS: m/z (%) = 586.23 $(M+H)^+$.

HR-MS (ESI): calcd. for $C_{27}H_{35}N_7O_4S_2$ $(M+H)^+$, m/z = 586.2265, found 586.2275.

(*E*)-8-(2-((4-((1-(3-((2-Aminoethyl)disulfaneyl)propyl)-3,5-dimethyl-1*H*-pyrazol-4-yl)diazenyl)phenethyl)amino)-1-hydroxyethyl)-5-hydroxy-2*H*-benzo[*b*][1,4]oxazin-3(4*H*)-one (**4**)

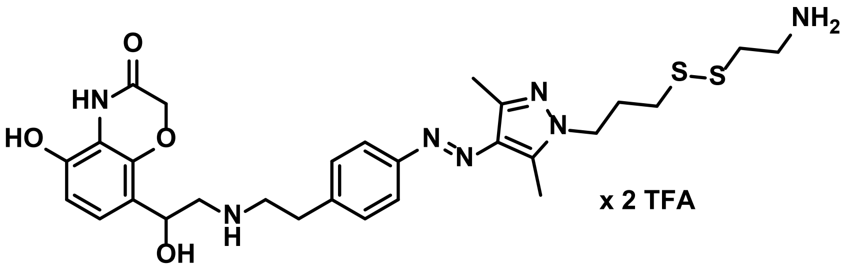

Chemical Formula: $C_{28}H_{37}N_7O_4S_2$
599,77

Yield: 3%

^{1}H NMR (400 MHz, DMSO-d6) δ 10.15 (s, 1H), 9.96 (s, 1H), 8.99 (s, 1H), 8.76 (s, 1H), 8.08 (s, 3H), 7.69 (d, J = 8.0 Hz, 2H), 7.39 (d, J = 8.1 Hz, 2H), 6.92 (d, J = 8.5 Hz, 1H), 6.58 (d, J = 8.5 Hz, 1H), 6.02 (s, 1H), 5.09 (dd, J = 10.1, 2.8 Hz, 1H), 4.53 (d, J = 2.2 Hz, 2H), 4.14 (t, J = 6.8 Hz, 2H), 3.22 (d, J = 8.3 Hz, 2H), 3.10 (q, J = 14.5, 10.8 Hz, 4H), 3.01 (dd, J = 15.3, 7.1 Hz, 2H), 2.93 (t, J = 7.1 Hz, 2H), 2.77 (t, J = 7.1 Hz, 2H), 2.57 (s, 3H), 2.38 (s, 3H), 2.15 (p, J = 7.0 Hz, 2H).

^{13}C NMR (101 MHz, DMSO) δ 164.3, 151.9, 145.0, 141.2, 140.7, 139.5, 138.8, 134.3, 129.5, 121.7, 120.1, 119.8, 115.3, 109.2, 67.0, 62.9, 52.2, 47.9, 46.7, 37.9, 34.2, 33.9, 31.2, 28.6, 14.0, 9.4.

ESI-MS: m/z (%) = 300.63 $(M+2H)^{2+}$.

HR-MS (ESI): calcd. for $C_{28}H_{37}N_7O_4S_2$ $(M+H)^+$, m/z = 600.2421, found 600.2419.

(*E*)-8-(2-((4-((1-(4-((2-Aminoethyl)disulfaneyl)butyl)-3,5-dimethyl-1*H*-pyrazol-4-yl)diazenyl)phenethyl)amino)-1-hydroxyethyl)-5-hydroxy-2*H*-benzo[*b*][1,4]oxazin-3(4*H*)-one (**5**)

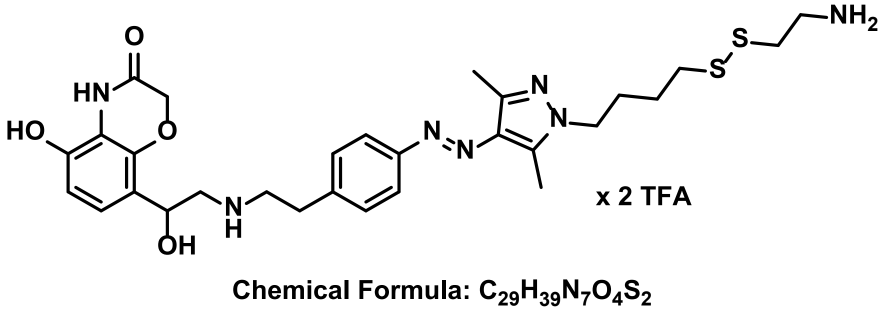

Chemical Formula: $C_{29}H_{39}N_7O_4S_2$
613,80

Yield: 4%

^{1}H NMR (400 MHz, DMSO-d6) δ 10.00 (d, J = 6.6 Hz, 1H), 9.98 (s, 1H), 8.84 (s, 1H), 8.68 (s, 1H), 7.93 (s, 3H), 7.72 – 7.67 (m, 2H), 7.43 – 7.37 (m, 2H), 6.93 (d, J = 8.5 Hz, 1H), 6.57 (d, J = 8.5 Hz, 1H), 5.98 (s, 1H), 5.08 (dd, J = 10.1, 2.8 Hz, 1H), 4.54 (d, J = 1.8 Hz, 2H), 4.08 (t, J = 7.0 Hz, 2H), 3.22 (s, 2H), 3.17 –

3.04 (m, 4H), 3.00 (dd, J = 14.7, 6.7 Hz, 2H), 2.89 (t, J = 7.0 Hz, 2H), 2.79 (t, J = 7.2 Hz, 2H), 2.57 (s, 3H), 2.37 (s, 3H), 1.86 (p, J = 7.1 Hz, 2H), 1.67 (p, J = 7.3 Hz, 2H).

^{13}C NMR (101 MHz, DMSO) δ 164.2, 151.9, 144.9, 141.2, 140.5, 139.3, 138.7, 134.3, 129.5, 121.6, 120.0, 119.8, 115.3, 109.1, 67.0, 62.8, 52.2, 47.9, 47.8, 37.8, 36.8, 34.0, 31.1, 27.9, 25.5, 14.0, 9.4.

ESI-MS: m/z (%) = 307.63 $(M+2H)^{2+}$.

HR-MS (ESI): calcd. for $C_{29}H_{39}N_7O_4S_2$ $(M+H)^+$, m/z = 614.2578, found 614.2575.

<u>(*E*)-8-(2-((4-((1-(5-((2-Aminoethyl)disulfaneyl)pentyl)-3,5-dimethyl-1*H*-pyrazol-4-yl)diazenyl)phenethyl)amino)-1-hydroxyethyl)-5-hydroxy-2*H*-benzo[*b*][1,4]oxazin-3(4*H*)-one (**6**)</u>

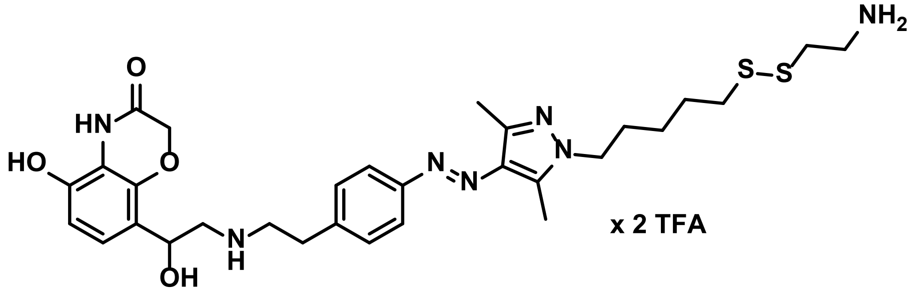

Chemical Formula: $C_{30}H_{41}N_7O_4S_2$
627,82

Yield: 5%

^{1}H NMR (400 MHz, DMSO-d6) δ 10.03 (s, 1H), 9.97 (s, 1H), 8.86 (s, 1H), 8.69 (s, 1H), 7.94 (s, 3H), 7.73 – 7.66 (m, 2H), 7.42 – 7.36 (m, 2H), 6.93 (d, J = 8.5 Hz, 1H), 6.57 (d, J = 8.5 Hz, 1H), 5.98 (s, 1H), 5.09 (dd, J = 10.2, 2.8 Hz, 1H), 4.54 (d, J = 1.9 Hz, 2H), 4.04 (t, J = 7.1 Hz, 2H), 3.22 (s, 2H), 3.17 – 3.05 (m, 4H), 3.02 (d, J = 9.0 Hz, 2H), 2.89 (dd, J = 7.7, 6.4 Hz, 2H), 2.75 (t, J = 7.3 Hz, 2H), 2.56 (s, 3H), 2.37 (s, 3H), 1.78 (p, J = 7.3 Hz, 2H), 1.67 (p, J = 7.4 Hz, 2H), 1.42 – 1.33 (m, 2H).

^{13}C NMR (101 MHz, DMSO) δ 164.2, 151.9, 144.9, 141.2, 140.4, 139.3, 138.7, 134.3, 129.5, 121.6, 120.0, 119.8, 115.3, 109.1, 67.0, 62.8, 52.2, 48.2, 47.9, 37.9, 37.3, 34.0, 31.1, 28.8, 28.1, 24.9, 14.0, 9.4.

ESI-MS: m/z (%) = 314.64 $(M+2H)^{2+}$.

HR-MS (ESI): calcd. for $C_{30}H_{41}N_7O_4S_2$ $(M+H)^+$, m/z = 628.2734, found 628.2734.

<u>General procedure for compounds **30** and **31**</u>

Compound **30** or **31** (1.0 eq.) and 2,2'-dithiodipyridine (1.2 eq.) were dissolved under argon in anhydrous methanol and solution of $NaOCH_3$ (0.5 M in methanol, 1.5 eq.) was added dropwise. The reaction mixture was stirred at rt for 2 h. 2-(Dimethylamino)ethanethiol hydrochloride (4.0 eq.) was added and stirring at room temperature was continued for another 30 min. The solvent was removed under vacuum and the residue was taken up in ethylene acetate. The organic phase was washed with

2 M NaOH and extracted with 2 M HCl solution. The acidic aqueous phase was brought to pH 10 and then extracted with ethylene acetate. The combined organic phases were dried over Na_2SO_4 and the solvent was evaporated. Purification was performed by flash column chromatography (dichloromethane/ethanol/NH_3 conc., 85:14:1).

(*E*)-2-(4-((1-(2-((2-(Dimethylamino)ethyl)disulfaneyl)ethyl)-3,5-dimethyl-1*H*-pyrazol-4-yl)diazenyl)phenyl)ethan-1-ol (**30**)

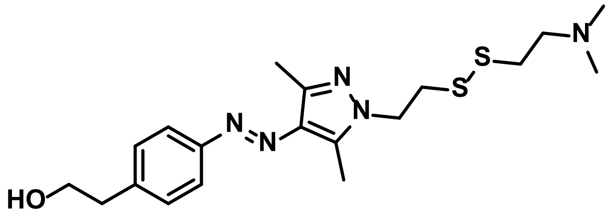

Chemical Formula: $C_{19}H_{29}N_5OS_2$
407,60

Yield: 60%

^{1}H-NMR (400 MHz, Chloroform-d) δ 7.76 – 7.70 (m, 2H), 7.35 – 7.29 (m, 2H), 4.38 – 4.31 (m, 2H), 3.90 (t, J = 6.5 Hz, 2H), 3.15 – 3.09 (m, 2H), 2.93 (t, J = 6.5 Hz, 2H), 2.87 – 2.81 (m, 2H), 2.65 – 2.58 (m, 5H), 2.49 (s, 3H), 2.27 (s, 6H).

^{13}C-NMR (101 MHz, $CDCl_3$) δ 152.4, 142.9, 140.0, 139.3, 135.0, 129.6, 122.0, 63.6, 58.5, 47.5, 45.3, 39.0, 37.5, 36.7, 14.1, 10.0.

ESI-MS: m/z (%) = 408.0 $(M+H)^+$.

(*E*)-2-(4-((1-(3-((2-(Dimethylamino)ethyl)disulfaneyl)propyl)-3,5-dimethyl-1*H*-pyrazol-4-yl)diazenyl)phenyl)ethan-1-ol (**31**)

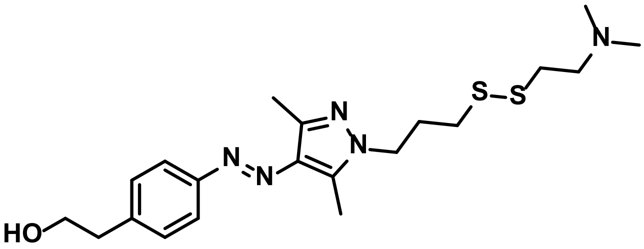

Chemical Formula: $C_{20}H_{31}N_5OS_2$
421,62

Yield: 59%

^{1}H-NMR (400 MHz, Chloroform-d) δ 7.76 – 7.70 (m, 2H), 7.35 – 7.29 (m, 2H), 4.15 (t, J = 6.8 Hz, 2H), 3.90 (t, J = 6.6 Hz, 2H), 2.93 (t, J = 6.5 Hz, 2H), 2.83 – 2.78 (m, 2H), 2.69 (t, J = 6.9 Hz, 2H), 2.62 – 2.57 (m, 5H), 2.49 (s, 3H), 2.31 – 2.24 (m, 8H).

^{13}C-NMR (101 MHz, $CDCl_3$) δ 152.6, 142.8, 140.2, 138.8, 135.2, 129.7, 122.1, 63.7, 58.9, 47.0, 45.5, 39.2, 36.9, 35.3, 28.9, 14.1, 10.1.
ESI-MS: m/z (%) = 422.1 $(M+H)^+$.

General procedure for compounds **32** and **33**

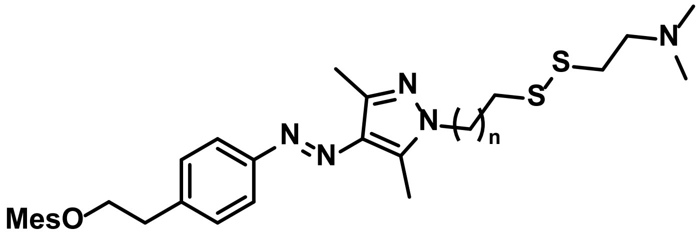

32 (n=1), **33** (n=2)

Compound **32** or **33** (1.0 eq.) was dissolved under argon in anhydrous dichloromethane and cooled to 0 °C. Methanesulfonyl chloride (3.8 eq.) was added dropwise. The mixture was allowed to warm to rt and stirred for 2 h. If the reaction is not completed, K_2CO_3 was added (1.0 eq.). The reaction was quenched with a sat. $NaHCO_3$ solution, ethylene acetate was added and the organic phase was washed with sat. $NaHCO_3$ solution. The organic phase was dried over Na_2SO_4 and the solvent was removed under vacuum. The compounds were used without further purification.

General procedure for compounds **1** and **2**
Compound **32** or **33** (1.0 eq.) was dissolved under argon and exclusion of light in anhydrous DMSO. The solution was added to norepinephrine (4.8 eq.) and the mixture was stirred under argon for 16 h at 70 °C. Water + 0.1% TFA was added and the solution was frozen and lyophilized. Purification was done with preparative HPLC (RP-C8, CH_3CN/water + 0.3% TFA 10-95% in 50 min).

(*R,E*)-4-(2-((4-((1-(2-((2-(Dimethylamino)ethyl)disulfaneyl)ethyl)-3,5-dimethyl-1*H*-pyrazol-4-yl)diazenyl)phenethyl)amino)-1-hydroxyethyl)benzene-1,2-diol (**1**)

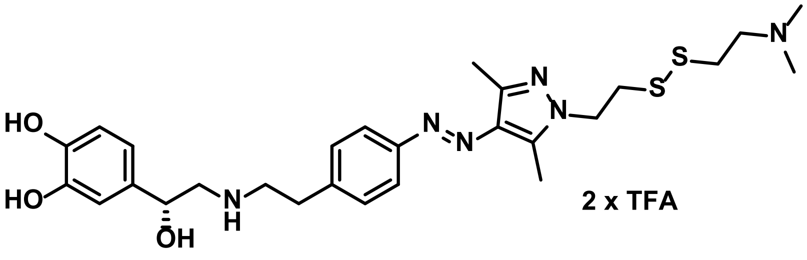

2 x TFA

Chemical Formula: $C_{27}H_{38}N_6O_3S_2$
558,76

Yield: 19%

^{1}H-NMR (600 MHz, DMSO-d6) δ 9.87 (s, 1H), 9.15 – 8.75 (m, 2H), 8.77 – 8.61 (m, 1H), 7.73 – 7.68 (m, 2H), 7.42 – 7.37 (m, 2H), 6.80 (d, J = 2.1 Hz, 1H), 6.73 (d, J = 8.1 Hz, 1H), 6.64 (dd, J = 8.1, 2.1 Hz, 1H), 4.75 (dd, J = 10.3, 3.0 Hz, 1H), 4.36 (t, J = 6.7 Hz, 2H), 3.38 (t, J = 7.8 Hz, 2H), 3.26 – 3.18 (m, 4H), 3.15 – 2.93 (m, 6H), 2.82 (s, 6H), 2.60 (s, 3H), 2.39 (s, 3H).
^{13}C-NMR (151 MHz, DMSO-d6) δ 151.8, 145.2, 144.9, 140.9, 139.9, 138.8, 134.2, 132.4, 129.4, 121.6, 116.7, 115.3, 113.3, 68.0, 55.4, 53.4, 47.7, 47.0, 42.3, 36.6, 31.1, 30.8, 13.9, 9.3.
ESI-MS: m/z (%) = 559.2 $(M+H)^+$.
HR-MS (ESI): calc. for $C_{27}H_{38}N_6O_3S_2$ $(M+H)^+$, m/z 559.2520, found: 559.2525.

<u>(*R,E*)-4-(2-((4-((1-(3-((2-(Dimethylamino)ethyl)disulfaneyl)propyl)-3,5-dimethyl-1*H*-pyrazol-4-yl)diazenyl)phenethyl)amino)-1-hydroxyethyl)benzene-1,2-diol (**2**)</u>

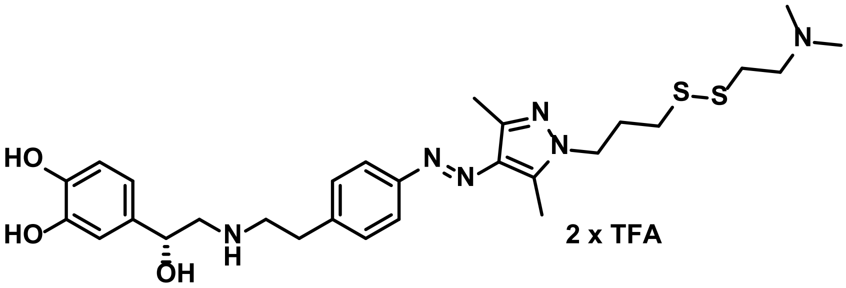

Chemical Formula: $C_{28}H_{40}N_6O_3S_2$
572,79

Yield: 8%
^{1}H-NMR (600 MHz, DMSO-d6) δ 9.90 (s, 1H), 9.30 – 8.90 8.95 (m, 2H), 8.82 (s, 1H), 8.69 (s, 1H), 7.72 – 7.67 (m, 2H), 7.43 – 7.36 (m, 2H), 6.79 (d, J = 2.1 Hz, 1H), 6.73 (d, J = 8.0 Hz, 1H), 6.63 (dd, J = 8.1, 2.1 Hz, 1H), 6.00 (s, 1H), 4.77 – 4.70 (m, 1H), 4.15 (t, J = 6.9 Hz, 2H), 3.39 – 3.30 (m, 3H), 3.25 – 3.17 (m, 2H), 3.15 – 2.96 (m, 4H), 2.85 – 2.75 (m, 9H), 2.58 (s, 3H), 2.38 (s, 4H), 2.20 – 2.11 (m, 2H).
^{13}C-NMR (151 MHz, DMSO-d6) δ 151.8, 145.1, 144.9, 140.6, 139.4, 138.7, 134.2, 132.4, 129.4, 121.5, 116.7, 115.3, 113.3, 68.0, 55.4, 53.4, 47.7, 46.6, 42.2, 34.2, 31.1, 30.7, 28.3, 13.9, 9.3.
ESI-MS: m/z (%) = 573.3 $(M+H)^+$.
HR-MS (ESI): calc. for $C_{28}H_{40}N_6O_3S_2$ $(M+H)^+$, m/z 573.2676, found: 573.2681.

<u>General procedure for compounds **42** and **43**</u>
Compound **22** or **23** (1.0 eq.) and 3a,4,7,7a-tetrahydro-1*H*-4,7-epoxyisoindole-1,3(2*H*)-dione (1.2 eq.) were dissolved in DMF. K_2CO_3 (3.0 eq.) was added and the mixture was stirred overnight at 50 °C. Water was added and the mixture was extracted with EtOAc. The organic phase was washed with water and brine. The combined organic phases were dried over $MgSO_4$ and the solvent was removed in vacuo. The crude product was purified by column chromatography (PE/EtOAc, 20-100%).

(*E*)-2-(2-(4-((4-(2-Hydroxyethyl)phenyl)diazenyl)-3,5-dimethyl-1*H*-pyrazol-1-yl)ethyl)-3a,4,7,7a-tetrahydro-1*H*-4,7-epoxyisoindole-1,3(2*H*)-dione (**42**)

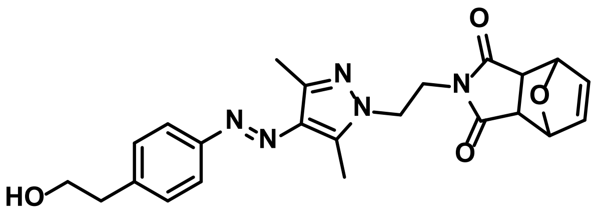

Chemical Formula: $C_{23}H_{25}N_5O_4$
435,48

Yield: 50%

^{1}H NMR (400 MHz, Chloroform-*d*) δ 7.72 (d, *J* = 8.3 Hz, 2H), 7.31 (d, *J* = 8.3 Hz, 2H), 6.49 (s, 2H), 5.24 (s, 2H), 4.22 (t, *J* = 6.4 Hz, 2H), 3.93 – 3.85 (m, 4H), 2.92 (t, *J* = 6.6 Hz, 2H), 2.83 (s, 2H), 2.57 (s, 3H), 2.45 (s, 3H).

^{13}C NMR (101 MHz, $CDCl_3$) δ 175.8, 152.5, 143.0, 140.2, 139.2, 136.7, 135.4, 129.7, 122.1, 81.0, 63.7, 47.6, 45.7, 39.2, 38.3, 14.1, 9.8.

ESI-MS: m/z (%) = 436.20 $(M+H)^+$.

(*E*)-2-(3-(4-((4-(2-Hydroxyethyl)phenyl)diazenyl)-3,5-dimethyl-1*H*-pyrazol-1-yl)propyl)-3a,4,7,7a-tetrahydro-1*H*-4,7-epoxyisoindole-1,3(2*H*)-dione (**43**)

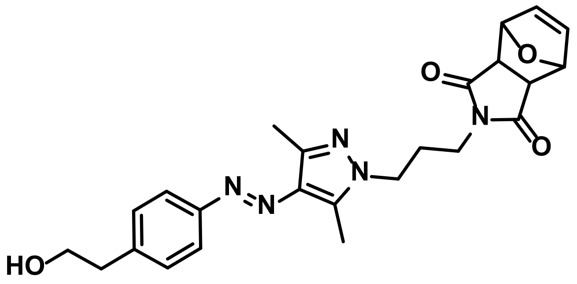

Chemical Formula: $C_{24}H_{27}N_5O_4$
449,51

Yield: 45%

^{1}H NMR (400 MHz, Chloroform-*d*) δ 7.70 (d, *J* = 8.3 Hz, 2H), 7.30 (d, *J* = 8.3 Hz, 2H), 6.50 (s, 2H), 5.25 (s, 2H), 4.02 – 3.96 (m, 2H), 3.88 (q, *J* = 6.3 Hz, 2H), 3.59 (t, *J* = 6.7 Hz, 2H), 2.91 (t, *J* = 6.6 Hz, 2H), 2.81 (s, 2H), 2.52 (s, 3H), 2.46 (s, 3H), 2.21 – 2.11 (m, 2H).

^{13}C NMR (101 MHz, $CDCl_3$) δ 176.2, 152.5, 142.5, 140.1, 138.7, 136.6, 135.2, 129.6, 122.1, 81.1, 63.7, 47.5, 46.5, 39.2, 36.6, 27.8, 14.1, 9.8.

ESI-MS: m/z (%) = 450.21 $(M+H)^+$.

<u>General procedure for compounds **44** and **45**</u>

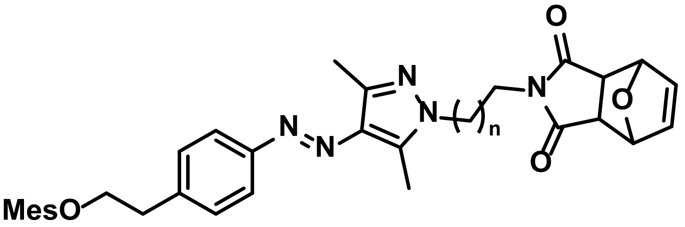

44 (n=1), **45** (n=2)

Compound **42** or **43** (1.0 eq.) was dissolved in CH_2Cl_2 and the mixture was cooled to 0 °C. Triethylamine (1.5 equiv.) and methanesulfonyl chloride (1.1 equiv.) were added and the mixture was warmed up to rt over 1 h. Water was added, and the aqueous phase was extracted with CH_2Cl_2. The solvent was removed *in vacuo*. The crude product was used in the next step without further purification.

<u>General procedure for compounds **46-48**</u>

Compound **44** or **45** (1.0 eq.) and **18** or norepinephrine (3.0 equiv.) were dissolved in DMSO in an argon atmosphere. The solution was stirred overnight at 70 °C. The crude product was purified by preparative HPLC (column: Luna 10, 250 x 21 mm; flow: 22 mL/min, solvent A: H_2O (0.05% TFA), solvent B: MeCN; gradient A/B: 0-20 min: 90/10, 20-25 min: 2/98) to obtain a yellow solid.

<u>2-(2-(4-((E)-(4-(2-(((R)-2-(3,4-Dihydroxyphenyl)-2-hydroxyethyl)amino)ethyl)phenyl)diazenyl)-3,5-dimethyl-1*H*-pyrazol-1-yl)ethyl)-3a,4,7,7a-tetrahydro-1*H*-4,7-epoxyisoindole-1,3(2*H*)-dione (**46**)</u>

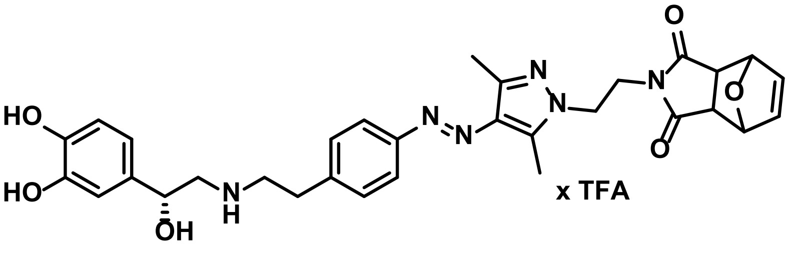

Chemical Formula: $C_{31}H_{34}N_6O_6$
586,65

Yield: 10%

^{1}H NMR (400 MHz, DMSO-d_6) δ 8.95 (s, 2H), 8.67 (s, 2H), 7.70 (d, *J* = 8.4 Hz, 2H), 7.39 (d, *J* = 8.4 Hz, 2H), 6.79 (d, *J* = 2.1 Hz, 1H), 6.73 (d, *J* = 8.1 Hz, 1H), 6.63 (dd, *J* = 8.1, 2.1 Hz, 1H), 6.53 (s, 2H), 6.00 (s, 1H), 5.11 (s, 2H), 4.73 (dd, *J* = 10.5, 2.9 Hz, 1H), 4.17 (t, *J* = 6.3 Hz, 2H), 3.74 (t, *J* = 6.3 Hz, 2H), 3.21 (q, *J* = 6.8 Hz, 2H), 3.04 (ddt, *J* = 22.1, 13.6, 6.4 Hz, 4H), 2.53 (s, 3H), 2.34 (s, 3H).

^{13}C NMR (101 MHz, DMSO) δ 176.1, 151.9, 145.3, 145.0, 140.9, 140.0, 138.8, 136.5, 134.4, 132.5, 129.5, 121.7, 116.8, 115.4, 113.4, 109.5, 80.3, 68.2, 53.5, 47.8, 47.2, 45.4, 37.5, 31.2, 13.9, 9.1.
ESI-MS: m/z (%) = 587.26 $(M+H)^+$.

(*E*)-2-(2-(4-((4-(2-((2-Hydroxy-2-(5-hydroxy-3-oxo-3,4-dihydro-2*H*-benzo[*b*][1,4]oxazin-8-yl)ethyl)amino)ethyl)phenyl)diazenyl)-3,5-dimethyl-1*H*-pyrazol-1-yl)ethyl)-3a,4,7,7a-tetrahydro-1*H*-4,7-epoxyisoindole-1,3(2*H*)-dione (**47**)

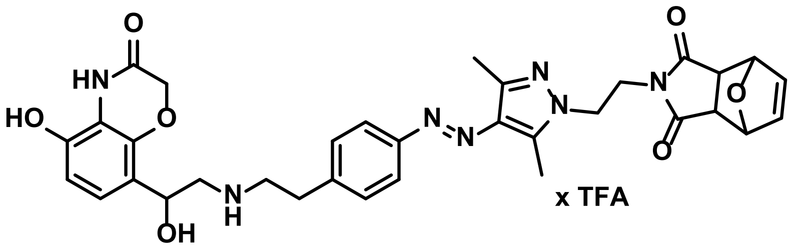

Chemical Formula: $C_{33}H_{35}N_7O_7$
641,69

Yield: 17%
^{1}H NMR (400 MHz, DMSO-*d*$_6$) δ 10.00 (s, 1H), 9.98 (s, 1H), 8.81 (s, 1H), 8.66 (s, 1H), 7.73 – 7.67 (m, 2H), 7.39 (d, *J* = 8.4 Hz, 2H), 6.93 (d, *J* = 8.5 Hz, 1H), 6.57 (d, *J* = 8.5 Hz, 1H), 6.53 (s, 2H), 5.97 (s, 1H), 5.10 (d, *J* = 1.0 Hz, 2H), 5.10 – 5.05 (m, 1H), 4.54 (d, *J* = 2.2 Hz, 2H), 4.18 (t, *J* = 6.3 Hz, 2H), 3.74 (t, *J* = 6.3 Hz, 2H), 3.22 (s, 2H), 3.18 – 3.06 (m, 2H), 3.04 – 2.98 (m, 2H), 2.93 (s, 2H), 2.53 (s, 3H), 2.34 (s, 3H).
^{13}C NMR (101 MHz, DMSO) δ 176.1, 164.2, 151.9, 144.9, 141.2, 140.9, 140.0, 138.8, 136.5, 134.4, 129.5, 121.7, 120.0, 119.8, 115.3, 109.1, 80.3, 67.0, 62.8, 52.2, 47.9, 47.2, 45.3, 37.5, 31.1, 13.9, 9.1.
ESI-MS: m/z (%) = 642.27 $(M+H)^+$.

(*E*)-2-(3-(4-((4-(2-((2-Hydroxy-2-(5-hydroxy-3-oxo-3,4-dihydro-2*H*-benzo[*b*][1,4]oxazin-8-yl)ethyl)amino)ethyl)phenyl)diazenyl)-3,5-dimethyl-1*H*-pyrazol-1-yl)propyl)-3a,4,7,7a-tetrahydro-1*H*-4,7-epoxyisoindole-1,3(2*H*)-dione (**48**)

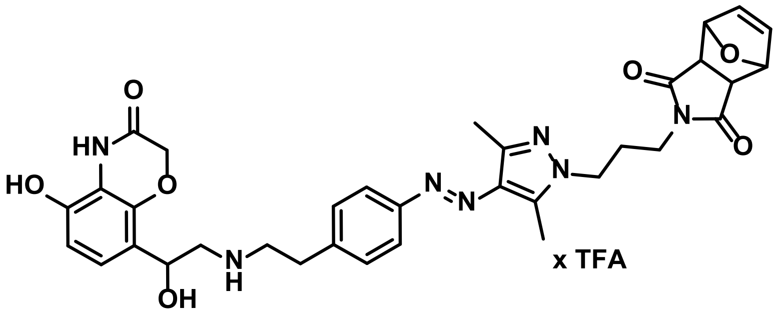

Chemical Formula: $C_{34}H_{37}N_7O_7$
655,71

Yield: 18%

^{1}H NMR (400 MHz, DMSO-d_6) δ 9.99 (d, *J* = 10.4 Hz, 2H), 8.80 (s, 1H), 8.66 (s, 1H), 7.69 (d, *J* = 8.3 Hz, 2H), 7.39 (d, *J* = 8.2 Hz, 2H), 6.92 (d, *J* = 8.5 Hz, 1H), 6.58 (s, 1H), 6.55 (d, *J* = 1.3 Hz, 2H), 5.97 (s, 1H), 5.14 (s, 2H), 5.08 (dd, *J* = 10.0, 2.9 Hz, 1H), 4.54 (d, *J* = 2.3 Hz, 2H), 3.99 (t, *J* = 7.2 Hz, 2H), 3.45 (d, *J* = 7.5 Hz, 2H), 3.21 (d, *J* = 8.8 Hz, 2H), 3.17 – 3.06 (m, 2H), 3.04 – 2.98 (m, 2H), 2.91 (s, 2H), 2.52 (s, 3H), 2.36 (s, 3H), 1.99 (p, *J* = 7.2 Hz, 2H).
^{13}C NMR (101 MHz, DMSO) δ 176.5, 164.2, 152.0, 144.9, 141.2, 140.6, 139.6, 138.7, 136.5, 134.3, 129.5, 121.7, 120.1, 119.8, 115.3, 109.2, 80.4, 67.0, 62.8, 52.2, 48.0, 47.2, 45.8, 35.6, 31.2, 27.0, 14.0, 9.2.
ESI-MS: m/z (%) = 656.28 $(M+H)^+$.

General procedures for compounds **7-9**

Compounds **46**-**48** were dissolved in DMSO under an argon atmosphere. The mixture was stirred at 110 °C for 3-4 h. The crude product was purified by preparative HPLC (column: Luna 10, 250 x 21 mm; flow: 22 mL/min, solvent A: H_2O (0.05% TFA), solvent B: MeCN; gradient A/B: 0-20 min: 90/10, 20-25 min: 2/98) to obtain a yellow solid.

(*R,E*)-1-(2-(4-((4-(2-((2-(3,4-Dihydroxyphenyl)-2-hydroxyethyl)amino)ethyl)phenyl)diazenyl)-3,5-dimethyl-1*H*-pyrazol-1-yl)ethyl)-1*H*-pyrrole-2,5-dione (**7**)

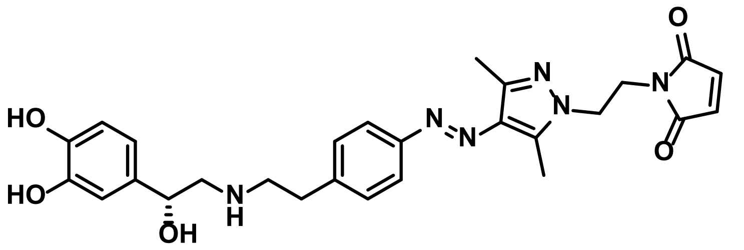

Chemical Formula: $C_{27}H_{30}N_6O_5$
518,57

Yield: 85%
^{1}H-NMR (600 MHz, DMSO-d6) δ 8.94 (s, 2H), 8.63 (s, 2H), 7.73 – 7.67 (m, 2H), 7.41 – 7,37 (m, 2H), 7.03 (s, 2H), 6.79 (d, J = 2.1 Hz, 1H), 6.73 (d, J = 8.1 Hz, 1H), 6.63 (dd, J = 8.1, 2.1 Hz, 1H), 6.02-5.96 (m, 1H), 4.76-4.69 (m, 1H), 4.23 (t, J = 6.0 Hz, 2H), 3.78 (t, J = 5.9 Hz, 2H), 3.26 – 3.19 (m, 2H), 3.17 – 2.89 (m, 4H), 2.53 (s, 3H), 2.30 (s, 3H).
^{13}C-NMR (151 MHz, DMSO-d6) δ 171.1, 152.3, 145.7, 145.5, 141.3, 140.1, 139.3, 135.2, 133.0, 130.0, 122.2, 117.3, 115.8, 113.9, 68.6, 53.9, 48.2, 46.7, 37.2, 31.6, 14.2, 9.6.
ESI-MS: m/z (%) = 519.2 $(M+H)^+$.
HR-MS (ESI): calc. for $C_{27}H_{30}N_6O_5$ $(M+H)^+$, m/z = 519.2350, found: 519.2361.

(*E*)-1-(2-(4-((4-(2-((2-Hydroxy-2-(5-hydroxy-3-oxo-3,4-dihydro-2*H*-benzo[*b*][1,4]oxazin-8-yl)ethyl)amino)ethyl)phenyl)diazenyl)-3,5-dimethyl-1*H*-pyrazol-1-yl)ethyl)-1*H*-pyrrole-2,5-dione (**8**)

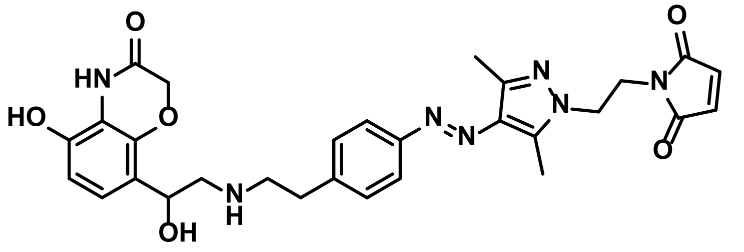

Chemical Formula: $C_{29}H_{31}N_7O_6$
573,61

Yield: 98%

^{1}H NMR (400 MHz, DMSO-d_6) δ 10.00 (s, 1H), 9.98 (s, 1H), 8.81 (s, 1H), 8.66 (s, 1H), 7.70 (d, *J* = 8.2 Hz, 2H), 7.39 (d, *J* = 8.2 Hz, 2H), 7.03 (s, 2H), 6.92 (d, *J* = 8.5 Hz, 1H), 6.57 (d, *J* = 8.5 Hz, 1H), 5.97 (s, 1H), 5.12 – 5.02 (m, 1H), 4.54 (d, *J* = 2.2 Hz, 2H), 4.22 (t, *J* = 5.9 Hz, 2H), 3.78 (t, *J* = 5.9 Hz, 2H), 3.21 (d, *J* = 7.8 Hz, 2H), 3.12 (s, 2H), 3.04 – 2.97 (m, 2H), 2.52 (s, 3H), 2.30 (s, 3H).

^{13}C NMR (101 MHz, DMSO) δ 170.6, 164.2, 151.9, 144.9, 141.2, 140.8, 139.7, 138.8, 134.7, 134.5, 129.5, 121.7, 120.0, 119.8, 115.3, 109.5, 67.0, 62.8, 47.9, 46.2, 46.2, 36.7, 31.1, 13.8, 9.2.

ESI-MS: m/z (%) = 574.24 $(M+H)^+$.

HR-MS (ESI): calc. for $C_{29}H_{31}N_7O_6$ $(M+H)^+$, m/z = 574.2409, found: 574.2419.

(*E*)-1-(3-(4-((4-(2-((2-Hydroxy-2-(5-hydroxy-3-oxo-3,4-dihydro-2*H*-benzo[*b*][1,4]oxazin-8-yl)ethyl)amino)ethyl)phenyl)diazenyl)-3,5-dimethyl-1*H*-pyrazol-1-yl)propyl)-1*H*-pyrrole-2,5-dione (**9**)

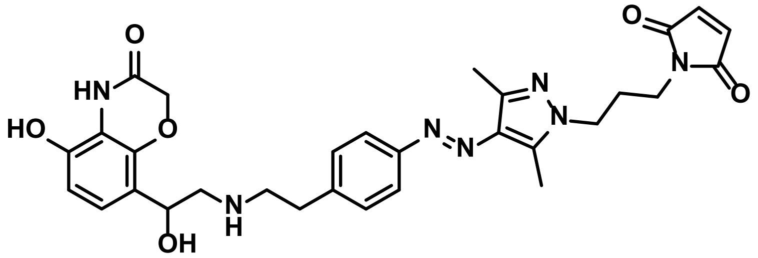

Chemical Formula: $C_{30}H_{33}N_7O_6$
587,64

Yield: 95%

^{1}H NMR (400 MHz, DMSO-d_6) δ 9.99 (s, 1H), 9.98 (s, 1H), 8.79 (s, 1H), 8.65 (s, 1H), 7.70 (d, *J* = 8.4 Hz, 2H), 7.39 (d, *J* = 8.4 Hz, 2H), 6.99 (s, 2H), 6.92 (d, *J* = 8.5 Hz, 1H), 6.57 (d, *J* = 8.5 Hz, 1H), 5.97 (s, 1H), 5.08 (d, *J* = 9.8 Hz, 1H), 4.54 (d, *J* = 2.2 Hz, 2H), 4.05 (t, *J* = 6.9 Hz, 2H), 3.48 (t, *J* = 6.9 Hz, 2H), 3.22 (s, 2H), 3.18 – 3.04 (m, 2H), 3.00 (dd, *J* = 15.1, 7.1 Hz, 2H), 2.53 (s, 3H), 2.35 (s, 3H), 2.09 – 2.03 (m, 2H).

^{13}C NMR (101 MHz, DMSO) δ 171.0, 164.2, 151.9, 144.9, 141.2, 140.5, 139.6, 138.7, 134.5, 134.3, 129.5, 121.7, 120.0, 119.8, 115.3, 109.1, 67.0, 62.8, 52.2, 47.9, 46.0, 35.0, 31.1, 27.6, 14.0, 9.3.

ESI-MS: m/z (%) = 588.26 $(M+H)^+$.

HR-MS (ESI): calc. for $C_{30}H_{33}N_7O_6$ $(M+H)^+$, m/z = 588.2565, found: 588.2573.

1.3. Purity

The purity of all compounds was measured on analytical HPLC at a detection wavelength of 220 nm.

Purity was measured either directly after purification or in DMSO.

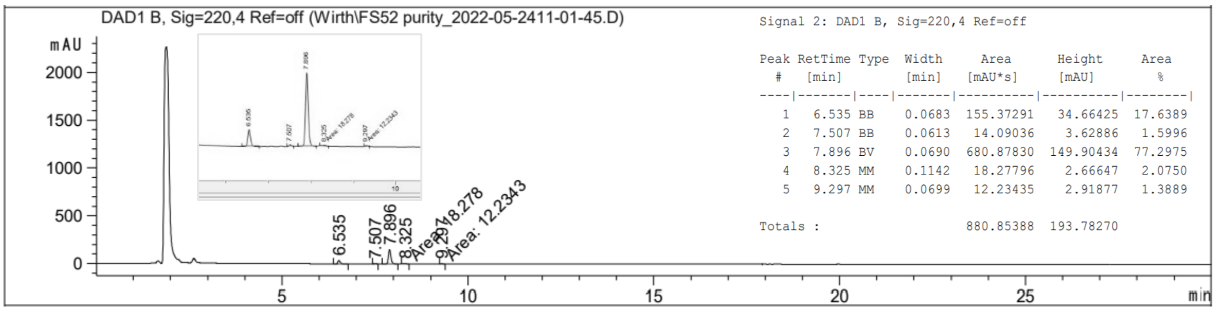

Signal 2: DAD1 B, Sig=220,4 Ref=off

Peak #	RetTime [min]	Type	Width [min]	Area [mAU*s]	Height [mAU]	Area %
1	6.535	BB	0.0683	155.37291	34.66425	17.6389
2	7.507	BB	0.0613	14.09036	3.62886	1.5996
3	7.896	BV	0.0690	680.87830	149.90434	77.2975
4	8.325	MM	0.1142	18.27796	2.66647	2.0750
5	9.297	MM	0.0699	12.23435	2.91877	1.3889
Totals :				880.85388	193.78270	

Figure S1. Purity of **1** (95%); *Z* isomer: 6.535 min, *E* isomer: 7.896 min.

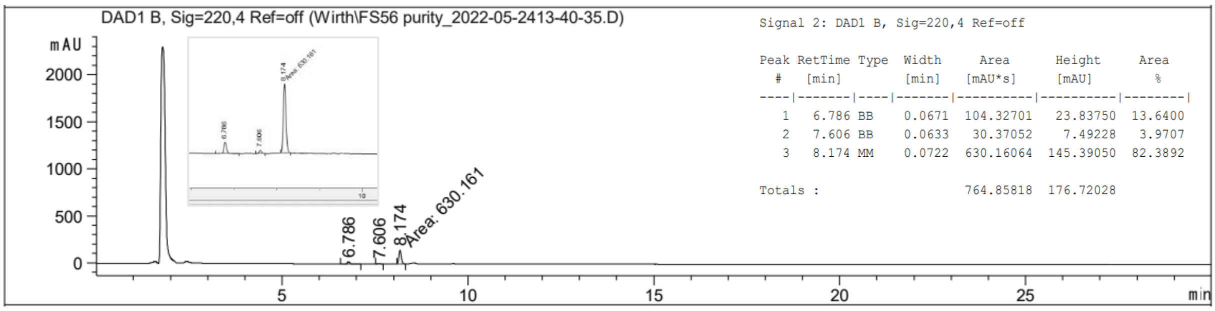

Signal 2: DAD1 B, Sig=220,4 Ref=off

Peak #	RetTime [min]	Type	Width [min]	Area [mAU*s]	Height [mAU]	Area %
1	6.786	BB	0.0671	104.32701	23.83750	13.6400
2	7.606	BB	0.0633	30.37052	7.49228	3.9707
3	8.174	MM	0.0722	630.16064	145.39050	82.3892
Totals :				764.85818	176.72028	

Figure S2. Purity of **2** (96%); *Z* isomer: 6.786 min, *E* isomer: 8.174 min.

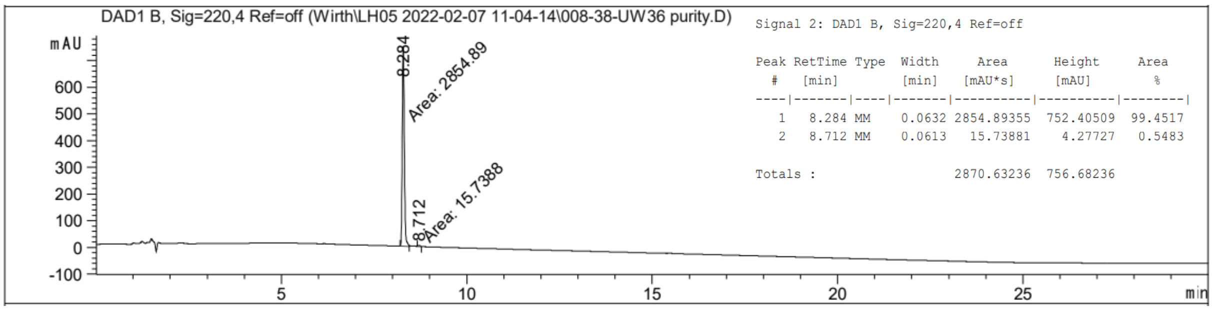

Signal 2: DAD1 B, Sig=220,4 Ref=off

Peak #	RetTime [min]	Type	Width [min]	Area [mAU*s]	Height [mAU]	Area %
1	8.284	MM	0.0632	2854.89355	752.40509	99.4517
2	8.712	MM	0.0613	15.73881	4.27727	0.5483
Totals :				2870.63236	756.68236	

Figure S3. Purity of **3** (99%).

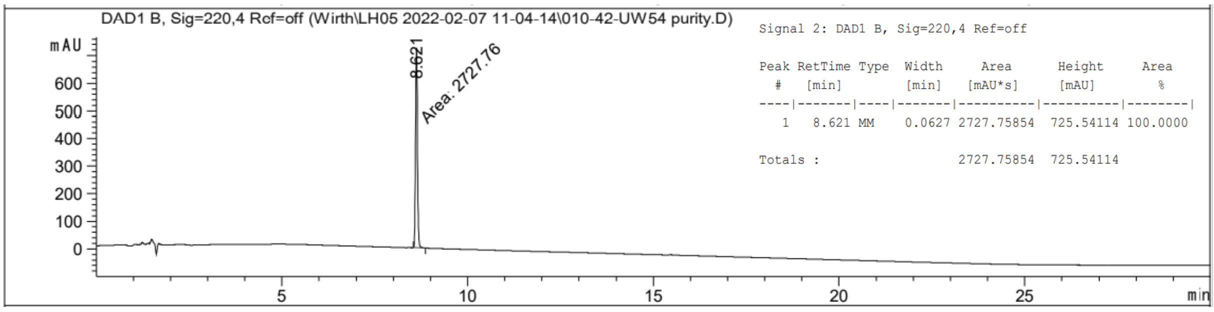

Signal 2: DAD1 B, Sig=220,4 Ref=off

Peak #	RetTime [min]	Type	Width [min]	Area [mAU*s]	Height [mAU]	Area %
1	8.621	MM	0.0627	2727.75854	725.54114	100.0000
Totals :				2727.75854	725.54114	

Figure S4. Purity of **4** (>99%).

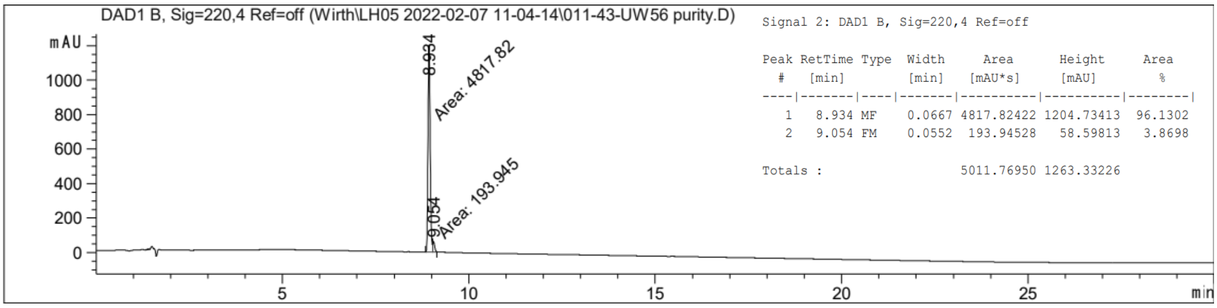

Figure S5. Purity of **5** (96%).

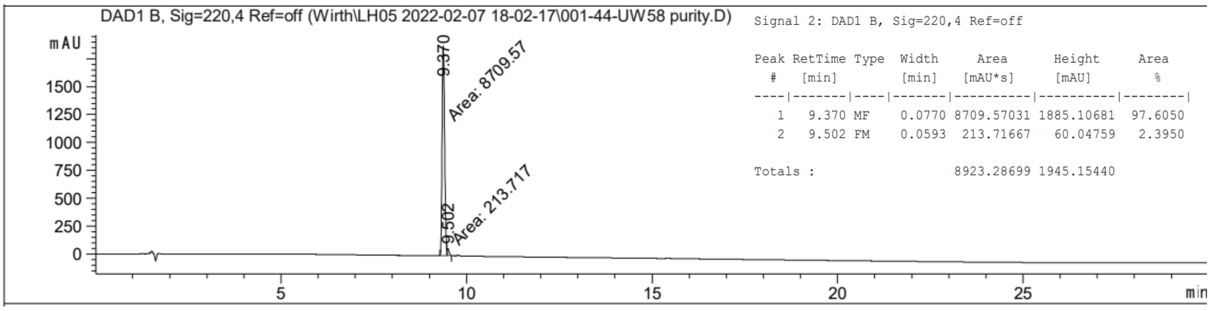

Figure S6. Purity of **6** (98%).

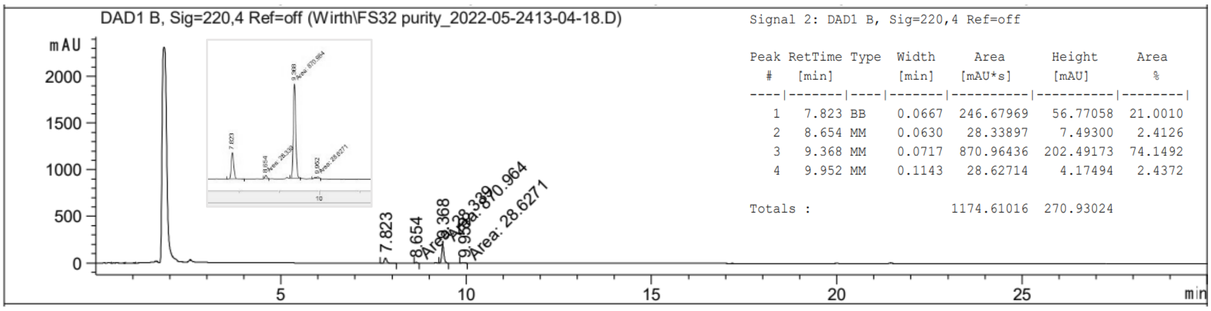

Figure S7. Purity of **7** (95%); *Z* isomer: 7.823 min, *E* isomer: 9.368 min.

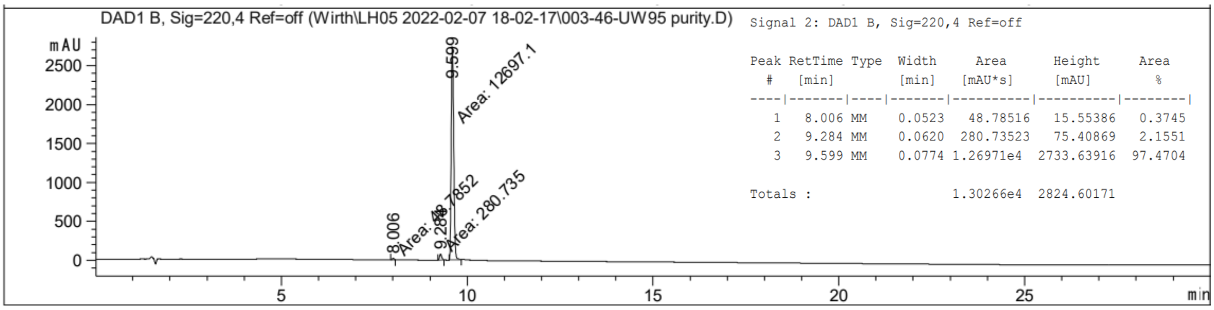

Figure S8. Purity of **8** (97%).

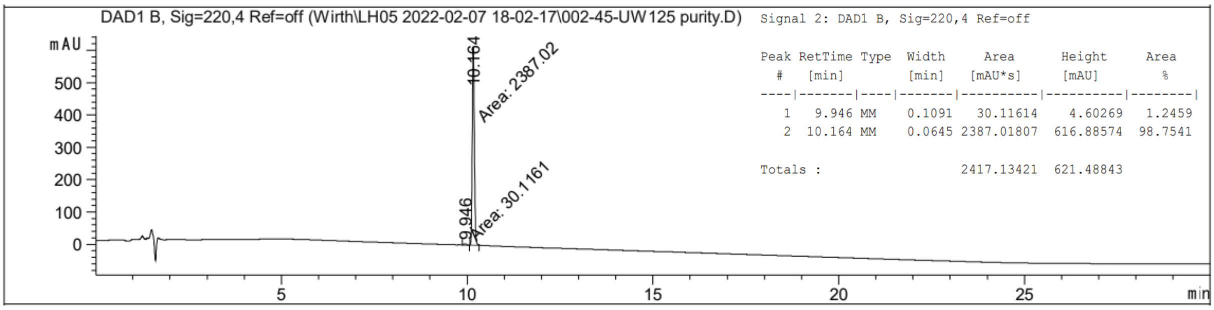

Figure S9. Purity of **9** (99%).

1.4. Photophysical Characterization

1.4.1. UV/Vis Spectra and Cycle Performance

UV/Vis absorption spectra were measured to characterize the photophysical properties of the synthesized photoswitches. The photoswitches were diluted with TRIS buffer (50 mM Tris, 1 mM EDTA, 1 mM $MgCl_2$, pH 7.4) to end up with a concentration of 10 µM + 0.5% DMSO. The spectra were measured in a quartz glass cuvette after different illuminations with LED modules. First, the solution was illuminated with 365 nm light for 10 s to switch to the *Z*-isomer. After measuring the UV/Vis spectrum of the *Z*-isomer (purple curve), the solution was illuminated with 528 nm light for 120 s and the spectrum for the *E*-isomer (black curve) was measured. This cycle was repeated ten times to show the stability and repeatability of switching the compound. The isosbestic points could be determined from an overlay of the UV/Vis spectra of both isomers.

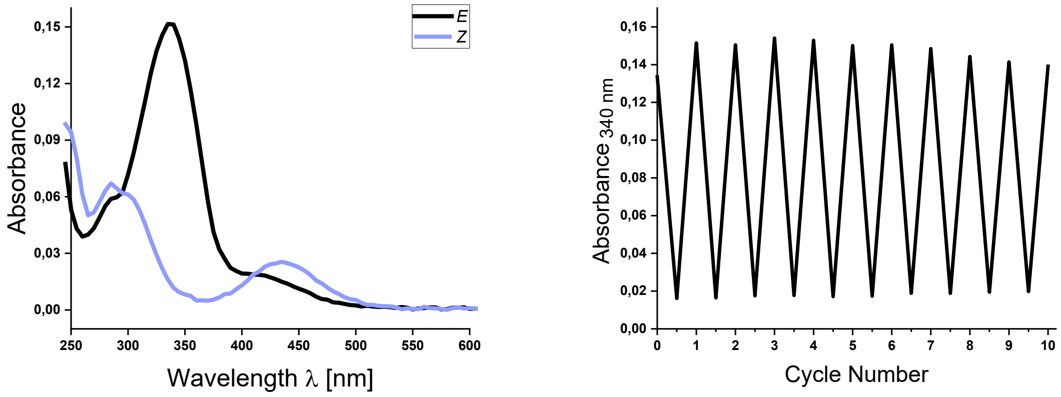

Figure S10. UV/Vis spectrum and cycle performance of **1**.

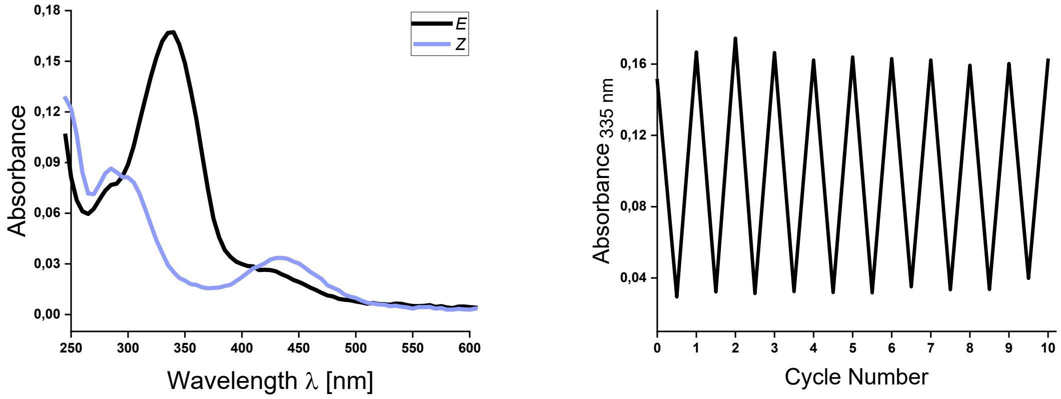

Figure S11. UV/Vis spectrum and cycle performance of **2**.

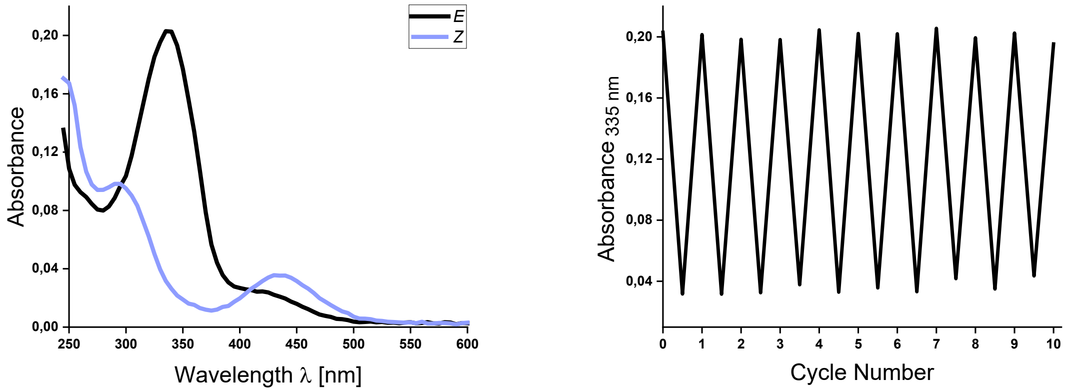

Figure S12. UV/Vis spectrum and cycle performance of **3**.

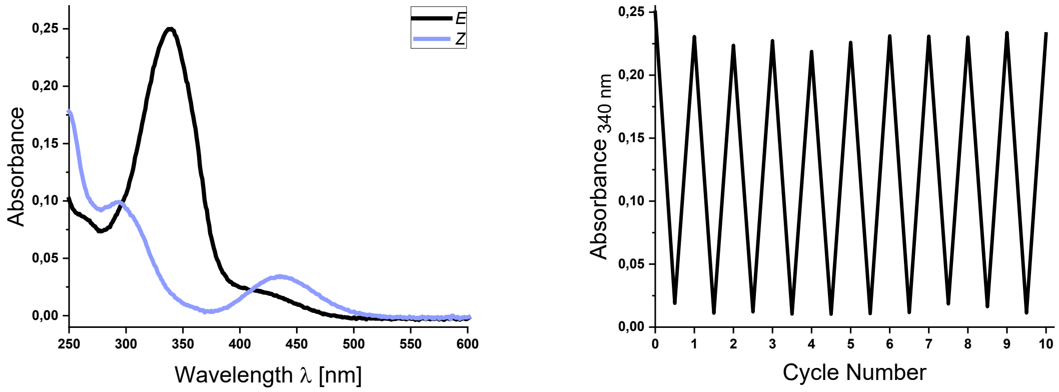

Figure S13. UV/Vis spectrum and cycle performance of **4**.

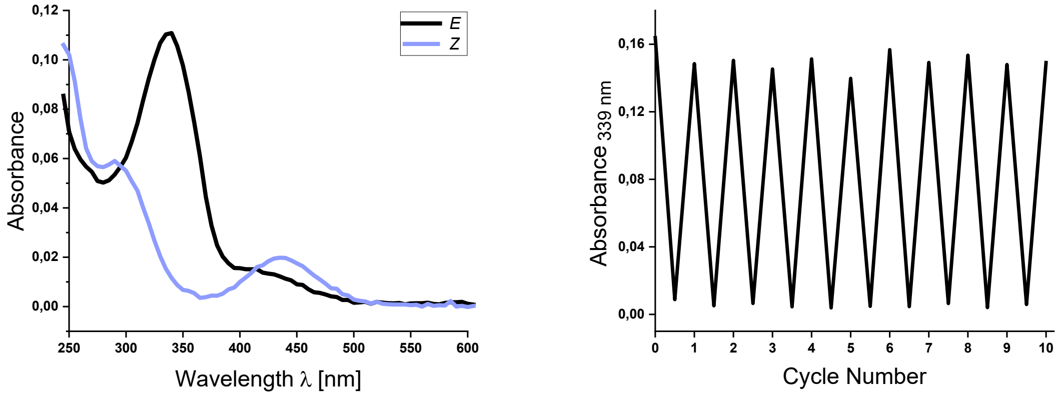

Figure S14. UV/Vis spectrum and cycle performance of **5**.

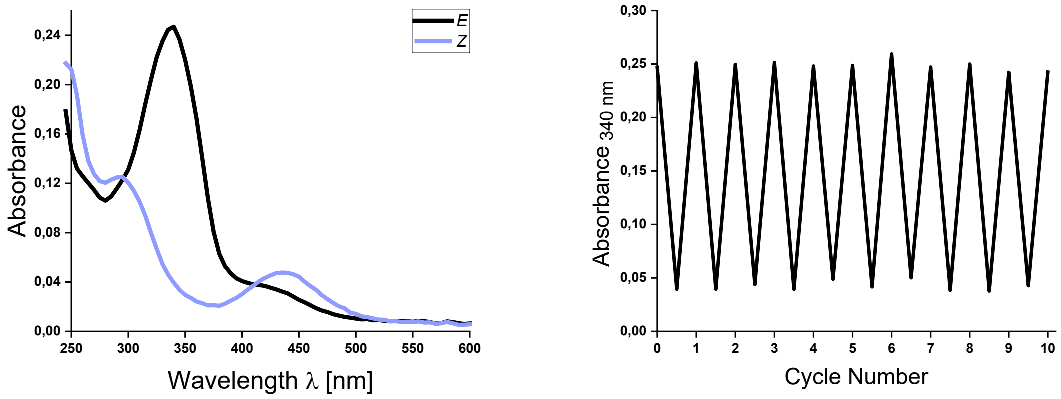

Figure S15. UV/Vis spectrum and cycle performance of **6**.

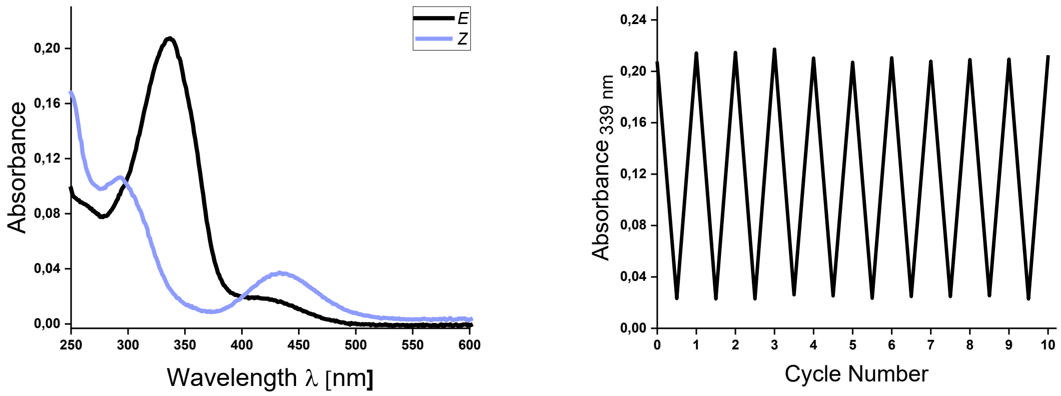

Figure S16. UV/Vis spectrum and cycle performance of **8**.

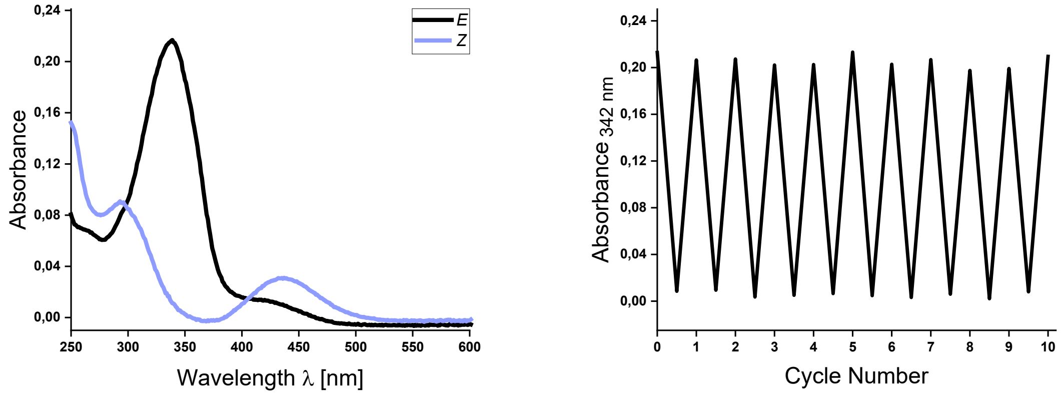

Figure S17. UV/Vis spectrum and cycle performance of **9**.

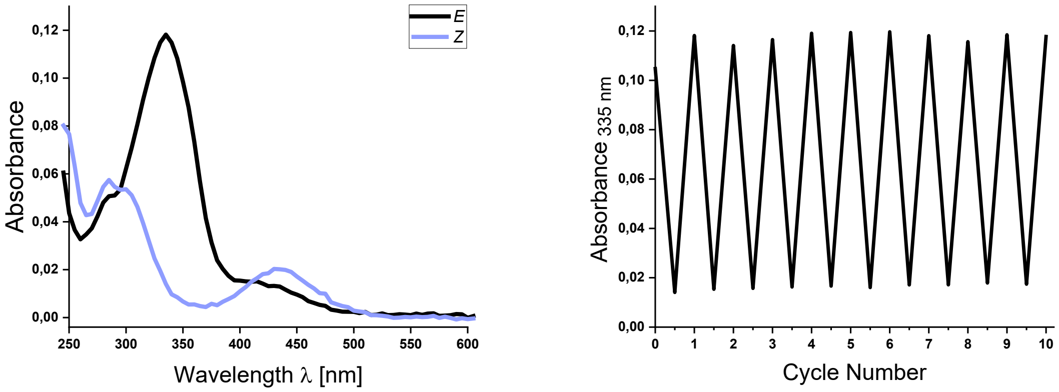

Figure S18. UV/Vis spectrum and cycle performance of **7**.

1.4.2. Photostationary States

Photostationary states (PSS) were measured on analytical HPLC. To determine the photostationary state of the photoswitches the samples (0.2 mM in Tris buffer + 2% DMSO) were irradiated first with 365 nm to get the *Z*-isomer (Peak 1). Afterwards, the sample was irradiated with 528 nm to get back to the *E*-isomer (Peak 2). The samples were measured at the isosbestic points.

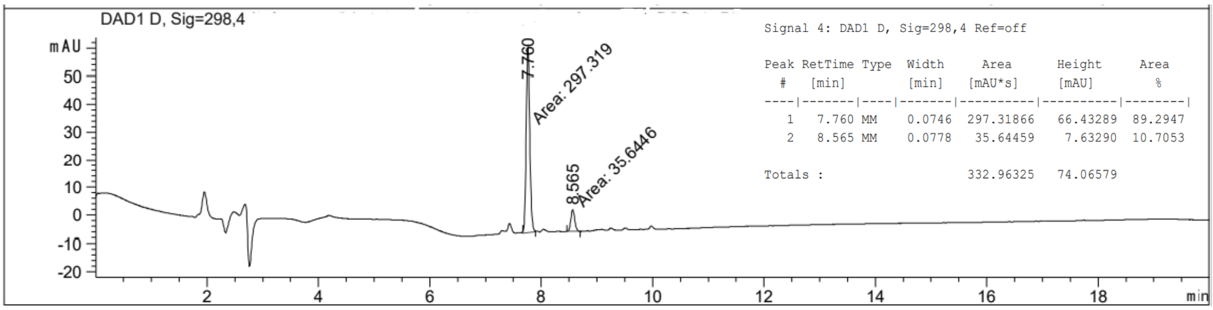

Figure S19. Photostationary state of **1** after irradiation with 365 nm (switching to the *Z*-isomer).

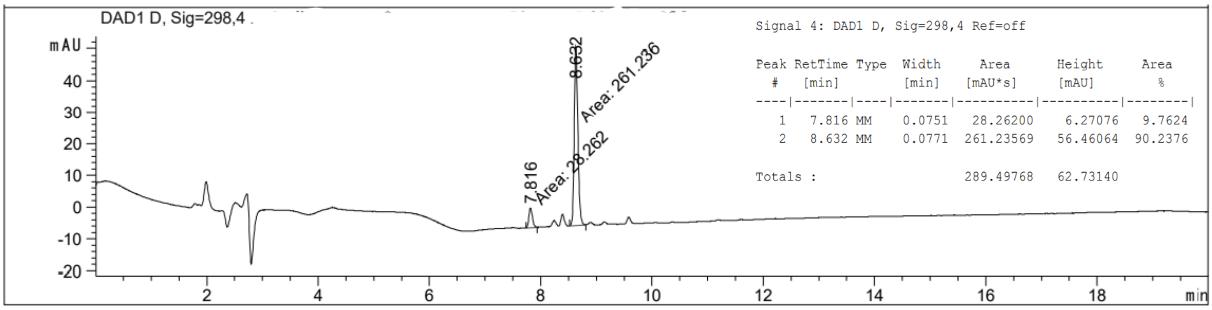

Figure S20. Photostationary state of **1** after irradiation with 528 nm (switching to the *E*-isomer).

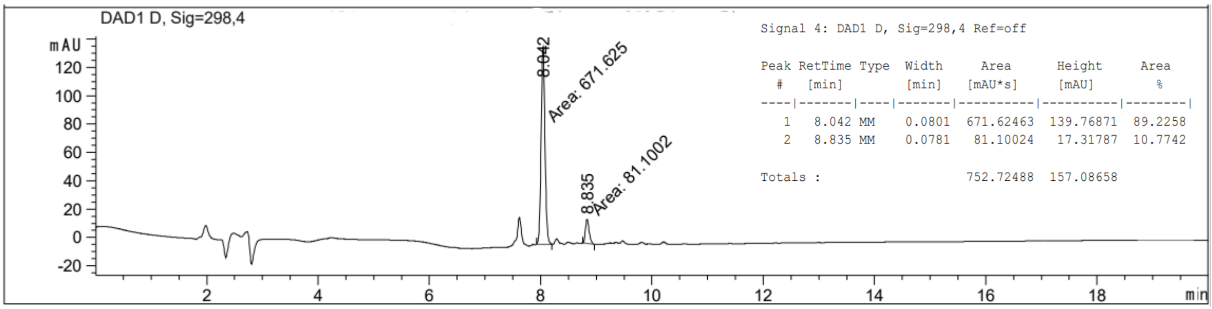

Figure S21. Photostationary state of **2** after irradiation with 365 nm (switching to the *Z*-isomer).

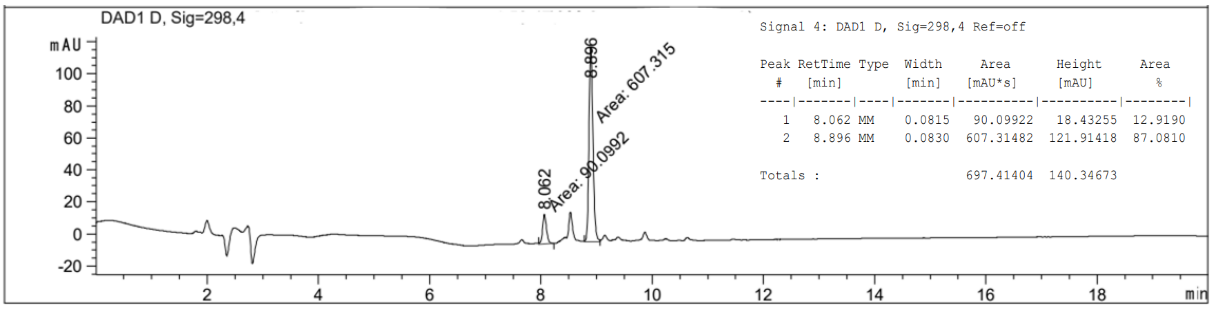

Figure S22. Photostationary state of **2** after irradiation with 528 nm (switching to the *E*-isomer).

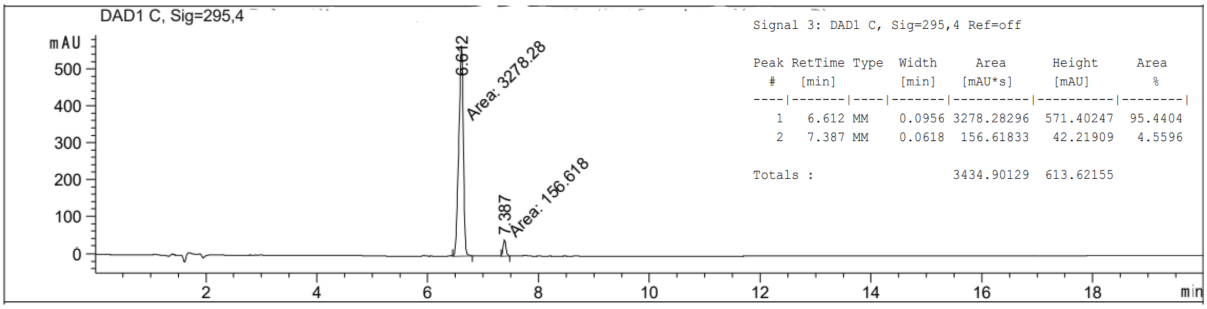

Figure S23. Photostationary state of **3** after irradiation with 365 nm (switching to the *Z*-isomer).

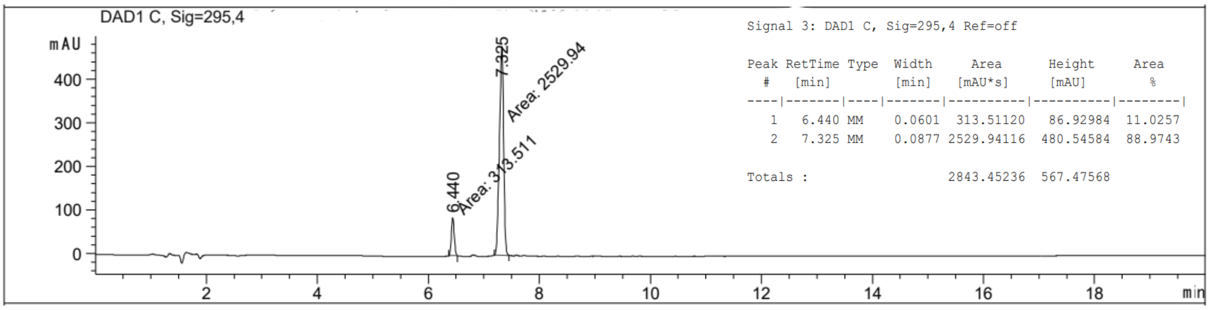

Figure S24. Photostationary state of **3** after irradiation with 528 nm (switching to the *E*-isomer).

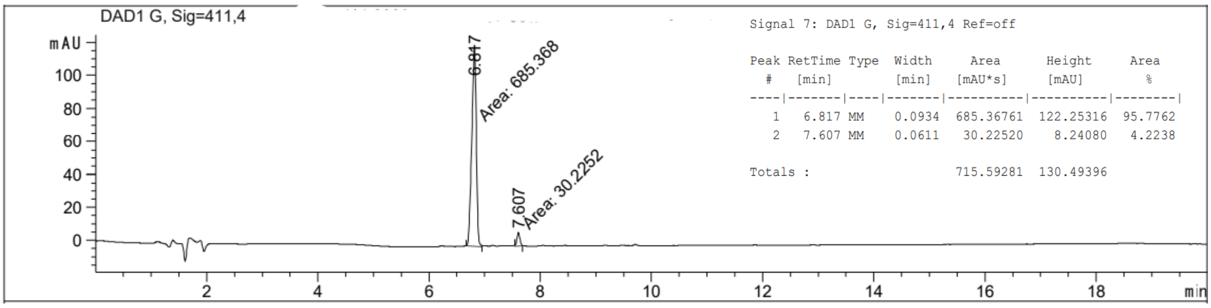

Figure S25. Photostationary state of **4** after irradiation with 365 nm (switching to the *Z*-isomer).

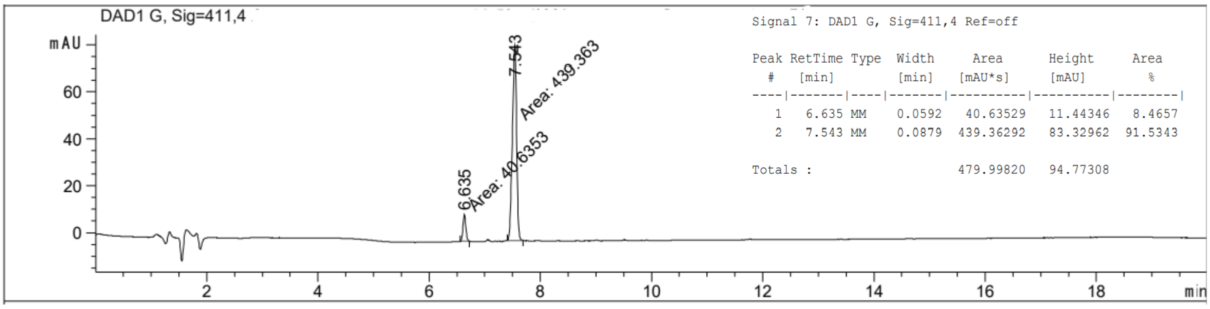

Figure S26. Photostationary state of **4** after irradiation with 528 nm (switching to the *E*-isomer).

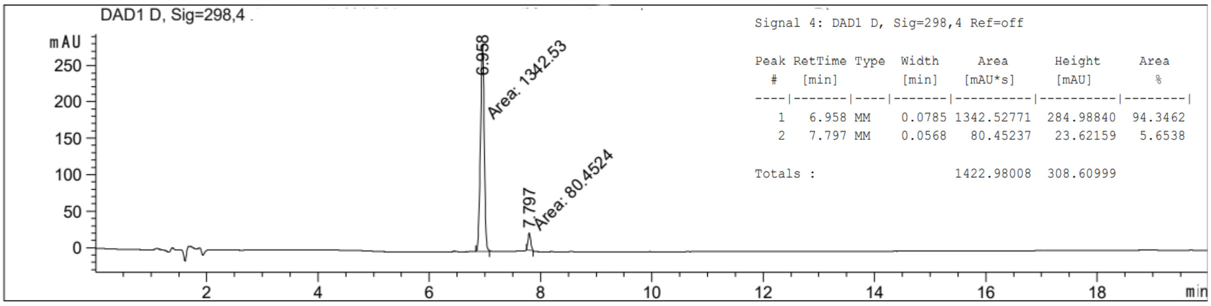

Figure S27. Photostationary state of **5** after irradiation with 365 nm (switching to the *Z*-isomer).

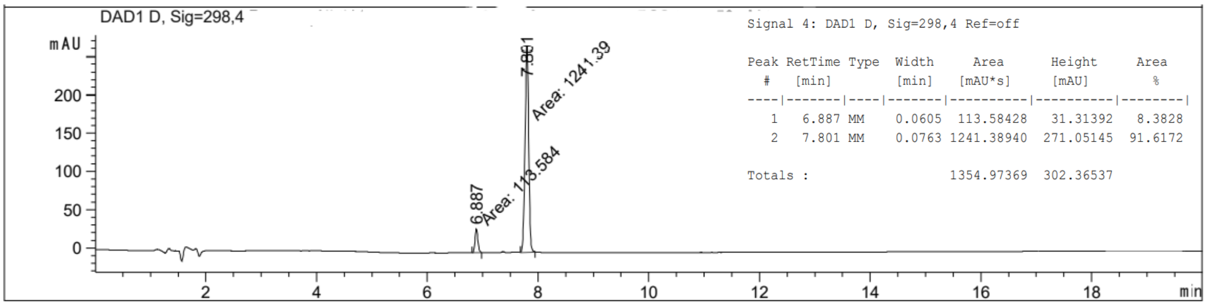

Figure S28. Photostationary state of **5** after irradiation with 528 nm (switching to the *E*-isomer).

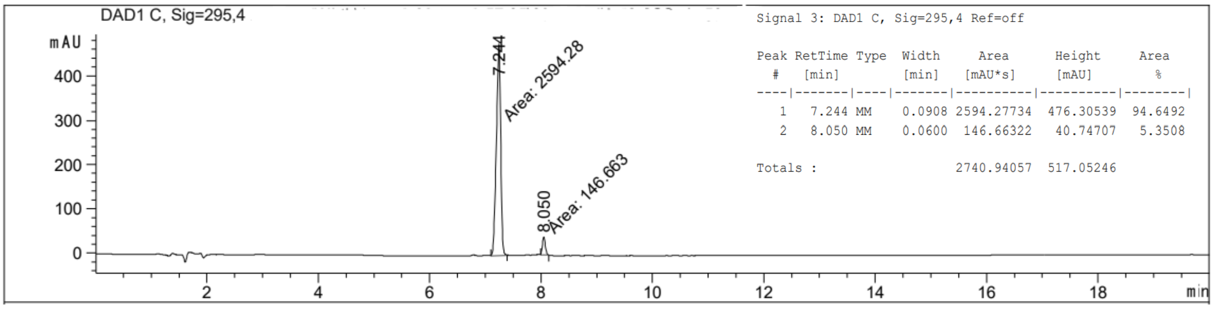

Figure S29. Photostationary state of **6** after irradiation with 365 nm (switching to the *Z*-isomer).

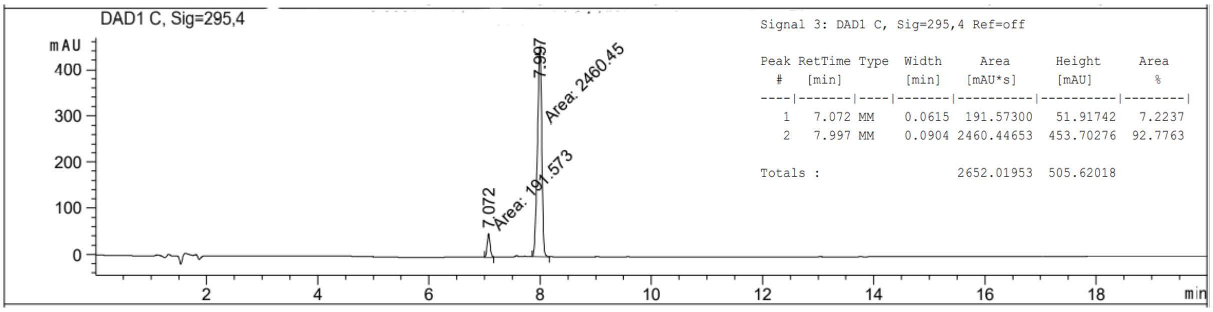

Figure S30. Photostationary state of **6** after irradiation with 528 nm (switching to the *E*-isomer).

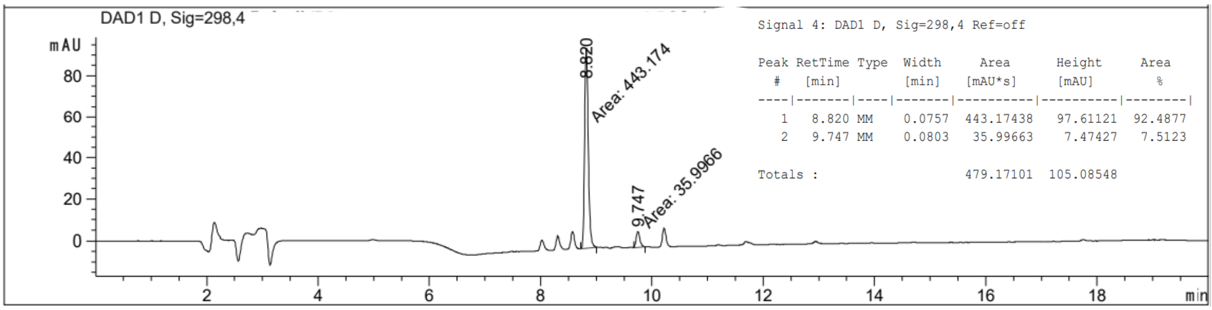

Figure S31. Photostationary state of **7** after irradiation with 365 nm (switching to the *Z*-isomer).

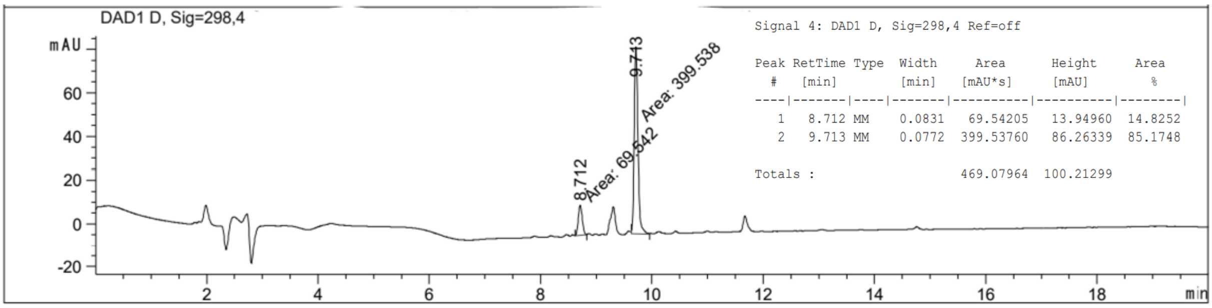

Figure S32. Photostationary state of **7** after irradiation with 528 nm (switching to the *E*-isomer).

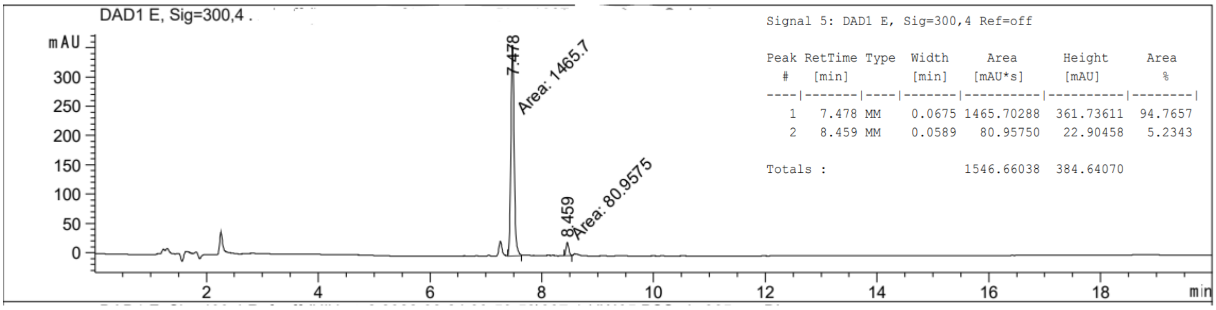

Figure S33. Photostationary state of **8** after irradiation with 365 nm (switching to the *Z*-isomer).

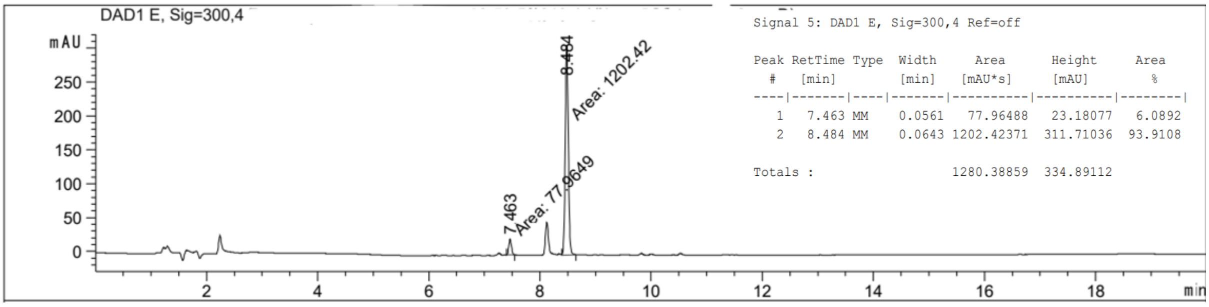

Figure S34. Photostationary state of **8** after irradiation with 528 nm (switching to the *E*-isomer).

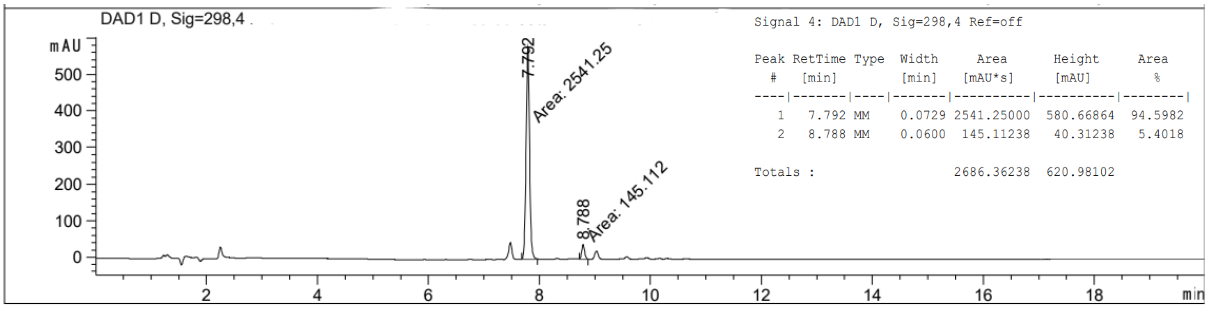

Figure S35. Photostationary state of **9** after irradiation with 365 nm (switching to the *Z*-isomer).

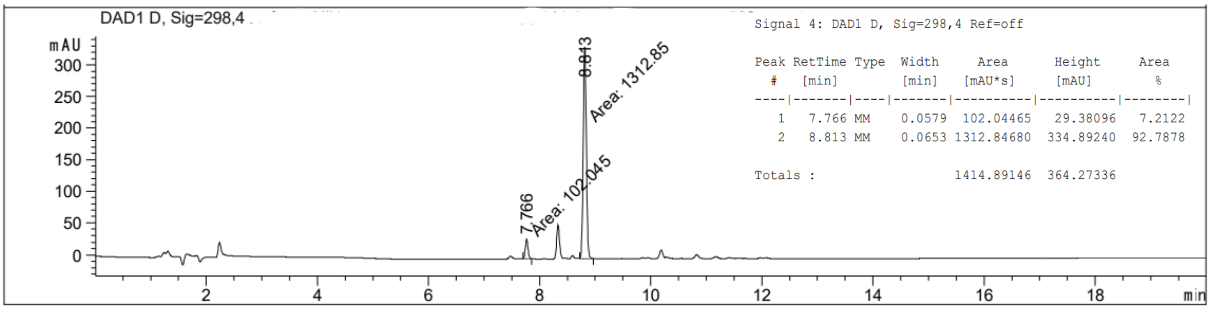

Figure S36. Photostationary state of **9** after irradiation with 528 nm (switching to the *E*-isomer).

1.4.3. Thermal Half-lives

For the determination of the thermal stability of the metastable *Z* isomer a 50 µM solution in Tris buffer + 0.5% DMSO was irradiated with 365 nm. For 4 weeks the absorption at 335 nm was measured every 3 h.

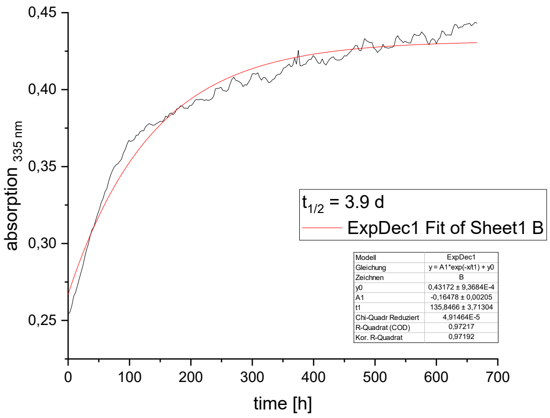

1

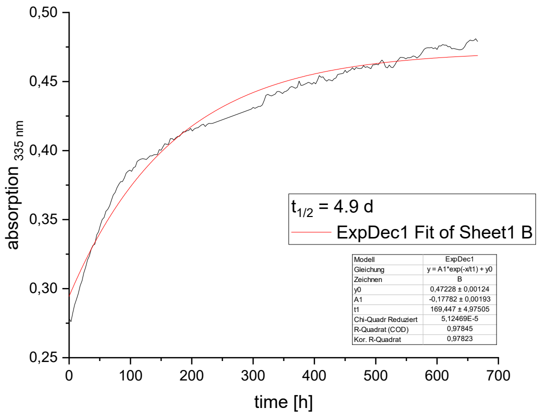

2

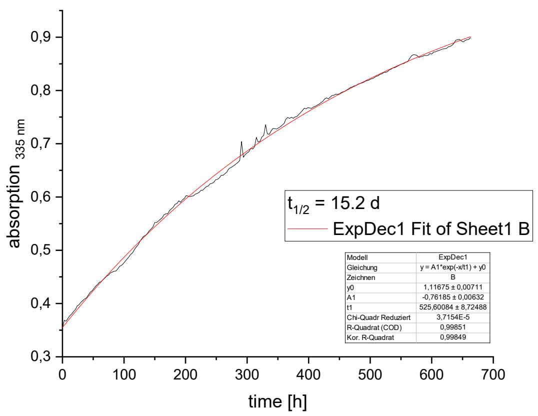
absorption 335 nm
0,3
0,4
0,5
0,6
0,7
0,8
0,9
0
100
200
300
400
500
600
700
time [h]
t1/2 = 15.2 d
ExpDec1 Fit of Sheet1 B
Modell ExpDec1
Gleichung y = A1*exp(-x/t1) + y0
Zeichnen B
y0 1,11675 ± 0,00711
A1 -0,76185 ± 0,00632
t1 525,60084 ± 8,72488
Chi-Quadr Reduziert 3,7154E-5
R-Quadrat (COD) 0,99851
Kor. R-Quadrat 0,99849

3

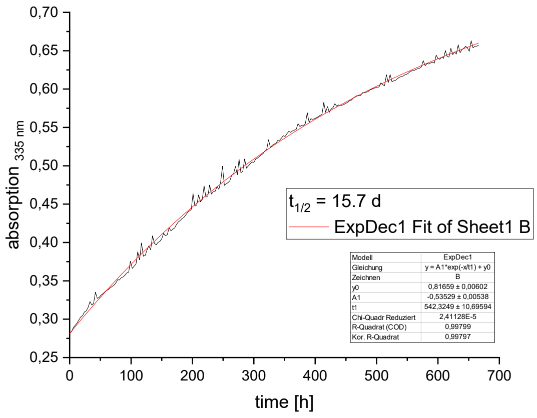
absorption 335 nm
0,25
0,30
0,35
0,40
0,45
0,50
0,55
0,60
0,65
0,70
0
100
200
300
400
500
600
700
time [h]
t1/2 = 15.7 d
ExpDec1 Fit of Sheet1 B
Modell ExpDec1
Gleichung y = A1*exp(-x/t1) + y0
Zeichnen B
y0 0,81659 ± 0,00602
A1 -0,53529 ± 0,00538
t1 542,3249 ± 10,69594
Chi-Quadr Reduziert 2,41128E-5
R-Quadrat (COD) 0,99799
Kor. R-Quadrat 0,99797

4

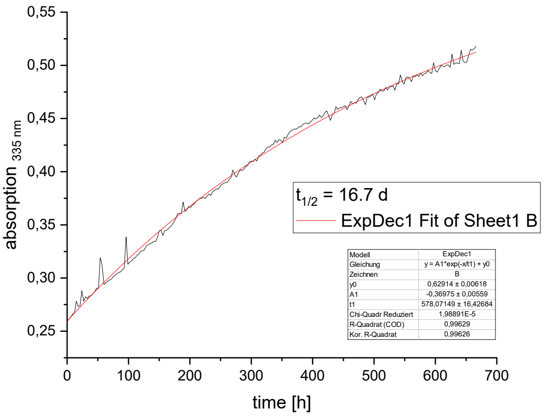
absorption 335 nm
0,25
0,30
0,35
0,40
0,45
0,50
0,55
0
100
200
300
400
500
600
700
time [h]
t1/2 = 16.7 d
ExpDec1 Fit of Sheet1 B
Modell ExpDec1
Gleichung y = A1*exp(-x/t1) + y0
Zeichnen B
y0 0,62914 ± 0,00618
A1 -0,36975 ± 0,00559
t1 578,07149 ± 16,42684
Chi-Quadr Reduziert 1,98891E-5
R-Quadrat (COD) 0,99629
Kor. R-Quadrat 0,99626

5

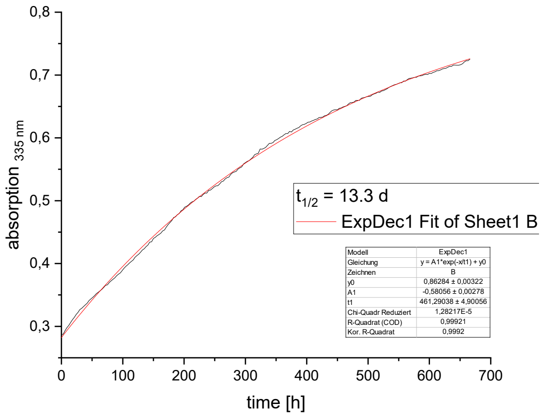
absorption 335 nm
0,3
0,4
0,5
0,6
0,7
0,8
0
100
200
300
400
500
600
700
time [h]
t1/2 = 13.3 d
ExpDec1 Fit of Sheet1 B
Modell ExpDec1
Gleichung y = A1*exp(-x/t1) + y0
Zeichnen B
y0 0,86284 ± 0,00322
A1 -0,58056 ± 0,00278
t1 461,29038 ± 4,90056
Chi-Quadr Reduziert 1,28217E-5
R-Quadrat (COD) 0,99921
Kor. R-Quadrat 0,9992

6

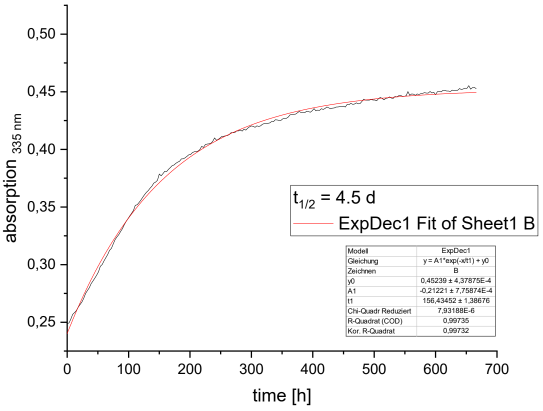

7

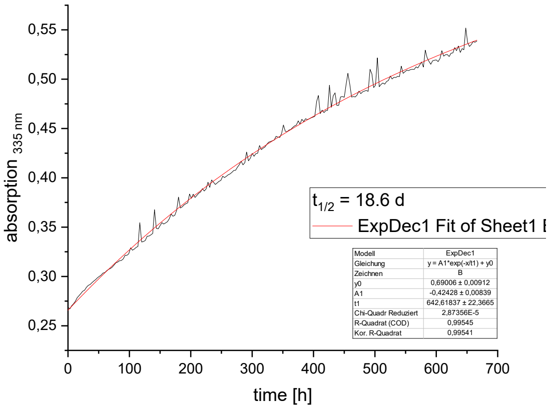

8

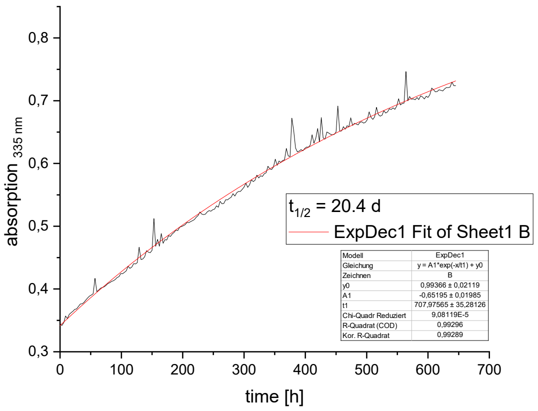

9

Figure S37. Thermal half-lives of compounds **1**-**9**.

2. Biological Investigations

Mammalian cell culture

Cell culture and transfection

HEK-293T cells were cultured in DMEM/F12 medium supplemented with 10% fetal bovine serum, 100 µg/mL penicillin, 100 µg/mL streptomycin, and 2 mM L-glutamine in culture dishes (10 cm). They were incubated at 37 °C and 5% CO_2 to grow to a confluency of 70 – 100%. To prepare cells for the IP-One assay, HEK-293T cells were transfected with the Mirus TransIT-293 reagent according to manufacturer's protocol when mixing the appropriate cDNA β_2AR wt (purchased from cDNA Resource Center, Bloomsburg University, Bloomsberg, PA) or β_2K97$^{2.68}$C (engineered by side-directed mutagenesis of β_2AR wt applying the quikchange method) and the hybrid G-protein $G\alpha_{qs}$, a $G\alpha_q$ protein with the last five amino acids at the C terminus replaced by the corresponding amino acids of $G\alpha_s$ (gifted from The J. David Gladstone Institutes, San Francisco, CA) in a ratio of 1:3 with the Mirus TransIT-293 reagent in serum-free medium in a 1:3 ratio. After equal distribution of the mixture to the cells incubation was continued for 48 h before performing the assay. For BRET experiments, HEK cells were detached, diluted to a cell concentration of 250,000 cells/mL and transfected with 100 ng cDNA of the β_2AR wt for β-arrestin recruitment (receptor:β-arrestin:GRK2:CAAX ratio 1:0.2:1:3) using linear polyethyleneimine (PEI, Polysciences, 3:1 PEI:DNA ratio). The DNA was complemented to a total amount of 1 µg DNA per $3 \cdot 10^5$ cells with ssDNA (Sigma Aldrich) and 20,000 cells per well were transferred into 96-well half-area plates (Greiner, Frickenhausen, Germany). Cells were incubated for 48 h before starting the assay.

Functional assays

Irradiation of photoswitches

To adjust the photoswitchable compounds in the desired *E*- or *Z*-isoform a 100 µM stock solution was irradiated with high-power LEDs emitting light at a wavelength of 365 nm (for 30 s to adjust the *Z*-isomer) or 528 nm (for 120 s to switch to *E*-isomer) directly before starting the assay.

IP-One accumulation assay

The evaluation of receptor activity of the β_2AR wild-type and the mutant was performed applying the IP-One HTRF assay (PerkinElmer, Rodgau, Germany). The experiments were conducted according to the manufacturer's protocol. In brief, HEK-293T cells were transiently co-transfected with appropriate cDNA and seeded into 384 well plates (Greiner Bio-One, Frickenhausen, Germany) and incubated for 24 h. For the assay test compounds in the range from 10 pM to 1 mM were added to the cells and incubated for 180 min followed by addition of IP1-d2 conjugate and 5 µL anti-IP1-cryptate-TB conjugate reagent to stop incubation and starting lysis. After further 60 min time resolved fluorescence

resonance energy transfer (HTRF) was determined using the CLARIOstar® plate reader (BMG, Ortenberg, Germany) (Filter set: donor: 620-10 nm; acceptor: 665-10 nm). Data was analyzed by calculating the ratio of acceptor emission to donor emission. FRET ratio was normalized to the effect of buffer (0%) and the maximum effect of norepinephrine (100%) using GraphPad Prizm 10.2 applying the equation for non-linear regression (dose-response curves (four parameters)). Mean values are the result of three to five independent experiments each done in duplicates.

Bioluminescence Resonance Energy Transfer (BRET) assay for recruitment of β–arrestin

For BRET assays microplates were coated with poly-*D*-Lysin (Sigma-Aldrich, Taufkirchen, Germany; MW: 70,000-150,000, 0.1 mg/mL aqueous solution) to improve cell adhesion. HEK-293T cells were transiently transfected applying the PEI reagent as described above. Determination of receptor mediated β-arrestin recruitment was performed by addition of 1 µM of test compound to the cells followed by incubation for 90 min. Preincubation was terminated by removing the incubation solution followed by a washing step with HBSS buffer. Buffer containing 10 µM propranolol was added to block all free binding sites. One half of the wells were kept in the dark, the second half was irradiated at 528 nm for 120 s to enable the switching of the *Z*-isomer to the *E*-isomer. Incubation was continued for 10 min and 3 µM of coelenterazine 400a was added for further 5 min. All incubation steps were performed at 37 °C. In parallel, control experiments were conducted with 1 mM of norepinephrine or buffer to derive reference values for normalization. BRET was monitored on a Clariostar plate reader (BMG, Ortenberg, Germany) with the appropriate filter sets (donor 410/80 nm, acceptor 515/30 nm) and was calculated as the ratio of acceptor emission to donor emission. Switching of the *E*-isomer to the *Z*-isomer after preincubation was done by irradiating the cells at 365 nm for 30 s. BRET ratio was normalized to the effect of buffer (0%) and the maximum effect of norepinephrine (100%). Mean E_{max} values are the result of 3-12 independent experiments each done in quadruplicates. Significance of E_{max} after switching the test compound from the *Z*- to the *E*-isomer and from *E* to *Z* was analyzed by paired t-test in Prism 10.2. The threshold for significance was set as 99% confidence interval and displayed as p-value ($p<0.01$).

2.1. Biological Characterization

2.1.1. IP-One Assay on Functional Activity of the Photoswitches 1-9 at the β_2-adrenoceptor

Functional activity of the photoswitches **1**-**9** were determined in the *E*- and *Z*-isomer applying an IP accumulation assay.[1] HEK 293T cells were transiently co-transfected with the plasmids of the human β_2 receptor and the hybrid G protein $G\alpha_{qs}$. Dose-response curves revealed intrinsic activity for all compounds in the in the range from 70-108 % (Figure 4, Table S1). The BI-167107 derivatives **3-6, 8,** and **9** showed potencies in the range of EC_{50} = 7.2 nM (*Z*-**3**) to EC_{50} = 79 nM (*E*-**5**), while for the catechol

derivatives **1, 2, 7** dose-response correlations resulted in potencies of EC_{50} = 480 nM (*Z*-**1**) to EC_{50} = 4,300 µM (*Z*-**7**).

Table S1. Functional properties of the photoswitches **1-9** at the wild type receptor β_2AR.

compound	*E*-isomer			*Z*-isomer		
	EC_{50} [nM ± S.E.M.][a]	E_{max} [% ± S.E.M.][b]	n[c]	EC_{50} [nM ± S.E.M.][a]	E_{max} [% ± S.E.M.][b]	n[c]
**1**	630 ± 72	108 ± 3	4	480 ± 110	78 ± 1	4
**2**	940 ± 34	96 ± 2	5	700 ± 35	97 ± 1	5
**3**	7.2 ± 3.0	85 ± 2	4	17 ± 6.3	78 ± 4	4
**4**	39 ± 7.0	88 ± 3	5	9.8 ± 0.9	87 ± 5	5
**5**	79 ± 25	98 ± 1	3	9.9 ± 1.7	93 ± 4	3
**6**	38 ± 5.8	70 ± 2	4	21 ± 1.3	89 ± 1	4
**7**	1,500 ± 78	103 ± 4	3	4,300 ± 1,300	101 ± 10	3
**8**	53 ± 2.6	84 ± 5	4	75 ± 2.3	90 ± 2	3
**9**	72 ± 12	88 ± 5	3	57 ± 8.5	85 ± 5	3

[a] Potencies for receptor activation as mean values in [nM ± S.E.M.]. [b] Maximum efficacy determined relative to the full maximum effect of norepinephrine. [c] Number of individual experiments each done in duplicates.

To evaluate the activation properties of the β_2AR mutant β_2K97$^{2.68}$C compared to the wild-type receptor an IP accumulation assay was performed applying the reference agonists norepinephrine and epinephrine. Norepinephrine showed an EC_{50} of 3200 nM for the mutant. This was in the same range as an EC_{50} of 1100 nM, which was determined for the wild-type receptor. Similar correlations could be observed for epinephrine revealing an EC_{50} = 240 nM and an E_{max} = 109% for the mutant and an EC_{50} = 190 nM and an E_{max} = 103% for the wild-type β_2AR (Supplementary Figure S38, Table S2).

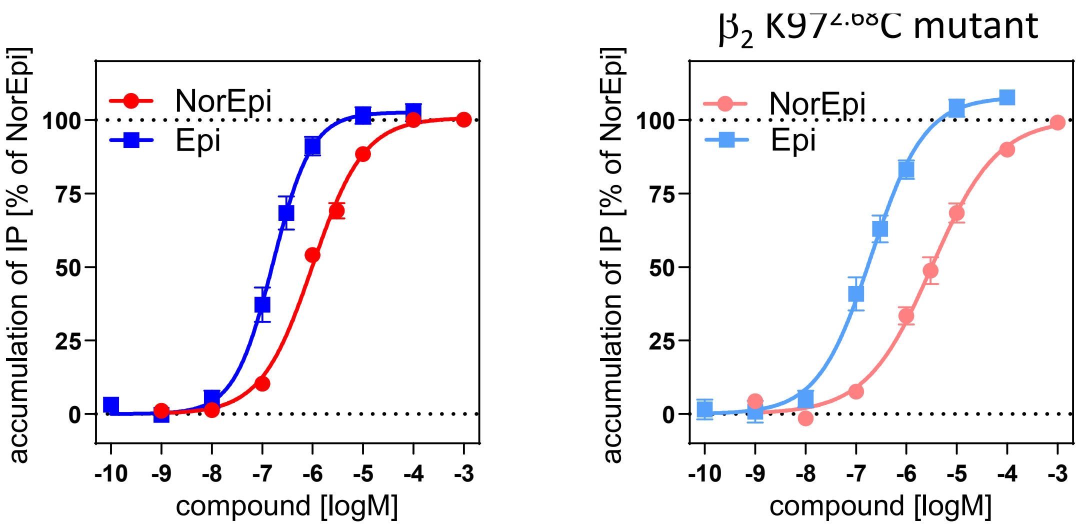

Figure S38. Evaluation of receptor activation for norepinephrine and epinephrine at the β_2 wt and the mutant β_2 K97$^{2.68}$C. An IP accumulation assay in HEK293T cells transiently transfected with β_2 wt or β_2 K97$^{2.68}$C and the hybrid G-protein Gα_{qs} was conducted to get dose-response curves as mean from 10-14 individual experiments with β_2 wt and 5-6 single experiments with the mutant β_2 K97$^{2.68}$C.

Table S2. Functional properties of the reference compounds norepinephrine and epinephrine at the wild-type and the mutant β_2AR.

compound	β_2 AR wild-type			β_2 AR K97$^{2.68}$C		
	EC$_{50}$ [nM ± SEM][a]	E$_{max}$ [%][b]	n	EC$_{50}$ [nM ± SEM][c]	E$_{max}$ [%][b]	n
norepinephrine	1,100 ± 109	100	14	3,200 ± 520	100	10
epinephrine	190 ± 34	103 ± 2	5	240 ± 58	109 ± 3	6

[a] Potencies derived from 10 to 14 independent experiments each done in duplicates. [b] Maximum efficacy determined relative to the reference norepinephrine. [c] Potencies derived from 5 to 6 independent experiments each done in duplicates.

2.1.2. Determination of Receptor Activation after Covalent Binding and Photoswitching Applying an Arrestin Recruitment Assay

Covalent binding to the receptor and subsequent switching of the bound isomer within the orthosteric ligand-binding site was determined with a bioluminescence resonance energy transfer (BRET) based biosensor system applying the enhanced bystander biosensor CAAX and the β_2AR mutant K97$^{2.68}$C in HEK293T cells.[2] To facilitate covalent binding, 1 µM of the *Z*-isomers of **1-9** was preincubated with cells before washing and blocking any free receptor binding site. To measure the effect of the formed *E*-isomer cells were irradiated with light at 528 nm. Determination of the resulting arrestin recruitment showed activation profiles with E$_{max}$ values in the range of 2-52% relative to norepinephrine indicating a certain amount of irreversible occupation and subsequent activation of the receptor (Supplementary Figure S39). The catechol derivatives **1**, **2**, and **7** showed only moderate E$_{max}$ values (2-19%). Greatest receptor activation was observed for the four-carbon linker-substituted disulfide **5** (E$_{max}$ = 52%) and its five-carbon analog **6** (E$_{max}$ = 51%). Comparison of the *Z*-isomers and their corresponding *E*-isomers, which have been formed after irradiation, yielded in compound **6** showing intrinsic effects with the greatest difference in E$_{max}$ between *Z* and *E* (51% for *Z*-**6** and 38% for the *E*-isomer).

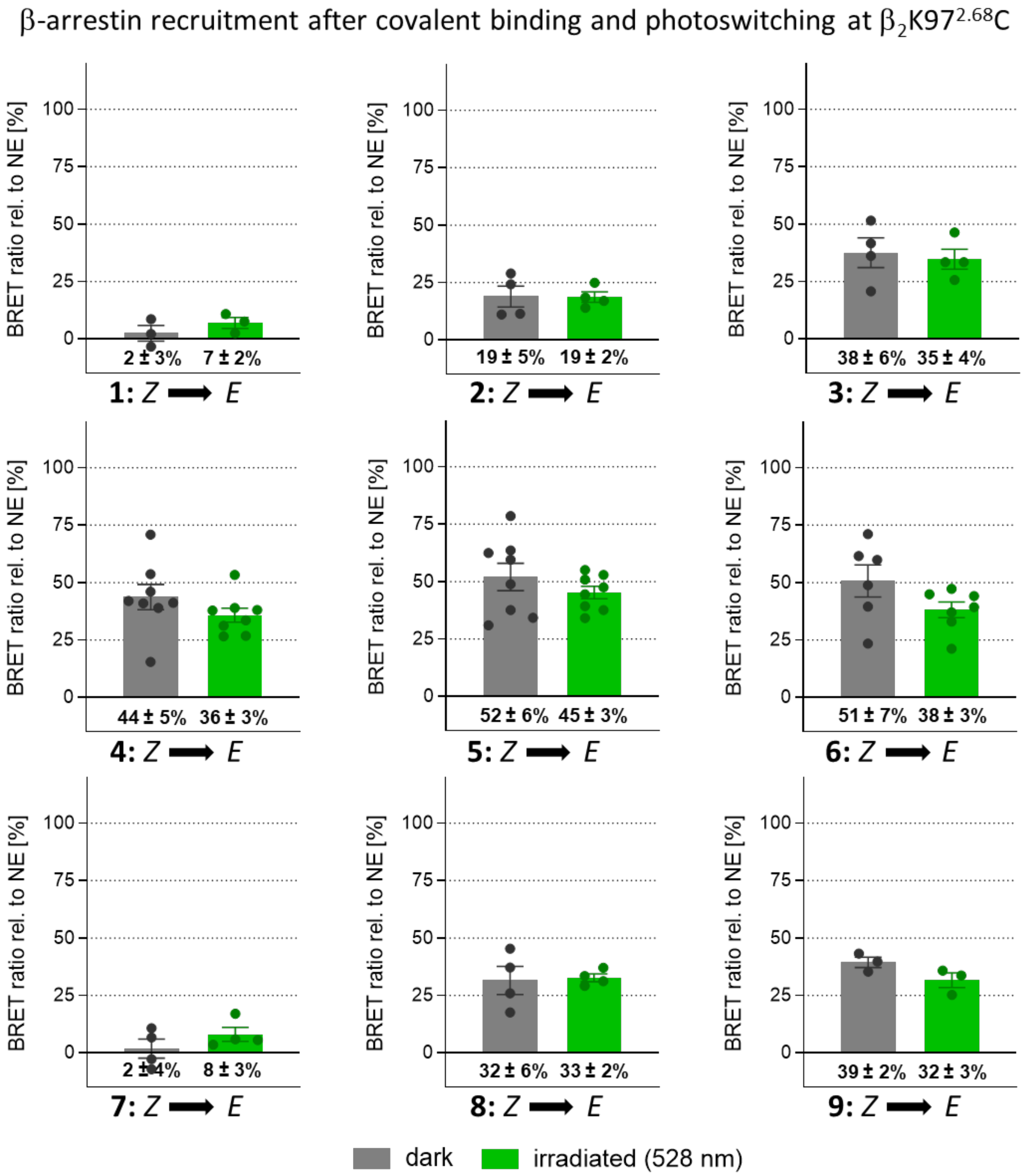

Figure S39. Intrinsic activity for the test compounds after covalent binding at the β_2AR K97$^{2.68}$C mutant and irradiation with light at 528 nm. A β-arrestin recruitment assay based on the BRET enhanced bystander biosensor CAAX and the β_2AR mutant K97$^{2.68}$C in HEK293T cells was performed for preincubation with 1 µM of the *Z*-isomers of **1-9**. After washing and blocking of any free receptor binding site arrestin recruitment was measured after 15 min to get the maximal efficacy for each *Z*-isomer (gray bars). Irradiation of cells after washing with light at 528 nm and subsequent incubation revealed maximum intrinsic activity for the corresponding *E*-isomer (green bars). Data represent mean values ± SEM and individual data points and are derived from 3-8 independent experiments each conducted in quadruplicates.

3. Computational Methods

3.1. Molecular docking

Different combinations of alkyl spacer lengths (n=1, 2, 3; m=1, 2, 3) and covalent warheads (maleimides, disulfides) were generated *in silico*. For each ligand, both *E*- and *Z*-isomers were docked. Ligands were prepared for covalent docking as described in the User Guide of the GOLD Suite version 5.2. Briefly, for all ligand combinations using disulfides as covalent warheads, the terminal aminoethyldisulfide group (R-S-S-C_2H_4-NH_2) was replaced with a hydrodisulfide group (R-S-S-H), with the terminal sulfur atom designated as the link atom for covalent docking. For all ligand combinations using maleimides as covalent warheads, the terminal maleimide was substituted with a pyrrolidine-2,5-dione ring, modified with either a primary amino group or a thiol group attached to the carbon at position 3. In these formal Michael adducts, the nitrogen of the primary amino group or the sulfur atom of the thiol group was selected as the link atom for covalent docking to lysine or cysteine residues, respectively. As the Michael addition to the maleimide derivatives creates a new stereocenter at the carbon in position 3 of the pyrrolidine-2,5-dione ring, both *R*- and *S*-stereoisomers were considered. The X-ray crystal structure of β_2AR bound to epinephrine (PDB: 4LDO)[3] was used for molecular docking. For covalent linkage, the Nε atoms of the lysines K97$^{2.64}$ and K305$^{7.32}$ (in the wild-type β_2AR) were selected for maleimides. For both maleimides and disulfides, the Sγ atoms of the β_2AR single-point cysteine mutants H93$^{2.64}$C, K97$^{2.68}$C and K305$^{7.32}$C were used. These cysteine mutants were generated using UCSF Chimera version 1.13[4] selecting the most probable cysteine rotamer based on the Dunbrack rotamer library[5]. A total of 234 covalent docking runs were conducted. For each run, ten poses were generated as output and inspected manually.

3.2. Molecular dynamics simulations

Simulation systems for the disengagement of the covalently bound *Z*-**6** and its thiol derivative from the β_2AR orthosteric ligand-binding pocket were built based on the high-resolution crystal structure of β_2AR in complex with the irreversible agonist FAUC50 (PDB: 3PDS)[6], considering the chemical similarity between **6** and FAUC50. To prepare the coordinates, all atoms except those of the receptor were removed. A sodium ion was introduced into its known allosteric binding site[7] by superimposing the adenosine A_{2A} receptor (A2AR) structure (PDB: 6LPK)[8], which includes a resolved sodium ion, onto our model and extracting the sodium ion coordinates. Specific mutations (H93$^{2.64}$C, N187E) were reverted to the human wild-type, while a K97$^{2.68}$C mutation was introduced to enable covalent docking at this position. The missing segment of the unresolved and flexible intracellular loop 3 (ICL3) corresponding to the sequence GSGSGS was modelled with MODELLER version 9.21[9]. The Protein

Preparation Wizard module of the Maestro suite (Schrödinger Release 2022-1[10]) was employed to optimize hydrogen bonding and model amino acids in their predominant protonation state at pH 7.4, with the exception of E122$^{3.41}$, which was protonated due to its close proximity to the lipid tails.[11] The covalently-bound *Z*-**6**-β_2AR complex was created via covalent docking with CovDock version 1.3.[12] First, the terminal aminoethyldisulfide (R-SS-CH_2H_4-NH_2) of *Z*-**6** was replaced with a thiol group (R-SH) to create the thiol derivative of *Z*-**6**. Ligand preparation for docking involved LigPrep to generate a low-energy structure while preserving chiral centers. The grid box for docking was centered at the centroid of D113$^{3.32}$, with an inner box of 10 Å × 10 Å × 10 Å and an outer box of 50 Å × 50 Å × 50 Å. The mutated residue C97$^{2.68}$ was chosen as the reactive site for disulfide formation. To obtain the coordinates of the thiol analog of *Z*-**6** bound to the β_2AR, the disulfide bond in the covalently-bound *Z*-**6**-β_2AR complex was removed, and hydrogen atoms were added.

Parameter topology and coordinate files for the simulated systems were generated using the LEAP module of AMBER22.[13] The ligand-bound receptors were first energy minimized using the PMEMD module of AMBER22, employing 500 steps of the steepest descent method followed by 4500 steps of the conjugated gradient method.[14] After conversion to GROMACS input files using ParmEd,[15] the receptor systems were aligned to the Orientations of Proteins in Membranes (OPM)[16] structure of β_2AR in complex with carazolol (PDB: 5D5A)[17]. Each complex was then embedded into a solvated and pre-equilibrated dioleoyl-phosphatidylcholine (DOPC) lipid membrane using the GROMACS tool g_membed.[18] Water molecules were replaced with sodium and chloride ions to maintain a neutral and physiological system at a 0.15 M NaCl concentration. The final simulation system dimensions were approximately 80 Å × 80 Å × 150 Å, containing around 96,600 atoms, including 156 DOPC molecules, roughly 23,400 waters, 87 sodium ions, and 92 chloride ions. In all simulations, the general AMBER force field (GAFF2)[19] was used for ligands, Lipid14[20] for DOPC, ff14SB[21] for protein residues, and the SPC/E water model[22] for water molecules. Ligand parameters were assigned using antechamber.[23] The structures of all ligands were optimized using Gaussian 16[24] with the B3LYP functional and the 6-31G(d) basis set, and charges were calculated using the Hartree-Fock (HF) functional with the same basis set. Atom point charges were assigned according to the RESP procedure.[25] A formal charge of +1 was defined for all ligands. Dihedral restraints of 133.5 kcal·mol^{-1}·rad^{-2} were applied to maintain the ligands as *Z*-isomers. Isomerization from *Z* to *E* was achieved by inverting the restraints.

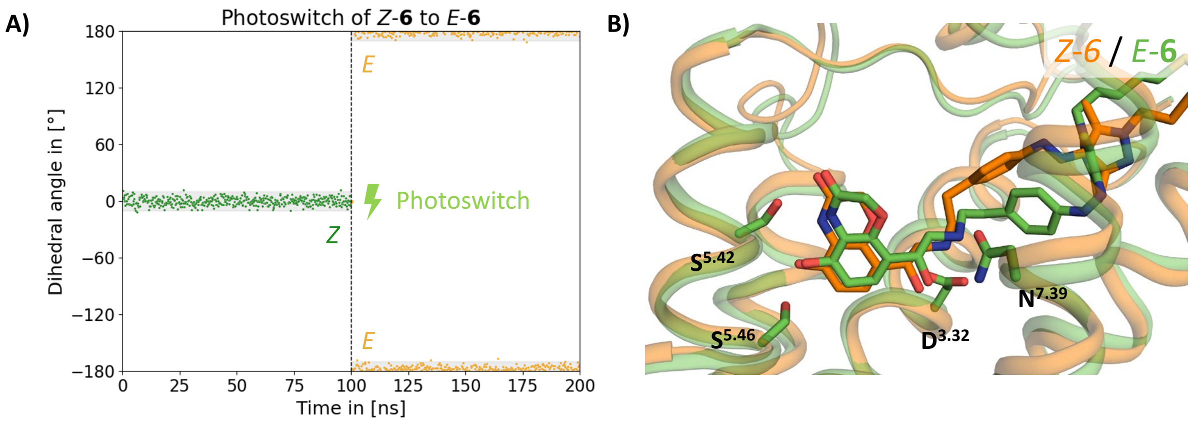

Figure S40. During the MD simulation, dihedral restraints were applied to the azo bond to maintain the *Z*-isomer configuration. To simulate photoswitching, the dihedral restraint was rapidly inverted, resulting in isomerization to the *E*-isomer. Photoswitching can occur within the binding pocket. **A)** Scatter plot showing the dihedral angle of the azo bond before and after photoswitching during the MD simulation. **B)** Representative binding poses of covalently bound *Z*-**6** and *E*-**6** within the orthosteric binding pocket.

Simulations were conducted using GROMACS 2022.5.[26] Each system underwent energy minimization and was equilibrated in the constant-temperature, constant-volume (NVT) ensemble, followed by the constant-temperature, constant-pressure (NPT) ensemble for 1 ns with harmonic restraints of 10.0 kcal·mol^{-1}·Å^{-2} on the protein and ligand. Subsequently, the system was equilibrated in the NPT ensemble for 25 ns with restraints on protein backbone and ligand atoms, which were progressively reduced every 5 ns from 10.0 to 0.1 kcal·mol^{-1}·Å^{-2}. Temperature control was achieved using the velocity-rescale thermostat[27] (time constant: 1.0 ps, reference temperature: 310 K), and pressure control was implemented with the stochastic cell rescaling barostat[28] (semi-isotropic; time constant: 5.0 ps; reference pressure: 1 bar; compressibility: 4.5 × 10^{-5} bar^{-1}). Bond lengths to hydrogen atoms were constrained using the LINCS[29] algorithm. Periodic boundary conditions were applied, with a 1.0 Å cutoff for Lennard-Jones and short-range electrostatic interactions. Long-range electrostatics were computed using the Particle Mesh Ewald (PME)[30] method with a fourth-order interpolation scheme and fast Fourier transform (FFT) grid spacing of 1.25 Å. Long-range van der Waals interactions were corrected for energy and pressure using a continuum model. The equations of motion were integrated with a time step of 2 fs.

Figures of the structures and models were prepared using PyMOL v2.4[31]. Analyses of the trajectories were performed with Visual Molecular Dynamics (VMD)[32] and CPPTRAJ[13]. Plots were created using Matplotlib 3.8.3[33] and Seaborn 0.13.2[34].

3.3. Metadynamics simulations

Metadynamics simulations were performed to investigate the disengagement of the covalently bound *Z*-**6** and its thiol derivative from the β$_2$AR orthosteric ligand-binding pocket. These simulations were performed using GROMACS 2022.5, patched with the open-source, community-developed PLUMED

library[35] version 2.8,[36] following previously established protocols.[37] Specifically, the approach combined well-tempered metadynamics (WT),[38] funnel metadynamics (FM),[39] and funnel-shaped walls to define path collective variables (PCVs). A history-dependent bias was applied along the z-axis component of the distance between the center of mass of the Cα atoms of V117$^{3.36}$ and W286$^{6.48}$, representing the center of the receptor, and the carbon atom of the phenyl ring connected to the diazo group of the ligand. This distance was used as a single collective variable. Gaussian hills with an initial height of 1.20 kcal·mol^{-1} were applied every 1 ps, with a hill width set to 1 Å. In the WT scheme, the Gaussian functions were rescaled using a bias factor of 30. An initial metadynamics simulation was run for each system until the ligand detached from the receptor. Following this, 32 frames were extracted from the initial simulation to initiate a multiple-walker metadynamics simulation.[40] These frames captured a broad range of ligand conformations, from being inside the β_2AR orthosteric ligand-binding pocket to the extracellular region of the receptor. During the multiple-walker metadynamics simulations, a funnel restraint was applied to the ligand's relative position on the xy plane to improve sampling of the relevant regions. The Gaussian hill height was adjusted to 0.48 kcal·mol^{-1}, and the bias factor was set to 10. For both ligands, the total simulation time reached 5.12 µs.

The generated trajectory data were subsequently analyzed using k-Means clustering [41], the Maximum Caliber (MaxCal)[42] method, and Markov State Models (MSMs)[43] to provide insight into the ligand binding thermodynamics and kinetics.

3.4. Clustering and Markov state modeling analysis

Interaction fingerprints for the ligand in each simulation frame were calculated by quantifying both intramolecular contacts (within the ligand moieties) and intermolecular contacts (between the ligand and the receptor), using the following formula:

$$C_{IJ} = \frac{1 - \left(\frac{\min_{i \in I, j \in J} d_{ij}}{d_0} \right)^n}{1 - \left(\frac{\min_{i \in I, j \in J} d_{ij}}{d_0} \right)^m}$$

where i, j denote atom indices, and I, J are indices for protein residues or ligand fragments. The distance d_0 was set to 0.4 nm, and the exponents n and m were set to 10 and 20, respectively. The ligands were divided into benzoxazinone, benzyl, phenyl, pyrazol, and disulfide fragments, and intramolecular contacts between protein residues were excluded from the analysis. The interaction fingerprints C_{IJ} were decomposed using standard principal component analysis (PCA), and the system's free-energy was recalculated as a function of the first two principal components (p_1, p_2) using

a standard reweighing approach.[44] Conformational microstates S_μ for the ligand dynamics were defined by binning the values of the PCA components as follows:

$$S_\mu = (x_{1,\mu} < p_1 \leq x_{1,\mu} + \delta, x_{2,\mu} < p_2 \leq x_{2,\mu} + \delta)$$

where $\delta = 5\ 10^{-3}$ and $x_{1,\mu}$ and $x_{2,\mu}$ represent the average values of the first and second components, respectively, in bin μ. The unbiased free energies of these microstates were estimated by reweighing the simulation frames and summing the weights assigned to each microstate using the approach described in [44]. The error bars for the free energies were determined by calculating the standard deviation between the free energy values obtained at the end of the simulation and those estimated using 75% of the simulation data. The dynamic averages of changes in the fingerprint principal components along the trajectory, as well as the average number of bin jumps along the trajectory J, were calculated as:

$$R_{x,i} = \frac{1}{T}\sum\nolimits_t w_t \left|x_{i,t} - x_{i,t+dt}\right|$$

The constraint matrices were defined as:

$$R_{\mu\nu}^{(x,i)} = \left|x_{1,\mu} - x_{1,\nu}\right|$$

and the jumps $J_{\mu\nu} = 1 - \delta_{\mu\nu}$, with the bin adjacency matrix $A_{\mu\nu}$ set to 1 if bins μ and ν were nearest or next-nearest neighbors, and 0 otherwise. The transition matrix was calculated using the MaxCal[42] method as described in ref. [45]. Specifically, by defining the operator $\mathcal{D}(x)_\nu = \pi_\nu x_\nu^{-1}$ for any vector x, we solve $\mathcal{D}(Mx) = x$, where:

$$M = A \exp(\lambda_J J_{\mu\nu} + \sum\nolimits_i \lambda_i R_{\mu\nu}^{(x,i)})$$

and compute the transition matrix as:

$$T_{\mu\nu} = \frac{x_\mu x_\nu}{\pi_\mu} M$$

The λ values are Lagrange multipliers that are determined self-consistently to match the calculated averages, such that $J = \sum_{\mu\nu} \pi_\mu T_{\mu\nu} J_{\mu\nu}$ and $R_{x,i} = \sum_{\mu\nu} \pi_\mu T_{\mu\nu} R_{\mu\nu}^{(x,i)}$. Conformational macrostates were obtained by clustering the microstates based on the interaction fingerprints using a k-means algorithm, resulting in 10 clusters. Table S3 reports the free energies of representative cluster states for the two simulated systems , while figure S47 shows the interaction fingerprints for the covalently-bound *Z*-**6** ligand capturing both intramolecular contacts within the ligand moieties and intermolecular contacts

between the ligand and receptor. Mean-first passage times and residence times (Table S4) were then calculated using transition path theory[46] applied to the MaxCal transition matrix. Although MaxCal and similar entropy maximization approaches provide only point estimates, these estimates depend on microstate free energies that are determined with limited precision. To reflect this uncertainty, we report confidence intervals of the calculated mean first passage and dwell times as standard deviations, obtained by propagating the variability in the microstate free energy estimates through the transition matrix and into the calculation of the mean-first passage and dwell times.

Table S3. State free energies (in kT) relative to the most stable state for the conformations identified by k-means clustering of the two simulated systems using metadynamics simulations. Total free energy values for each cluster group are reported relative to the cluster featuring the BI-167107-based pharmacophore interacting with $D^{3.32}$. Only probabilities larger than 0.01% are reported.

System	Cluster_group	Cluster	Free-energy	Probability
Covalently-bound *Z*-**6**	with the BI-167107-based pharmacophore interacting with $D^{3.32}$	#1	0.75±0.01	31.8%±0.3%
		#2	0±0.005	68%±0.3%
		#3	8±1	0.0104%±0.0003%
		total	*0±0.03*	*99.98%±0.01%*
	with the BI-167107-based pharmacophore not interacting with $D^{3.32}$ but still within the TM bundle	#4	8.77±0.03	0.0104%±0.0003%
		#5	9.3±0.06	–
		#6	11.09±0.09	–
		total	*8.6±0.3*	*0.01%±0.01%*
	with the BI-167107-based pharmacophore not forming an interaction with $D^{3.32}$ and outside the TM bundle	#7	12.3±0.3	–
		#8	13±5	–
		total	*12.57±0.01*	–
Non-covalently bound *Z*-**6**	with the BI-167107-based pharmacophore interacting with $D^{3.32}$	#1	22±2	–
		#2	0±0.0009	96.71±0.09

	#3	12.9±0.3	–
	#4	3.38±0.02	3.28±0.09
	total	*0±0.1*	*99.99%±0.01%*
with the BI-167107-based pharmacophore not interacting with $D^{3.32}$ but still within the TM bundle	#5	12.5±0.4	–
	#6	19.1±0.1	–
	#7	19.8±0.1	–
	#8	11.5±0.1	–
	total	*11.23±0.09*	–
with the BI-167107-based pharmacophore not forming an interaction with $D^{3.32}$ and outside the TM bundle	#9	11.9±0.2	–
	total	*12±0.3*	–

**Table S4.** Mean-first passage and dwell times (in µs) along the exit pathways for the two metadynamics-simulated systems, one with covalently bound Z-6 and the other with non-covalently-bound Z-6.	MFPT covalently-bound *Z*-**6** (µs)	MFPT non-covalently-bound *Z*-**6** (µs)
Inside Orthosteric Site-Outside TM bundle	2500±300	510±30
Inside Orthosteric Site-Alternative Site	410±30	79±2
Alternative Site- Outside TM bundle	1400±200	320±30
Outside TM bundle - Inside Orthosteric Site	1500±100	340±10
Outside TM bundle -Alternative Site	30±30	23±4
Alternative Site-Inside Orthosteric Site	890±60	211±7
	Dwell covalently-bound *Z*-**6**	Dwell non-covalently-bound *Z*-**6**
Inside Orthosteric Site	400±30	87±3
Alternative Site	130±20	52±4
Outside TM bundle	180±50	52±5

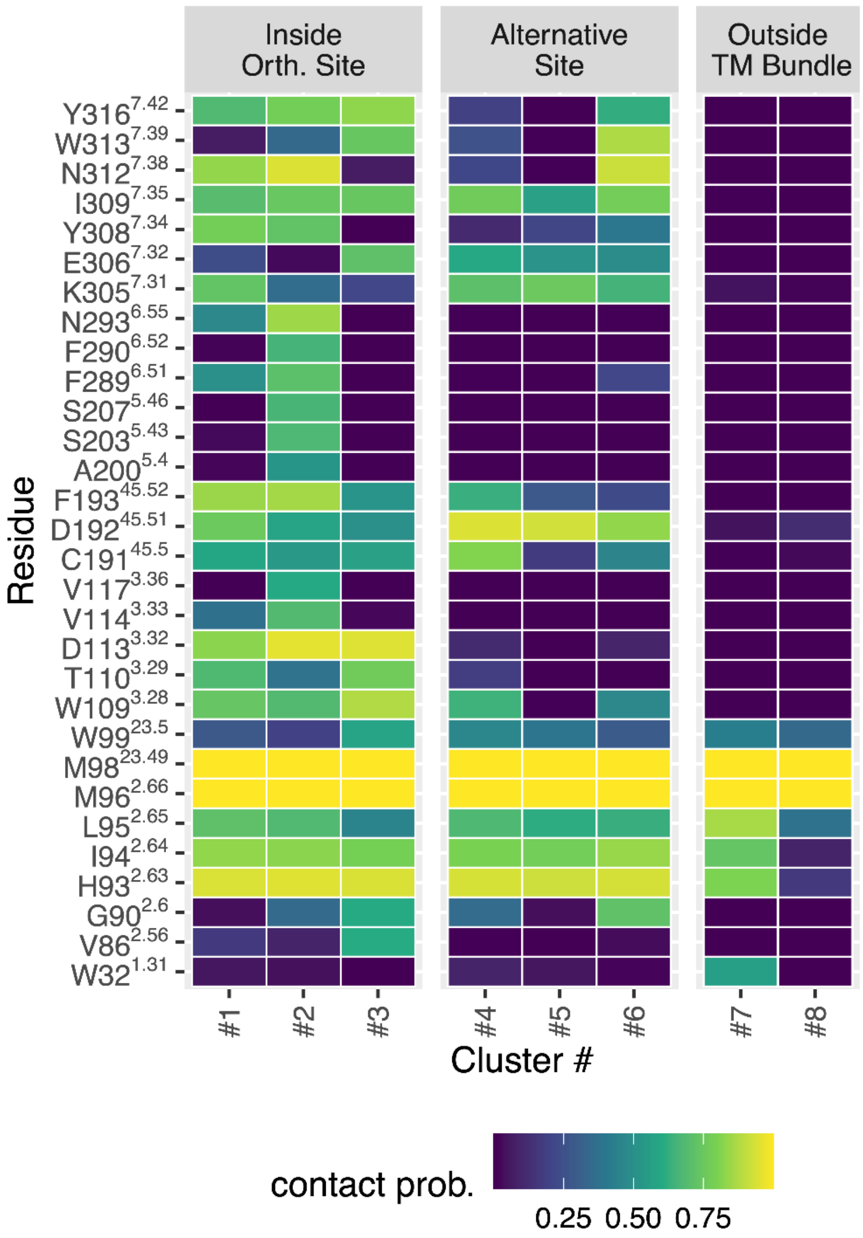

Figure S41. Interaction fingerprints (i.e., intramolecular contacts between the ligand moieties and intermolecular contacts between the ligand and the receptor) for the covalently-bound *Z*-**6** ligand. Clusters #1-#3 contain states with the BI-167107-based pharmacophore located within the β_2AR orthosteric ligand-binding pocket. Clusters #4-#6 are characterized by alternative binding sites along the exit pathway, while Clusters #7 and #8 represent binding sites outside the TM bundle. Interactions between the ligand and the receptor residues are color-coded from blue (low probability) to yellow (high probability).

4. Abbreviations

AcOH	acetic acid
A2AR	A_{2A} adenosine receptor
BI	Boehringer Ingelheim
BnBr	benzyl bromide
BRET	bioluminescence resonance energy transfer
$BTMA\text{-}ICl_2$	benzyltrimethylammonium dichloroiodate
C	cysteine
CB_1	cannabinoid receptor 1
COPD	chronic obstructive pulmonary disease
cryo-EM	cryo-electron microscopy
CXCR3	chemokine receptor CXCR3
DMF	dimethyl formamide
DMP	Dess-Martin periodinane
DMSO	dimethyl sulfoxide
DOPC	dioleoyl-phosphatidylcholine
EC_{50}	half maximal effective concentration
ECL	extracellular loop
EDTA	ethylenediaminetetraacetic acid
EtOH	ethanol
FFT	fast Fourier transform
FRET	fluorescence resonance energy transfer
FM	funnel metadynamics
G	glycine
GPCR	G-protein coupled receptor
GRK2	G protein-coupled receptor kinase 2
H	histidine
His	histidine
HF	Hartree–Fock
HPLC	high performance liquid chromatography
H_3R	histamine H_3 receptor
IP	inositol phosphate
K	lysine
LED	light-emitting diode
Lys	lysine
MaxCal	Maximum Caliber
MD	molecular dynamics
MeCN	acetonitrile
MeOH	methanol
MesCl	mesyl chloride
MetaD	metadynamics
MFPT	mean-first passage times
$mGlu_5$	metabotropic glutamate receptor 5
MS	mass spectrometry
MSM	Markov State Model
MSMs	Markov State Models
NE	norepinephrine

NMR	nuclear magnetic resonance spectroscopy
OPM	Orientations of Proteins in Membranes
PCA	principal component analysis
PCVs	path collective variables
PSS	photostationary state
rt	room temperature
S	serine
$t_{1/2}$	thermal half-life
TFA	trifluoroacetic acid
TLC	thin layer chromatography
TM	transmembrane helix
Tris	tris(hydroxymethyl)aminomethane
VMD	Visual Molecular Dynamics
WT	well-tempered metadynamics
wt	wild type
Y_4R	neuropeptide Y receptor type 4
Zn	zinc
$\alpha_{2A}AR$	α_{2A}-adrenoceptor
β_2AR	β_2-adrenoceptor
µOR	µ-opioid receptor

5. NMR-Spectra

Compound **17**

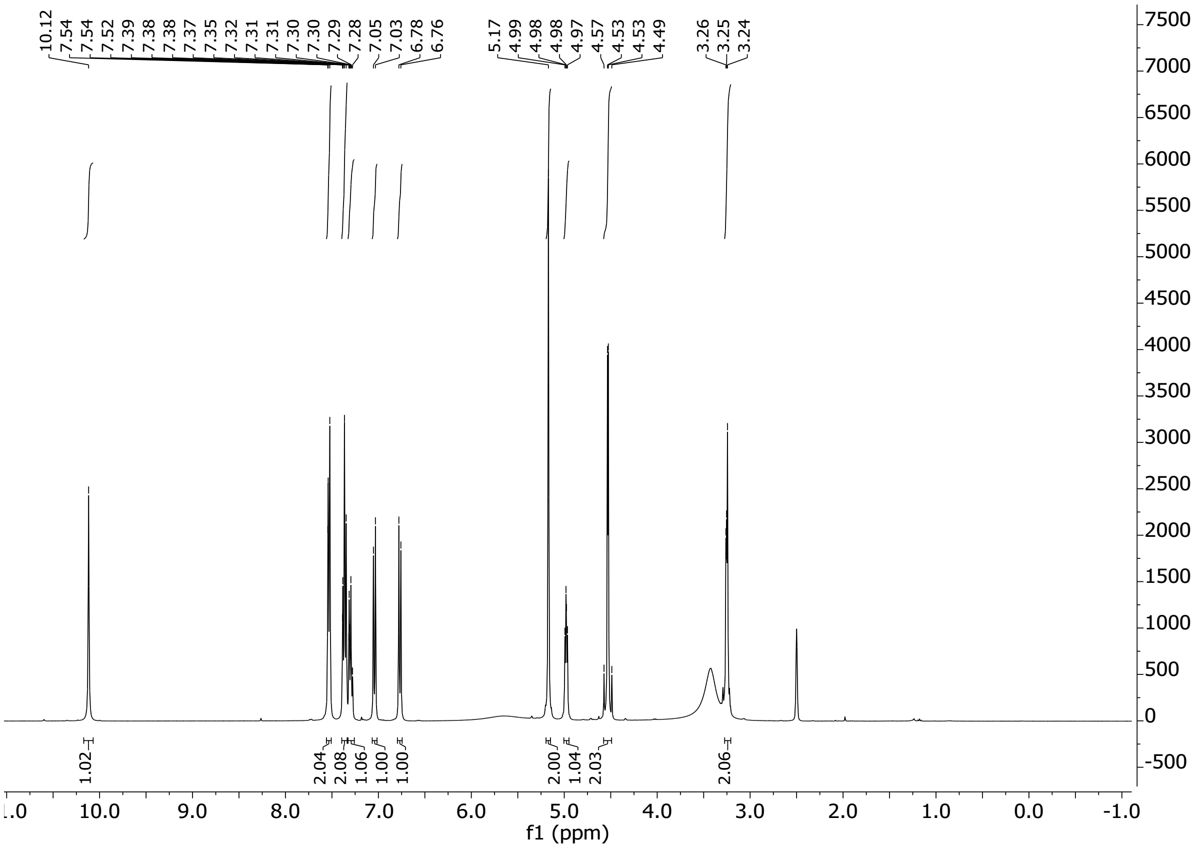

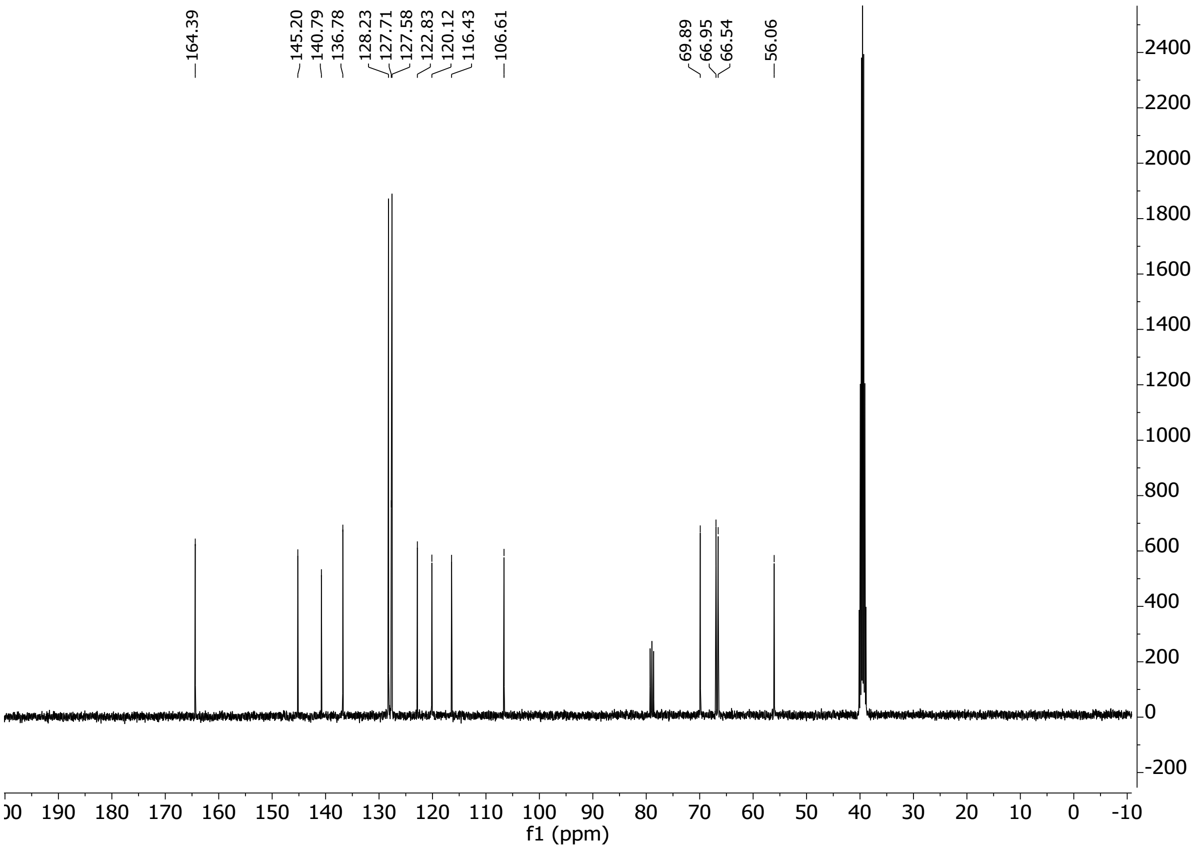

Compound **18**

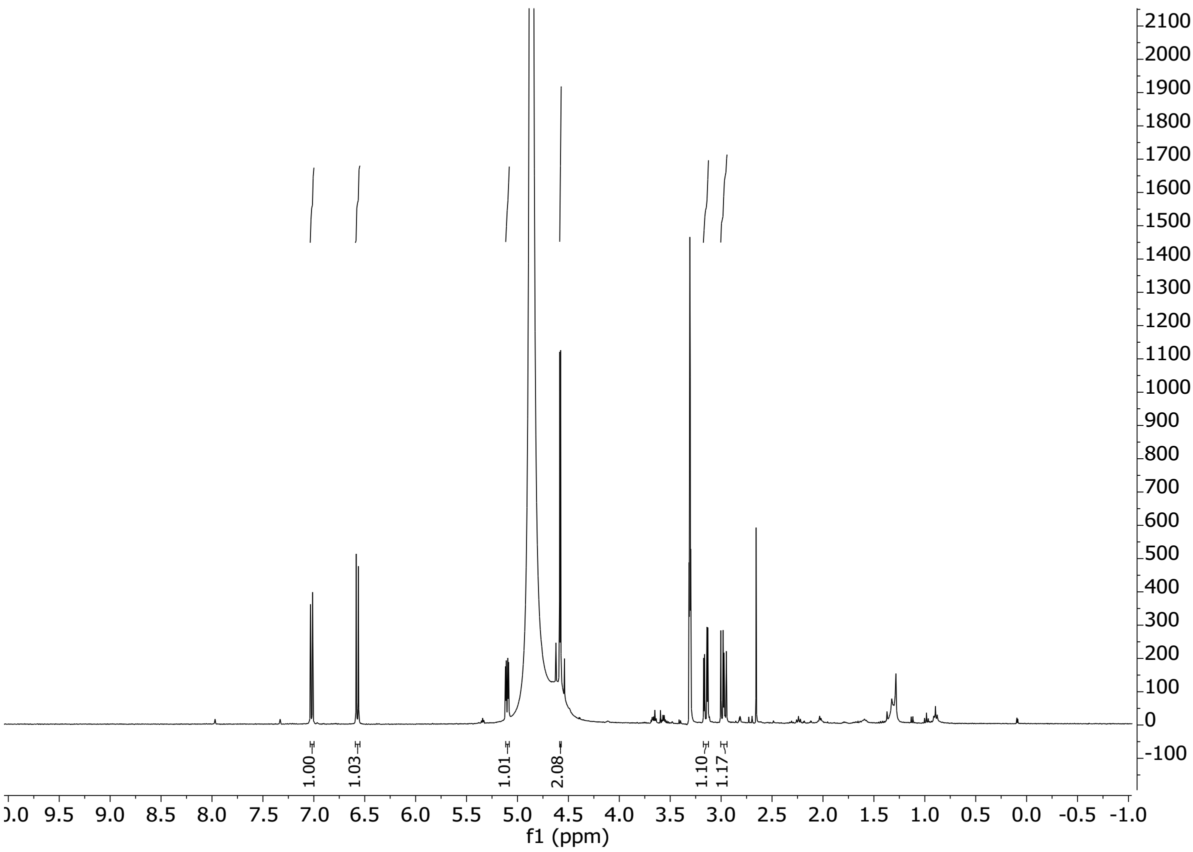
1.00
1.03
1.01
2.08
1.10
1.17
f1 (ppm)

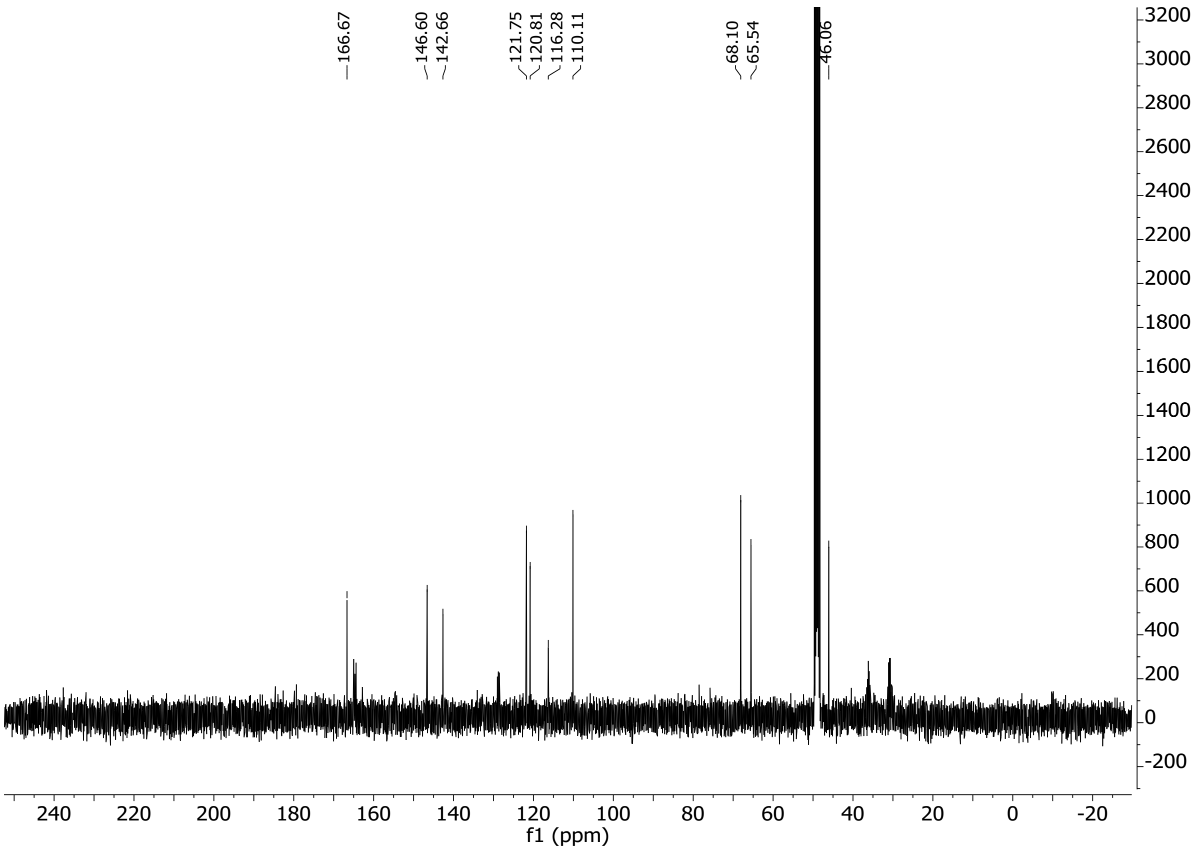
166.67
146.60
142.66
121.75
120.81
116.28
110.11
68.10
65.54
46.06
f1 (ppm)

Compound **20**

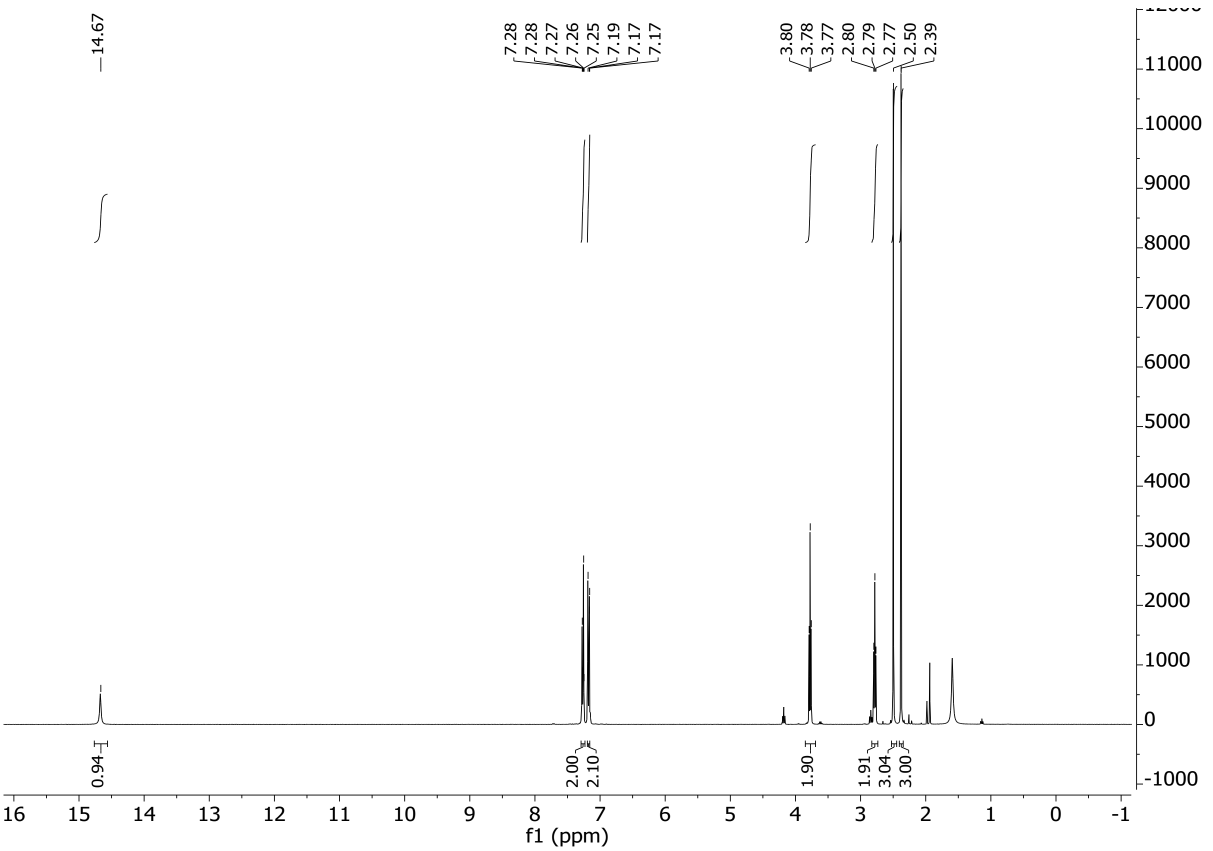

14.67
7.28
7.28
7.27
7.26
7.25
7.19
7.17
7.17
3.80
3.78
3.77
2.80
2.79
2.77
2.50
2.39
0.94
2.00
2.10
1.90
1.91
3.04
3.00
f1 (ppm)

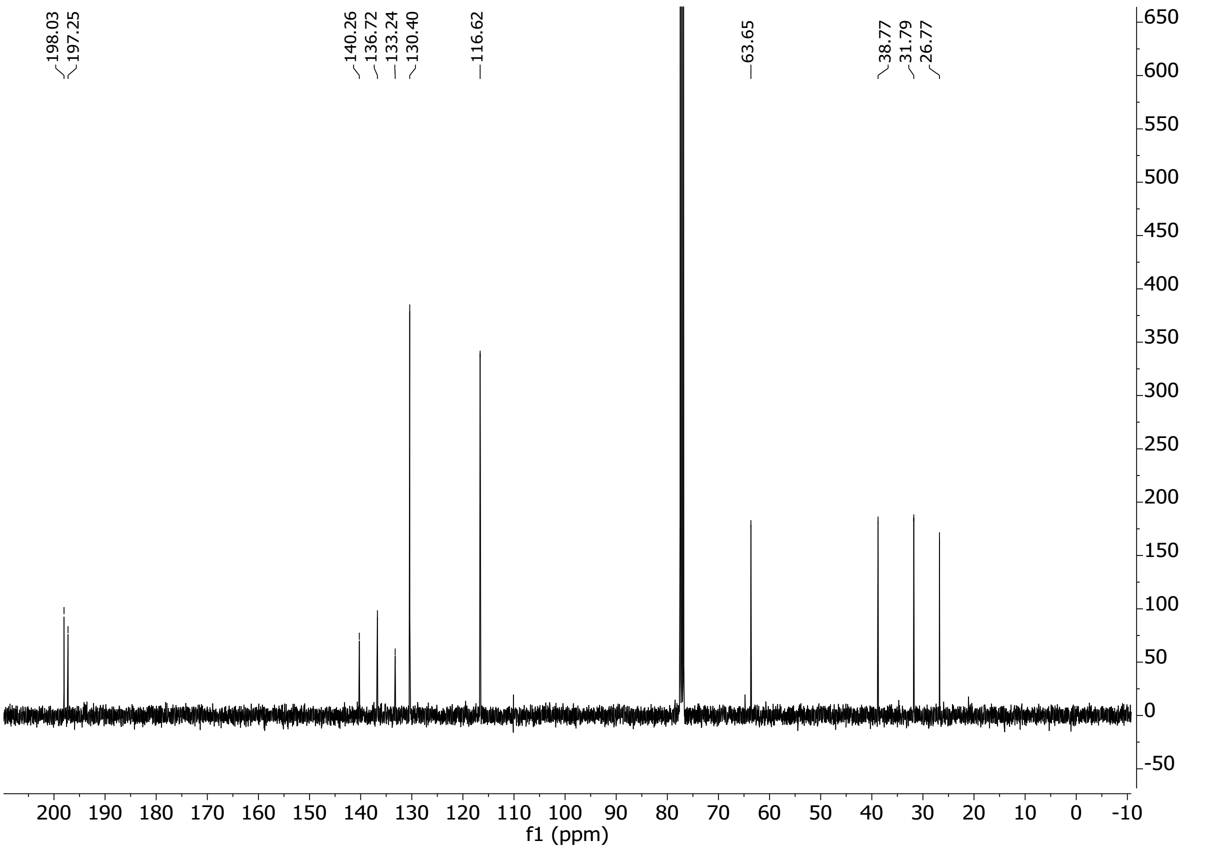

198.03
197.25
140.26
136.72
133.24
130.40
116.62
63.65
38.77
31.79
26.77
f1 (ppm)

Compound **21**

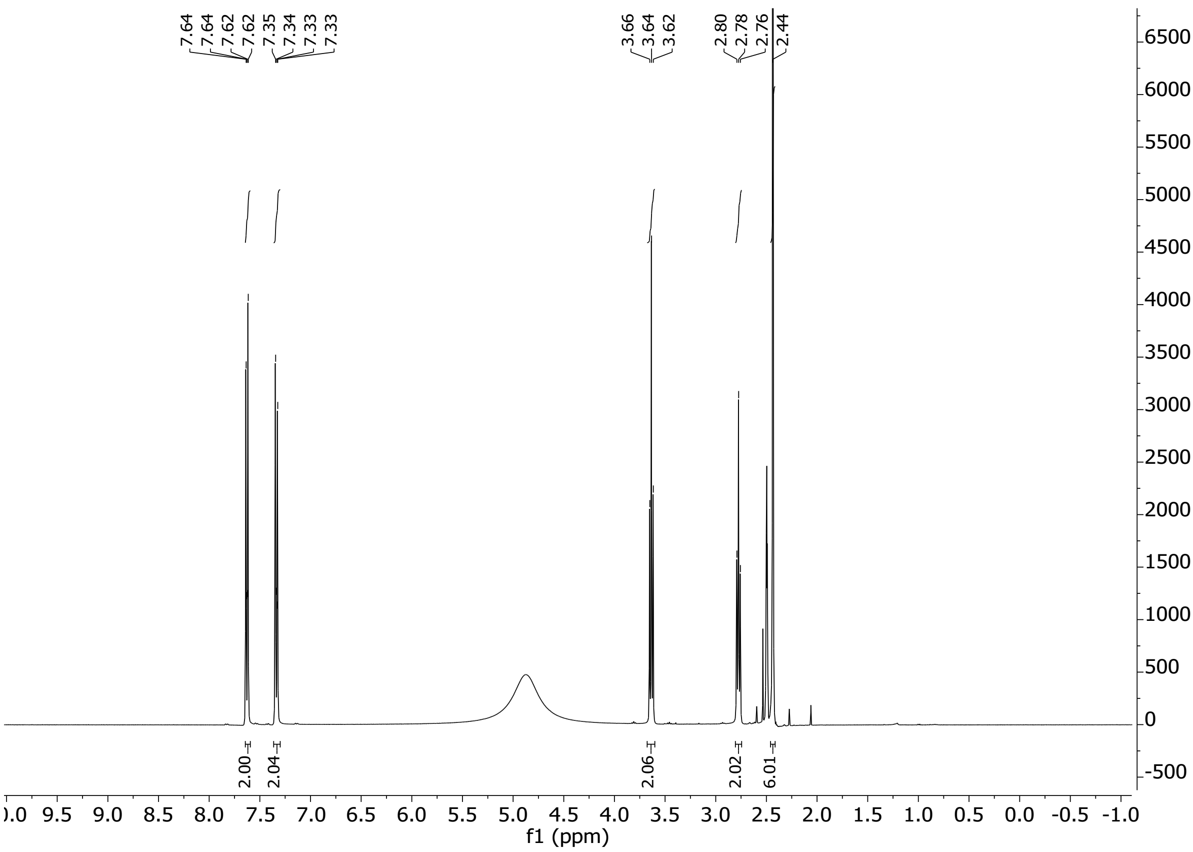

7.64 7.64 7.62 7.62 7.35 7.34 7.33 7.33
3.66 3.64 3.62
2.80 2.78 2.76 2.44
2.00 2.04 2.06 2.02 6.01
f1 (ppm)

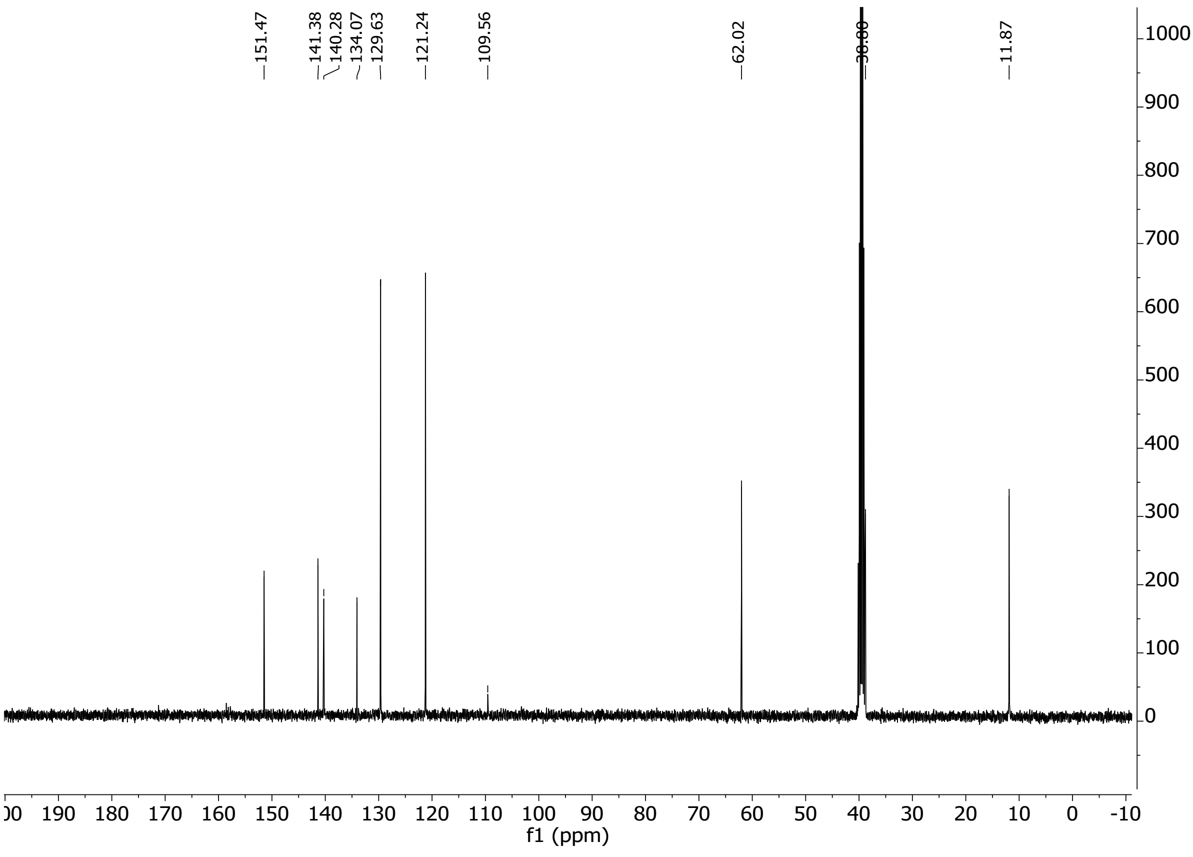

151.47 141.38 140.28 134.07 129.63 121.24 109.56 62.02 11.87
f1 (ppm)

Compound **22**

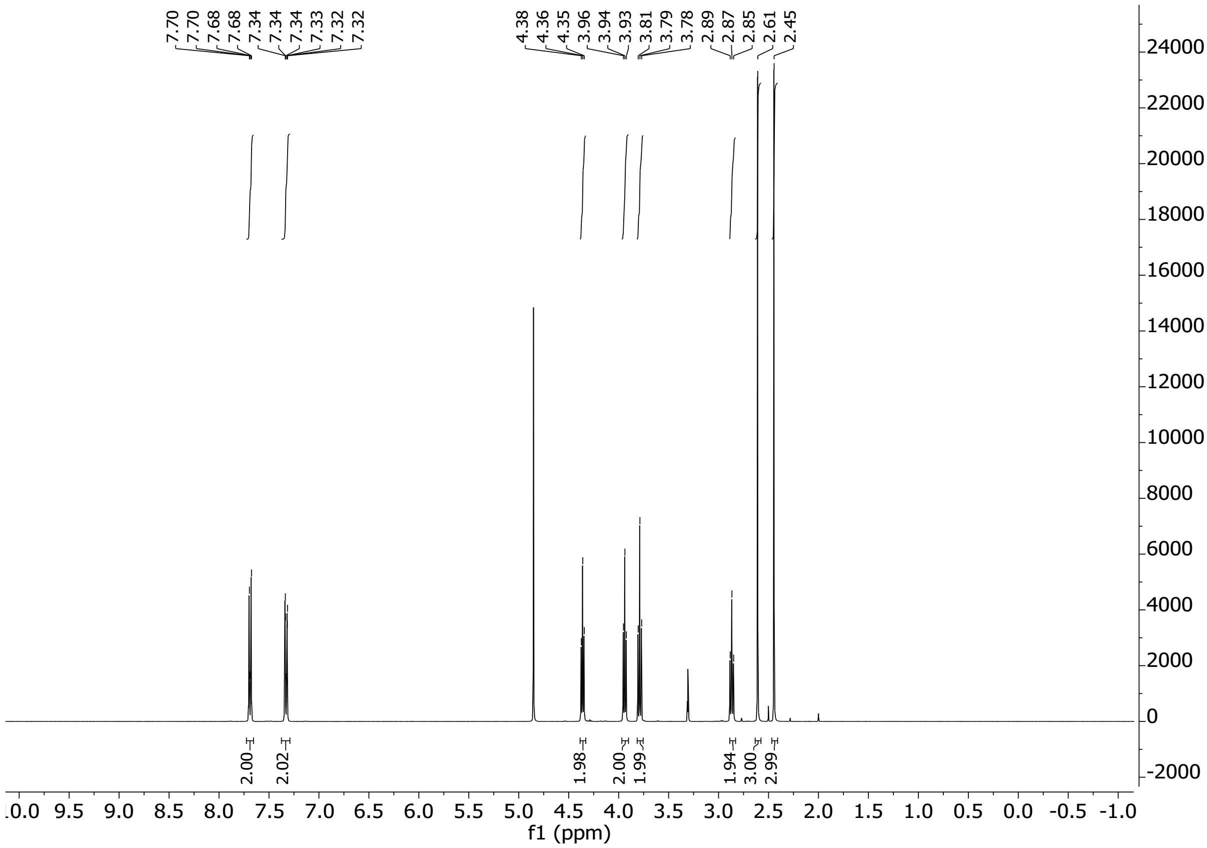

7.70
7.70
7.68
7.68
7.34
7.34
7.34
7.33
7.32
7.32
4.38
4.36
4.35
3.96
3.94
3.93
3.81
3.79
3.78
2.89
2.87
2.85
2.61
2.45
2.00
2.02
1.98
2.00
1.99
1.94
3.00
2.99
f1 (ppm)

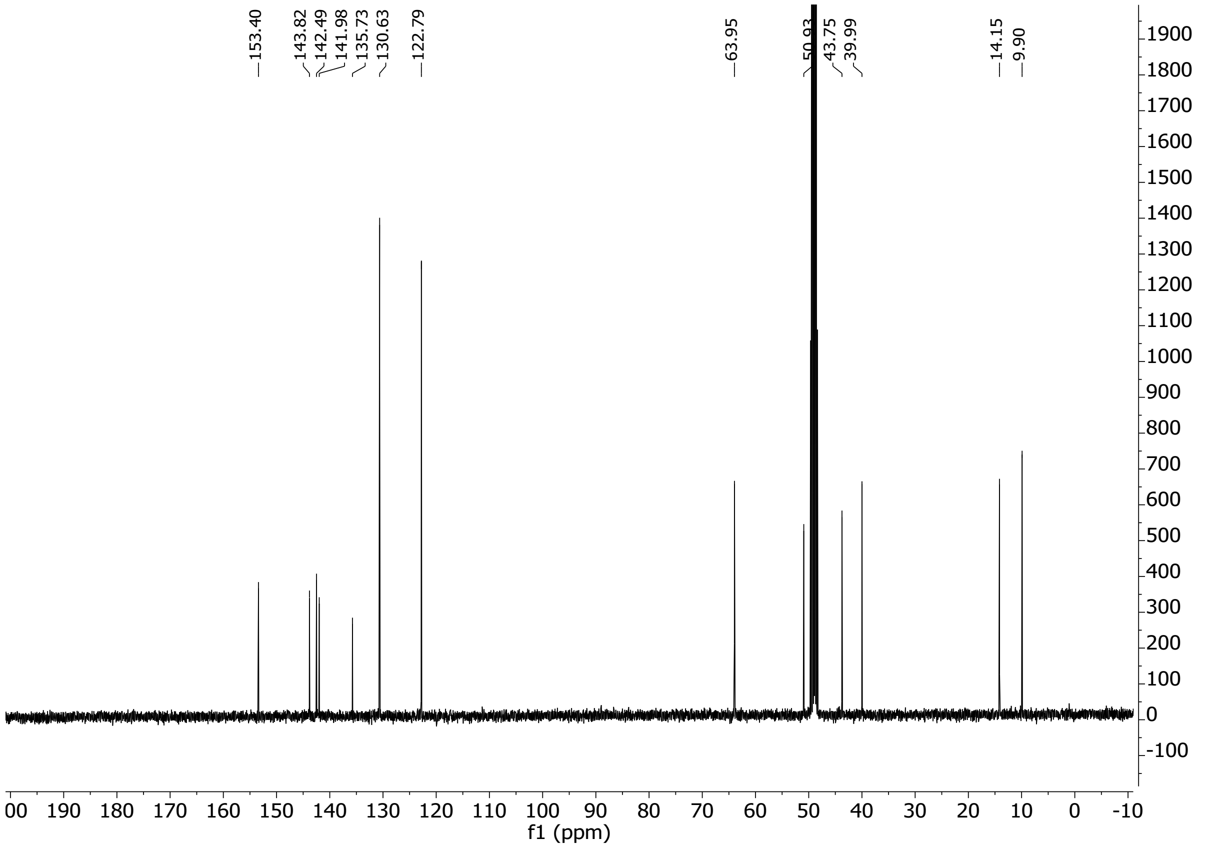

153.40
143.82
142.49
141.98
135.73
130.63
122.79
63.95
50.93
43.75
39.99
14.15
9.90
f1 (ppm)

Compound **23**

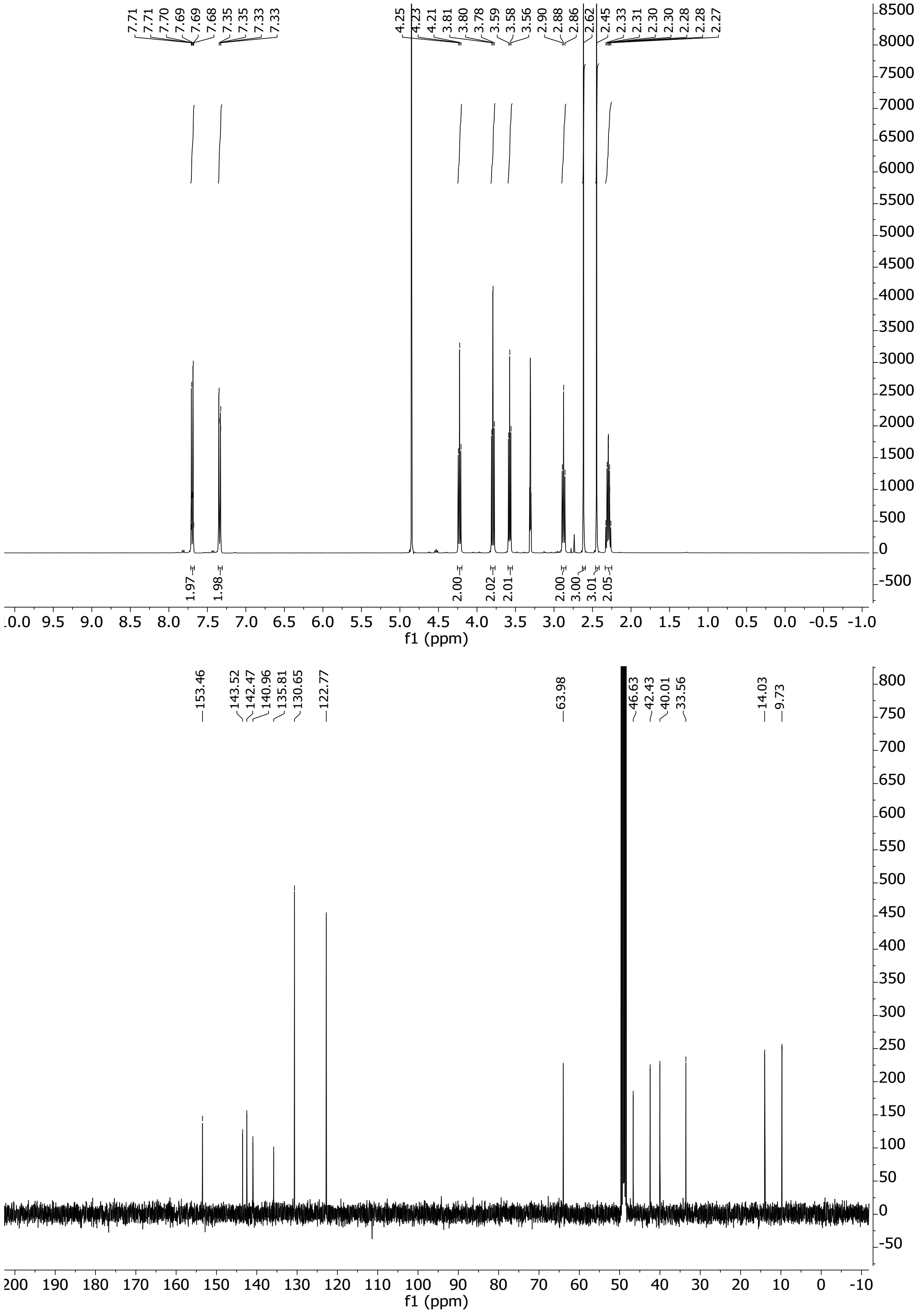
7.71
7.71
7.70
7.69
7.69
7.68
7.35
7.35
7.33
7.33
4.25
4.23
4.21
3.81
3.80
3.78
3.59
3.58
3.56
2.90
2.88
2.86
2.62
2.45
2.33
2.31
2.30
2.30
2.28
2.28
2.27
1.97
1.98
2.00
2.02
2.01
2.00
3.00
3.01
2.05
f1 (ppm)
153.46
143.52
142.47
140.96
135.81
130.65
122.77
63.98
46.63
42.43
40.01
33.56
14.03
9.73
f1 (ppm)

Compound **24**

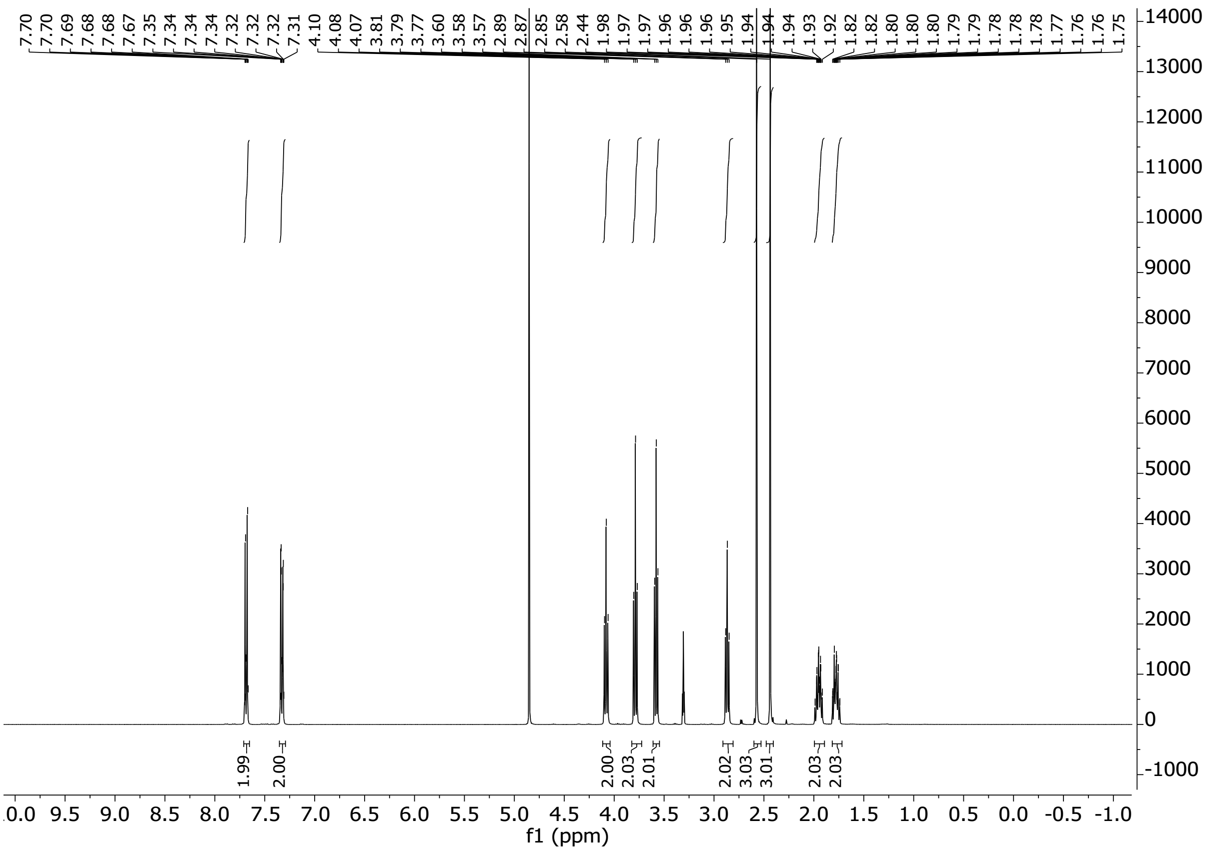

7.70 7.70 7.69 7.68 7.68 7.67 7.35 7.34 7.34 7.34 7.32 7.32 7.32 7.31 4.10 4.08 4.07 3.81 3.79 3.77 3.60 3.58 3.57 2.89 2.87 2.85 2.58 2.44 1.98 1.97 1.97 1.96 1.96 1.96 1.95 1.94 1.94 1.94 1.93 1.92 1.82 1.82 1.80 1.80 1.80 1.79 1.79 1.78 1.78 1.78 1.77 1.76 1.75
1.99
2.00
2.00
2.03
2.01
2.02
3.03
3.01
2.03
2.03
f1 (ppm)

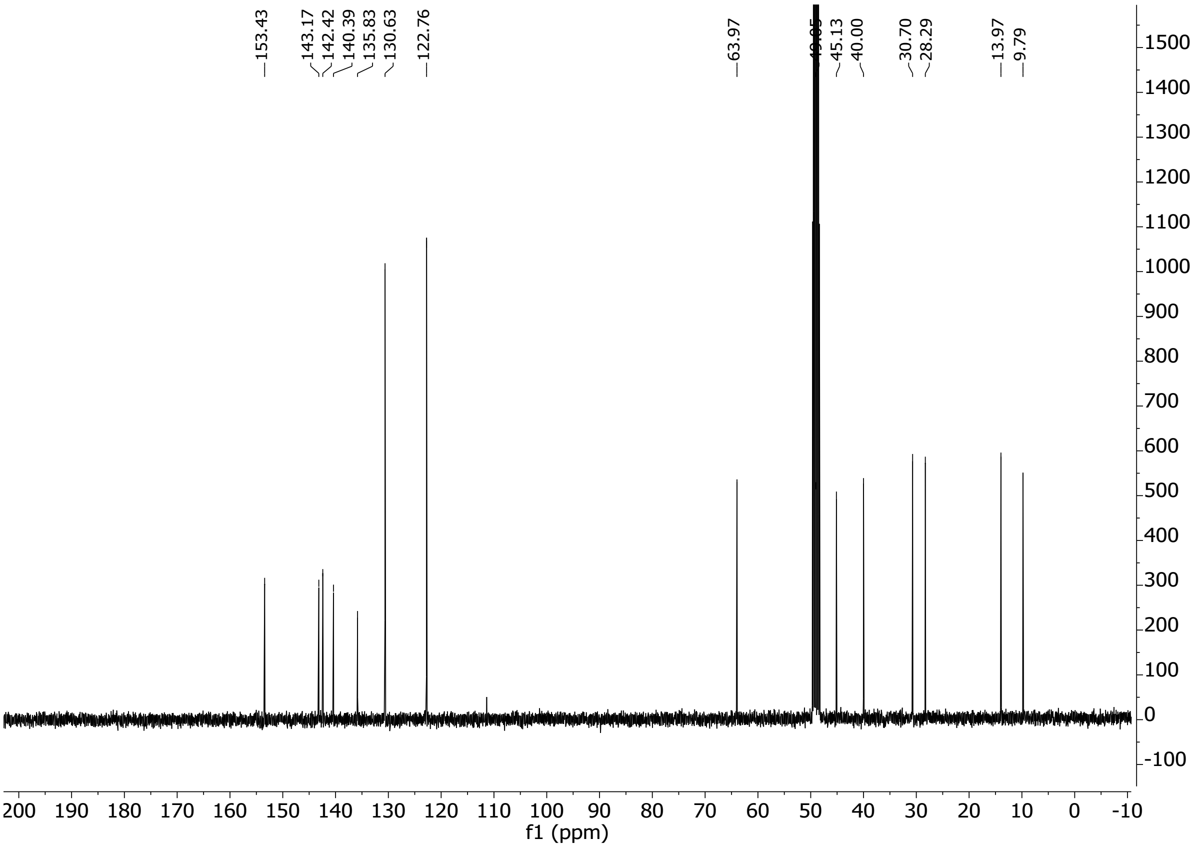

153.43
143.17 142.42 140.39 135.83 130.63
122.76
63.97
45.13 40.00
30.70 28.29
13.97 9.79
f1 (ppm)

Compound **25**

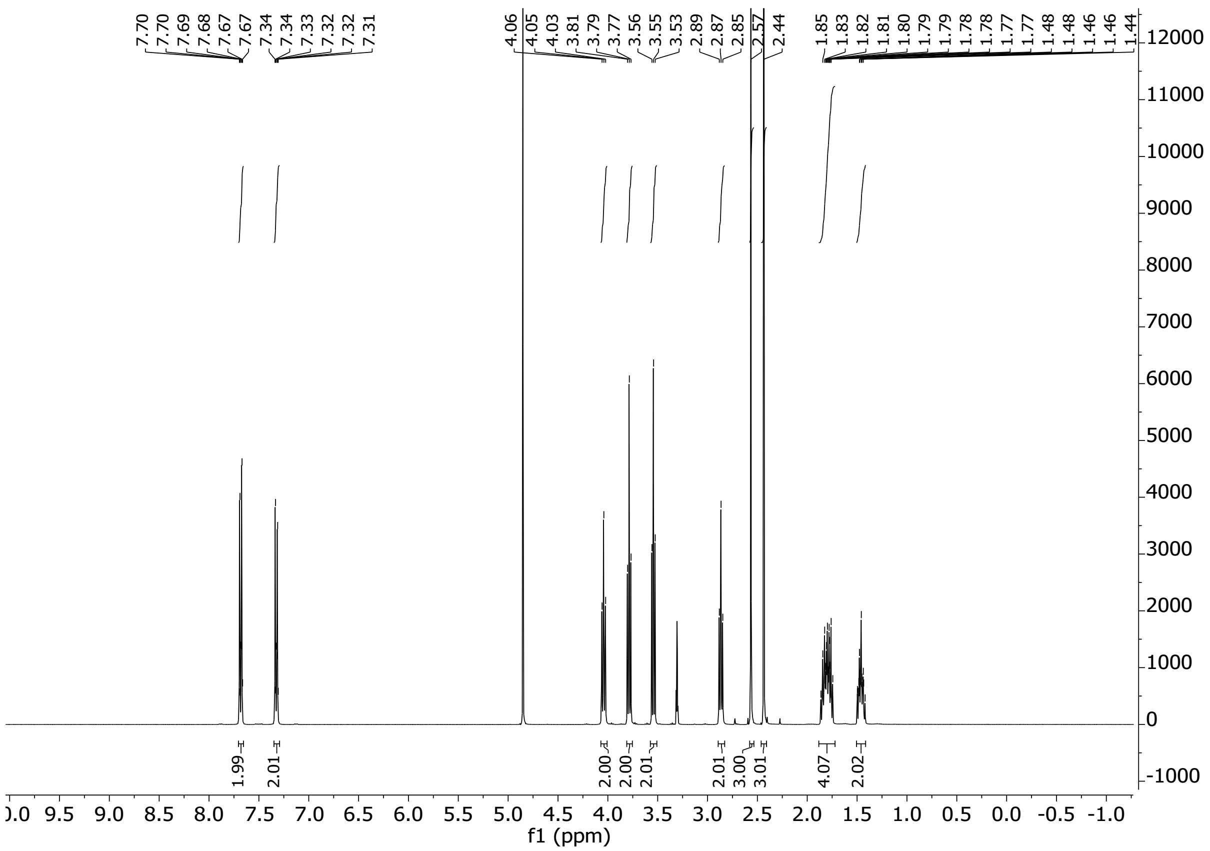

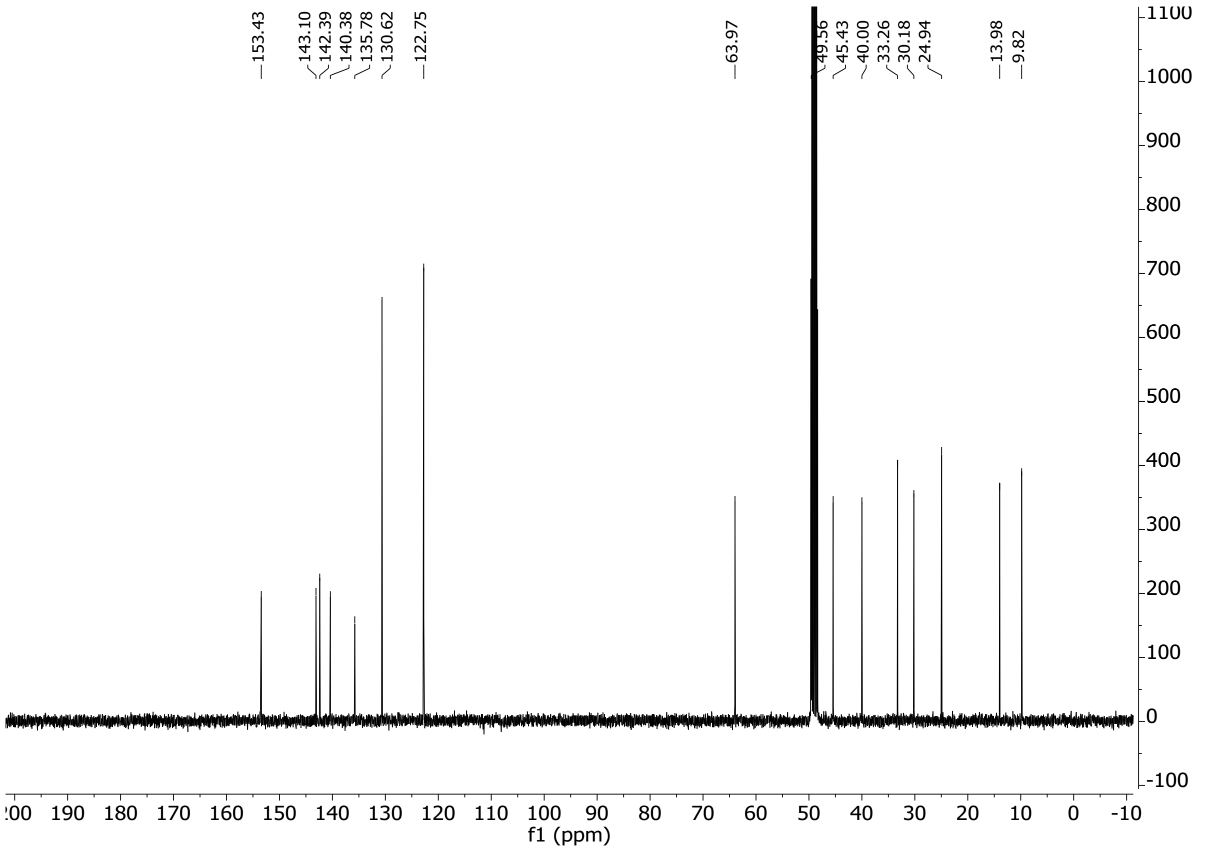

Compound **26**

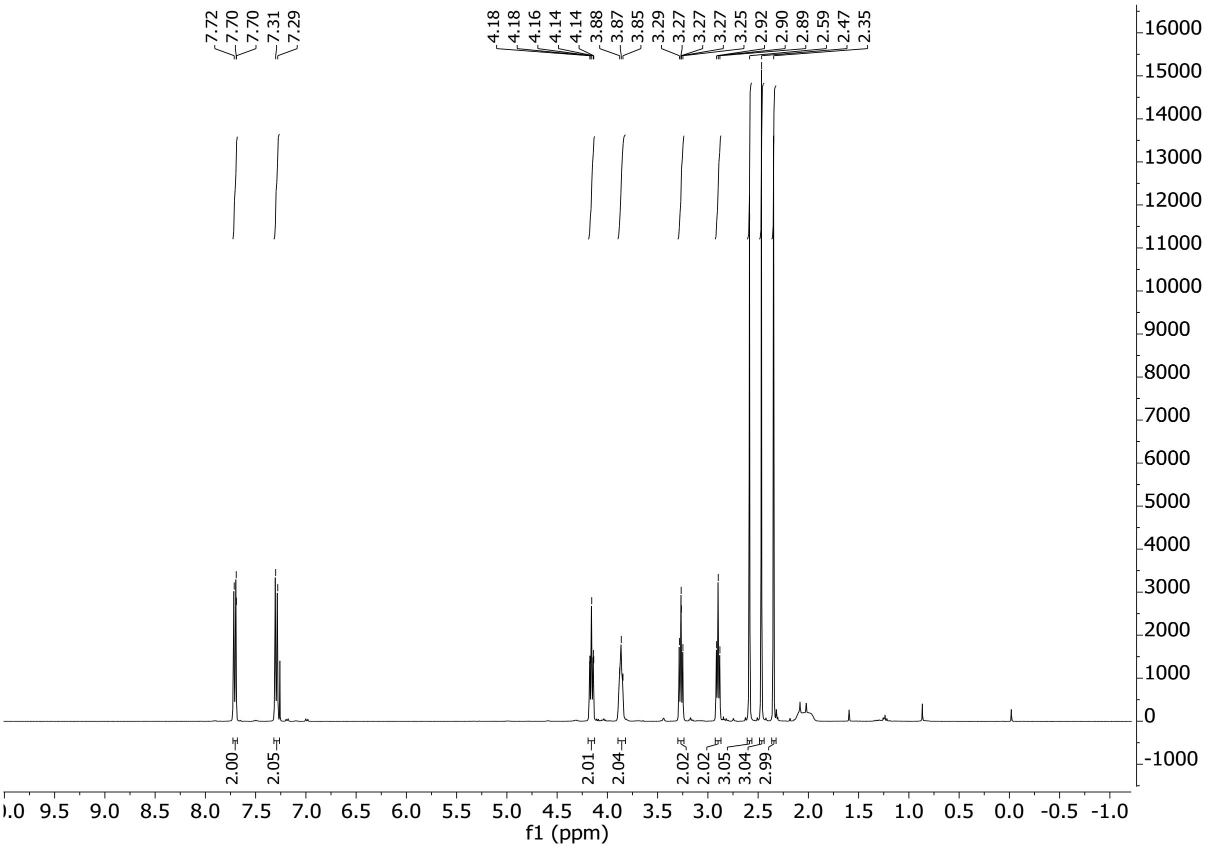
7.72
7.70
7.70
7.31
7.29
4.18
4.18
4.16
4.14
4.14
3.88
3.87
3.85
3.29
3.27
3.27
3.27
3.25
2.92
2.90
2.89
2.59
2.47
2.35
2.00
2.05
2.01
2.04
2.02
2.02
3.05
3.04
2.99
f1 (ppm)

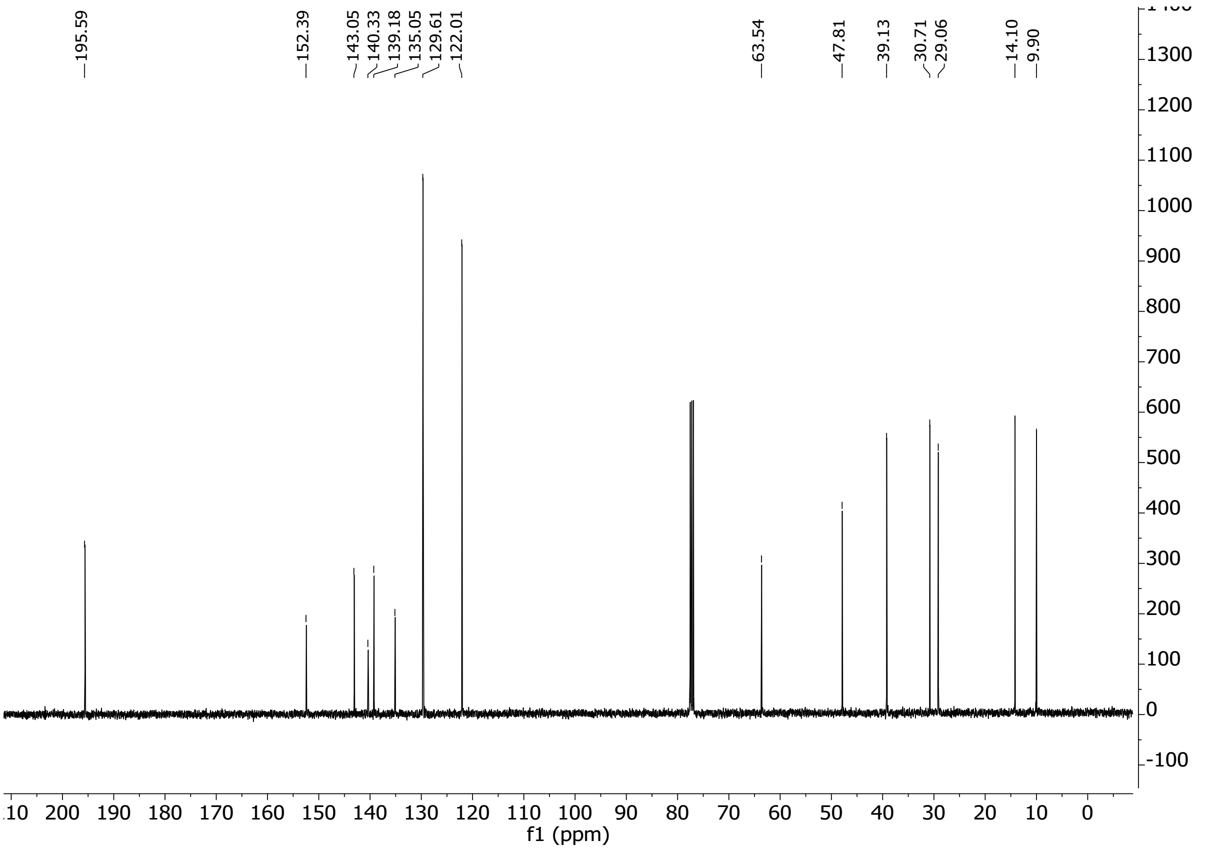
195.59
152.39
143.05
140.33
139.18
135.05
129.61
122.01
63.54
47.81
39.13
30.71
29.06
14.10
9.90
f1 (ppm)

Compound **27**

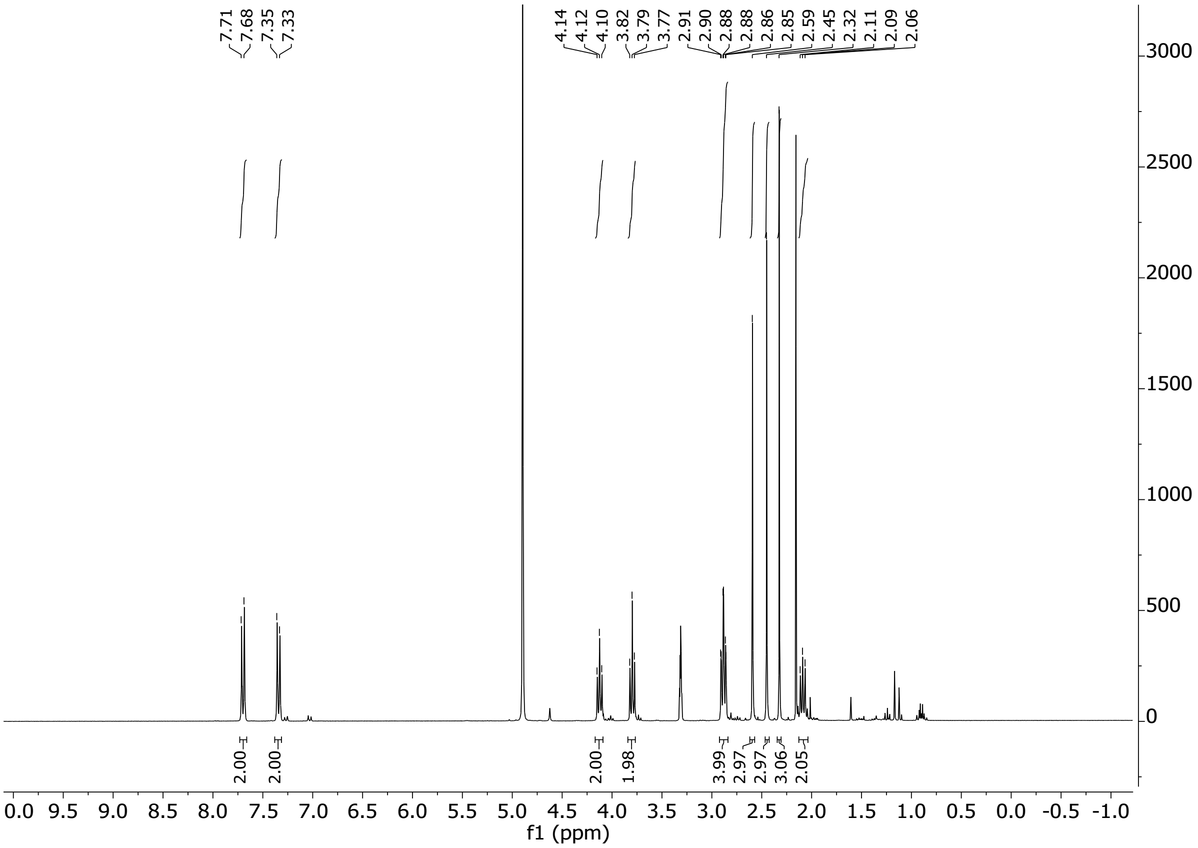

7.71
7.68
7.35
7.33
4.14
4.12
4.10
3.82
3.79
3.77
2.91
2.90
2.88
2.88
2.86
2.85
2.59
2.45
2.32
2.11
2.09
2.06
2.00
2.00
2.00
1.98
3.99
2.97
2.97
3.06
2.05
f1 (ppm)

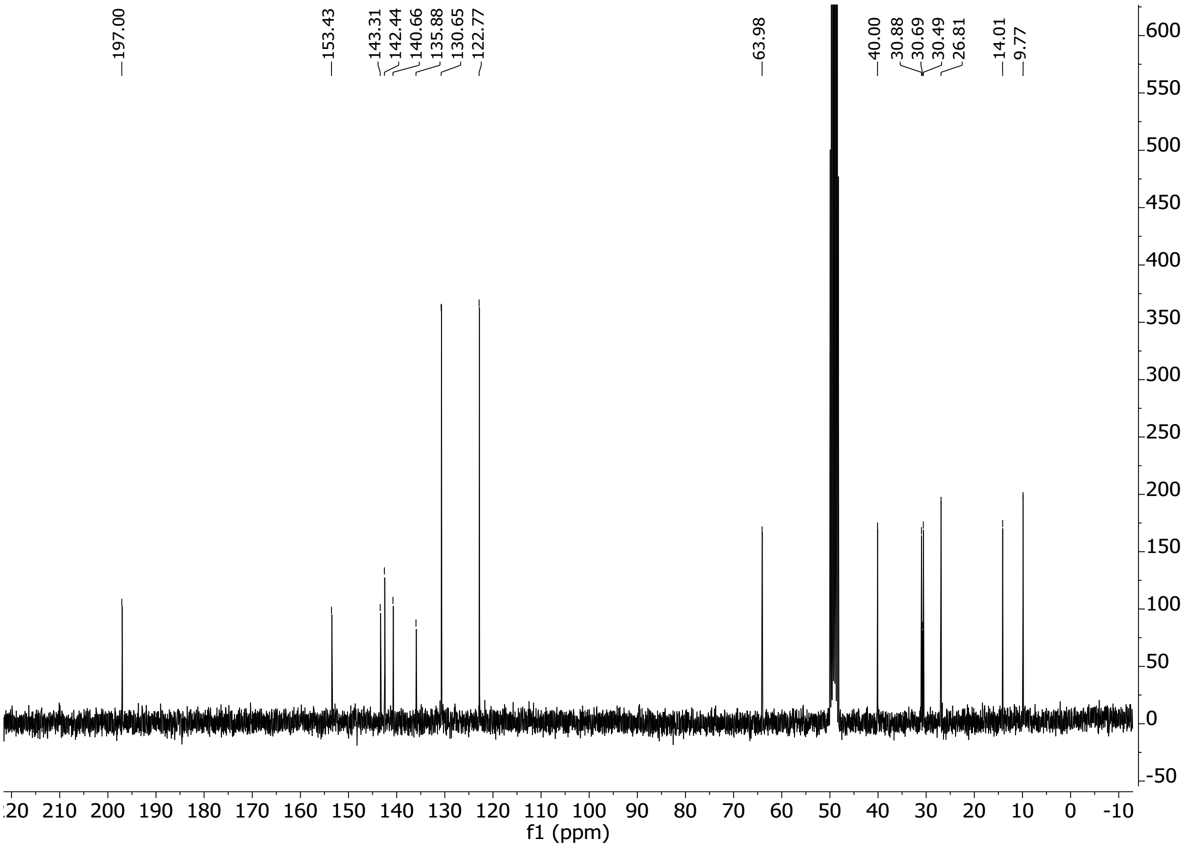

197.00
153.43
143.31
142.44
140.66
135.88
130.65
122.77
63.98
40.00
30.88
30.69
30.49
26.81
14.01
9.77
f1 (ppm)

Compound **28**

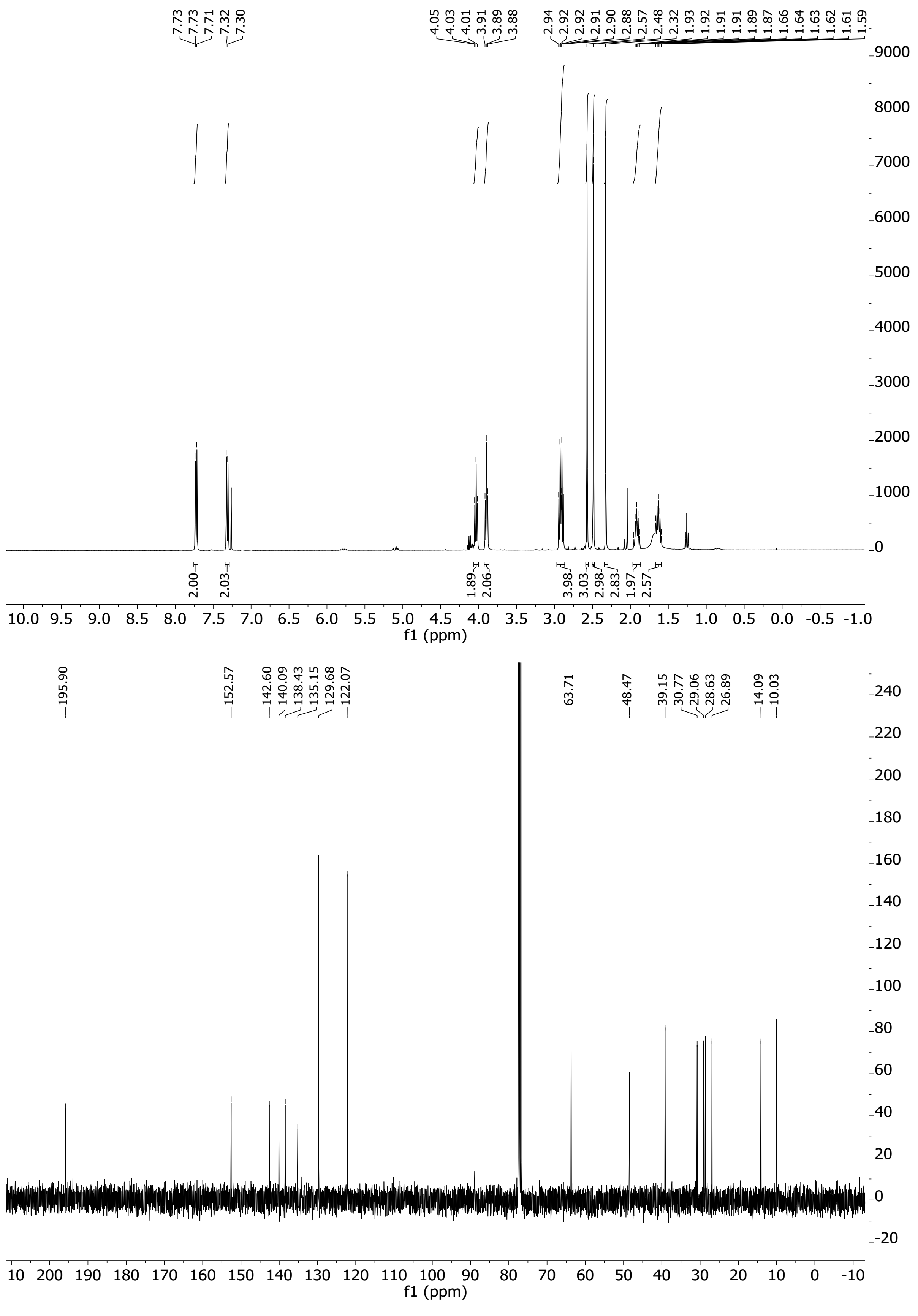

7.73
7.73
7.71
7.32
7.30
4.05
4.03
4.01
3.91
3.89
3.88
2.94
2.92
2.92
2.91
2.90
2.88
2.57
2.48
2.32
1.93
1.92
1.91
1.91
1.89
1.87
1.66
1.64
1.63
1.62
1.61
1.59
2.00
2.03
1.89
2.06
3.98
3.03
2.98
2.83
1.97
2.57
f1 (ppm)
195.90
152.57
142.60
140.09
138.43
135.15
129.68
122.07
63.71
48.47
39.15
30.77
29.06
28.63
26.89
14.09
10.03
f1 (ppm)

Compound **29**

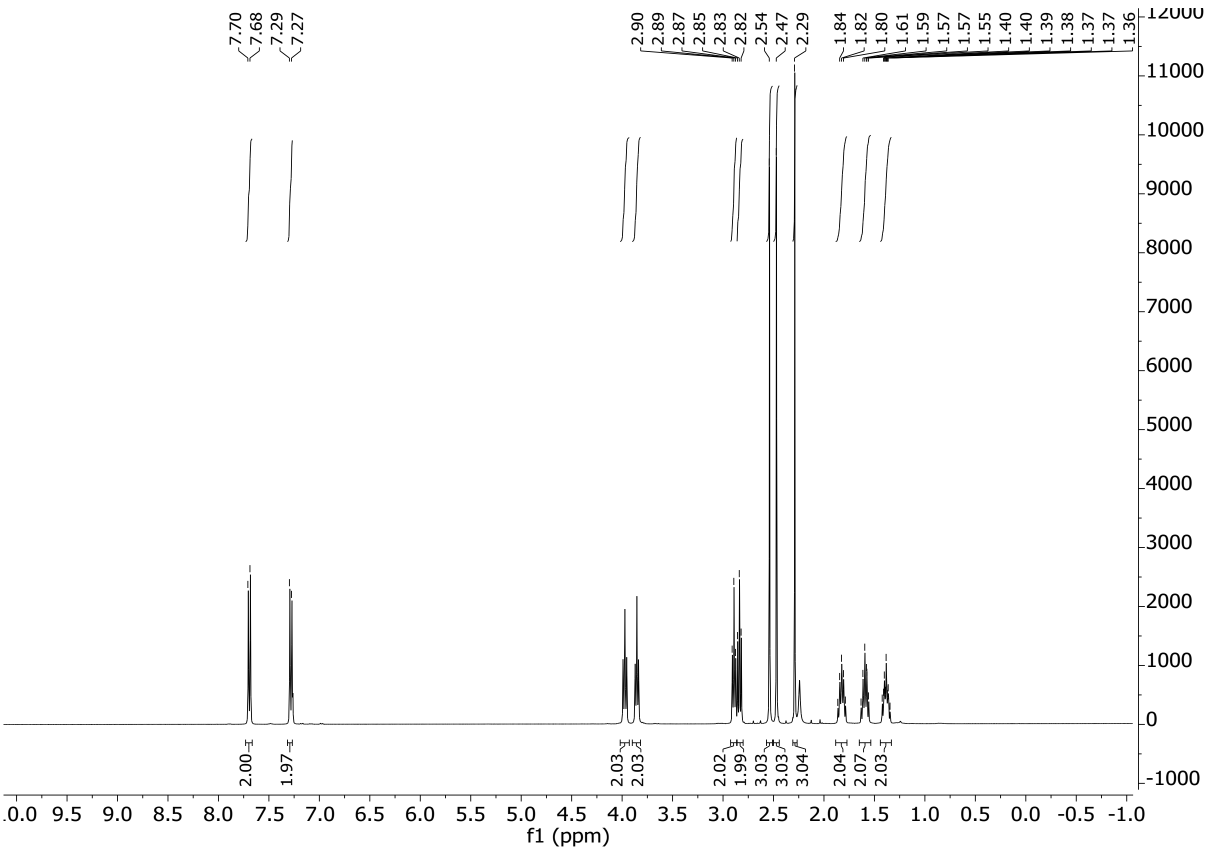

7.70
7.68
7.29
7.27
2.90
2.89
2.87
2.85
2.83
2.82
2.54
2.47
2.29
1.84
1.82
1.80
1.61
1.59
1.57
1.57
1.55
1.40
1.40
1.39
1.38
1.37
1.37
1.36
2.00
1.97
2.03
2.03
2.02
1.99
3.03
3.03
3.04
2.04
2.07
2.03
f1 (ppm)

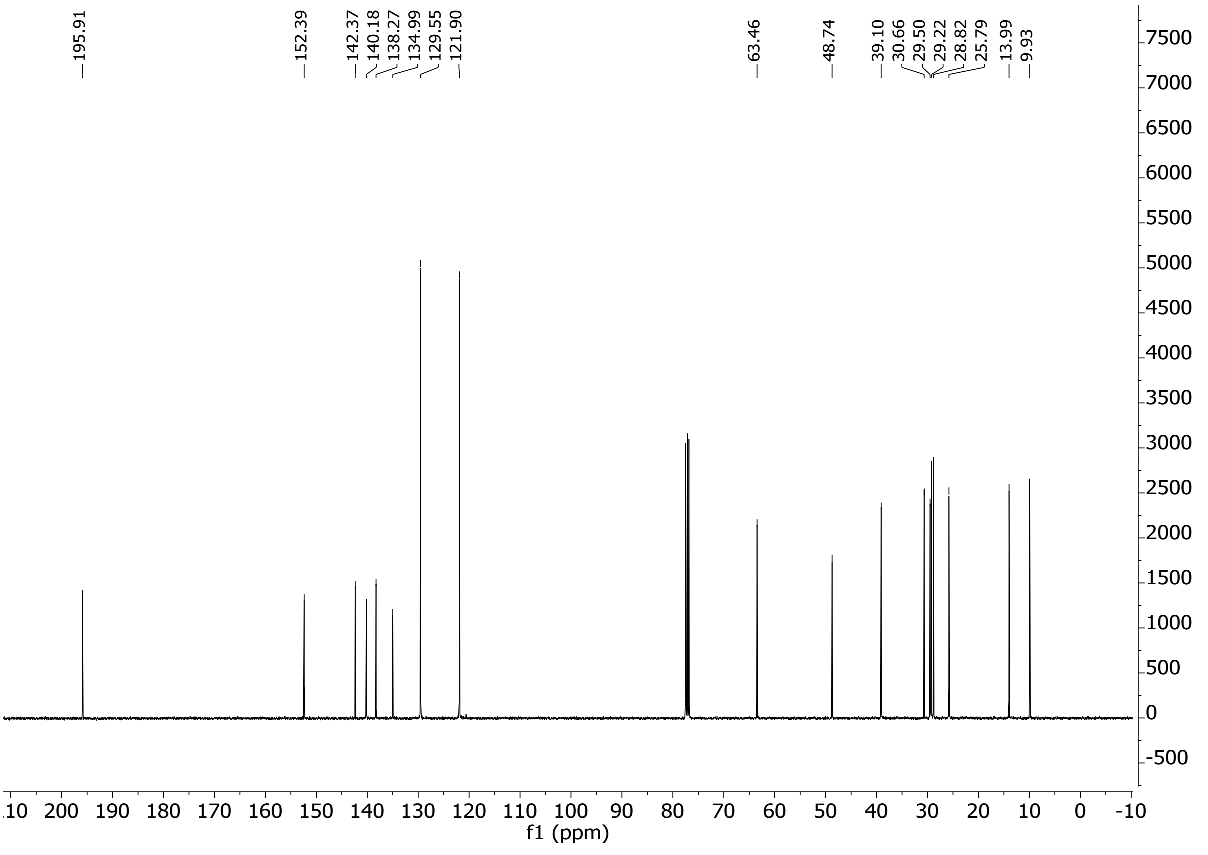

195.91
152.39
142.37
140.18
138.27
134.99
129.55
121.90
63.46
48.74
39.10
30.66
29.50
29.22
28.82
25.79
13.99
9.93
f1 (ppm)

Compound **30**

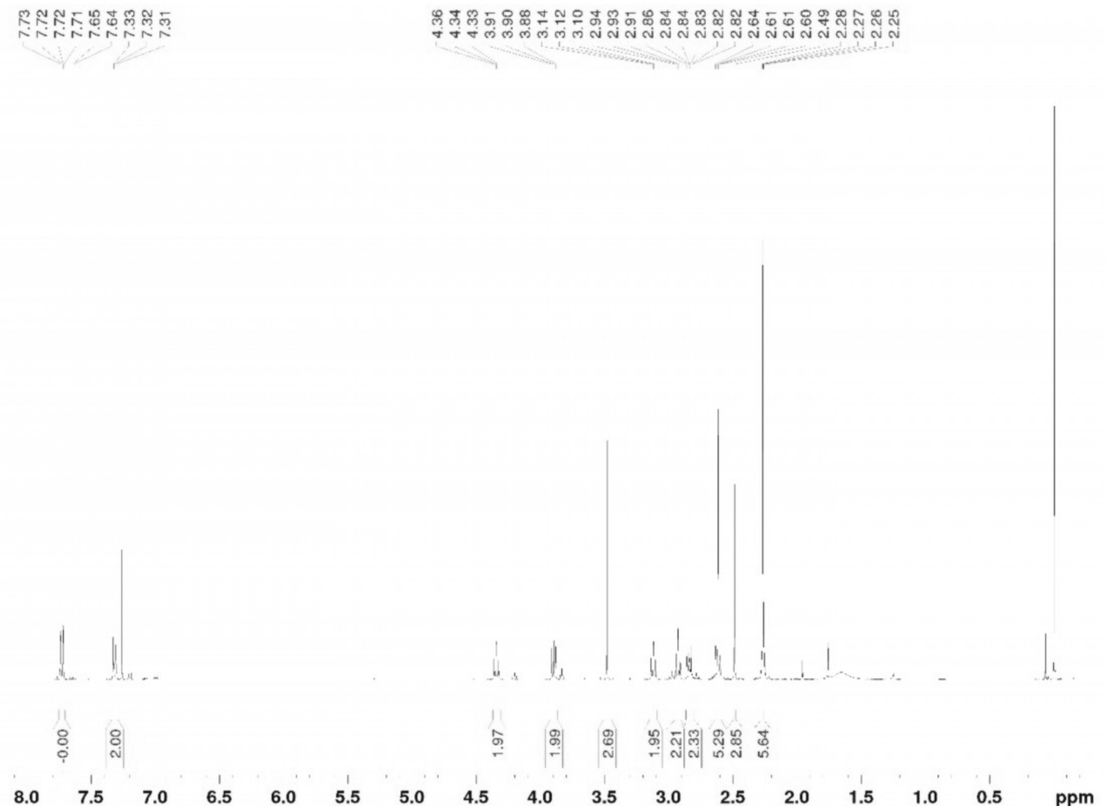
7.73
7.72
7.72
7.71
7.65
7.64
7.33
7.32
7.31
4.36
4.34
4.33
3.91
3.90
3.88
3.14
3.12
3.10
2.94
2.93
2.91
2.86
2.84
2.84
2.83
2.82
2.82
2.64
2.61
2.61
2.60
2.49
2.28
2.27
2.26
2.25
-0.00
2.00
1.97
1.99
2.69
1.95
2.21
2.33
5.29
2.85
5.64
8.0
7.5
7.0
6.5
6.0
5.5
5.0
4.5
4.0
3.5
3.0
2.5
2.0
1.5
1.0
0.5
ppm

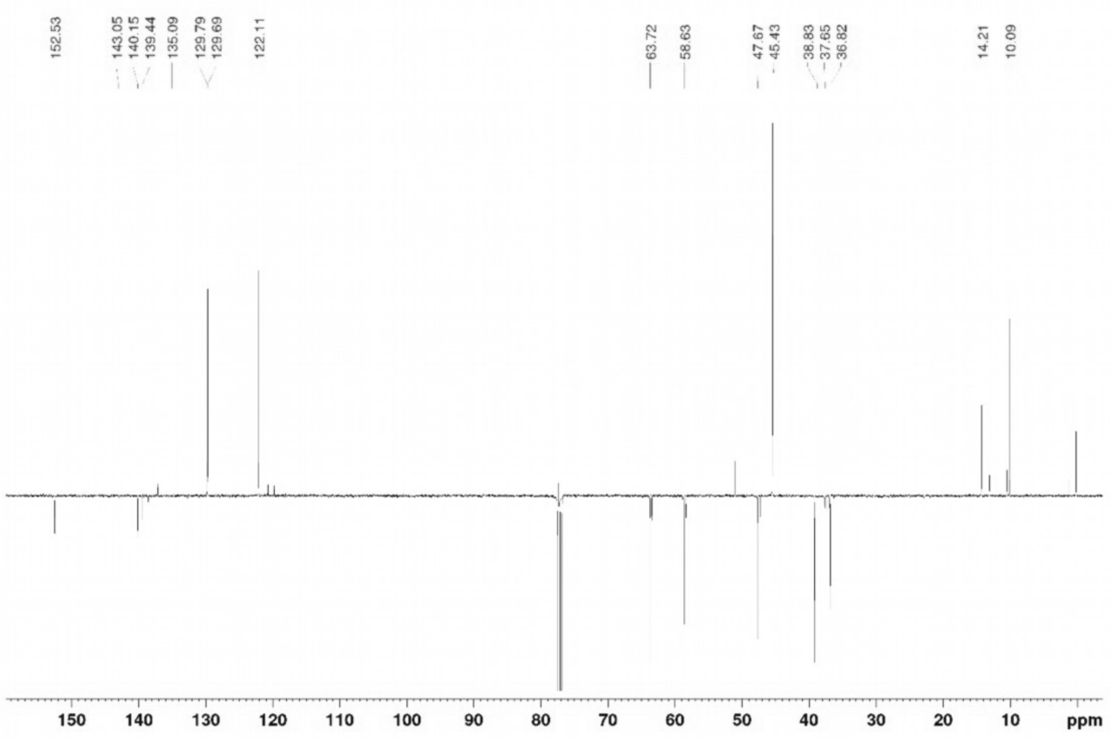
152.53
143.05
140.15
139.44
135.09
129.79
129.69
122.11
63.72
58.63
47.67
45.43
38.83
37.65
36.82
14.21
10.09
150
140
130
120
110
100
90
80
70
60
50
40
30
20
10
ppm

Compound **31**

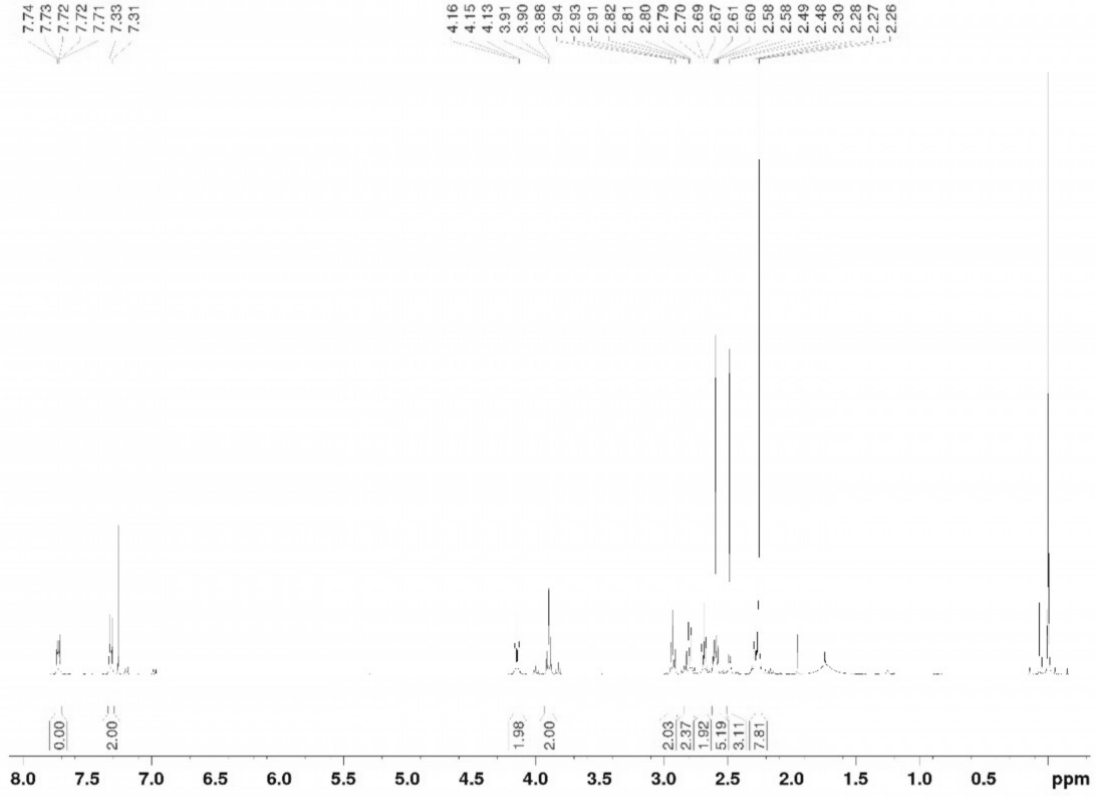
7.74
7.73
7.72
7.72
7.71
7.33
7.31
4.16
4.15
4.13
3.91
3.90
3.88
2.94
2.93
2.91
2.82
2.81
2.80
2.79
2.70
2.69
2.67
2.61
2.60
2.58
2.58
2.49
2.48
2.30
2.28
2.27
2.26
0.00
2.00
1.98
2.00
2.03
2.37
1.92
5.19
3.11
7.81
8.0 7.5 7.0 6.5 6.0 5.5 5.0 4.5 4.0 3.5 3.0 2.5 2.0 1.5 1.0 0.5 ppm

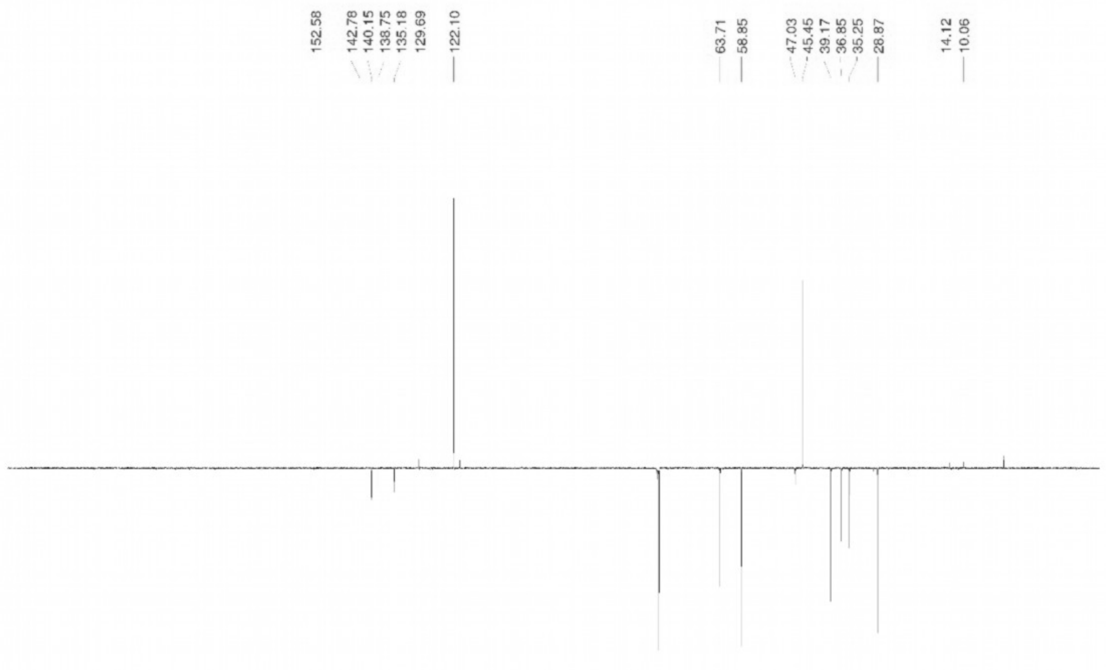
152.58
142.78
140.15
138.75
135.18
129.69
122.10
63.71
58.85
47.03
45.45
39.17
36.85
35.25
28.87
14.12
10.06
210 200 190 180 170 160 150 140 130 120 110 100 90 80 70 60 50 40 30 20 10 0 ppm

Compound **1**

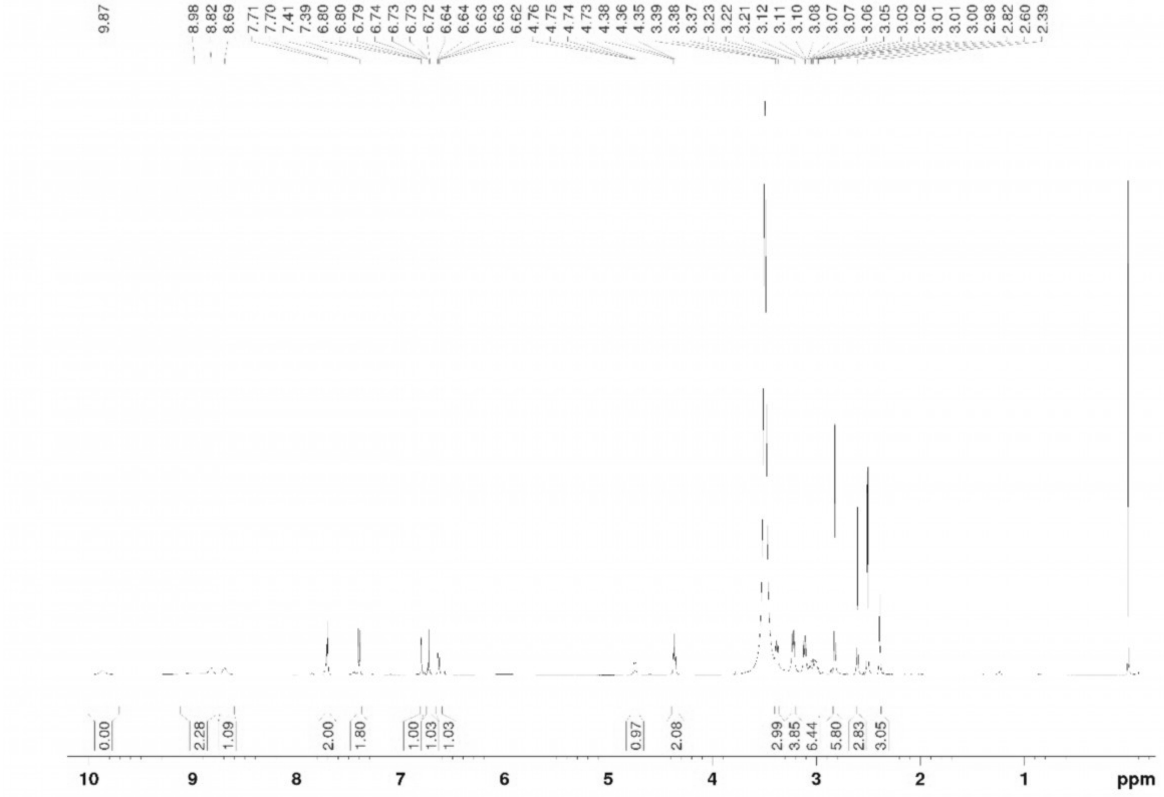

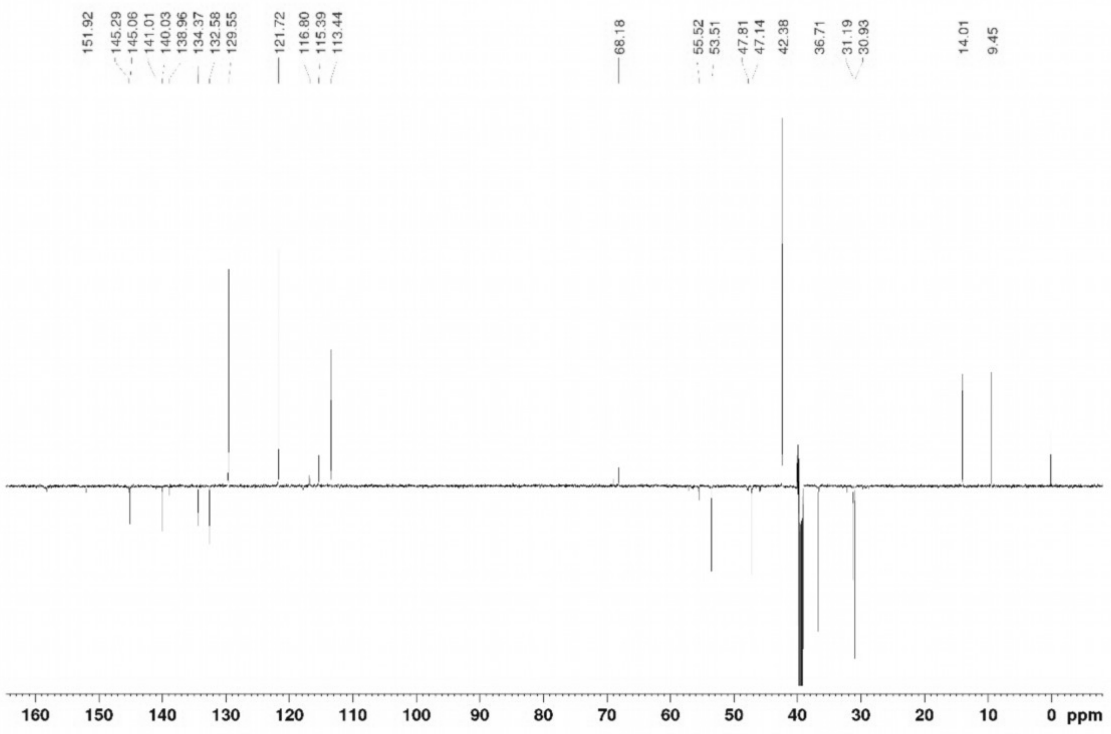

Compound **2**

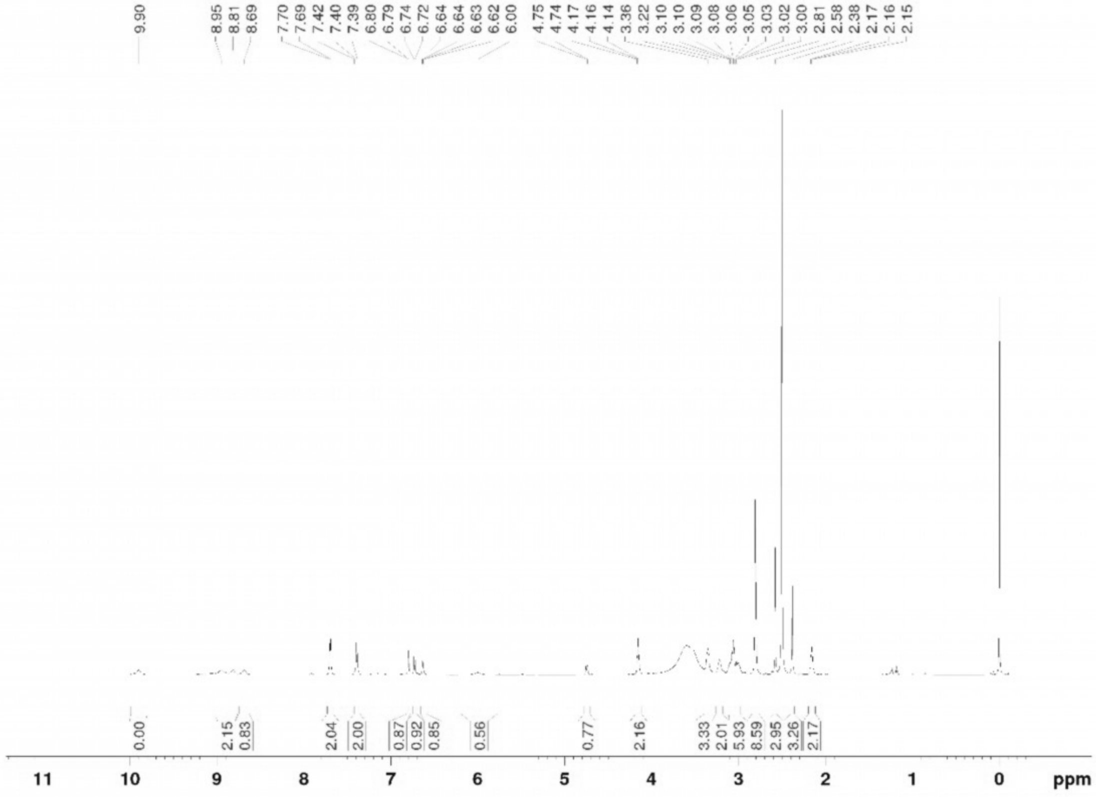

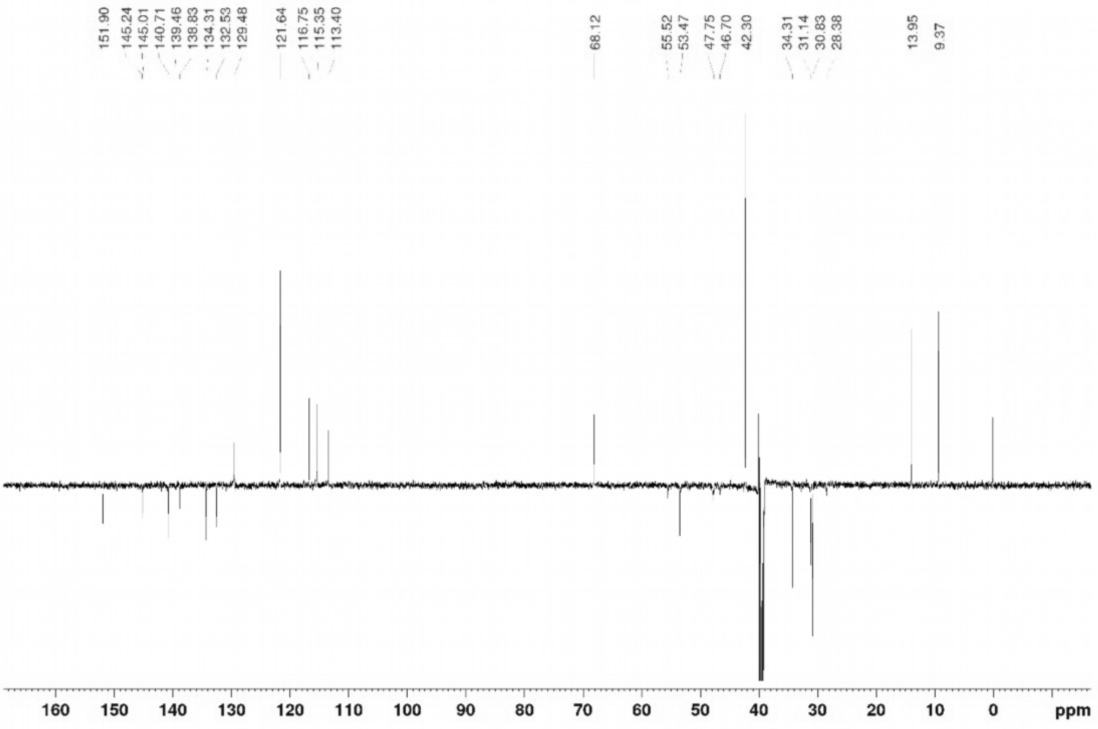

Compound **34**

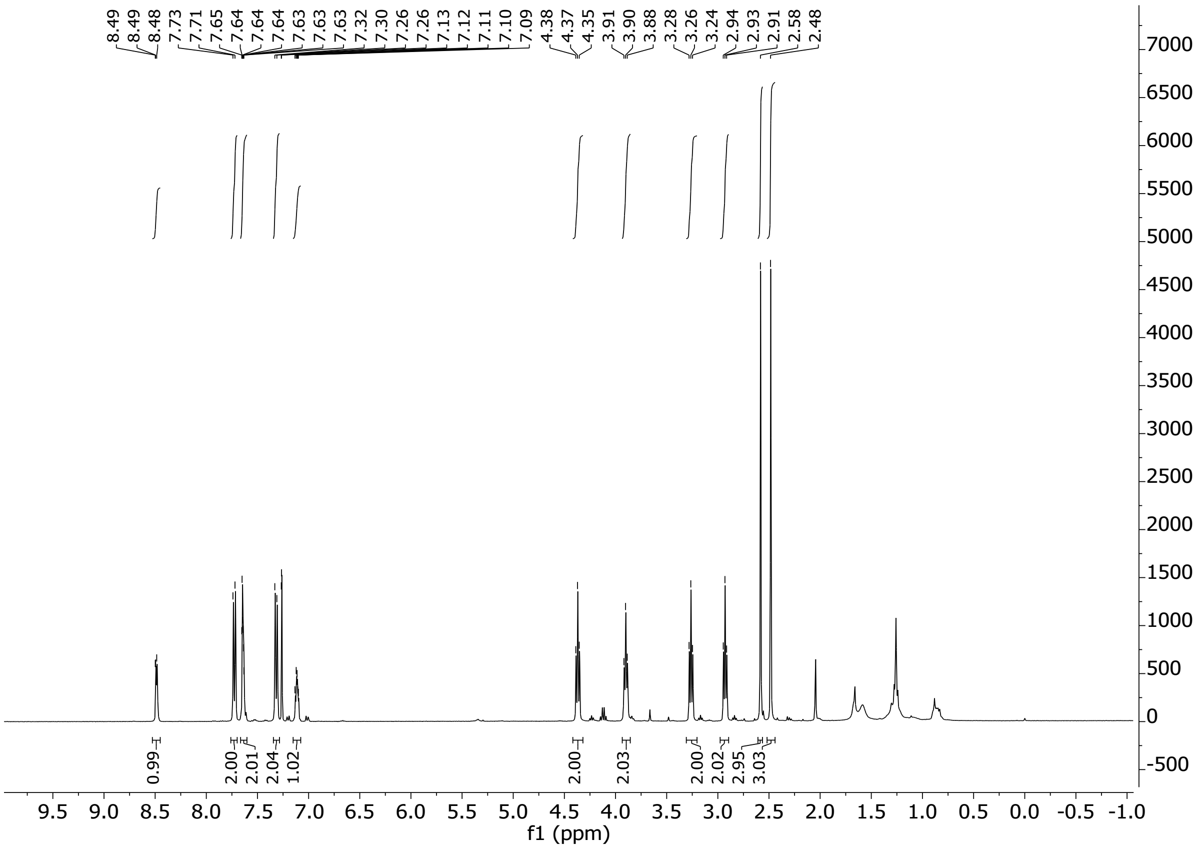

8.49
8.49
8.48
7.73
7.71
7.65
7.64
7.64
7.64
7.63
7.63
7.63
7.32
7.30
7.26
7.26
7.13
7.12
7.11
7.10
7.09
4.38
4.37
4.35
3.91
3.90
3.88
3.28
3.26
3.24
2.94
2.93
2.91
2.58
2.48
0.99
2.00
2.01
2.04
1.02
2.00
2.03
2.00
2.02
2.95
3.03
f1 (ppm)

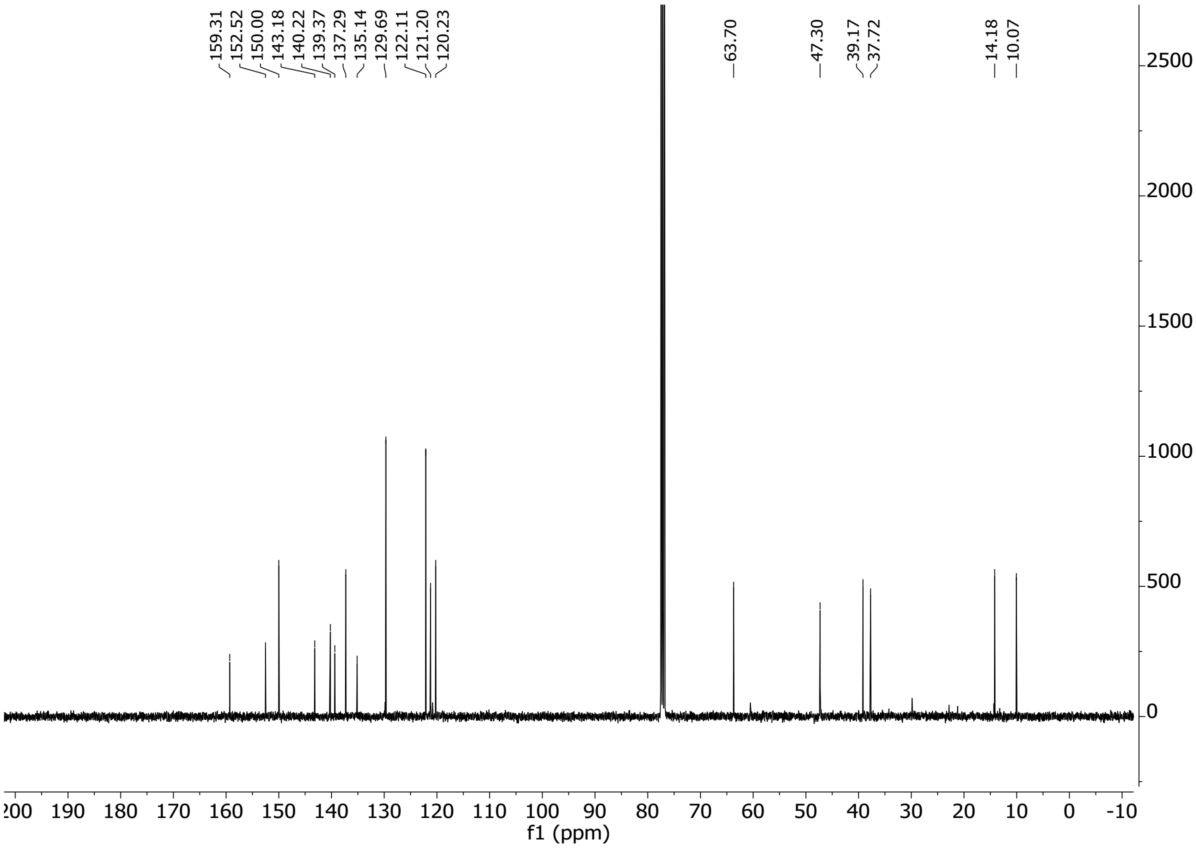

159.31
152.52
150.00
143.18
140.22
139.37
137.29
135.14
129.69
122.11
121.20
120.23
63.70
47.30
39.17
37.72
14.18
10.07
f1 (ppm)

Compound **35**

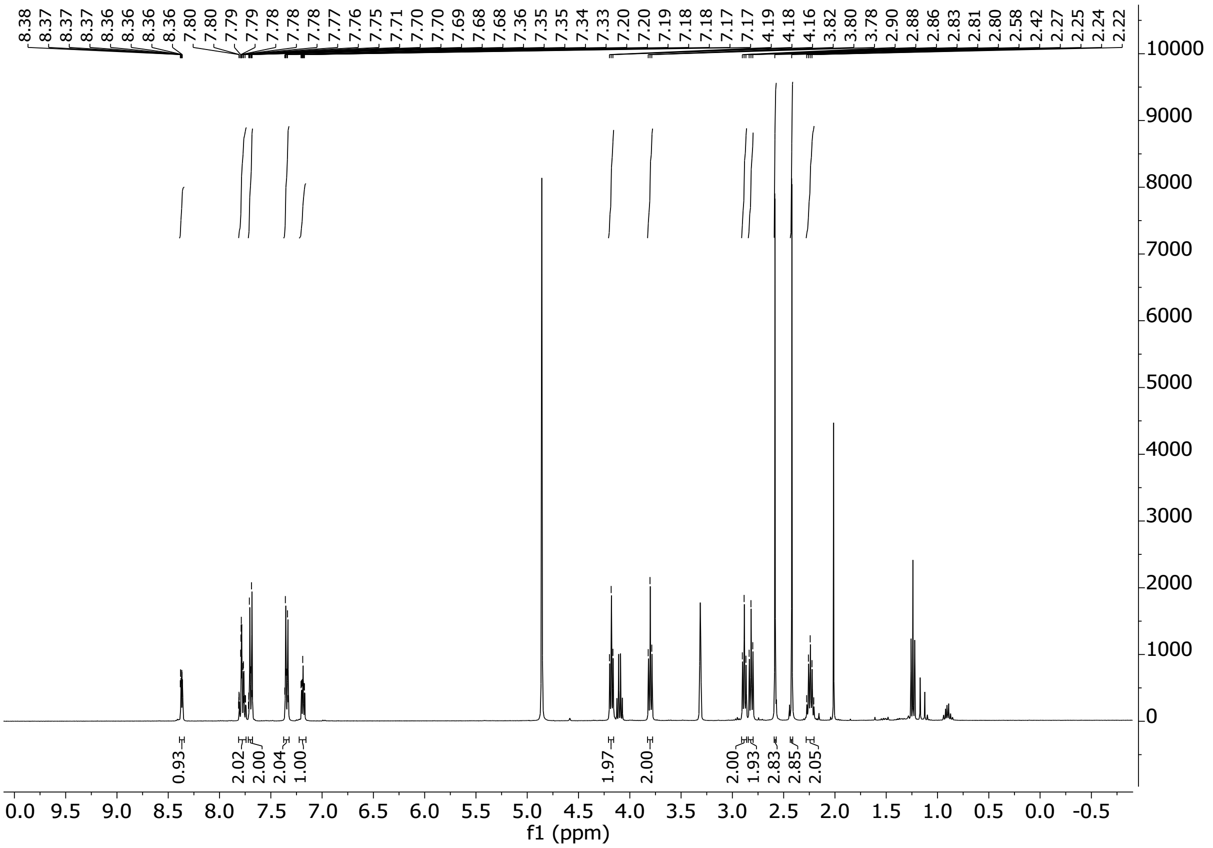

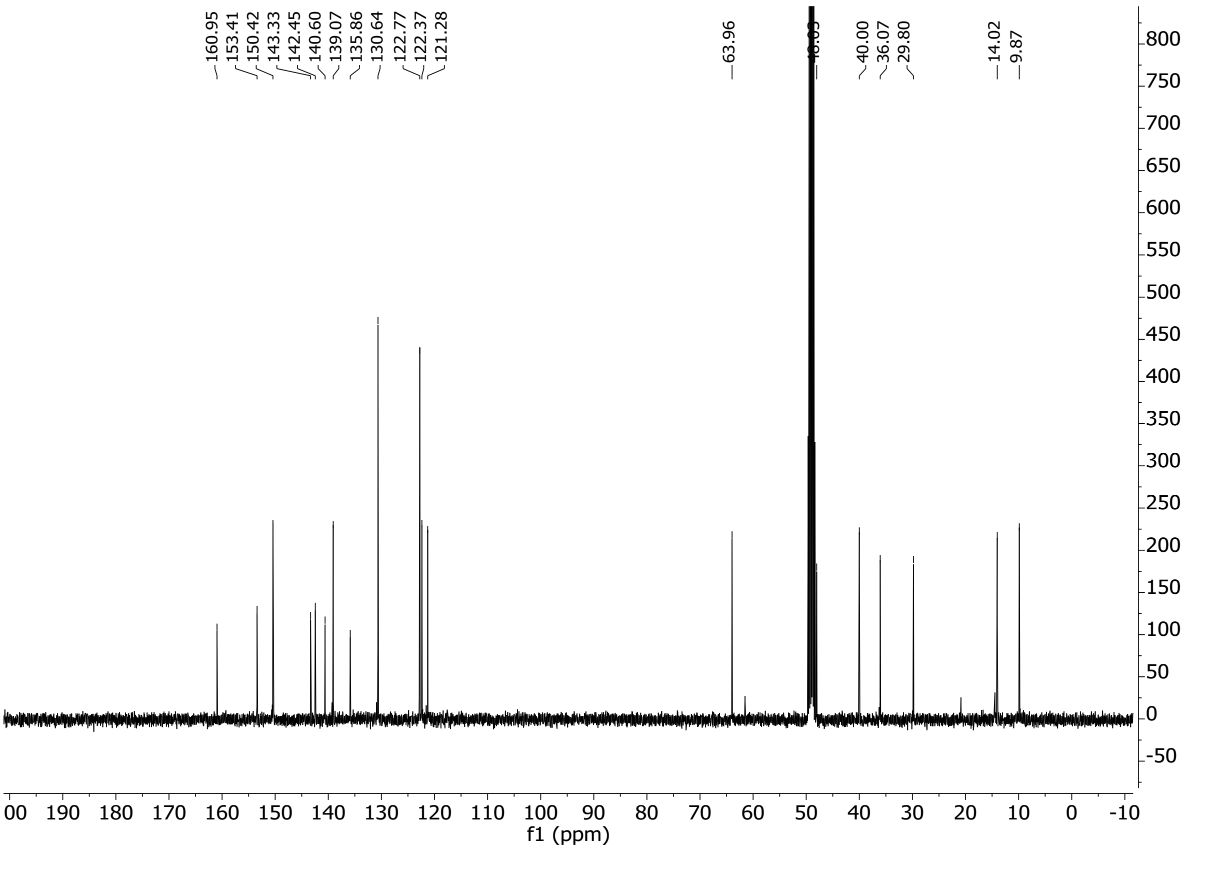

Compound **36**

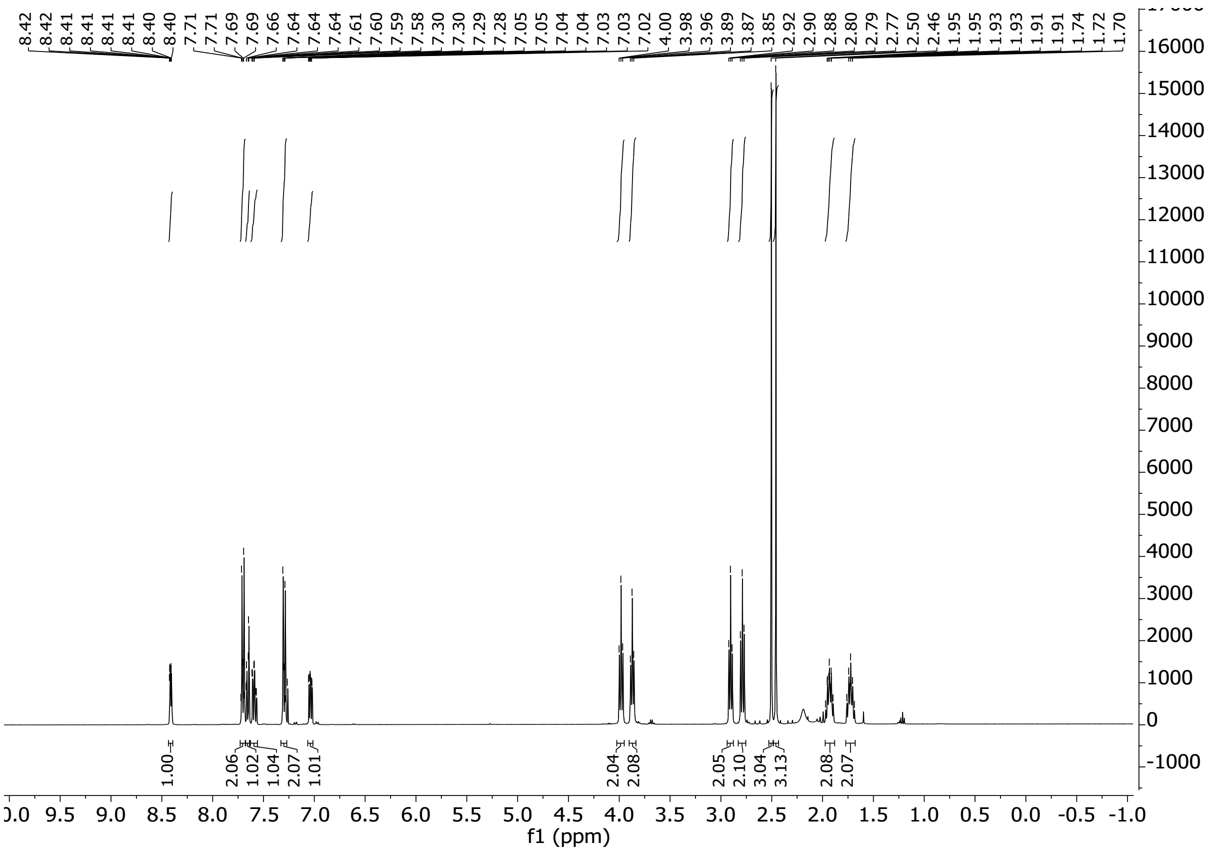

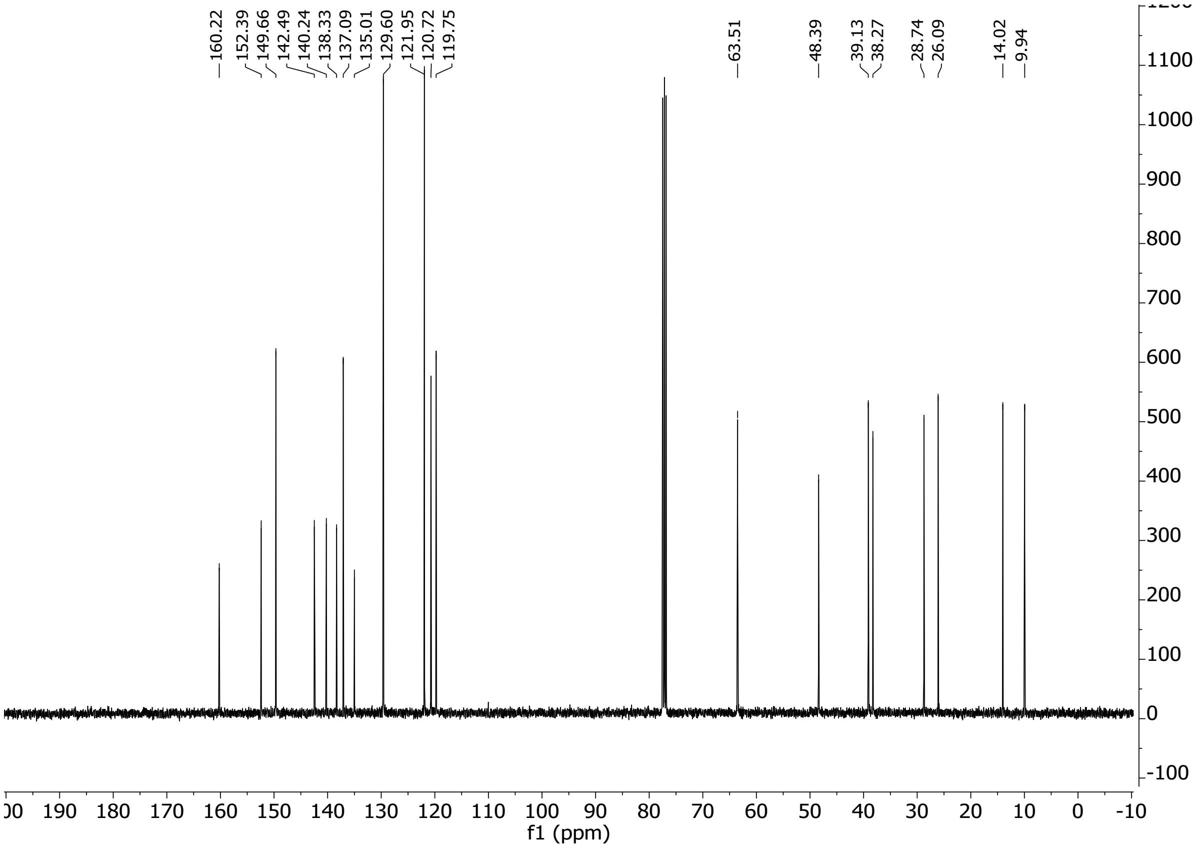

Compound **37**

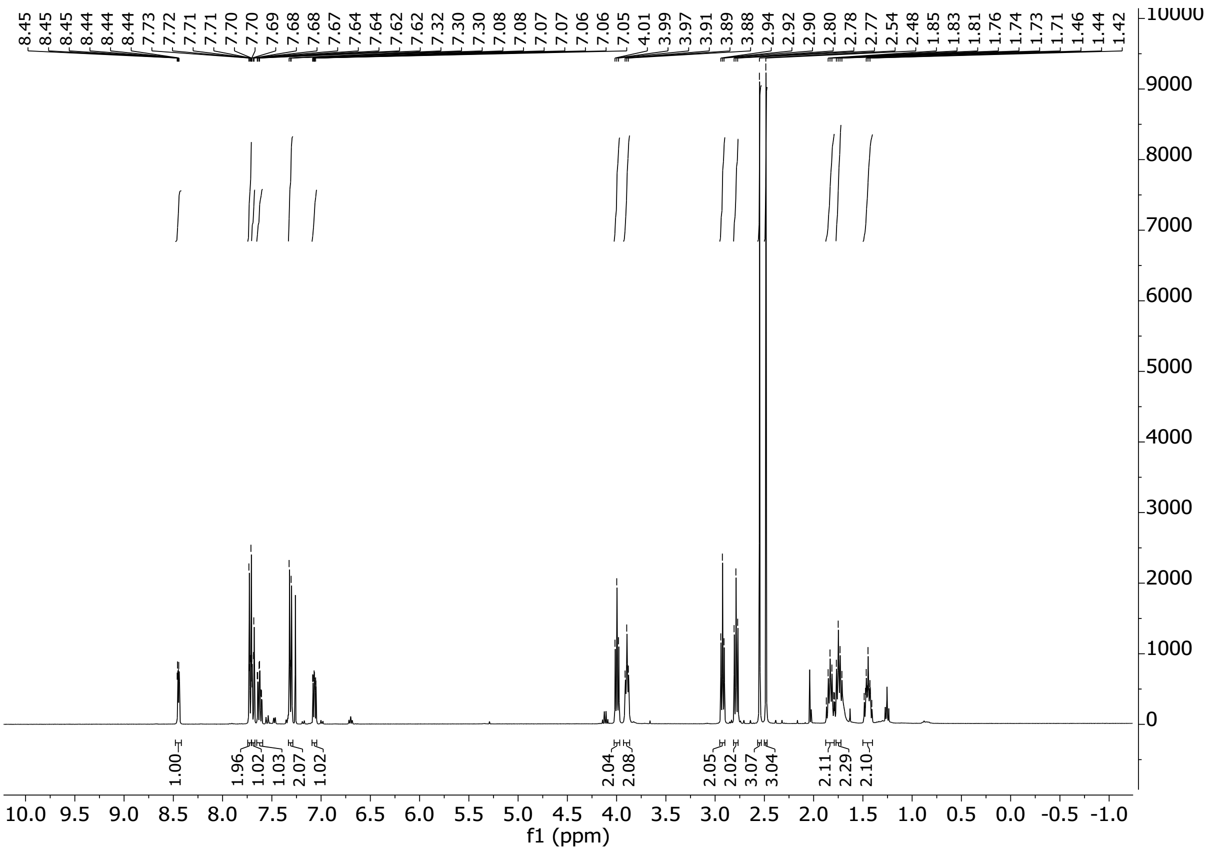

8.45
8.45
8.45
8.44
8.44
8.44
7.73
7.72
7.71
7.71
7.70
7.70
7.69
7.68
7.68
7.67
7.64
7.64
7.62
7.62
7.32
7.30
7.08
7.08
7.07
7.07
7.06
7.05
4.01
3.99
3.97
3.91
3.89
3.88
2.94
2.92
2.90
2.80
2.78
2.77
2.54
2.48
1.85
1.83
1.81
1.76
1.74
1.73
1.71
1.46
1.44
1.42
1.00
1.96
1.02
1.03
2.07
1.02
2.04
2.08
2.05
2.02
3.07
3.04
2.11
2.29
2.10
f1 (ppm)

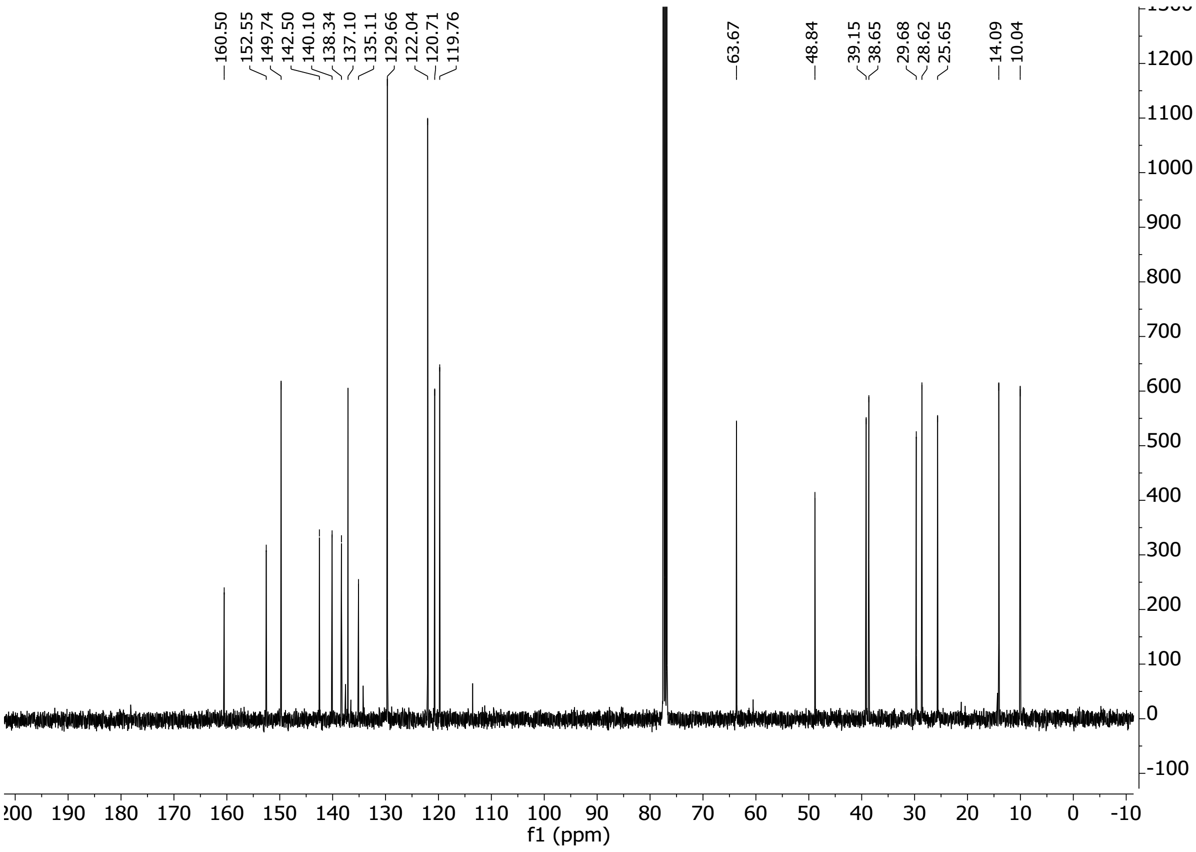

160.50
152.55
149.74
142.50
140.10
138.34
137.10
135.11
129.66
122.04
120.71
119.76
63.67
48.84
39.15
38.65
29.68
28.62
25.65
14.09
10.04
f1 (ppm)

Compound **3**

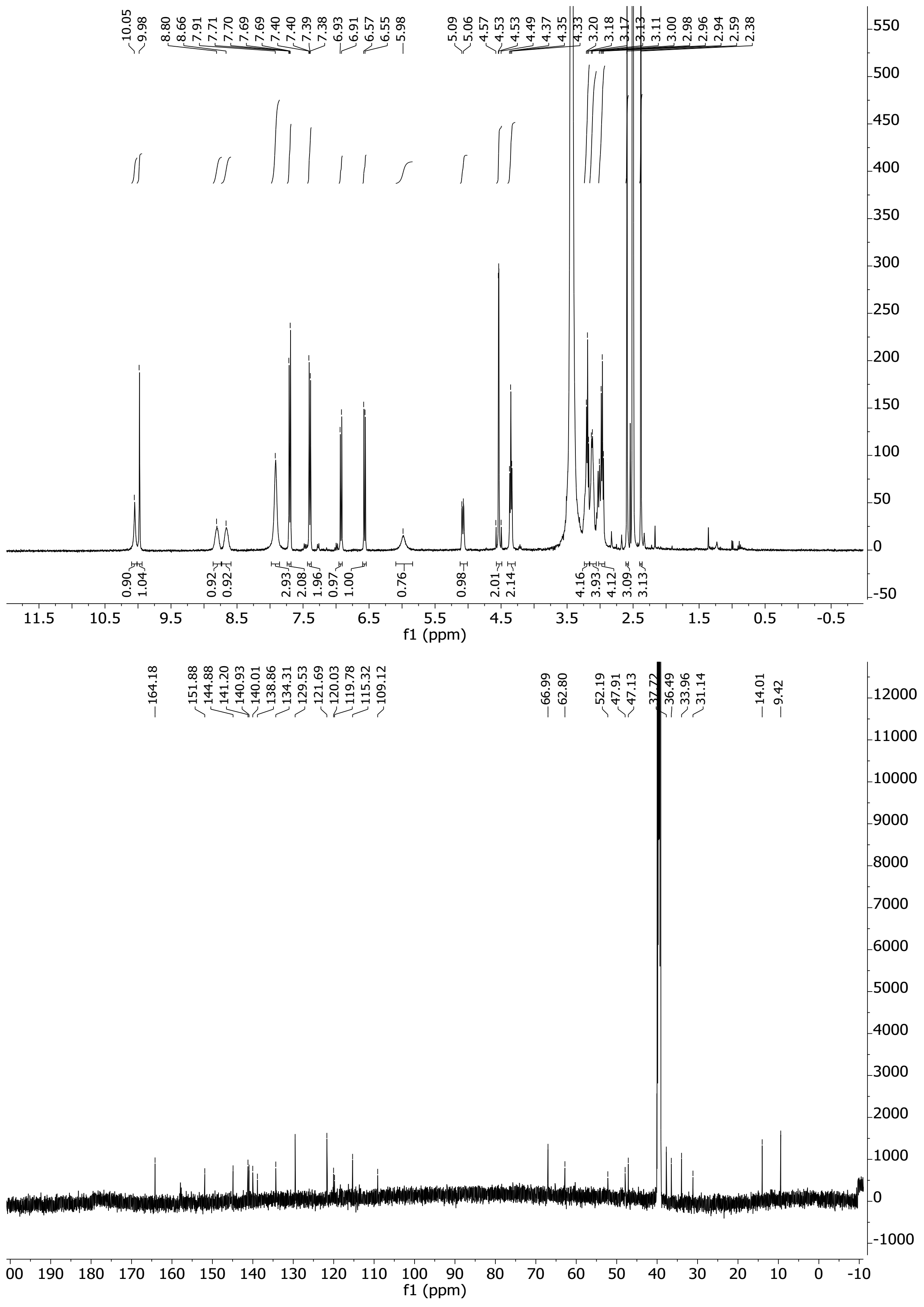

Compound **4**

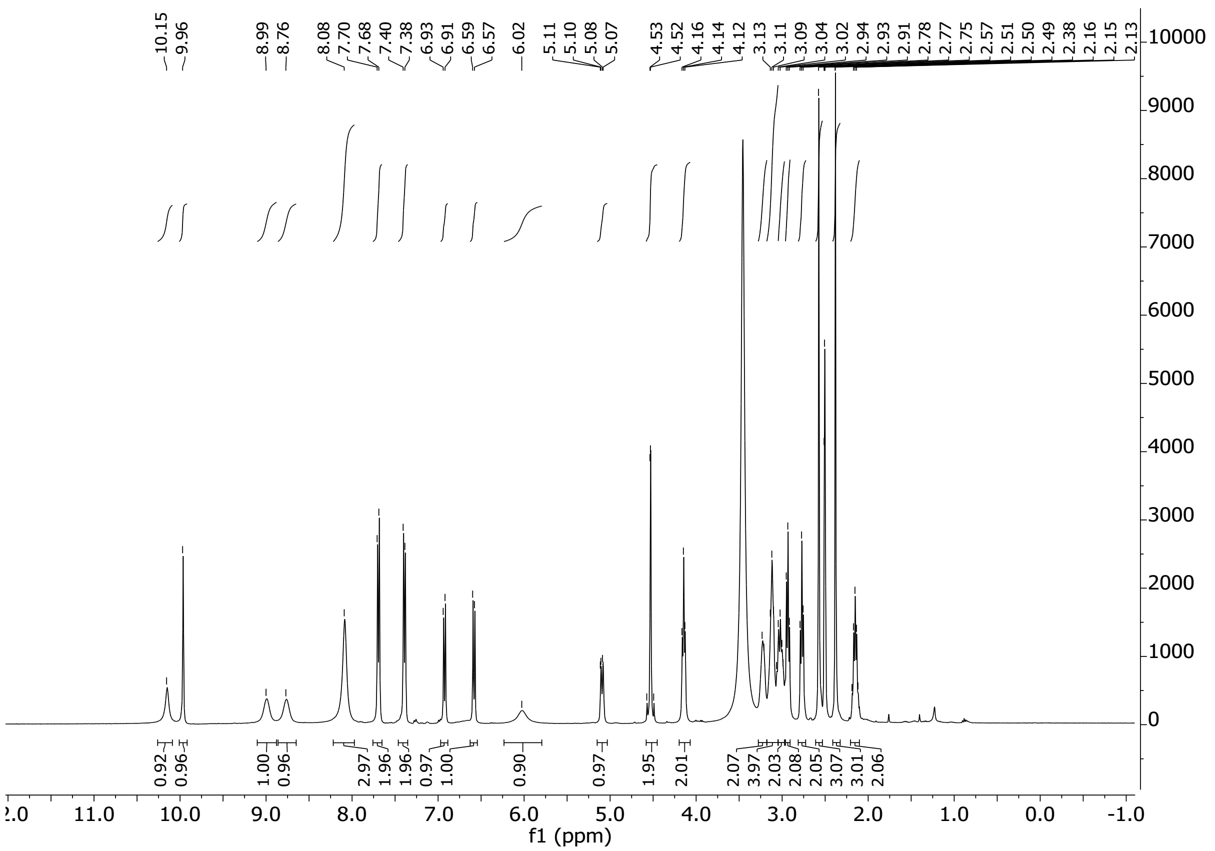

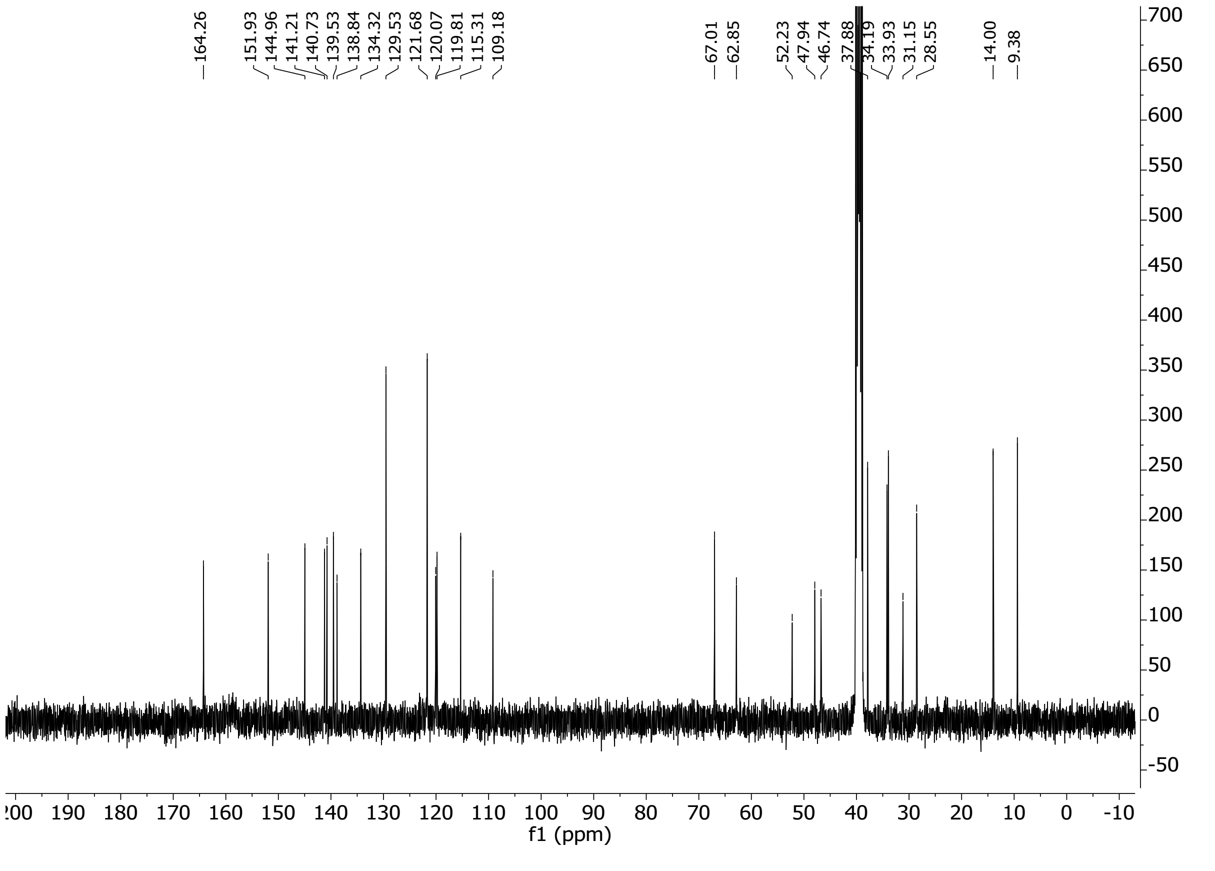

Compound **5**

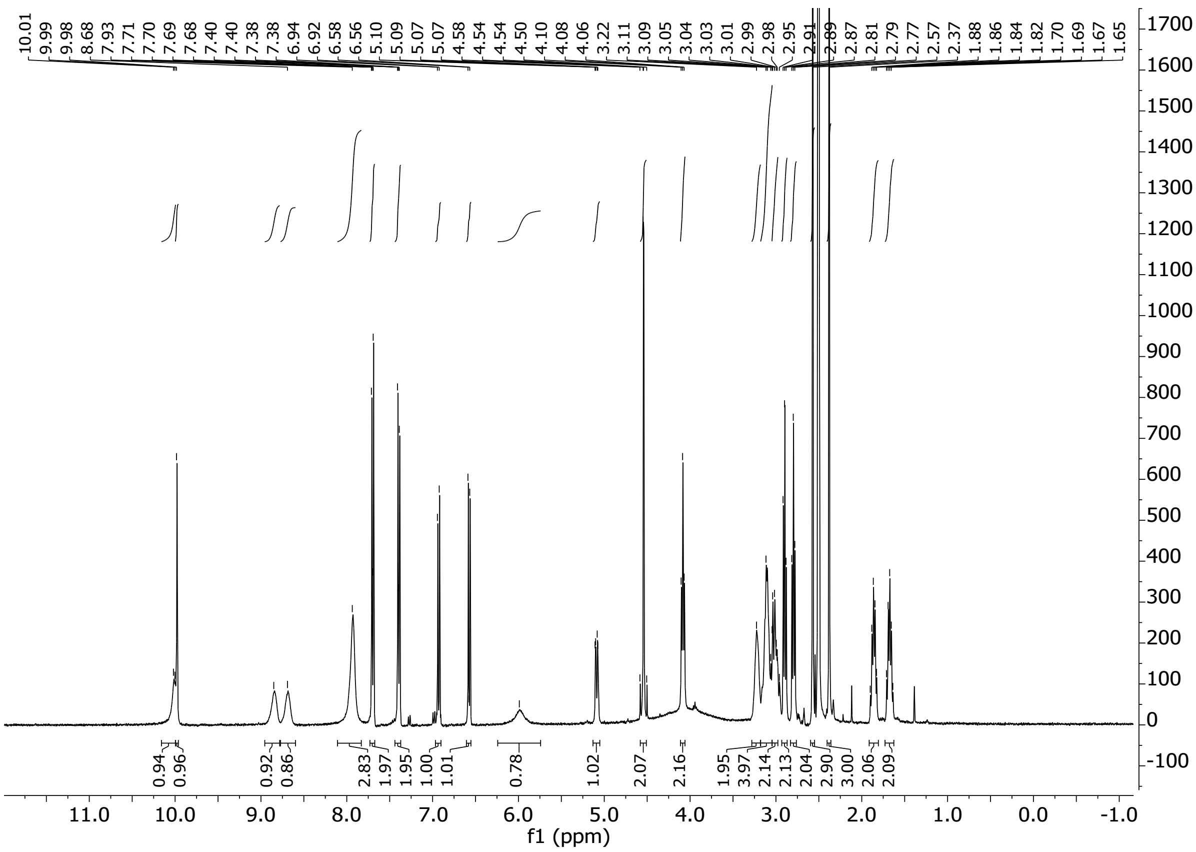
10.01 9.99 9.98 8.68 7.93 7.71 7.70 7.69 7.68 7.40 7.40 7.38 7.38 6.94 6.92 6.58 6.56 5.10 5.09 5.07 5.07 4.58 4.54 4.54 4.50 4.10 4.08 4.06 3.22 3.11 3.09 3.05 3.04 3.03 3.01 2.99 2.98 2.95 2.91 2.89 2.87 2.81 2.79 2.77 2.57 2.37 1.88 1.86 1.84 1.82 1.70 1.69 1.67 1.65
0.94 0.96 0.92 0.86 2.83 1.97 1.95 1.00 1.01 0.78 1.02 2.07 2.16 1.95 3.97 2.14 2.13 2.04 2.90 3.00 2.06 2.09
11.0 10.0 9.0 8.0 7.0 6.0 5.0 4.0 3.0 2.0 1.0 0.0 -1.0
f1 (ppm)
1700 1600 1500 1400 1300 1200 1100 1000 900 800 700 600 500 400 300 200 100 0 -100

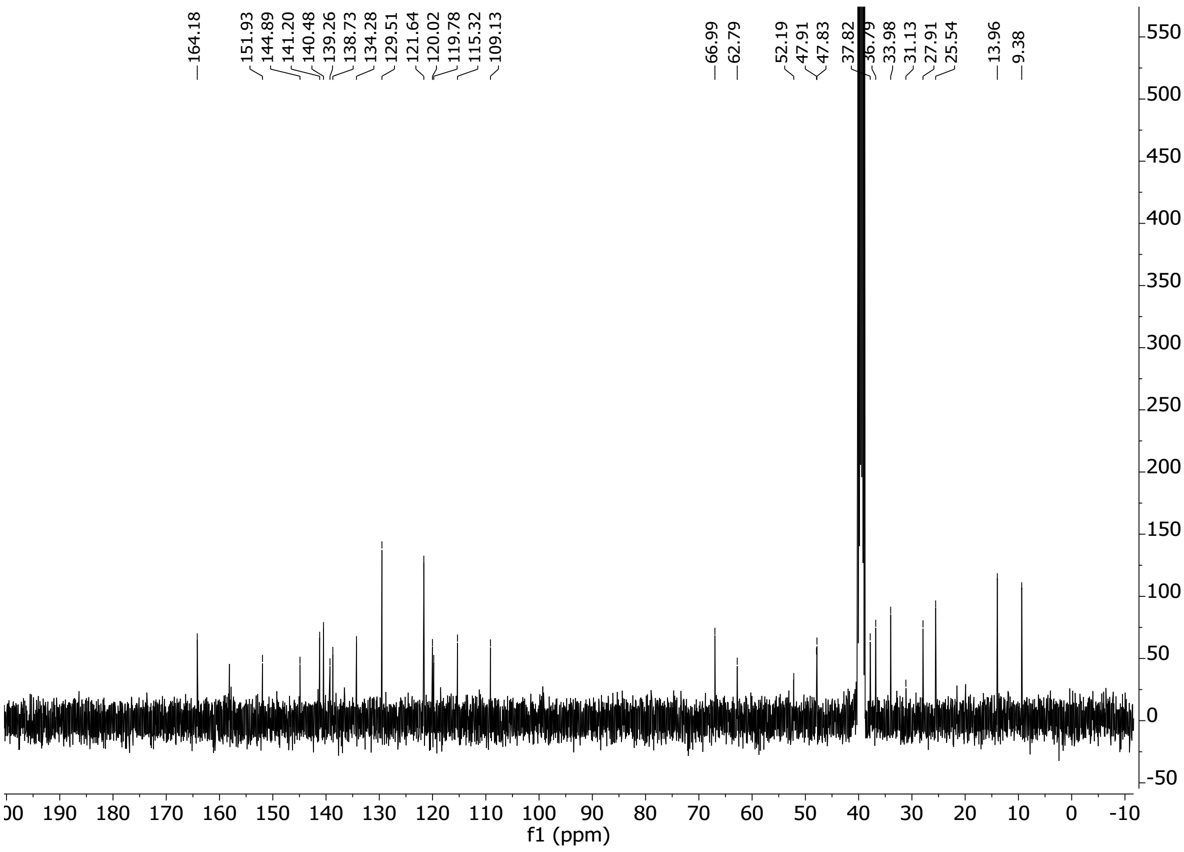
164.18 151.93 144.89 141.20 140.48 139.26 138.73 134.28 129.51 121.64 120.02 119.78 115.32 109.13
66.99 62.79 52.19 47.91 47.83 37.82 36.79 33.98 31.13 27.91 25.54 13.96 9.38
190 180 170 160 150 140 130 120 110 100 90 80 70 60 50 40 30 20 10 0 -10
f1 (ppm)
550 500 450 400 350 300 250 200 150 100 50 0 -50

Compound **6**

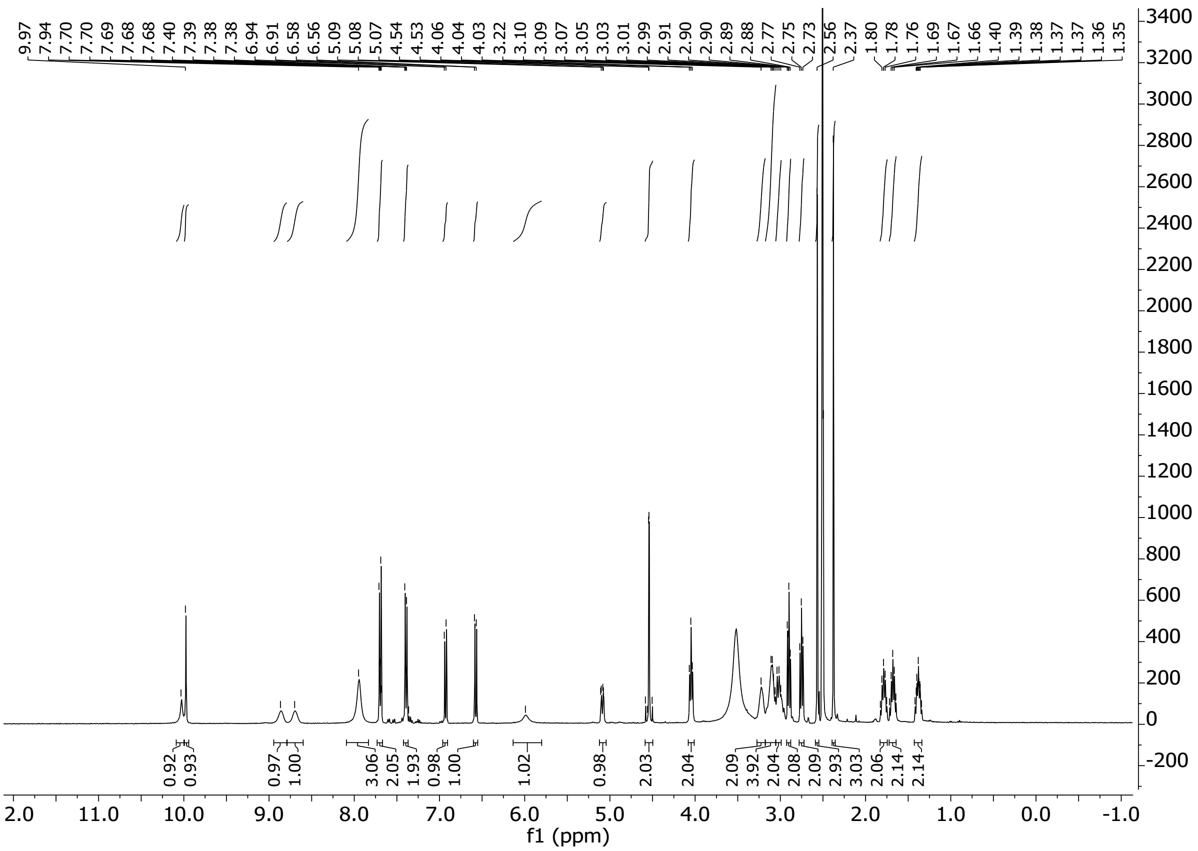

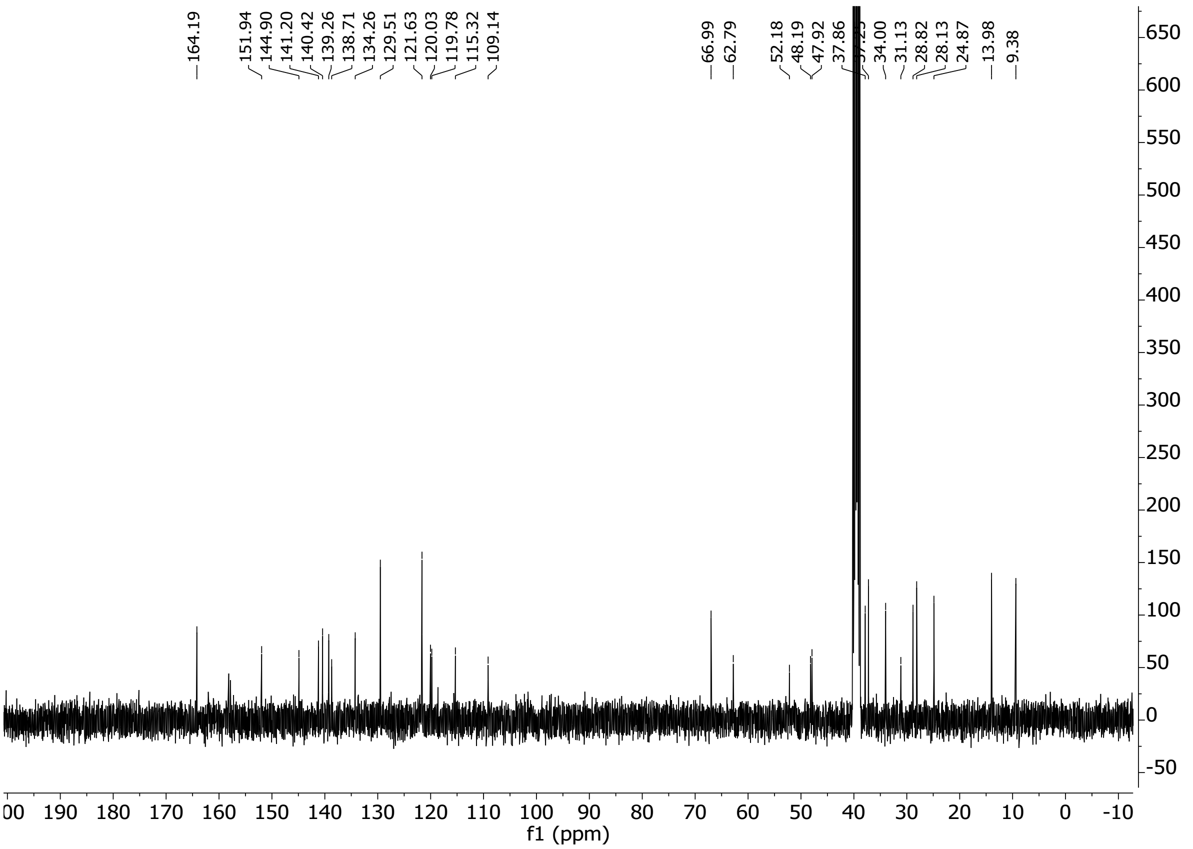

Compound **42**

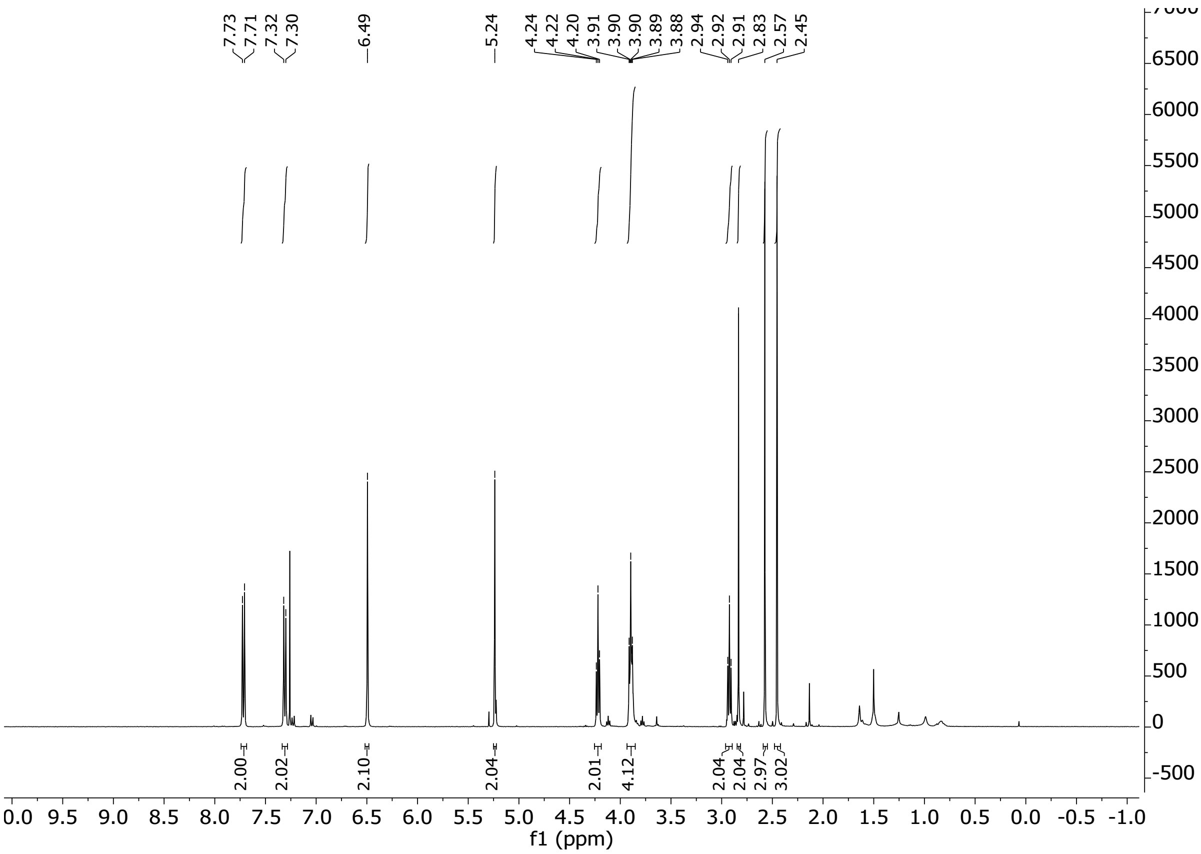

7.73
7.71
7.32
7.30
6.49
5.24
4.24
4.22
4.20
3.91
3.90
3.90
3.89
3.88
2.94
2.92
2.91
2.83
2.57
2.45
2.00
2.02
2.10
2.04
2.01
4.12
2.04
2.04
2.97
3.02
f1 (ppm)

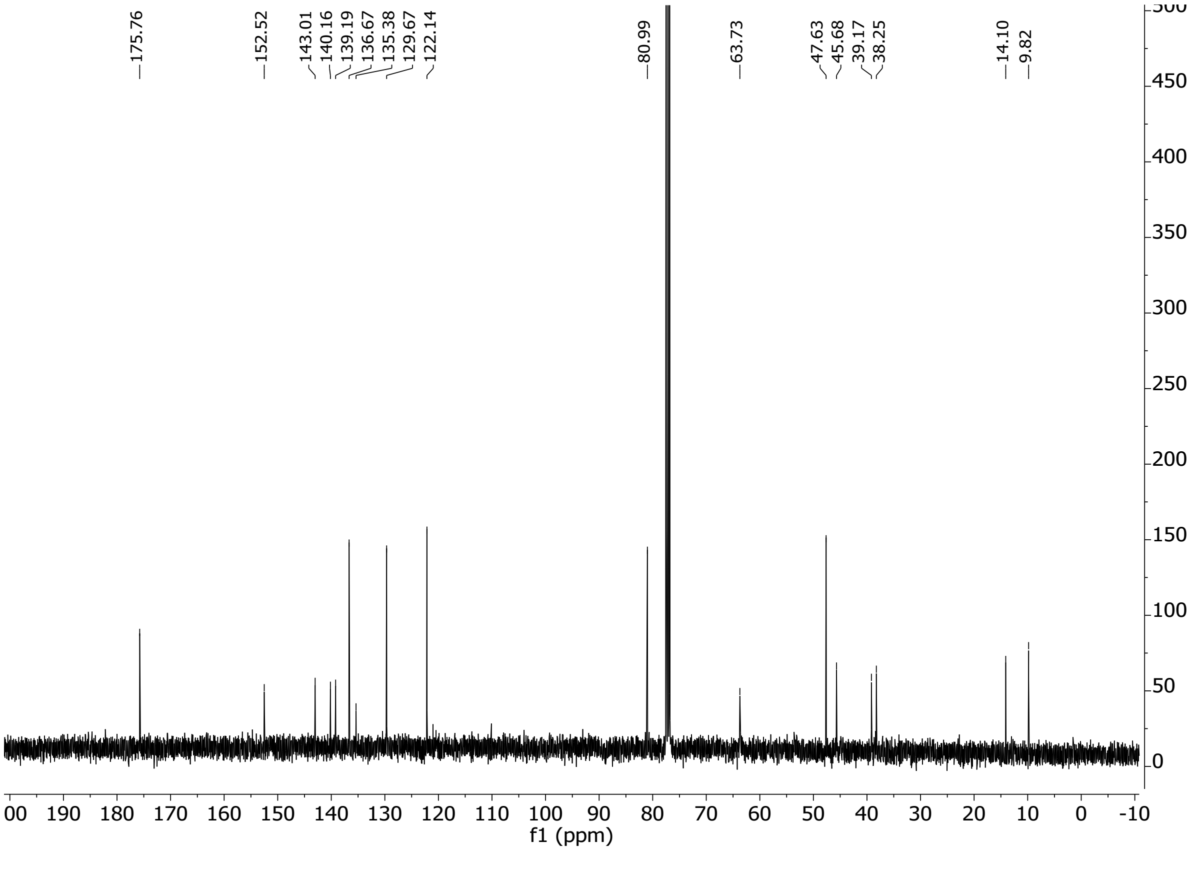

175.76
152.52
143.01
140.16
139.19
136.67
135.38
129.67
122.14
80.99
63.73
47.63
45.68
39.17
38.25
14.10
9.82
f1 (ppm)

Compound **43**

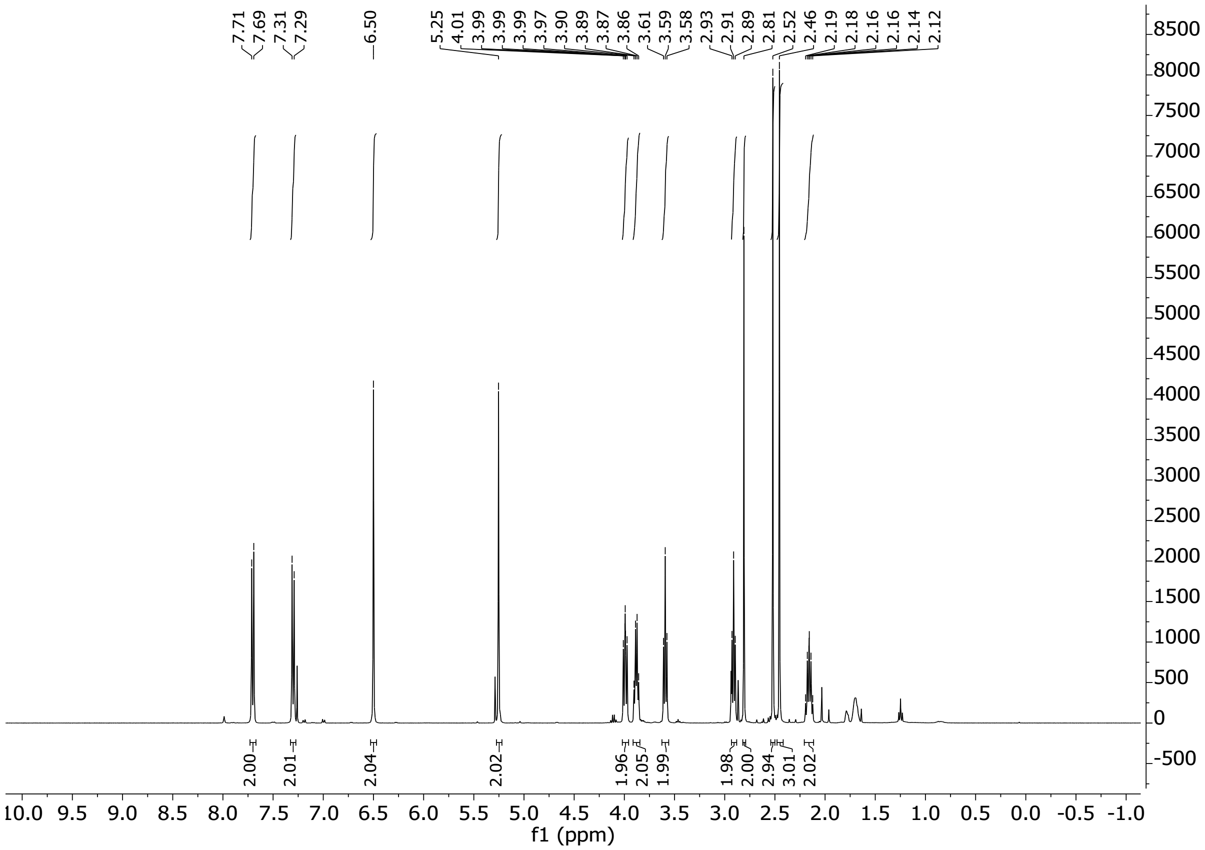

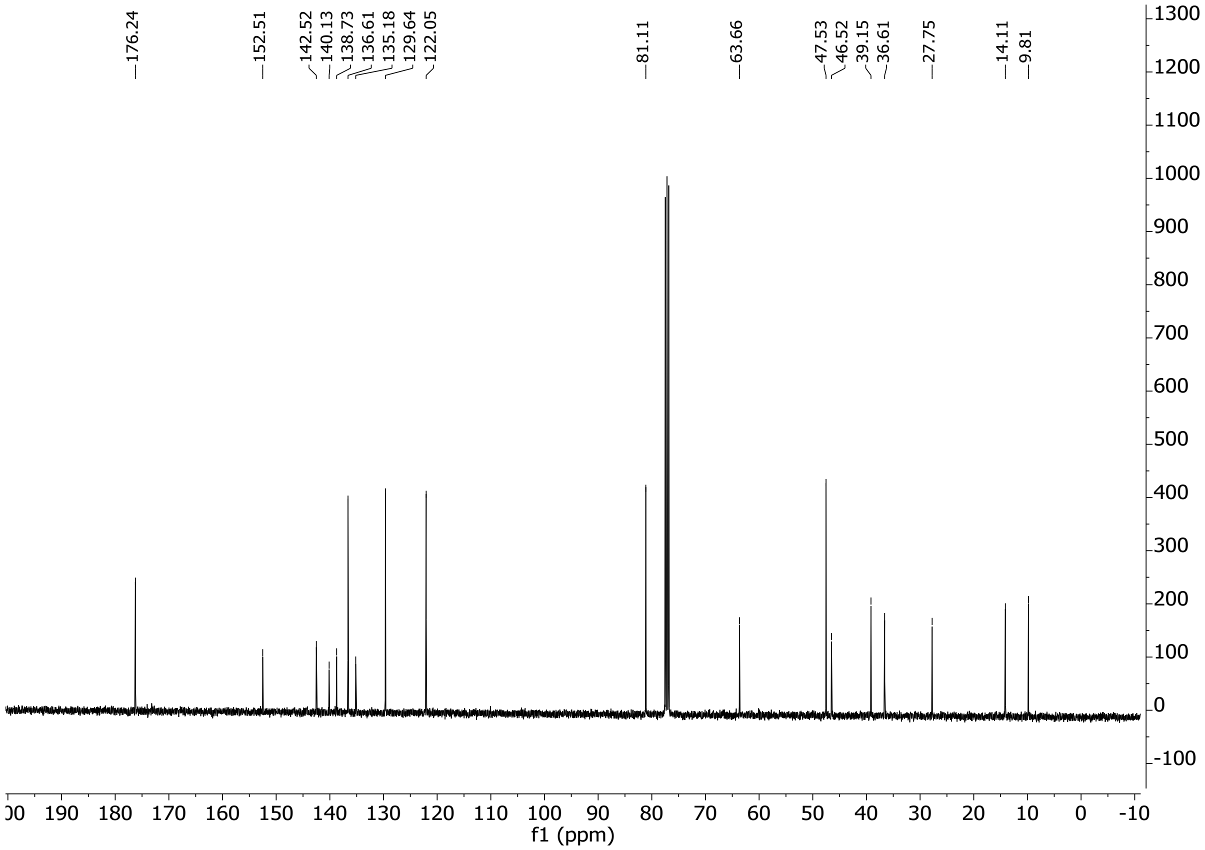

Compound **46**

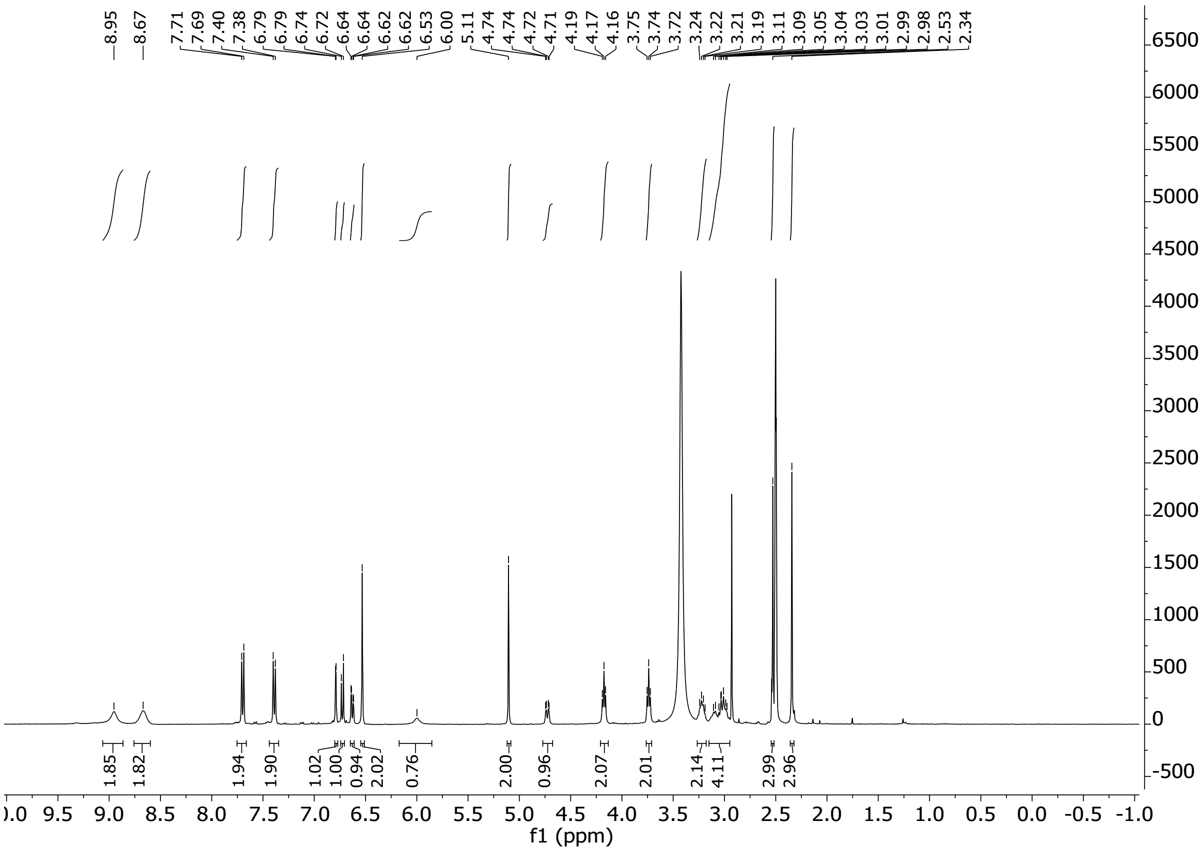

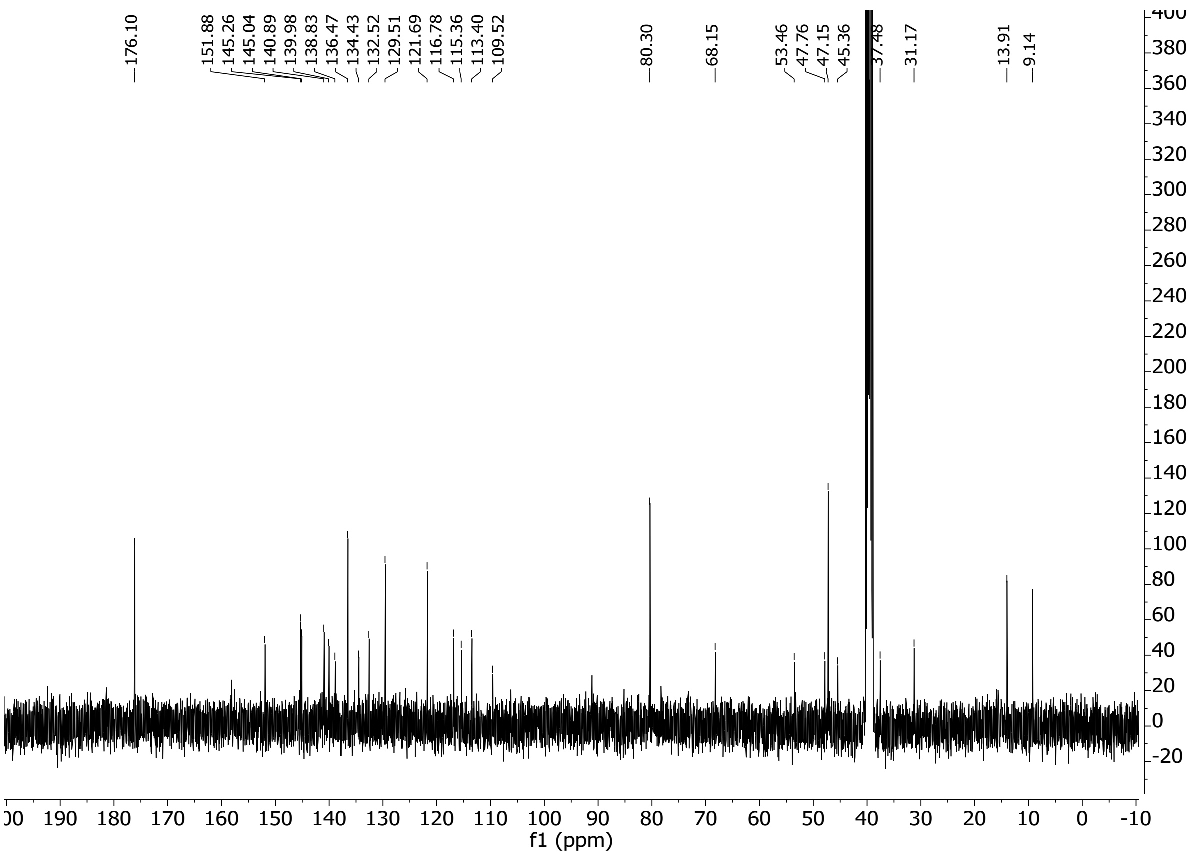

Compound **47**

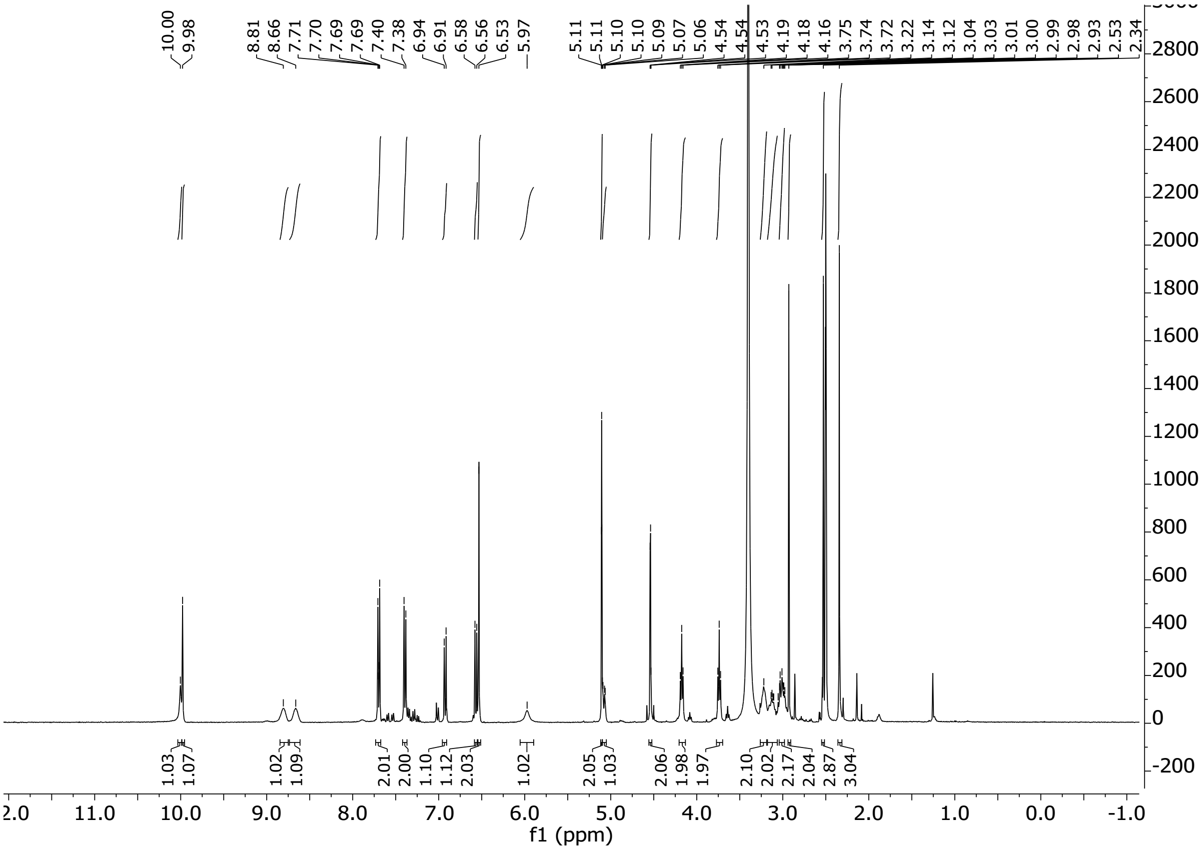

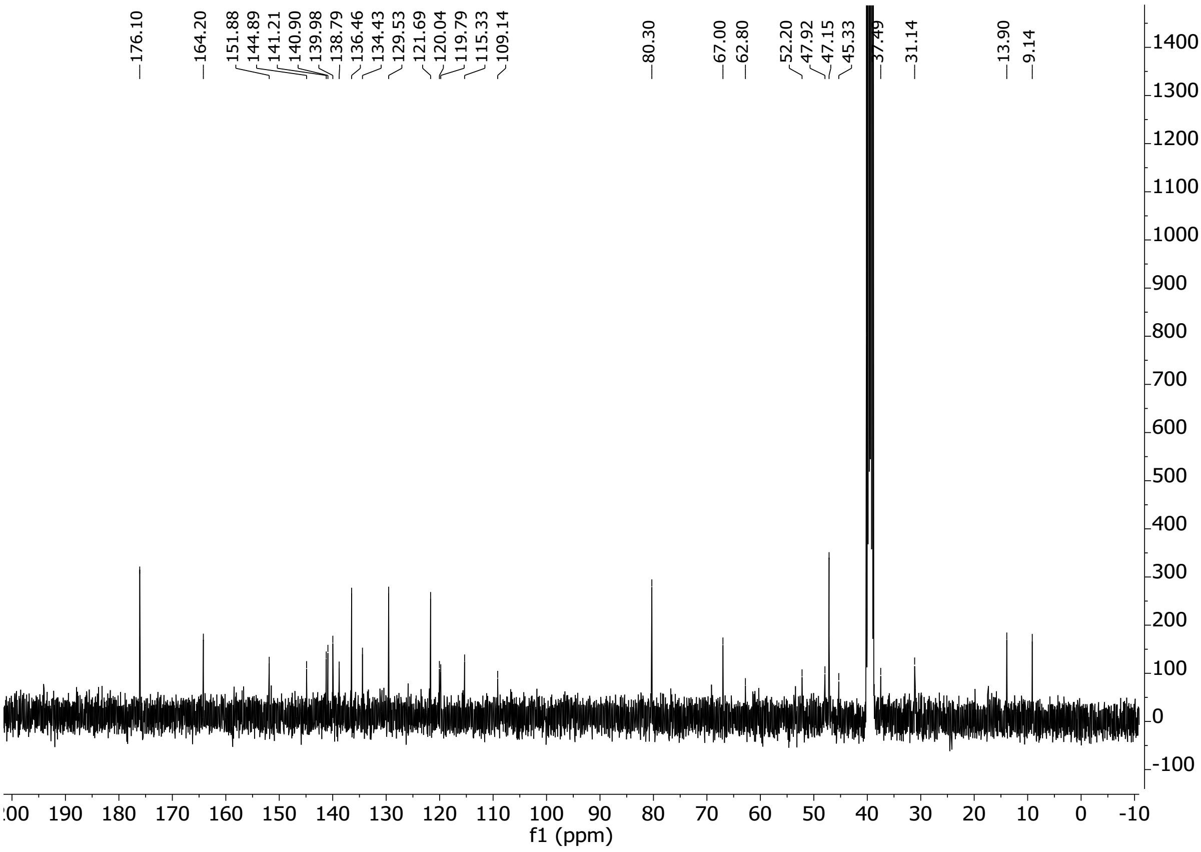

Compound **48**

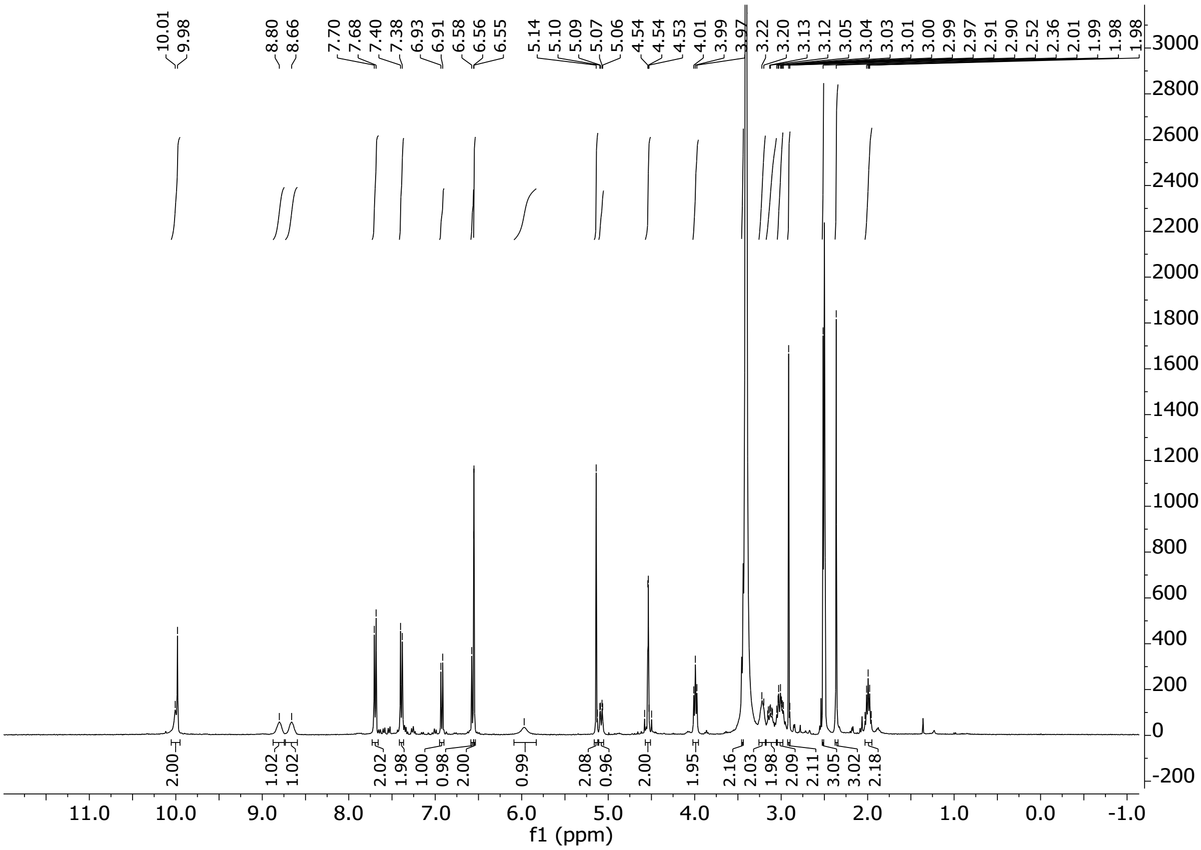

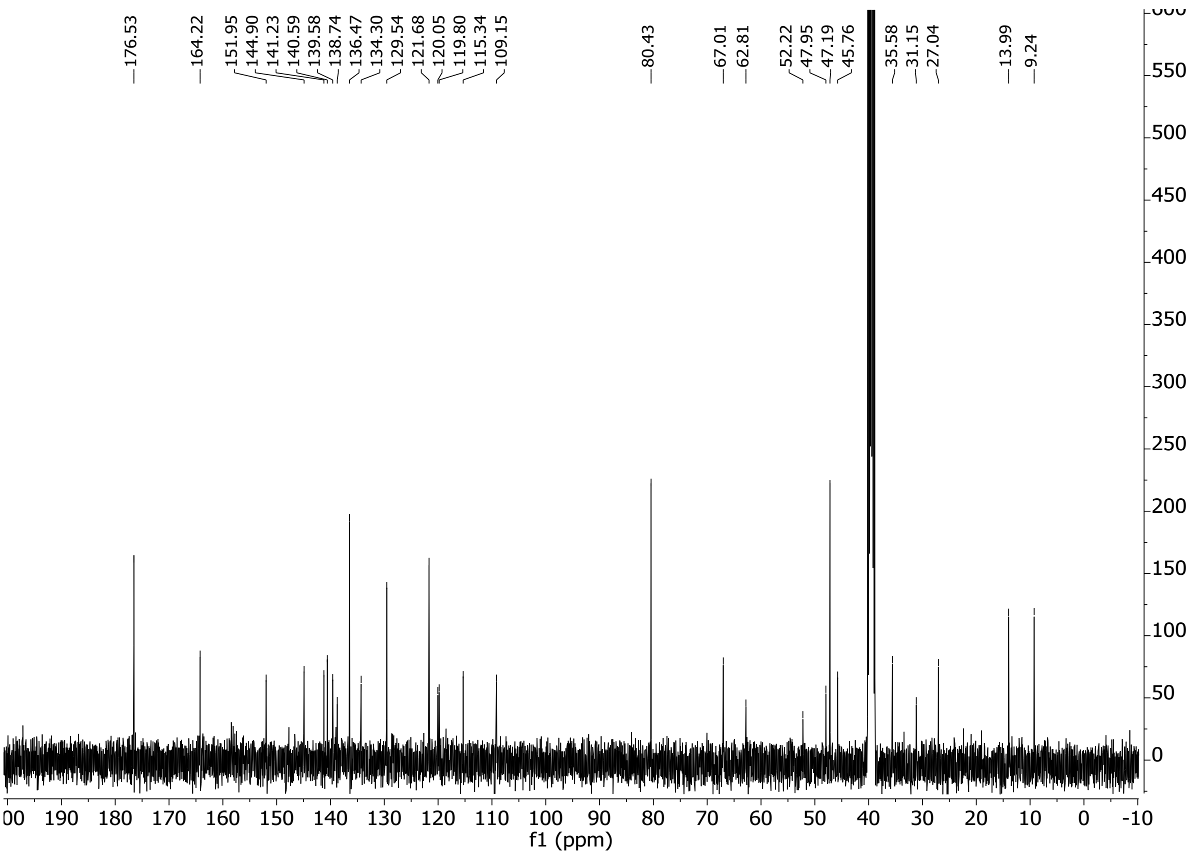

Compound **7**

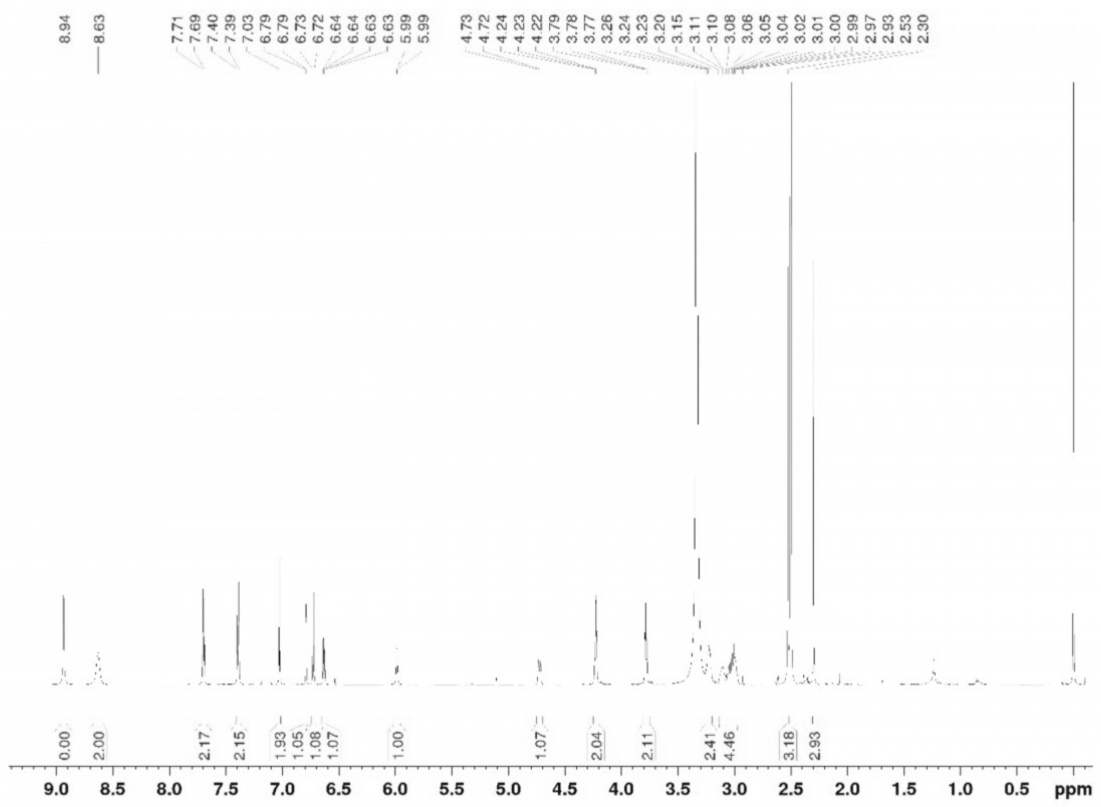

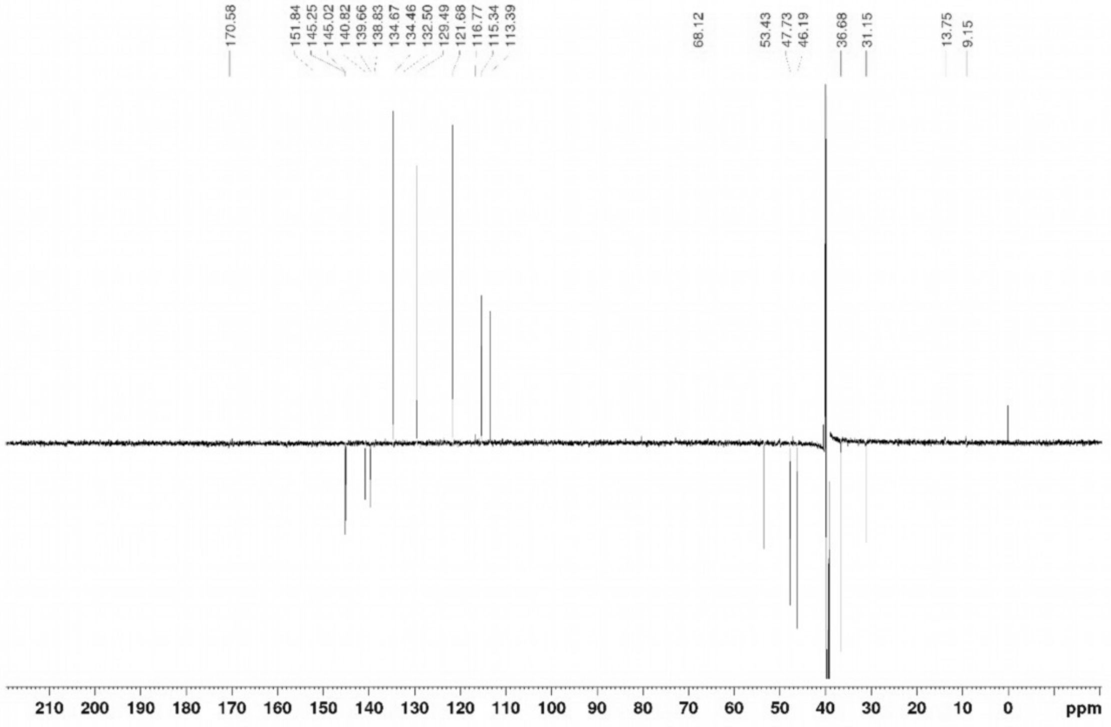

Compound **8**

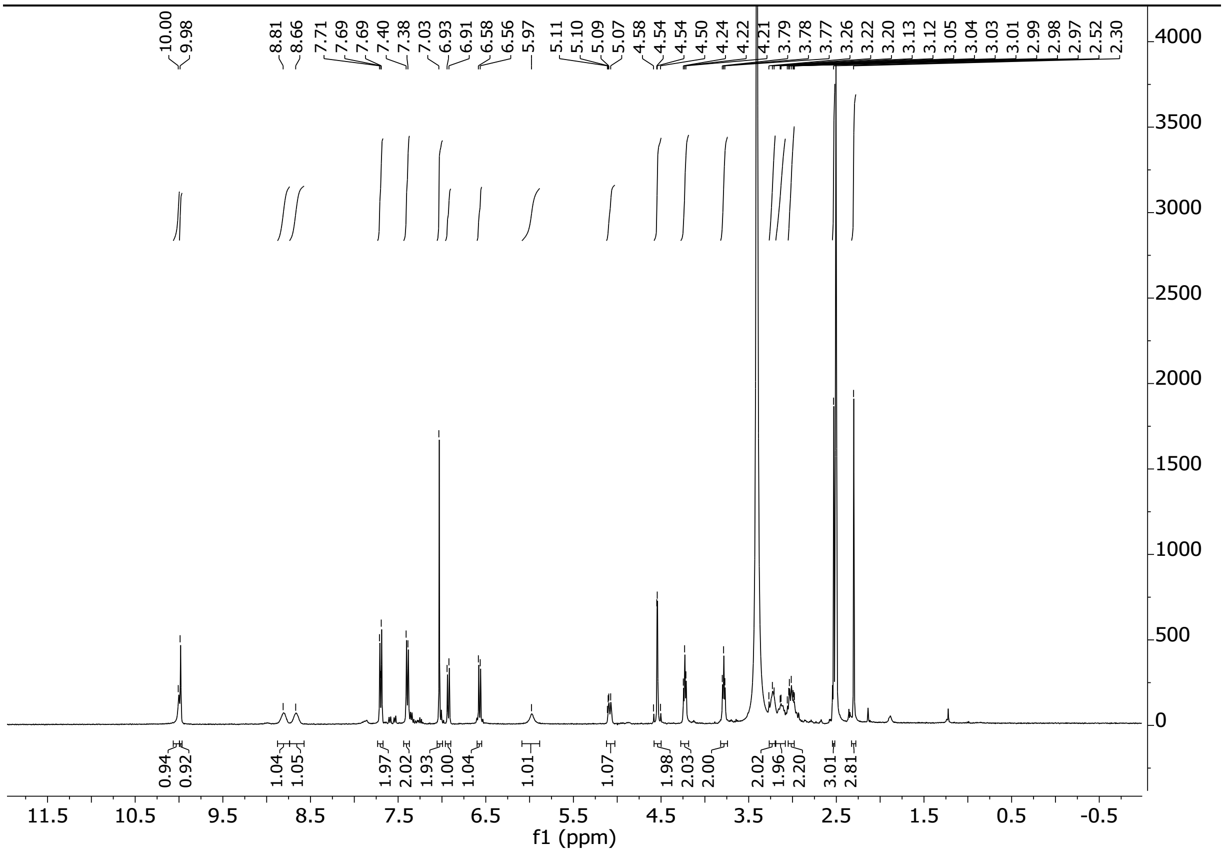

10.00
9.98
8.81
8.66
7.71
7.69
7.69
7.40
7.38
7.03
6.93
6.91
6.58
6.56
5.97
5.11
5.10
5.09
5.07
4.58
4.54
4.54
4.50
4.24
4.22
4.21
3.79
3.78
3.77
3.26
3.22
3.20
3.13
3.12
3.05
3.04
3.03
3.01
2.99
2.98
2.97
2.52
2.30
0.94
0.92
1.04
1.05
1.97
2.02
1.93
1.00
1.04
1.01
1.07
1.98
2.03
2.00
2.02
1.96
2.20
3.01
2.81
f1 (ppm)

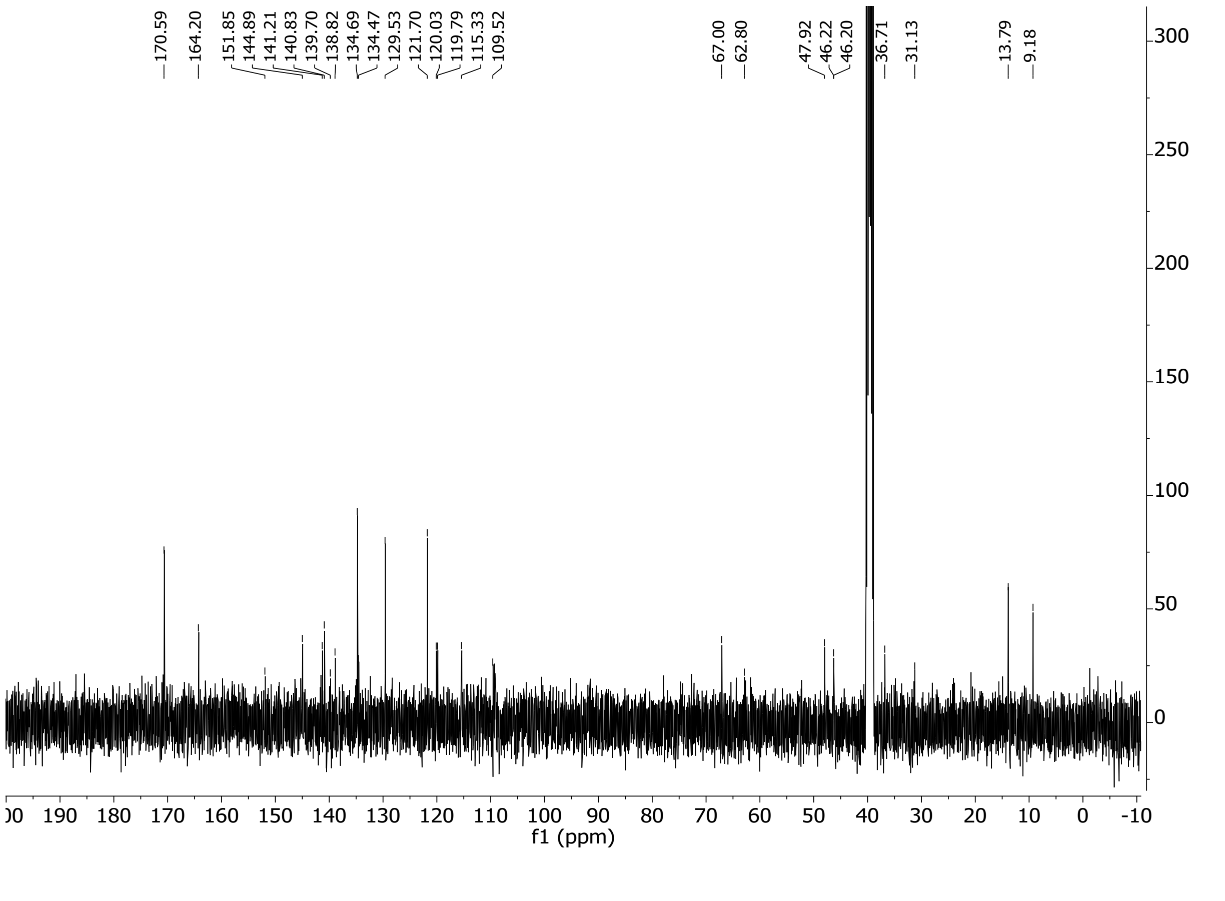

170.59
164.20
151.85
144.89
141.21
140.83
139.70
138.82
134.69
134.47
129.53
121.70
120.03
119.79
115.33
109.52
67.00
62.80
47.92
46.22
46.20
36.71
31.13
13.79
9.18
f1 (ppm)

Compound **9**

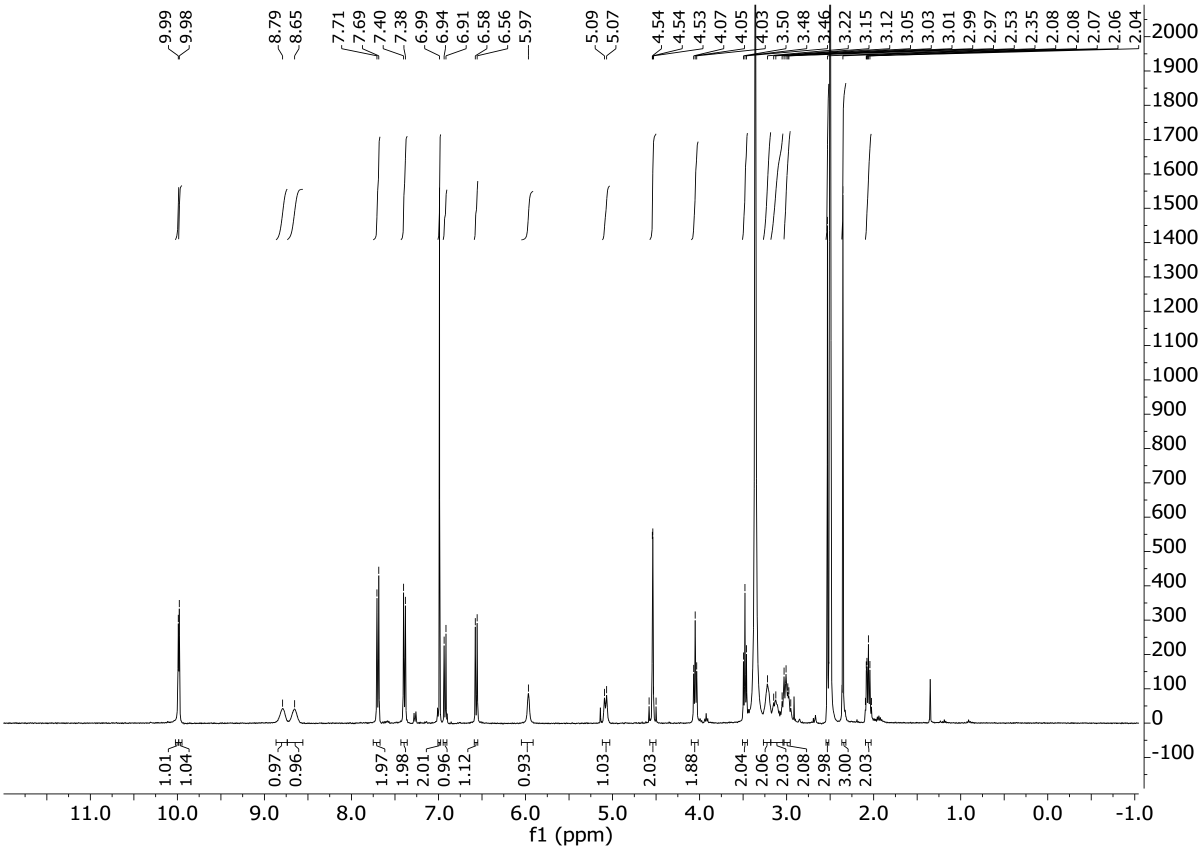

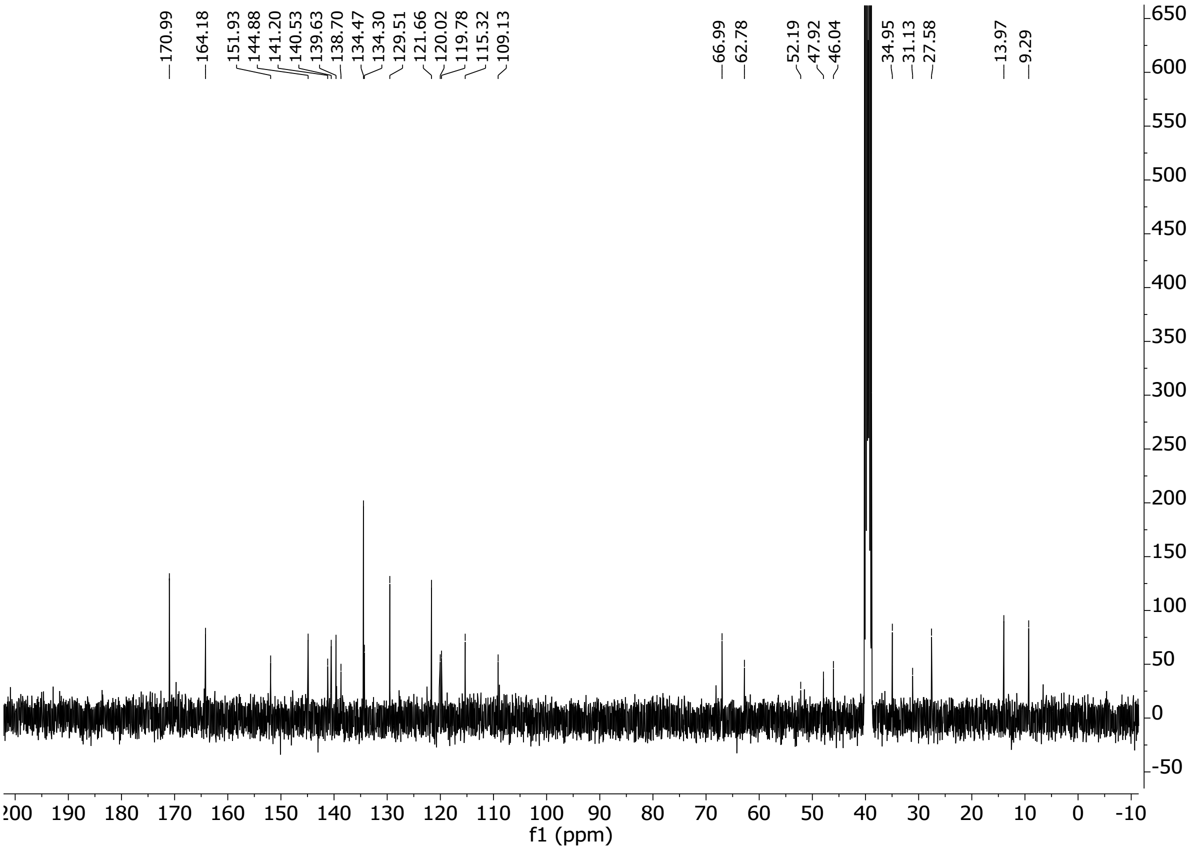

6. References

[1] C. Bergsdorf, C. Kropp-Goerkis, I. Kaehler, L. Ketscher, U. Boemer, K. Parczyk, B. Bader, *Assay Drug Dev. Technol.* **2008**, *6*, 39-53.

[2] Y. Namkung, C. Le Gouill, V. Lukashova, H. Kobayashi, M. Hogue, E. Khoury, M. Song, M. Bouvier, S. A. Laporte, *Nat. Commun.* **2016**, *7*, 12178.

[3] A. M. Ring, A. Manglik, A. C. Kruse, M. D. Enos, W. I. Weis, K. C. Garcia, B. K. Kobilka, *Nature* **2013**, *502*, 575-579.

[4] E. F. Pettersen, T. D. Goddard, C. C. Huang, G. S. Couch, D. M. Greenblatt, E. C. Meng, T. E. Ferrin, *J. Comput. Chem.* **2004**, *25*, 1605-1612.

[5] M. V. Shapovalov, R. L. Dunbrack, Jr, *Structure* **2011**, *19*, 844-858.

[6] D. M. Rosenbaum, C. Zhang, J. A. Lyons, R. Holl, D. Aragao, D. H. Arlow, S. G. F. Rasmussen, H.-J. Choi, B. T. DeVree, R. K. Sunahara, P. S. Chae, S. H. Gellman, R. O. Dror, D. E. Shaw, W. I. Weis, M. Caffrey, P. Gmeiner, B. K. Kobilka, *Nature* **2011**, *469*, 236-240.

[7] V. Katritch, G. Fenalti, E. E. Abola, B. L. Roth, V. Cherezov, R. C. Stevens, *Trends Biochem. Sci.* **2014**, *39*, 233-244.

[8] K. Ihara, M. Hato, T. Nakane, K. Yamashita, T. Kimura-Someya, T. Hosaka, Y. Ishizuka-Katsura, R. Tanaka, T. Tanaka, M. Sugahara, K. Hirata, M. Yamamoto, O. Nureki, K. Tono, E. Nango, S. Iwata, M. Shirouzu, *Sci. Rep.* **2020**, *10*, 19305.

[9] A. Šali, T. L. Blundell, *J. Mol. Biol.* **1993**, *234*, 779-815.

[10] Schrödinger Release 2022-1: Maestro, Schrödinger, LLC, New York, NY, 2022.

[11] R. O. Dror, D. H. Arlow, D. W. Borhani, M. Ø. Jensen, S. Piana, D. E. Shaw, *Proc. Natl. Acad. Sci. U. S. A.* **2009**, *106*, 4689-4694.

[12] K. Zhu, K. W. Borrelli, J. R. Greenwood, T. Day, R. Abel, R. S. Farid, E. Harder, *J. Chem. Inf. Model.* **2014**, *54*, 1932-1940.

[13] D. A. Case, H. M. Aktulga, K. Belfon, D. S. Cerutti, G. A. Cisneros, V. W. D. Cruzeiro, N. Forouzesh, T. J. Giese, A. W. Götz, H. Gohlke, S. Izadi, K. Kasavajhala, M. C. Kaymak, E. King, T. Kurtzman, T.-S. Lee, P. Li, J. Liu, T. Luchko, R. Luo, M. Manathunga, M. R. Machado, H. M. Nguyen, K. A. O'Hearn, A. V. Onufriev, F. Pan, S. Pantano, R. Qi, A. Rahnamoun, A. Risheh, S. Schott-Verdugo, A. Shajan, J. Swails, J. Wang, H. Wei, X. Wu, Y. Wu, S. Zhang, S. Zhao, Q. Zhu, T. E. Cheatham, D. R. Roe, A. Roitberg, C. Simmerling, D. M. York, M. C. Nagan, K. M. Merz, *J. Chem. Inf. Model.* **2023**, *63*, 6183-6191.

[14] D.A. Case, H.M. Aktulga, K. Belfon, I.Y. Ben-Shalom, J.T. Berryman, S.R. Brozell, D.S. Cerutti, T.E. Cheatham, III, G.A. Cisneros, V.W.D. Cruzeiro, T.A. Darden, R.E. Duke, G. Giambasu, M.K. Gilson, H. Gohlke, A.W. Goetz, R. Harris, S. Izadi, S.A. Izmailov, K. Kasavajhala, M.C. Kaymak, E. King, A. Kovalenko, T. Kurtzman, T.S. Lee, S. LeGrand, P. Li, C. Lin, J. Liu, T. Luchko, R. Luo, M. Machado, V. Man, M. Manathunga, K.M. Merz, Y. Miao, O. Mikhailovskii, G. Monard, H. Nguyen, K.A. O'Hearn, A. Onufriev, F. Pan, S. Pantano, R. Qi, A. Rahnamoun, D.R. Roe, A. Roitberg, C. Sagui, S. Schott-Verdugo, A. Shajan, J. Shen, C.L. Simmerling, N.R. Skrynnikov, J. Smith, J. Swails, R.C. Walker, J. Wang, J. Wang, H. Wei, R.M. Wolf, X. Wu, Y. Xiong, Y. Xue, D.M. York, S. Zhao, and P.A. Kollman (2022), Amber 2022, University of California, San Francisco.

[15] M. R. Shirts, C. Klein, J. M. Swails, J. Yin, M. K. Gilson, D. L. Mobley, D. A. Case, E. D. Zhong, *J. Comput. Aided Mol. Des.* **2017**, *31*, 147-161.

[16] M. A. Lomize, I. D. Pogozheva, H. Joo, H. I. Mosberg, A. L. Lomize, *Nucleic Acids Res.* **2012**, *40*, D370-D376.

[17] C.-Y. Huang, V. Olieric, P. Ma, N. Howe, L. Vogeley, X. Liu, R. Warshamanage, T. Weinert, E. Panepucci, B. Kobilka, K. Diederichs, M. Wang, M. Caffrey, *Acta Crystallogr. Sect. D* **2016**, *72*, 93-112.

[18] M. G. Wolf, M. Hoefling, C. Aponte-Santamaría, H. Grubmuller, G. Groenhof, *J. Comput. Chem.* **2010**, *31*, 2169-2174.
[19] J. Wang, R. M. Wolf, J. W. Caldwell, P. A. Kollman, D. A. Case, *J. Comput. Chem.* **2004**, *25*, 1157-1174.
[20] C. J. Dickson, B. D. Madej, Å. A. Skjevik, R. M. Betz, K. Teigen, I. R. Gould, R. C. Walker, *J. Chem. Theory Comput.* **2014**, *10*, 865-879.
[21] J. A. Maier, C. Martinez, K. Kasavajhala, L. Wickstrom, K. E. Hauser, C. Simmerling, *J. Chem. Theory Comput.* **2015**, *11*, 3696-3713.
[22] H. J. C. Berendsen, J. R. Grigera, T. P. Straatsma, *J. Phys. Chem.* **1987**, *91*, 6269-6271.
[23] J. Wang, W. Wang, P. A. Kollman, D. A. Case, *J. Mol. Graphics Modell.* **2006**, *25*, 247-260.
[24] M. J. Frisch, G. W. Trucks, H. B. Schlegel, G. E. Scuseria, M. A. Robb, J. R. Cheeseman, G. Scalmani, V. Barone, G. A. Petersson, H. Nakatsuji, X. Li, M. Caricato, A. V. Marenich, J. Bloino, B. G. Janesko, R. Gomperts, B. Mennucci, H. P. Hratchian, J. V. Ortiz, A. F. Izmaylov, J. L. Sonnenberg, Williams, F. Ding, F. Lipparini, F. Egidi, J. Goings, B. Peng, A. Petrone, T. Henderson, D. Ranasinghe, V. G. Zakrzewski, J. Gao, N. Rega, G. Zheng, W. Liang, M. Hada, M. Ehara, K. Toyota, R. Fukuda, J. Hasegawa, M. Ishida, T. Nakajima, Y. Honda, O. Kitao, H. Nakai, T. Vreven, K. Throssell, J. A. Montgomery Jr., J. E. Peralta, F. Ogliaro, M. J. Bearpark, J. J. Heyd, E. N. Brothers, K. N. Kudin, V. N. Staroverov, T. A. Keith, R. Kobayashi, J. Normand, K. Raghavachari, A. P. Rendell, J. C. Burant, S. S. Iyengar, J. Tomasi, M. Cossi, J. M. Millam, M. Klene, C. Adamo, R. Cammi, J. W. Ochterski, R. L. Martin, K. Morokuma, O. Farkas, J. B. Foresman, D. J. Fox, Gaussian 16, Revision B.01, Gaussian, Inc., Wallingford (CT), **2016**.
[25] C. I. Bayly, P. Cieplak, W. Cornell, P. A. Kollman, *J. Phys. Chem.* **1993**, *97*, 10269-10280.
[26] a) D. Van Der Spoel, E. Lindahl, B. Hess, G. Groenhof, A. E. Mark, H. J. C. Berendsen, *J. Comput. Chem.* **2005**, *26*, 1701-1718; b) B. Hess, C. Kutzner, D. van der Spoel, E. Lindahl, *J. Chem. Theory Comput.* **2008**, *4*, 435-447; c) M. J. Abraham, T. Murtola, R. Schulz, S. Páll, J. C. Smith, B. Hess, E. Lindahl, *SoftwareX* **2015**, *1-2*, 19-25.
[27] G. Bussi, D. Donadio, M. Parrinello, *J. Chem. Phys.* **2007**, *126*, 014101.
[28] M. Bernetti, G. Bussi, *J. Chem. Phys.* **2020**, *153*, 114107.
[29] B. Hess, H. Bekker, H. J. C. Berendsen, J. G. E. M. Fraaije, *J. Comput. Chem.* **1997**, *18*, 1463-1472.
[30] T. Darden, D. York, L. Pedersen, *J. Chem. Phys.* **1993**, *98*, 10089-10092.
[31] The PyMOL Molecular Graphics System, Version 2.4.1 Schrödinger, LLC, New York, NY, 2020.
[32] W. Humphrey, A. Dalke, K. Schulten, *J. Mol. Graphics* **1996**, *14*, 33-38.
[33] J. D. Hunter, *Comput. Sci. Eng.* **2007**, *9*, 90-95.
[34] M. Waskom, *J. Open Source Softw.* **2021**, *6*, 3021.
[35] The PLUMED consortium, *Nat. Methods* **2019**, *16*, 670-673.
[36] G. A. Tribello, M. Bonomi, D. Branduardi, C. Camilloni, G. Bussi, *Comput. Phys. Commun.* **2014**, *185*, 604-613.
[37] a) N. Saleh, P. Ibrahim, G. Saladino, F. L. Gervasio, T. Clark, *J. Chem. Inf. Model.* **2017**, *57*, 1210-1217; b) X. Xu, J. Kaindl, M. J. Clark, H. Hübner, K. Hirata, R. K. Sunahara, P. Gmeiner, B. K. Kobilka, X. Liu, *Cell Res.* **2021**, *31*, 569-579.
[38] a) A. Laio, M. Parrinello, *Proc. Natl. Acad. Sci. U. S. A.* **2002**, *99*, 12562-12566; b) A. Barducci, G. Bussi, M. Parrinello, *Phys. Rev. Lett.* **2008**, *100*, 020603.
[39] a) V. Limongelli, M. Bonomi, M. Parrinello, *Proc. Natl. Acad. Sci. U. S. A.* **2013**, *110*, 6358-6363; b) S. Raniolo, V. Limongelli, *Nat. Protoc.* **2020**, *15*, 2837-2866.

[40] P. Raiteri, A. Laio, F. L. Gervasio, C. Micheletti, M. Parrinello, *J. Phys. Chem. B* **2006**, *110*, 3533-3539.
[41] J. MacQueen in *Proceedings of the Fifth Berkeley Symposium on Mathematical Statistics and Probability., Vol. 1* (Eds.: L. M. Le Cam , J. Neyman), University of California Press, Berkeley and Los Angeles, **1967**, pp. 281-297.
[42] P. D. Dixit, J. Wagoner, C. Weistuch, S. Pressé, K. Ghosh, K. A. Dill, *J. Chem. Phys.* **2018**, *148*, 010901.
[43] B. E. Husic, V. S. Pande, *J. Am. Chem. Soc.* **2018**, *140*, 2386-2396.
[44] P. Tiwary, M. Parrinello, *J. Phys. Chem. B* **2015**, *119*, 736-742.
[45] D. Meral, D. Provasi, M. Filizola, *J. Chem. Phys.* **2018**, *149*, 224101.
[46] F. Noé, C. Schütte, E. Vanden-Eijnden, L. Reich, T. R. Weikl, *Proc. Natl. Acad. Sci. U. S. A.* **2009**, *106*, 19011-19016.
